# A taxonomic revision of the Neotropical genus *Cremastosperma* (Annonaceae), including five new species

**DOI:** 10.3897/phytokeys.112.24897

**Published:** 2018-11-26

**Authors:** Michael D. Pirie, Lars W. Chatrou, Paul J. M. Maas

**Affiliations:** 1 Institut für Organismische und Molekulare Evolutionsbiologie, Johannes Gutenberg-Universität, Anselm-Franz-von-Bentzelweg 9a, 55099 Mainz, Germany Johannes Gutenberg-Universität Mainz Germany; 2 Wageningen University and Research, Biosystematics Group, Droevendaalsesteeg 1, 6708 PB Wageningen, The Netherlands Wageningen University Wageningen Netherlands; 3 Ghent University, Systematic and Evolutionary Botany lab, K.L. Ledeganckstraat 35, 9000 Ghent, Belgium Ghent University Ghent Belgium; 4 Naturalis Biodiversity Center, section Botany, P.O. Box 9517, 2300 RA Leiden, The Netherlands Naturalis Biodiversity Center Wageningen Netherlands

**Keywords:** Annonaceae, *
Cremastosperma
*, endangered species, endemic species, IUCN, Neotropics, taxonomy, tropical rainforest

## Abstract

We present a taxonomic revision of *Cremastosperma*, a genus of Neotropical Annonaceae occurring in lowland to premontane wet forest, mostly in areas surrounding the Andean mountain chain. We recognise 34 species, describing five as new here: from east of the Andes, *C.brachypodum* Pirie & Chatrou, **sp. nov.** and *C.dolichopodum* Pirie & Maas, **sp. nov.**, endemic to Peru; *C.confusum* Pirie, **sp. nov.**, from southern Peru and adjacent Bolivia and Brazil; and *C.alticola* Pirie & Chatrou, **sp. nov.**, at higher elevations in northern Peru and Ecuador; and from west of the Andes, *C.osicola* Pirie & Chatrou, **sp. nov.** endemic to Costa Rica, the most northerly distributed species of the genus. We provide an identification key, document diagnostic characters and distributions and provide illustrations and extensive lists of specimens, also presenting the latter in the form of mapping data with embedded links to images available online. Of the 34 species, 22 are regional endemics. On the basis of the extent of occurrence and area of occupancy of species estimated from the distribution data, we designate IUCN threat categries for all species. Fourteen species proved to be endangered (EN) and a further one critically endangered (CR), reflecting their rarity and narrow known distributions.

## Introduction

**Figure 1.**
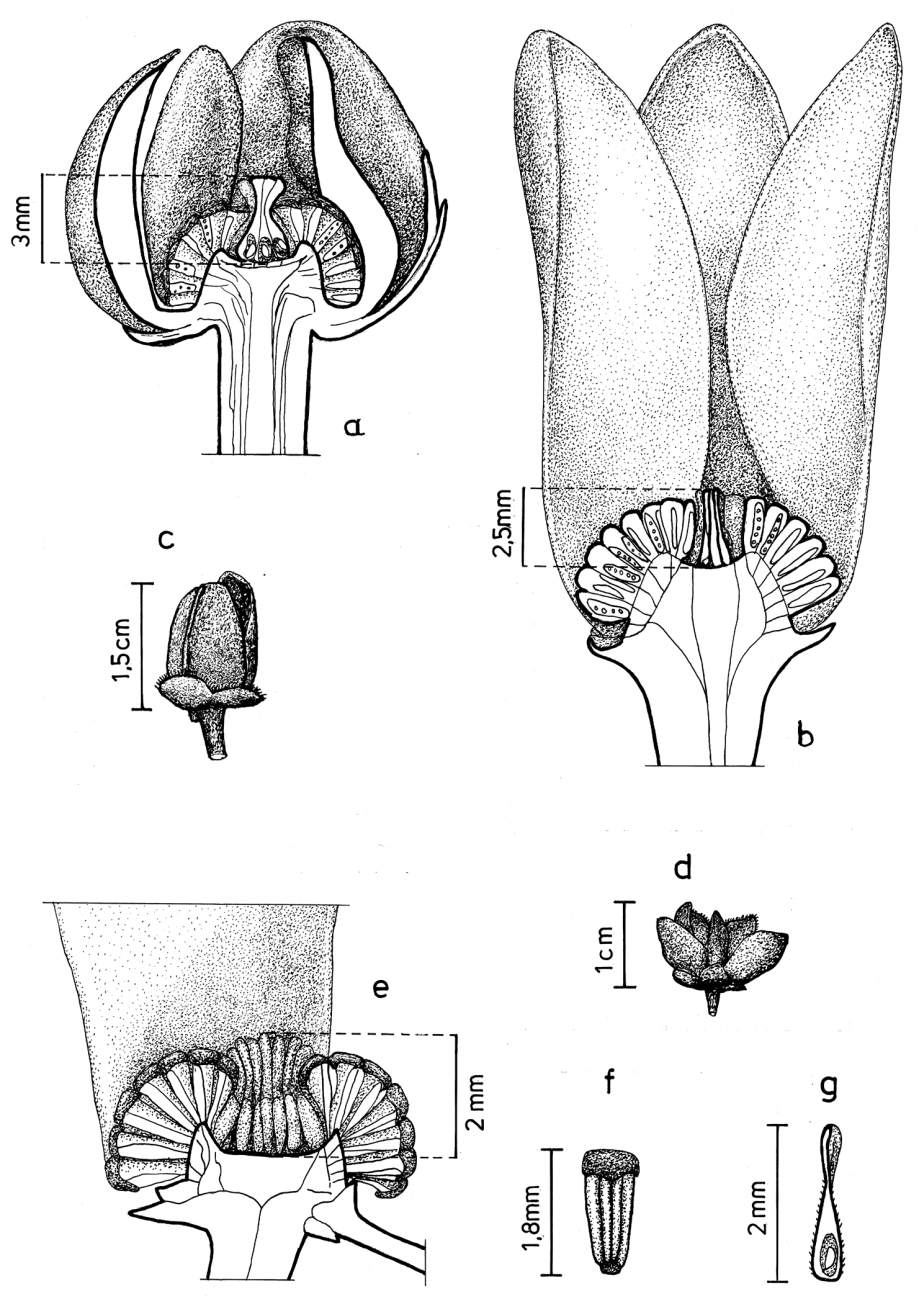
Flowers of *Cremastosperma*. Fig. 11 from van Heusden (1992). **a***C.microcarpum*: longitudinal section **b***C.oblongum* R.E.Fr.: longitudinal section **c***C.monospermum* (Rusby) R.E.Fr.: bud **d***C.gracilipes* R.E.Fr.: flower **e, f***C.cauliflorum* R.E.Fr.: longitudinal section (**e**) and stamen (**f**) **g***C.microcarpum*: carpel (**a***Maas et al. 6281***b***Maas et al. 4592***c***Nelson 763***d***Luteyn et al. 4890***e, f***Holm-Nielsen et al. 21501***g***Prance et al. 3527*).

**Figure 2.**
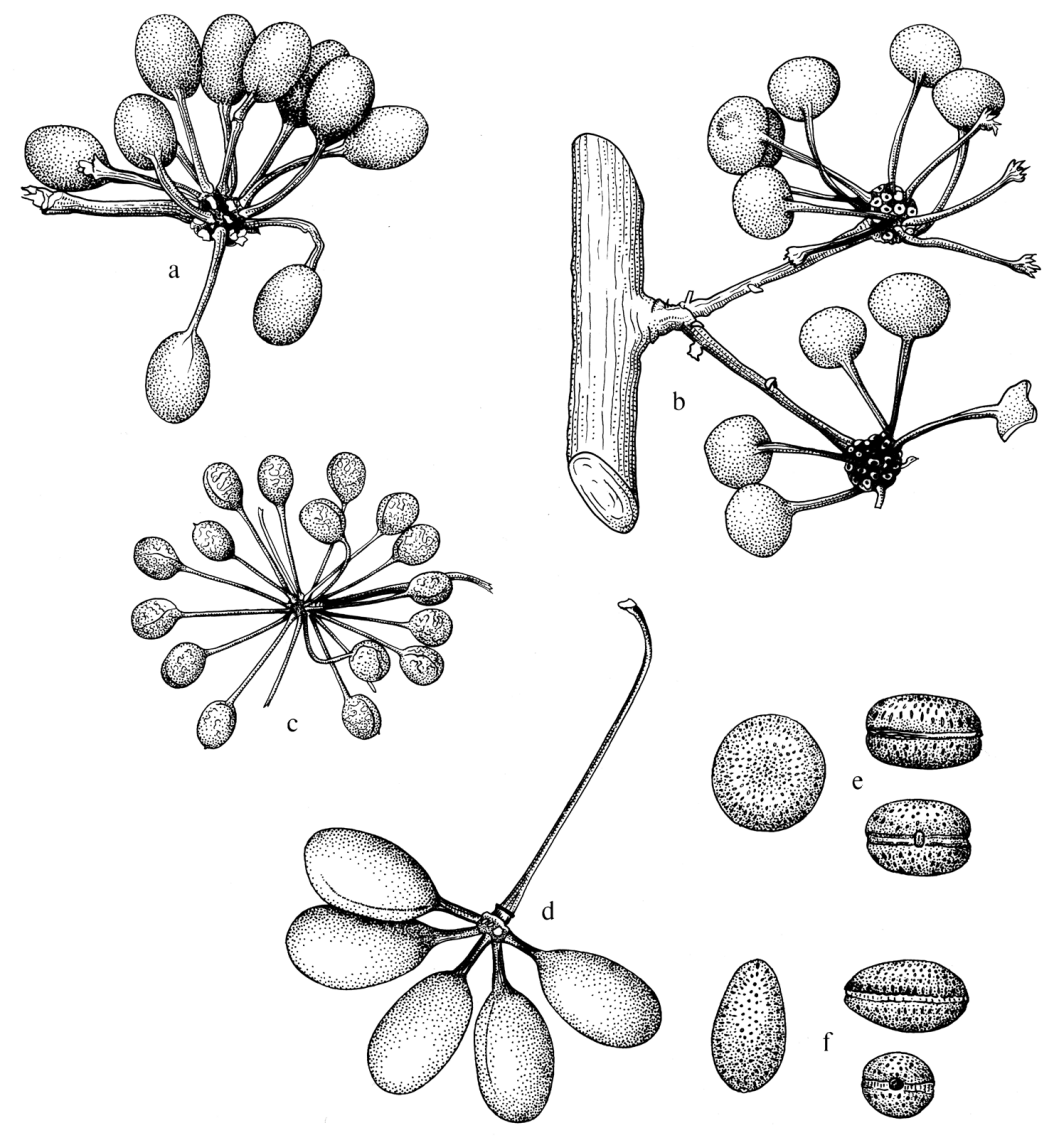
Fruits and seeds of *Cremastosperma*. Adapted from plate 8 from van Setten & Koek-Noorman (1992). **a***Cremastospermamegalophyllum* R.E.Fr. **b, e***C.cauliflorum* R.E.Fr. **c***C.microcarpum* R.E.Fr. **d***C.macrocarpum* Maas **f***C.monospermum* (Rusby) R.E.Fr. (**a***Brandbyge & Asanza C. 30017***b, e***Prance et al. 24094***c***Gentry et al. 32153***d***Wingfield & van der Werff 6751***f***Sperling et al. 6198*).

**Figure 3.**
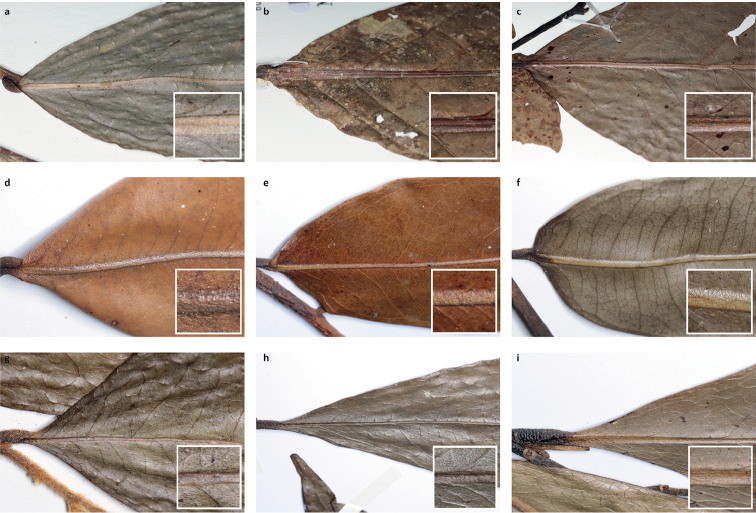
Leaf upper surfaces, particularly showing typical characteristics of the midrib: *Cremastosperma*, raised, grooved (**a***C.gracilipes* R.E.Fr., *Clark 10186***b***C.cauliflorum*, *Lewis 12049***c***C.alticola* Pirie & Chatrou, *Perea 2540*); *Pseudoxandra*, raised (**d***P.bahiensis* Maas, *Carvalho 3627***e***P.lucida* R.E.Fr., *Gentry 1854***f***P.rionegrensis* Maas, *Stevenson 898*); and *Malmea*, sunken (**g***M.surinamensis* Chatrou, *Daniëls 859*; *M.dimera* Chatrou, *Maas 9557*; *M.dielsiana* Saff. ex R.E.Fr., *Graham 2746*). Bottom right inset in each case – detail of the midrib; scales not comparable.

**Figure 4.**
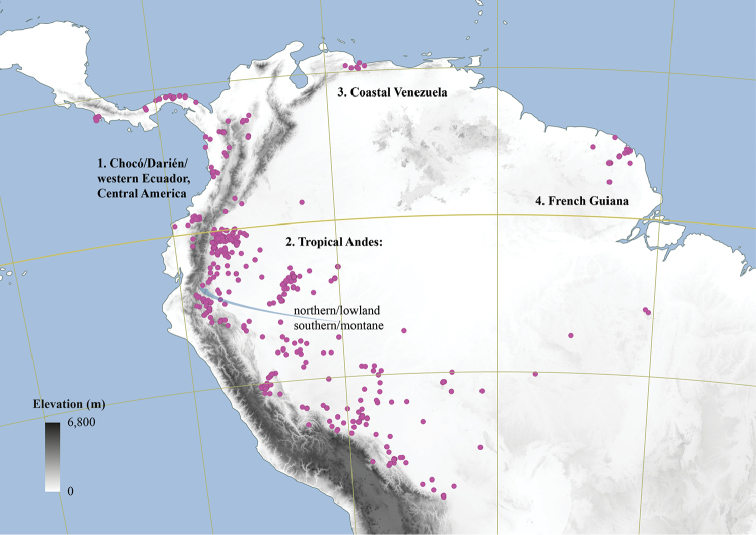
Distribution map of *Cremastosperma* showing the four disjunct areas of the distribution: **1** the Chocó/Darién/western Ecuador region north into Central America **2** the tropical Andes (including a rough demarcation between areas occupied by northern/lowland and southern/montane clades) **3** coastal Venezuela **4** French Guiana.

Arguably the most familiar representatives of the largely tropical flowering plant family Annonaceae are those with large, edible fruits, such as species of *Annona* (Pinto et al. 2005). By contrast, in *Cremastosperma* R.E.Fr. and most other Annonaceae, the individual carpels that in species of *Annona* fuse to form syncarpous fruits instead develop into one or more separate monocarps (Van Setten and Koek-Noorman 1992; Chatrou et al. 2004). Whilst Annonaceae can form large canopy trees (e.g. *Bocageopsis* R.E.Fr., *Guatteria* Ruiz & Pav.; *Hexalobus* A.DC., *Miliusa* Lesch. ex A.DC., *Oxandra* A.Rich, *Xylopia* L. (Fries 1959; Mols and Kessler 2003; Maas et al. 2007; Botermans et al. 2011; Maas et al. 2015; Junikka et al. 2016) and can exhibit spectacular floral morphologies (e.g. *Monodora* Dunal, *Uvaria* L.; Couvreur 2009; Gautier and Deroin 2013), species of *Cremastosperma* are generally somewhat inconspicuous in the field. They are small understorey trees with typical Annonaceae floral morphology (sepals and petals in whorls of three; indefinite numbers of spirally arranged stamens and carpels; Fig. 1a, b, e) bearing a resemblance to various other Neotropical genera with apocarpous, single-seeded, stipitate fruits (Fig. 2), including the much more abundant, species-rich and widespread *Guatteria* (Maas et al. 2015). The most useful, albeit somewhat obscure, character by which they can be distinguished from these and other similar Neotropical Annonaceae is displayed by the primary vein of the leaves, which is raised on the upper side with an unusual, mostly conspicuous, longitudinal groove (Fig. 3). The individual distributions of the species are restricted to four disjunct areas of lowland to pre-montane tropical forest in South and Central America: the Chocó/Darién/western Ecuador region (the narrow tropical zone to the west of the Andean mountain chain on the Pacific Ocean side of north-western South America) north into Central America; the tropical Andes (including forests on the eastern side of the Andes extending from Colombia through eastern Ecuador and Peru as far south as Bolivia); coastal Venezuela; and French Guiana (Pirie et al. 2005; Pirie et al. 2018; Fig. 4).

### Morphology, phylogeny and classification of Cremastosperma

Prior to DNA-based analyses of Annonaceae, various morphological characters were emphasised by different authors in placing *Cremastosperma* in more or less, formal classifications of Annonaceae. Van Heusden (1992) defined her *Cremastosperma* group (also including the Neotropical genera *Ephedranthus* S.Moore, *Malmea* R.E.Fr., *Oxandra*, *Pseudephedranthus* Aristeg., *Pseudoxandra* R.E.Fr., and *Ruizodendron* R.E.Fr.) on a combination of floral characters: imbricate, often ciliate sepals and petals; small sepals (rarely over 4 mm long); usually whitish or greenish, sometimes yellowish flowers; and one basal, lateral or apical ovule (Fig. 1g). The broader *Malmea* group of Walker (1971) also included the Neotropical genera *Unonopsis* R.E.Fr., *Bocageopsis* and *Onychopetalum* R.E.Fr. and the African genus *Annickia* Setten & Maas. It was characterised by solitary, medium to large, sulcate pollen grains. Group 4 of Van Setten and Koek-Noorman (1992), defined by transversely grooved or pitted seeds lacking arils, included most of the *Cremastosperma* group genera of Van Heusden plus a number of Asian taxa (but neither *Unonopsis*, *Bocageopsis* and *Onychopetalum*, nor *Annickia*). Amongst these taxa, *Cremastosperma* was noted to be exceptional in the combination of a pitted seed wall (Fig. 2e, f) with spiniform ruminations – a condition shared with *Pseudoxandra* and *Malmea* sensu Chatrou (1998), i.e. not including species formerly included in *Malmea* which now represent the genera *Klarobelia* Chatrou, *Mosannona* Chatrou and *Pseudomalmea* Chatrou.

**Figure 5.**
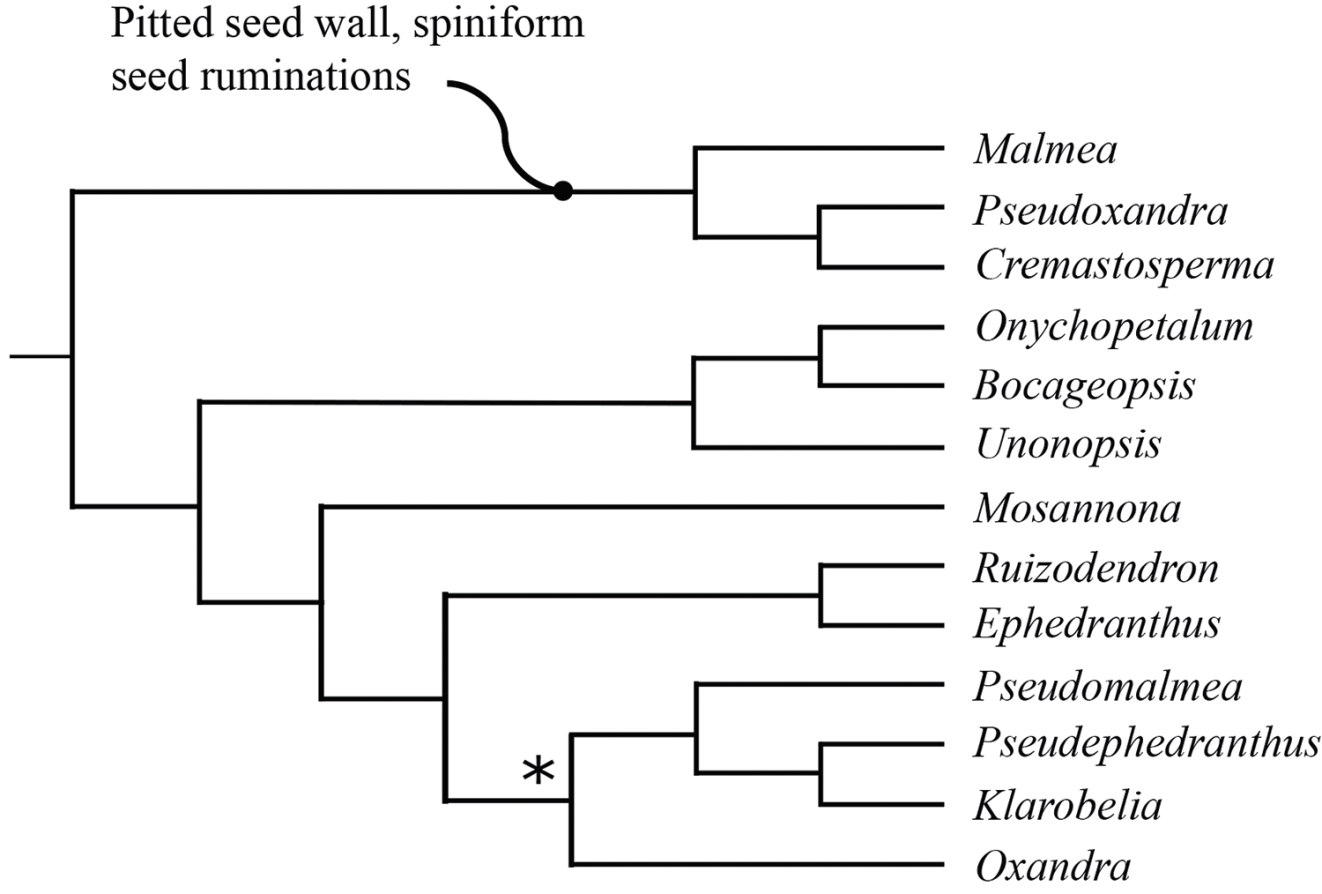
Summary phylogeny of Malmeeae, adapted from Chatrou et al. (2018). A putative synapomorphy for the the *Malmea*, *Pseudoxandra* and *Cremastosperma* clade is indicated; one node that is not subject to significant support is indicated with an asterisk.

Molecular phylogenetic studies of Annonaceae using plastid DNA sequence data (Richardson et al. 2004; Mols et al. 2004; Pirie et al. 2005, 2006; Couvreur et al. 2011; Chatrou et al. 2012; Guo et al. 2017) have shown strong support for a clade including all the above-mentioned taxa plus various African and a large number of Asian species, classified by Chatrou et al. (2012) under the subfamily Malmeoideae (Fig. 5). Pirie et al. (2006) identified a clade including all Malmeoideae genera with distributions centred in South America (the “SAC clade”; Pirie et al. 2006), which corresponded to the *Cremastosperma* group of Van Heusden (1992) plus *Unonopsis*, *Bocageopsis* and *Onychopetalum*. This SAC clade was classified formally as tribe Malmeeae by Chatrou et al. (2012) and sister group relationships between *Cremastosperma* and one or other lineage of (a not demonstrably monophyletic) *Pseudoxandra* and between the *Cremastosperma*/*Pseudoxandra* clade and *Malmea* were confirmed. The pitted seed wall (Figs 2 e and f) and spiniform seed ruminations identified by Van Setten and Koek-Noorman (1992) may thus represent synapomorphies for the *Cremastosperma*, *Pseudoxandra* and *Malmea* clade (Fig. 5).

### Taxonomic history of Cremastosperma

The name *Cremastosperma* was first introduced by Robert E. Fries (1930), based on a species originally described by Diels (1906) under the genus *Aberemoa*: *A.pedunculata* Diels (which thus became the type species *Cremastospermapedunculatum* (Diels) R.E.Fr.). The name *Cremastosperma* referred to the position of the ovules, apically attached and pendant within the ovaries (hence “hanging seeds”), as observed by Fries initially in just *A.pedunculata* (Fries 1930). Fries considered this unusual enough compared to the basally attached ovules of, for example, *Malmea* and *Guatteria* to set it aside from other genera and it proved to be largely – though not entirely – consistent in species that he subsequently described under *Cremastosperma*. Further work by Fries over the subsequent twenty years increased the number of species of *Cremastosperma* to 17. In 1931, Fries described *C.cauliflorum* R.E.Fr., *C.gracilipes* R.E.Fr. and *C.megalophyllum* R.E.Fr. and made five new combinations transferring species from *Cymbopetalum* (*C.monospermum* (Rusby) R.E.Fr.), *Guatteria* (*C.leiophyllum* (Diels) R.E.Fr., *C.pendulum* (Ruiz & Pav.) R.E.Fr., and *C.poiteaui* (Diels) R.E.Fr.) and *Unonopsis* (*C.polyphlebum* (Diels) R.E.Fr.). In 1934, he described four new species: *C.longicuspe* R.E.Fr., *C.peruvianum* R.E.Fr., *C.guianense* R.E.Fr. and *C.williamsii* R.E.Fr. (Fries, 1934); in 1937 one: *C.juruense* R.E.Fr. (in which publication he also transferred *C.guianense*, *C.polyphlebum* and *C.williamsii* to the genus *Pseudoxandra* Fries, 1937); and in 1939, one: *C.microcarpum* R.E.Fr., plus a variety of *C.monospermum*: C.monospermum(Rusby)R.E.Fr.var.brachypodum R.E.Fr. (Fries 1939). In the latter paper, he also made a new combination: *C.brevipes* (DC.) R.E.Fr., under which he brought both names *Guatteriabrevipes* DC. and *Cremastospermapoiteaui* (Diels) R.E.Fr. into synonymy. In 1948 and 1950, Fries described his last five new species of *Cremastosperma*: *C.anomalum* R.E.Fr., *C.killipii* R.E.Fr. and *C.oblongum* R.E.Fr., followed by the first species of the genus from the Pacific coast of Colombia: *C.novogranatense* R.E.Fr. and *C.pacificum* R.E.Fr. (Fries 1948, 1950).

The death of Fries in 1966 (Peterson 1966; Buchwald 1970) marked a significant hiatus in taxonomic work on Neotropical Annonaceae in general and work on *Cremastosperma* was only resumed subsequent to the establishment of the international Annonaceae project 17 years later (Maas 1983). Maas (in Maas et al. 1986) described one species from Panama (*C.panamense* Maas) and one from Venezuela (*C.macrocarpum* Maas) and transferred *C.anomalum* to *Malmea*. Notably, this latter change removed the single case of a putative *Cremastosperma* with basally attached ovules; Chatrou (1998) subsequently further transferred it to the newly described *Klarobelia*, along with a number of more distantly related species formerly placed under *Malmea*. Pirie and Zapata Cruz (2004) described three species of *Cremastosperma* from the Marañon basin in northern Peru and Pirie (in Chatrou and Pirie 2005) increased the number of species known from Venezuela from one to two. However, the greatest underestimation of species diversity appeared to be represented by collections from the Chocó/Darién/western Ecuador region. Although many new collections of *Cremastosperma* had been made since the final contribution of Fries (Fries 1950), these were predominantly on the Amazonian side of the Andean mountain chain, with far fewer in areas of north-western South America. Nevertheless, the degree of clear morphological differentiation, clear from the previously largely undetermined collections that were available, led Pirie (2005) to describe eight new species from extra-Amazonian Colombia and Ecuador plus Panama. One additional new species, endemic to Amazonian Ecuador, also published in the latter paper, brought the total number of recognised species of *Cremastosperma* to 31 (including two species, *C.killipii* and *C.juruense*, which are synonymised here under *C.longicuspe* and *C.monospermum*, respectively). In a precursor to this work, Pirie et al. (2005b) presented informal descriptions of two further putatively new species, for which the material available was at the time deemed insufficient for formal description. Here, we present a fully updated taxonomic revision of the genus including the description of these two plus a further three new species and comprehensive identification keys, plus IUCN preliminary conservation status assessments for all species.

## Materials and Methods

### Morphological descriptions and taxonomic revision

During multiple field trips since 1971 (PJMM); 1994 (LWC); and 2001 (MDP)] to localities in Brazil, Colombia, Costa Rica, Ecuador, French Guiana, Bolivia, Panama and Peru, we have observed species of *Cremastosperma*, making herbarium vouchers, taking photos, preserving flowers and fruit in alcohol and, in the last 20 years, drying leaf samples in silica gel for DNA extraction. We studied specimens representing 1037 individual collections from herbaria A, AAU, AMAZ, B, C, CAY, COL, CUZ, ECON, F, G, GB, GH, HAO, HBG, HOXA, HUA, HUT, INB, K, L, LE, LPB, M, MICH, MO, MOL, NY, OXF, P, QAME, QCA, QCNE, RB, S, U, UC, US, USM, USZ, VEN, WIS, WU and Z. We curated the collections data using BRAHMS version 7.9.9 (http://herbaria.plants.ox.ac.uk/bol/). Using BRAHMS, we exported 1) citations of specimens examined (mostly restricting these to georeferenced specimens, selecting single collections per species from a given general locality and sorting alphabetically by country, major area and first collector, then by collector number); 2) data for creating distribution maps with QGIS (https://www.qgis.org/en/site/; for which we used basemap data from the CGIAR-CSI GeoPortal http://srtm.csi.cgiar.org/ with the projection “South_America_Albers_Equal_Area_Conic” EPSG 102033); 3) a file suitable for viewing all georeferenced collections details using Google Earth (https://www.google.com/earth/; Supplementary file 1); and 4) a comprehensive table of collections data (Supplementary file 2). The latter two resources also incorporate links to images available online, at Naturalis (http://bioportal.naturalis.nl/), the Botany Collections Database of the Field Museum of Natural History (http://emuweb.fieldmuseum.org/botany/Query.php), the Virtual Herbaria database of the University of Vienna, Austria (http://herbarium.univie.ac.at/database/search.php), TROPICOS (http://www.tropicos.org/Home.aspx), JSTOR Global Plants (http://plants.jstor.org/), the Muséum national d’Histoire naturelle, Paris, France (https://science.mnhn.fr/institution/mnhn/collection/p/item/search), the Natural Sciences Institute of the National University of Colombia (http://www.biovirtual.unal.edu.co/en/) and the AAU Herbarium Database, Aarhus University, Denmark (http://www.aubot.dk/search_form.php).

Descriptions follow the form of Pirie et al. (2005b). Measurements are based mostly on dried material. Where measurements are derived from material preserved in alcohol (and dimensions therefore often greater, as the structures are not subject to water loss), these are indicated between square brackets. Unless otherwise indicated, descriptions of the indument of bracts, sepals and petals refer to that on the outer side (the petals generally being rather flat, with the largest surfaces facing in, towards the centre of the flower, or out, away from the centre). According to Chatrou (1998), the inflorescences of all Annonaceae can be considered as terminal: apparently axillary inflorescences in genera such as *Guatteria*, *Klarobelia* and *Pseudomalmea* consist of a short shoot, developing primarily from a leaf-axillary position, subtending a terminal pedicel with flower. The distinction between these two structures is in most (but not all) cases clear, demarcated by an articulation. The short shoot bears a variable number of bracts (referred to here as lower bracts) which in occasional specimens are larger and leaf-like in appearance. The short axillary shoot is described here under the convenient term ‘peduncle’, following Maas and Westra (2003).

### IUCN preliminary conservation status

We calculated the extent of occurrence (EOO) and area of occupancy (AOO) per species using ConR (Dauby et al. 2017), using these summaries in addition to the nature of the known distributions of each species to propose preliminary conservation status for all species of *Cremastosperma* following the IUCN Red List Category Criteria (IUCN 2012). Following IUCN (2012), we adopt a precautionary attitude to applying criteria. Many *Cremastosperma* species are only infrequently collected and particularly when the only known collections are from a restricted area or considerable time has elapsed since the last collection, we suggest endangered status despite the paucity of data. In general, in the absence of detailed analyses of habitat decline across the wide areas of the distributions of the different species, we assume that decline is ongoing unless it is potentially ameliorated by presence within protected areas (although these are of course not immune from potential threats; e.g. Bass et al. 2010) or where there is evience of local abundance despite a degree of disturbance (e.g. species apparently common in areas close to roads or human habitation).

### Data resources

The data underpinning the analyses reported in this paper are deposited at GBIF, the Global Biodiversity Information Facility, https://doi.org/10.15468/41rneo.

## Results

### Morphological descriptions and taxonomic revision

Thirty-four species are recognised here, of which five (*C.alticola* Pirie & Chatrou; *C.brachypodum* Pirie & Chatrou; *C.confusum* Pirie; *C.dolichopodum* Pirie & Maas and *C.osicola* Pirie & Chatrou) are described as new. In most species of *Cremastosperma*, inflorescences are of single flowers, but in three (*C.cauliflorum* R.E.Fr.; Fig. 2b, *C.napoense* Pirie and *C.alticola* Pirie & Chatrou), a rhipidium is formed by the development of shoots from the axils of bracts on the short axillary shoot. Most flowering collections are in fact in bud, since the mature stage of the flower is relatively brief; mature flowers tend to drop their petals on pressing. Bud shape and development differ between species: bud development can be fully open, giving the appearance of a mature flower (e.g. *C.brevipes*; *C.leiophyllum*), loosely open (e.g. *C.pedunculatum*; *C.megalophyllum*) or closed; and if closed, buds can be depressed ovoid (e.g. *C.pendulum*; *C.yamayakatense*) to broadly ovoid-triangular (e.g. *C.monospermum*). The presence and type of indument on flowers, fruits and sometimes leaves, is diagnostic for several of the species, e.g. the long golden hairs on all parts of *C.bullatum*; the dense but shorter indument of *C.cauliflorum* and *C.microcarpum*; the unique pattern of indument on the petals of *C.awaense*; and the lack of indument on many species leading to a characteristic blackish colour of flowers and fruits of dried specimens.

Monocarp shape (i.e. that of the portions of the fruit containing the seeds, as distinct from the stipes on which they are borne) also differs consistently between species, from narrowly ellipsoid (e.g. *C.dolichocarpum*) through to globose (e.g. *C.magdalenae*) and from symmetrical to strongly asymmetrical (e.g. *C.chococola*; *C.antioquense*).

### IUCN prelimanary conservation status

From the extent of occurrence (EOO) and area of occupancy (AOO) of each species estimated using ConR, taking into account presence in protected areas, 10 are classified as Least Concern (LC), six as Near Threatened (NT), three as Vulnerable (VU); fourteen as Endangered (EN) and one (*C.brachypodum*) as Critically Endangered (CR). Detailed results are presented in Table 1, with discussion of each assessment under the corresponding species treatments.

**Table 1.** **Summary of IUCN preliminary conservation status per species including calculations using ConR (Dauby et al. 2017).** The total number of collections is shown, including the number georeferenced (thus included in the calculations using ConR). “Unique occurrences” represent those with differing coordinates, whereby for poorly collected species, numbers of collections that could not be precisely georeferenced but nevertheless represent additional occurrences are indicated; “locations” refers to the IUCN definition as “a geographically or ecologically distinct area in which a single threat can rapidly affect all individuals of the taxon present”; and “subpopulations” are “geographically or otherwise distinct groups in the population between which there is little demographic or genetic exchange (IUCN 2012). EOO = extent of occurrence, whereby the criteria contributing to endangered status are CR <100 km^2^, EN <5,000 km^2^ and VU <20,000 km^2^; AOO = area of occupancy, whereby the criteria are CR <10 km^2^, EN <500 km^2^ and VU <2,000 km^2^. We estimated EOO and AOO using ConR, also indicating for (potentially) threatened species where the values would be higher taking non-georeferenced specimens into account. Known occurrences (or not) in protected areas (national parks or reserves with protected status according to the IUCN; https://protectedplanet.net/ accessed 04.09.2018) are documented and an overall threat category with IUCN code is presented which takes into account each of these factors (as discussed individually in each species treatment).

Species	Number of collections (with coordinates)	Number of unique occurrences (inc. without coordinates)	Number of locations	Number of sub-populations	EOO (km^2^)	AOO (km^2^)	Protected areas (numbers of collections)	Category code
* Cremastosperma alticola *	2	2	2	2	NA	8	None	EN B2ab
* Cremastosperma antioquense *	3	3	3	3	124	12	None	EN B1ab+B2ab
* Cremastosperma awaense *	15 (14)	13	12	10	27640	52	None	NT
* Cremastosperma brachypodum *	1	1	1	1	NA	4	None	CR B2ab
* Cremastosperma brevipes *	55 (29)	18	14	11	47395	68	French Guiana: Les Nouragues [IUCN IV] (6)	LC
* Cremastosperma bullatum *	9	6	3	3	582	20	None	EN B1ab+B2ab
* Cremastosperma cauliflorum *	69 (48)	35	32	28	379557	140	Colombia: Parque Nacional Amacayacu [IUCN II] (1). Ecuador: Reserva Cuyabeno [IUCN: Fauna Production Reserve] (2).	LC
* Cremastosperma cenepense *	4	3	2	2	660	12	None	EN B1ab+B2ab
* Cremastosperma chococola *	3	3	3	3	1128	12	Colombia: Parque Nacional Utría [reportedly IUCN II, but not on https://protectedplanet.net/] (1)	EN B1ab+B2ab
* Cremastosperma confusum *	37 (33)	19	16	13	56103	64	Peru: Parque Nacional Manu [IUCN: World Heritage Site] (c. 6); Tambopata Reserve [IUCN VI] (c. 13)	LC
* Cremastosperma dolichocarpum *	14	7	5	4	1444	28	Colombia: Parque Nacional Las Orchídeas [IUCN II] (9); Parque Nacional Paramillo [IUCN II] (1)	EN B1ab+B2ab
* Cremastosperma dolichopodum *	3	2	2	2	NA	4	Peru: Reserva Comunal Yanesha [IUCN VI] (2)	EN B2ab
* Cremastosperma gracilipes *	88 (81)	51	45	40	248036	200	Colombia: Parque Nacional Amacayacu [IUCN II] (1). Ecuador: Parque Nacional Sumaco [IUCN: National Park] (1); Parque Nacional Yasuní [IUCN: National Park] (14).	LC
* Cremastosperma leiophyllum *	45 (31)	21	18	17	303604	84	Bolivia: Parque Nacional Isiboro-Secure [IUCN: National Park] (2); Parque Nacional Amboró [IUCN: National Park] (10)	LC
* Cremastosperma longicuspe *	20 (10)	8 (13)	8	8	30760	32	None	VU B2ab
* Cremastosperma longipes *	3	3	3	3	1634	12	None	EN B1ab+B2ab
* Cremastosperma macrocarpum *	6 (3)	3 (5)	3	3	420	12	None	EN B1ab+B2ab
* Cremastosperma magdalenae *	4	2	2	2	NA	4	None	EN B2ab
* Cremastosperma megalophyllum *	112 (96)	62	52	39	306660	248	Ecuador: Parque Nacional Sumaco (3); Parque Nacional Yasuní (9)	LC
* Cremastosperma microcarpum *	68 (45)	22	19	16	250581	88	Colombia: Parque Nacional Amacayacu (2). Peru: Reserva Nacional Pacaya-Samiria [IUCN VI] (6)	LC
* Cremastosperma monospermum *	131 (89)	67	63	57	2173227	264	Brazil: Alto Juruá Extractive Reserve [reportedly IUCN VI, but not on https://protectedplanet.net/] (2). Peru: Parque Nacional Bahuaja-Sonene [IUCN II] (2); Parque Nacional Manu (1); Tambopata Reserve (2).	LC
* Cremastosperma napoense *	30 (27)	20	15	11	10200	80	Ecuador: Parque Nacional Sumaco (4).	NT B1a+B2a
* Cremastosperma novogranatense *	7	7	6	6	1078	28	None	EN B1ab+B2ab
* Cremastosperma oblongum *	53 (44)	37	19	11	124687	108	Parque Nacional Yanachaga-Chemillén [IUCN II] (12); Reserva Comunal Yanesha [IUCN VI] (6)	LC
* Cremastosperma osicola *	9 (8)	8 (9)	4	3	273	28	Costa Rica: Corcovado National Park [IUCN II] (2)	NT B1a+B2a
* Cremastosperma pacificum *	7	6	6	4	3397	24	Colombia: San Cipriano Natural Reserve [IUCN VI] (2)	EN B1a+B2a
* Cremastosperma panamense *	40 (23)	13	11	9	8294	52	None	NT B1a+B2a
* Cremastosperma pedunculatum *	37 (27)	22	17	15	118272	84	Ecuador: Parque Nacional Podocarpus [IUCN: National Park] (2).	LC
* Cremastosperma pendulum *	22 (18)	16	11	7	152472	52	Peru: Reserva Nacional Pacaya-Samiria (1); Bosque de Protección San Matias [IUCN VI] (1); Parque Nacional Yanachaga-Chemillén (6); Reserva Comunal Yanesha (1)	NT B2a
* Cremastosperma peruvianum *	16 (14)	9	8	8	9706	36	None	VU B1ab+B2ab
* Cremastosperma stenophyllum *	3	3	3	3	3927	12	None	EN B1ab+B2ab
* Cremastosperma venezuelanum *	4	4	4	4	1102	16	Venezuela: Parque Nacional Henri Pittier [IUCN: National Park] (1)	NT B1a+B2a
* Cremastosperma westrae *	6	6	4	4	1649	24	None	EN B1ab+B2ab
* Cremastosperma yamayakatense *	25 (23)	11	8	8	6046	40	None	VU B1ab+B2ab

## Discussion

### Undescribed diversity, regional endemism and threat of extinction in Cremastosperma species

Since the ground-breaking taxonomic work of R.E. Fries (Fries 1930, 1931, 1934, 1937, 1939, 1948, 1950), there have been considerable increases in the numbers of plant collections available for study (ter Steege et al. 2016) and, in recent revisions, numerous previously unknown species of Neotropical Annonaceae have been described. The most extreme example may be that of *Pseudoxandra*, revised by Maas and Westra (2003), in which 80% of species treated were new or very recently described; *Klarobelia* now comprises 12 species, of which only 3 were described prior to the treatment of Chatrou (1998; i.e. 75% new); and *Unonopsis* now includes 50 species of which 23 (46%) were described by Maas et al. (2007). The previous state of knowledge of *Cremastosperma* species diversity was similar: since 1986, we have described 15 new species, which along with the additional five presented here, represents well over half of the 34 now recognised. Notably, the case of the largest Neotropical genus, *Guatteria*, is somewhat exceptional in this context. Recently revised by Maas et al. (2015), the number of recognised species in fact decreased, with many brought into synonymy. Both phenomena serve to emphasise the importance of performing and using, modern revisionary work: taking *Cremastosperma* as an example, of the 17 Amazon species documented by Cardoso et al. (2017), one (*C.killipii*) was placed in synonymy by Pirie, Kankainen and Maas (2005), but adding the new species described here, the total would still rise, to 19.

Of the five *Cremastosperma* species that we describe as new here, two are endemic to Peru (*C.brachypodum* and *C.dolichopodum*) and one is endemic to Costa Rica (*C.osicola*). Ulloa et al. (2004) reported 236 species of Annonaceae for Peru, of which León and Monsalve (2006) documented 44 endemics, including six species of *Cremastosperma*. With the newly described species and our updated assessment of species delimitations and distributions, the number of *Cremastosperma* species native to Peru increases to 17, of which seven are endemic (*C.brachypodum*, *C.cenepense*, *C.dolichopodum*, *C.longicuspe*, *C.pendulum*, *C.peruvianum* and *C.yamayakatense*). *Cremastospermaosicola*, the only *Cremastosperma* found in Costa Rica and the most northerly distributed species of the genus, belongs to the 4.8% of the 454 tree species native to the Osa Peninsula that are found nowhere else (Cornejo et al. 2012). The Osa Peninsula is an important refuge for species with distributions limited to Costa Rica and surrounding regions of Central America (Cornejo et al. 2012), harbouring other Costa Rican endemic Annonaceae, including the recently described *Desmopsisverrucipes* Chatrou, G.E. Schatz & N. Zamora, *Guatteriareinaldii* Erkens & Maas, and *G.rostrata* Erkens & Maas (Erkens et al. 2006).

Across the Neotropics, a similarly high degree of *Cremastosperma* species endemism is apparent: both species found in Panama (*C.panamense* and *C.westrae*) and both in Venezuela (*C.macrocarpum* and *C.venezuelanum*) are endemic, as is the one found in French Guiana (*C.brevipes*). West of the Andes, Colombia houses no fewer than six endemics (*C.antioquense*, *C.chococola*, *C.dolichocarpum*, *C.magdalenae*, *C.novogranatense* and *C.pacificum*) and Ecuador a further one (*C.stenophyllum*). Despite being more contiguous, the expanses of lowland rainforest found east of the Andes also harbour endemic species in Bolivia (*C.leiophyllum*) and Ecuador (*C.napoense*), in addition to those of Peru. Overall, with 22 of 34 species being national endemics and several of the others with similarly restricted distributions, widespread species such as *C.monospermum* are the exception rather than the rule in the genus. This proportion of species endemism may appear rather high, but is fairly typical for the Neotropics in general and for Neotropical Annonaceae genera in particular. Ulloa Ulloa et al. (2017) presented a recent tally of vascular plant species diversity in the Americas. They documented over 19,000 species for Peru, of which around 40% are endemic. This is more or less the same as the proportion for *Cremastosperma* species. For Ecuador and Colombia, they documented around 17,500 and 23,000 species, of which 31% and 29%, respectively were endemic. In *Cremastosperma*, the proportions of endemic species is somewhat lower in Ecuador (two of ten; 20%) and higher in Colombia (6 of 11; 55%), reflecting the greater proportion of more widespread Amazonian species found in Ecuador. Overall regional endemism of other Neotropical Annonaceae genera is similar to that of *Cremastosperma*, both in other less species rich, Andean-centred clades such as *Klarobelia* (7 of 12), *Malmea* (4 of 6), *Mosannona* (8 of 15) and *Pseudomalmea* (3 of 4) (Chatrou 1998; Chatrou and Pirie 2005; Erkens et al. 2006); *Cymbopetalum* Benth. (16 of 27 spp.) and *Porcelia* Ruiz & Pav. (6 of 8) (Murray 1993); and *Trigynaea* Schltdl. (5 spp. of 8; Johnson and Murray 1995); and in more species rich and widespread genera such as *Duguetia* A.St.-Hil. (42 of 93; Maas et al. 2003) and *Guatteria* (92 of 177; Maas et al. 2015).

Particularly for local endemic and rare species, ongoing rates of habitat loss present a severe threat of extinction (IUCN 2012). León and Monsalve (2006) proposed IUCN threat categories for three Peruvian endemic species: *C.oblongum* and *C.peruvianum* as vulnerable (VU, B1a: due to restricted and/or fragmented distributions) and *C.killipii* (= *C.longicuspe*) as endangered (EN, B1ab(iii): additionally due to decline in area, quality or extent of habitat). In this paper, we present assessments of levels of threat for all of the species of the genus, the majority of these for the first time. The method we employed is based on extent of occurrence (EOO) and area of occupancy (AOO), like that of Johnson and Murray (2018) in their treatment of Africa *Xylopia*. We also attempt to take into account factors such as trends in habitat loss, although this is based principally on known presence in (at least nominally) protected areas. The results are not entirely consistent with those of León and Monsalve (2006): our assessments for *C.longicuspe* (VU) and *C.oblongum* (LC; Table 1) are less severe. However, these species were in need of revision and collections have often been overlooked or misidentified. By contrast, *C.peruvianum* is morphologically distinctive, apparently genuinely range restricted and also classified as VU according to our results. This suggests that, particularly for the less well known, rarer and more range restricted species (i.e. many of those most likely to be of concern), our assessments should be a useful first estimate. For example, *C.peruvianum* is one of a number of northern-Peruvian (near) endemics, also including *C.bullatum*, *C.cenepense* and *C.yamayakatense*, for which the threat imposed by narrow distribution is compounded by the lack of protected areas in the region (particularly compared to in adjacent Ecuador; Bass et al. 2010). Overall, the threat assessment results present a picture similar to that reflected in the broad brushstrokes of regional endemism: over half of the species are threatened (Table 1) and most of those fall under the more severe threat categories as endangered (14) or critically so (1).

### Phylogeny and biogeography of Cremastosperma

**Figure 6.**
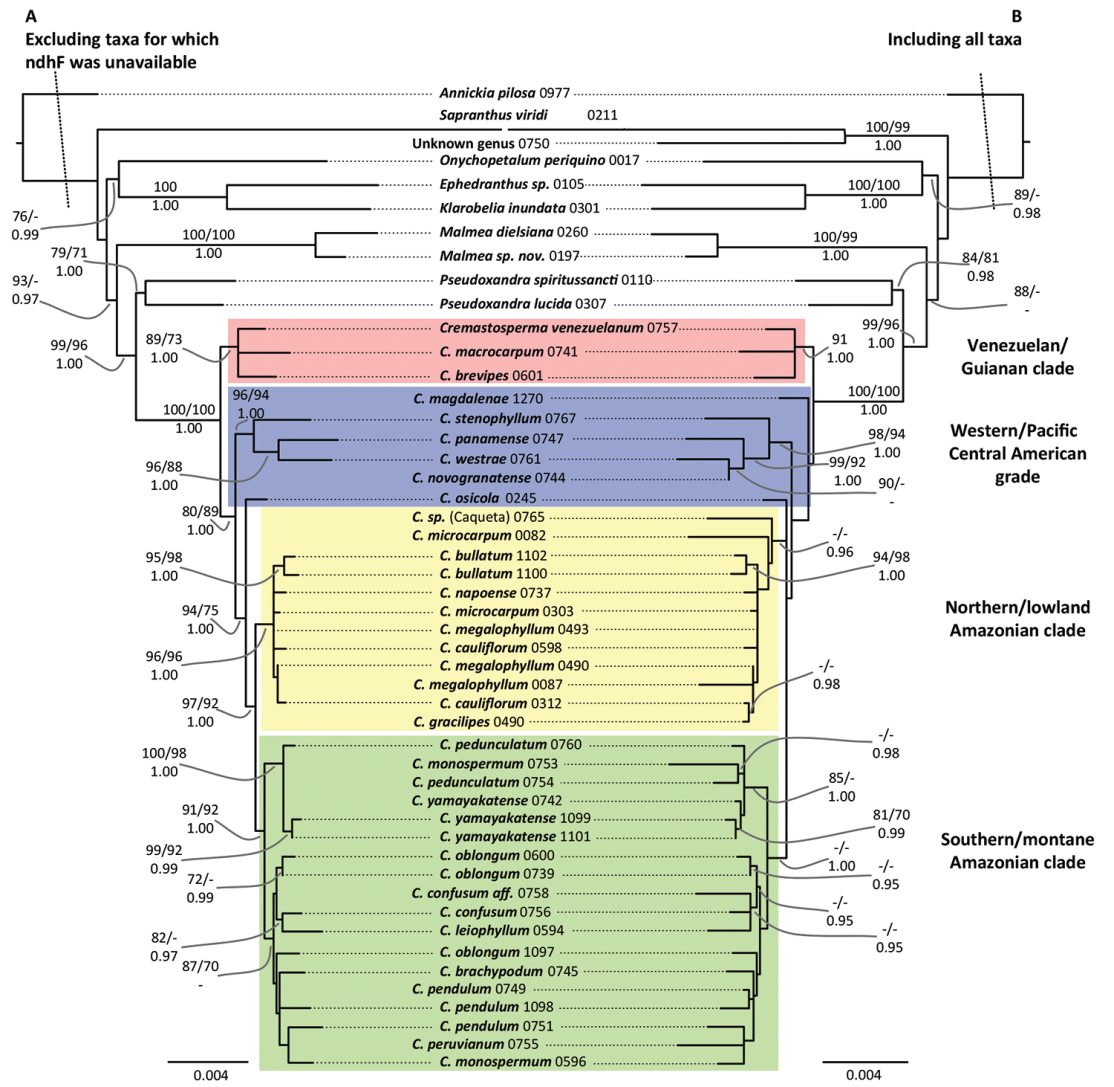
Phylogenetic hypotheses for *Cremastosperma* based on *rbcL*, *matK*, *trnT-F*, *psbA-trnH*, *ndhF* and pseud*trnL-F* (adapted from Pirie et al. (2018)). **A** Excluding taxa for which *ndhF* was unavailable **B** including all taxa. Topologies and branch lengths are of the best scoring ML trees with scale in substitutions per site. Branch lengths subtending the ingroup are not to scale. Clade support is indicated: ML and parsimony bootstrap percentages (above; left and right, respectively) and Bayesian posterior probabilities (below). Geographically restricted clades/lineages are highlighted with different colours and labelled on the right.

As part of a biogeographic analysis comparing the similar distribution patterns of *Cremastosperma* and *Mosannona* species, Pirie et al. (2018) presented a phylogenetic hypothesis for *Cremastosperma* based on multiple plastid markers. An updated version of that phylogenetic tree, modified to represent names of previously undescribed species, is presented in Fig. 6. The 34 accessions analysed by Pirie et al. (2018) represented 24 of 29 plus two informal species recognised prior to this work. With the taxonomic changes implemented here, this is now 25 of 34 species, of which those from the Pacific coast and Andean valleys of Colombia remain the least well represented.

Within well-supported geographically defined clades, resolution of the plastid phylogeny is limited, with polytomies and low clade support reflecting zero-length or short internal branches (Fig. 6). Plastid markers provided few informative characters at this low taxonomic level and Sanger sequencing approaches are probably not a realistic strategy for deriving fully resolved phylogenies of clades of closely related species in Malmeoideae, which is characterised by low rates of molecular evolution (Richardson et al. 2004; Mols et al. 2004). This also applies to larger genera in the faster evolving Annonoideae, the challenge of *Guatteria* (Erkens et al. 2007) being a case in point. One alternative currently being explored is to expand plastid datasets using high throughput sequencing (Hoekstra et al. 2017; Lopes et al. 2018). Whole plastid sequences might be expected to deliver a well-resolved plastid gene tree, but particularly at low taxonomic levels, this might differ markedly from the underlying species tree (Doyle 1992; Maddison 1997). The lack of clear monophyly of species, represented by multiple accessions in our analyses, may indicate the influence of population-level processes at recent timescales, which might be frequent in rainforest species due to large effective population sizes maintained over large areas (Pennington and Lavin 2016). Approaches that can target multiple independent nuclear coded markers (e.g. hybrid capture techniques) may be more fruitful and the development of protocols for such markers is of importance to further address problems in species-level analyses in Annonaceae (Couvreur et al. 2018).

Despite these limitations, the current phylogenetic hypothesis (Fig. 6) shows clades restricted to regions to the west and to the east of the Andes and even to regions within Amazonia (Fig. 4). The closely related species of the Northern/lowland Amazonia clade, which appear to have diversified within the region formerly inundated by Lake Pebas (Pirie et al. 2018), includes both hairy (e.g. *C.cauliflorum*; *C.bullatum*) and glabrous (*C.megalophyllum*; *C.brachypodum*) species and those with both simple (e.g. *C.microcarpum*; *C.gracilipes*) and branching (*C.cauliflorum*) inflorescences. Pollinator-mediated selection for divergent floral traits presents a tempting hypothetical driver for this species radiation, but data on pollinators within the clade is needed to test this. Species of the Southern/montane sister-clade appear more similar one to another, particularly in the common absence of indument, but do include both species with open (*C.leiophyllum*; *C.oblongum*) and closed (e.g. *C.monospermum*; *C.yamayakatense*) flower bud development. Given the more rugged topography typical of the habitats, particularly of the montane species, shifts in geographic range might have been more important in the speciation process in this clade. Again, more data is needed; in this case on the dispersal vectors of *Cremastosperma* species, in order to test whether and how dispersal between regions was limited within the timeframe of the species radiation.

The biogeographic scenario inferred by Pirie et al. (2018), on the basis of phylogeny, age estimates and niche modelling, reinforces the importance of the Andean orogeny in creating a barrier to dispersal during the diversification of the clade (Pirie et al. 2006). Further sampling of species of the Pacific coast and Andean valleys might be expected to reveal more ancient divergences, predating the Pliocene/Early Pleistocene rise of the Cordillera Oriental of the northern Andes (Gregory-Wodzicki 2000) or more recent close relationships with other western and Central American species. The strong geographical structure in the phylogeny, as well as the high degree of local species endemism, is consistent with phylogenetically conserved shifts in niche, combined with limited dispersal capability of these understorey trees.

## Systematic treatment

### Key to *Malmea*, *Pseudoxandra* and *Cremastosperma*

**Table:** 

1	Primary vein raised on upper side of leaf, flowers and fruit axillary or sometimes terminal on a short, axillary shoot	**2**
–	Primary vein impressed on upper side of leaf, flowers and fruit terminal, leaf-opposed or supra-axillary	*** Malmea ***
2	Primary vein on upper side of leaf not grooved, marginal vein of lamina distinct, monocarps (sub)globose to depressed globose, very rarely ellipsoid	*** Pseudoxandra ***
–	Primary vein on upper side of leaf distinctly grooved, marginal vein of lamina absent, monocarps (broadly) ellipsoid, rarely globose	*** Cremastosperma ***

### Cremastosperma

Taxon classificationPlantaeMagnolialesAnnonaceae

R.E.Fr.

Fig. 4 (Map 1)

Cremastosperma R.E.Fr., Acta Horti Bergiani 10: 46, f. 6a–c. 1930.

#### Type.

*Cremastospermapedunculatum* (Diels) R.E.Fr.

#### Description.

*Trees* or *shrubs* (0.5–)1.5–20 m tall; young twigs and petioles glabrous to densely covered with appressed or erect, simple, whitish to golden, up to 1 mm long hairs. *Leaves* distichous, simple, entire, petiolate, exstipulate; lamina elliptic to obovate or narrowly so, index 1.6–5, chartaceous to coriaceous, glabrous (rarely sparsely covered with appressed or erect, simple, up to 1 mm long hairs) above, glabrous to densely hairy (particularly at the base and on veins) below, base acute, obtuse or rounded, rarely subcordate to cordate, apex acuminate, sometimes caudate, rarely obtuse to acute, extreme tip rounded, venation brochidodromous, primary vein raised over entire leaf length above with an often conspicuous longitudinal groove particularly in the basal half, secondary veins 5–20(–30) on either side of the primary vein, often with 1–6 intersecondary veins, running parallel to primary vein for a short distance, thereafter angles with primary vein either increasing or decreasing towards the apex (or consistent), sometimes branching, often forming distinct loops, smallest distance between loops and margin 1–7 mm, tertiary veins percurrent (or reticulate). *Inflorescence* of single flowers or occasionally up to 8 in a rhipidium, pendant, clustered in groups of up to 7, terminal on short axillary shoots (i.e. peduncles) on leafy or leafless twigs, older branches or on the main trunk (then often on brachyblasts); 1-several lower bracts, deltate to depressed ovate, rarely narrowly elliptic, leafy, rounded to acute, soon falling off or persistent; single upper bract attached to pedicel, ovate to deltate, acute to obtuse; flower buds open or closed in development, when closed (ovoid to triangular) broadly to depressed ovoid; peduncles, pedicels, outer sides of bracts, sepals and petals glabrous to densely covered with appressed or erect, simple, up to 1 mm long hairs, bracts, sepals and petals ciliate. *Flowers* actinomorphic, bisexual, with one whorl of free or slightly connate, imbricate, sepals and two whorls of free, imbricate, petals, green, creamy or yellow *in vivo*, often black *in sicco*; sepals and petals thin at margins, occasionally with prominent venation; sepals three, much smaller than petals; petals six, the outer ones ovate, elliptic or broadly so, the inner ones elliptic to obovate or narrowly so, stamens numerous (ca. 100), spirally arranged, extrorse, inserted on and below a ventral ridge encircling a central depression in the receptacle in which the carpels are inserted, 1–2 mm long, connective appendage transversely rhombic-hexagonal; carpels 20–40, spirally arranged, free, ovary 1-locular, glabrous or hairy, with 1 basal, lateral or apical ovule (reported by Van Heusden 1992), stigma sessile. *Fruit* apocarpous, monocarps 5–40, stipitate, mostly asymmetrical, sometimes strongly so, sometimes with an (often excentric) apicule, green maturing mostly through red to brown or black *in vivo*, light brown to black *in sicco*. *Seeds* 1, lateral or apical (reported by Van Setten & Koek-Noorman 1992), ellipsoid to globose, yellow to reddish-brown, surface deeply to shallowly pitted, lacking an aril, with a raised or sunken raphe encircling seed longitudinally (diagonally), regularly (or more sinuously), ruminations spiniform.

#### Distribution.

34 species in the Neotropics: from southern Costa Rica in the north to Bolivia in the south. Most species are distributed in regions surrounding the Andean mountain range, two in coastal Venezuela (*Cremastospermamacrocarpum* Maas and *C.venezuelanum* Pirie), one in French Guiana (*C.brevipes* (DC.) R.E.Fr.) and one widespread across Brazil, south of the Amazon River (*C.monospermum* (Rusby) R.E.Fr.).

#### Habitat and ecology.

Lowland to premontane tropical wet forest, inundated areas and terra firme. At elevations of 0–2000 m. Flowers and fruit of species of *Cremastosperma* appear similar in overall morphology and ontology to those of other Annonaceae demonstrated or presumed to be beetle-pollinated and/or bird-dispersed. We have observed in various species that the inner petals form a loose pollination chamber when mature, similar to that observed in, for example, *Guatteria* (Gottsberger 1970); and flowers of Malmeoideae genera in general are visited at least predominantly by small beetles (Saunders 2012). Similarly, we have observed in various species that the fruits become fleshy at maturity and often present a colour contrast between, for example, black monocarps and bright red stipes, representing a classic bird/monkey dispersal syndrome (Gautier-Hion et al. 1985). However, we are unaware of detailed studies of either pollination biology or seed dispersal in species of *Cremastosperma*.

### Key to species of *Cremastosperma*

Identifying particular species of *Cremastosperma* with either only flowering or only fruiting material can be challenging. Some species are best distinguished on the basis of fruit characters, others with those of flowers (and only very few can be identified with any confidence on the basis of vegetative characters alone). Unfortunately, collections with both flowers and fruit are the exception rather than the rule. With this key, we attempted to use both flowering and fruiting characters in combination as far as seemed practical, but subsequently pursued separate leads for flowering and fruiting specimens, not all of which will necessarily deliver an unambiguous identification. Some species found in geographically widely disjunct areas can be morphologically rather similar (e.g. *C.oblongum* in central Peru and *C.pacificum* from the Pacific coast of Colombia) and others can be rather variable in what usually would be reliable diagnostic characters, such as pedicel, stipe or monocarp lengths (e.g. *C.megalophyllum*; *C.confusum*). Where identifications with limited (or potentially ambiguous) material might be uncertain, we list possible species compatible with the leads to that point and would refer the user to individual diagnoses and known distributions.

**Figure 7.**
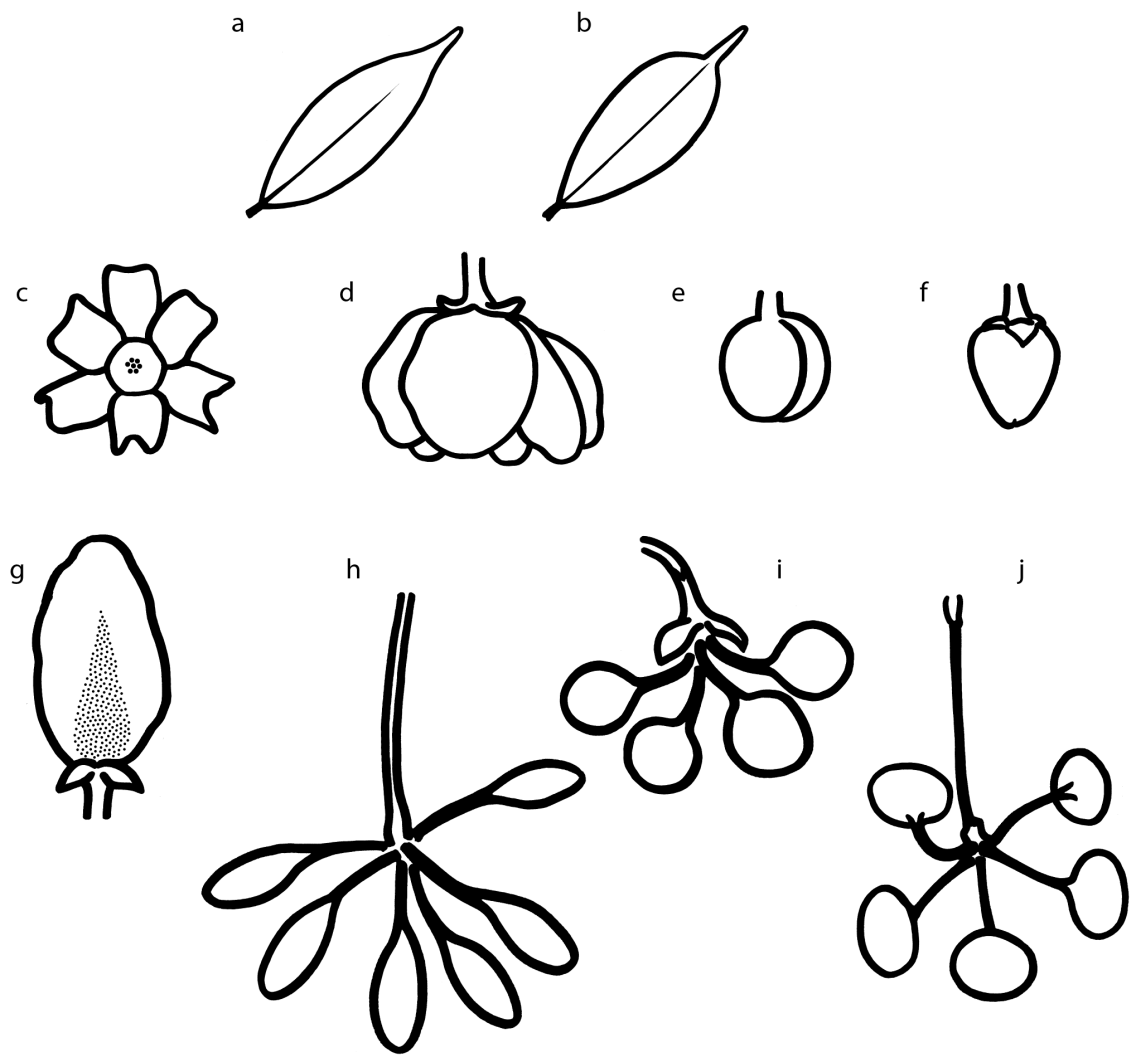
Illustrations of diagnostic characters used in the identification key to species of *Cremastosperma*, by Hendrik Rypkema, adapted from Pirie et al. (2005). Leaf shape: **a** acuminate **b** caudate. Flower buds: **c** with open development **d** loosely open **e** flower bud with closed development, depressed ovoid **f** flower bud with closed development, broadly ovoid-triangular **g** the unique pattern of indument on the petals of *C.awaense*. Monocarp shape **h** narrowly ellipsoid, symmetrical **i** globose, symmetrical **j** strongly asymmetrical.

A number of important diagnostic characters are illustrated in Fig. 7. In a few cases, we make a distinction between acuminate Fig. 7a (e.g. *C.microcarpum*) and caudate (in the sense of abruptly acuminate; Fig. 7b, e.g. *C.gracilipes*) leaf apex shapes. This represents an arbitrary, but, the first author believes, nevertheless generally useful, delimitation within a pattern of continuous variation. Nevertheless, there can be considerable variation within species and even within individual plants, which means that this distinction will not necessarily be helpful in every case.

**Table:** 

1	Pedicels >120 mm long in flower, >150 mm long in fruit	**2**
–	Pedicels <120 mm long in flower, ≤150 mm long in fruit	**3**
2	Leaf lamina 35–60 cm long. Flowers and fruits covered with appressed hairs – (Pacific coast of Colombia and Ecuador)	**16. *C.longipes***
–	Leaf lamina 22–35 cm long. Flowers and fruits glabrous – (Central Peru)	**12. *C.dolichopodum***
3	Leaf lamina bullate (in fresh and dry material), densely covered with hairs up to 1 mm long below and on margin. Pedicels up to 120 mm long in flower, 110–150 mm long in fruit – (The Peruvian state of Amazonas and adjacent Ecuador)	**6. *C.bullatum***
–	Leaf lamina not, or rarely slightly, bullate, sparsely to densely covered with hairs up to 0.5 mm long or glabrous below. Pedicels <95 mm long in flower, <110 mm long in fruit	**4**
4	Inflorescence branching, of multiple flowers/fruits	**5**
–	Inflorescence not branching, of single flowers/fruits	**7**
5	Outer side of sepals and of outer petals glabrous. Stipes shorter than monocarps – (N Peru, Ecuador)	**1. *C.alticola***
–	Outer side of sepals and outer petals densely covered with hairs. Stipes longer than monocarps	**6**
6	Outer side of inner petals densely covered with hairs. Monocarps globose to transversely broadly ellipsoid, rather densely covered with hairs – (Amazonian Brazil, Ecuador and Peru)	**7. *C.cauliflorum***
–	Outer side of inner petals largely glabrous but with a narrow, dense, sometimes branching band of appressed whitish-golden hairs extending from the base to halfway towards the apex. Monocarps ellipsoid, glabrous – (Amazonian Ecuador)	**22. *C.napoense***
7	Leaf apex obtuse to acute, base cordate (rarely rounded), secondary veins 15–30 on each side – (N Venezuela)	**32. *C.venezuelanum***
–	Leaf apex acuminate to caudate, base acute to obtuse (rarely rounded or cordate), secondary veins mostly <20 on each side	**8**
8	Leaf base cordate to subcordate	**9**
–	Leaf base acute to obtuse, rarely rounded	**10**
9	Leaf lamina <25 cm long. Pedicels ca. 8 mm long. Stipes 7–8 mm long – (The Peruvian state of Amazonas)	**8. *C.cenepense***
–	Leaf lamina >35 cm long. Pedicels ≥18 mm long. Stipes 20–40 mm long – (The Peruvian state of Amazonas)	**30. *C.peruvianum***
10	Leaf axillary buds conspicuous with dense indument. Monocarps narrowly ellipsoid (Fig. 7h), 27–28 mm long – (The Colombian state of Antioquia)	**11. *C.dolichocarpum***
–	Leaf axillary buds inconspicuous. Monocarps globose to ellipsoid, usually <27 mm long	**11**
11	Pedicels in flower and fruit hairy (even sparsely or minutely so)	**12**
–	Pedicels in flower and fruit entirely glabrous	**22**
12	Sepals and monocarps hairy (flowering and/or fruiting material is available; neither sepals nor monocarps are glabrous)	**13**
–	Sepals and/or monocarps glabrous (flowering and/or fruiting material is available; either sepals or monocarps or both are glabrous)	**19**
13	Sepals 7–10 mm long. Stipes 1.5–4 mm long. – (Pacific Coast of Colombia)	**23. *C.novogranatense***
–	Sepals <6 mm long. Stipes >4 mm long	**14**
14	Sepals <3 mm long. Monocarps ≥18 mm long	**15**
–	Sepals ≥3 mm long. Monocarps ≤15 mm long	**18**
15	Pedicels ≤17 mm long in flower or fruit. – (Panama)	**33. *C.westrae***
–	Pedicels ≥20 mm in flower or fruit	**16**
16	Pedicel ca. 45 mm in flower, ca. 62 mm in fruit. – (Pacific Coast of Ecuador)	**31. *C.stenophyllum***
–	Pedicel ≤28 mm in flower, ≤60 mm in fruit	**17**
17	Outer side of petals with dense hairs at base and in a line leading to the petal apex (Fig. 7g). Monocarps 22–28 mm long, longer than stipes, slightly asymmetrical – (Pacific coast of Colombia and Ecuador)	**3. *C.awaense***
–	Outer side of petals rather densely (evenly) covered with hairs. Monocarps 13–14 mm long, shorter than stipes, strongly asymmetrical (Fig. 7j) – (The Colombian state of Antioquia)	**2. *C.antioquense***
18	Leaf apex caudate (Fig. 7b), lamina glabrous on both sides. Flowers covered with hairs ca. 0.2 mm long. Monocarps 10–15 mm long – (Amazonian Colombia, Ecuador and Peru)	**13. *C.gracilipes***
–	Leaf apex acuminate (Fig. 7a), lamina glabrous above, glabrous to rather densely hairy at the base and on primary and secondary veins below. Flowers covered with hairs ca. 0.3 mm long. Monocarps 8–11 mm long – (Amazonian Brazil, Colombia, Ecuador and Peru)	**20. *C.microcarpum***
19	Flowering material available	**20**
–	Fruiting material available	**21**
20	Sepals glabrous [compare to *C.brevipes*, *C.oblongum*, *C.pacificum* and *C.pedunculatum* (note widely differing distributions); or follow lead 22]	
–	Sepals hairy	**15**
21	Monocarps strongly asymmetrical (Fig. 7j) – (The Colombian state of Antioquia)	**2. *C.antioquense***
–	Monocarps slightly asymmetrical [compare to *C.awaense*, *C.brevipes*, *C.oblongum*, *C.pacificum*, *C.pedunculatum* and *C.westrae*; note widely differing distributions]	
22	Pedicel length in flower ≤20 mm, in fruit ≤30 mm	**23**
–	Pedicel length in flower >20 mm, in fruit >30 mm	**68**
23	Flowering material available	**24**
–	Fruiting material available	**48**
24	Outer petals ≤15 mm long	**25**
–	Outer petals >15 mm long	**39**
25	Sepals ≤3 mm long	**26**
–	Sepals >3 mm long	**34**
26	Pedicels (in flower) ≤14 mm long	**27**
–	Pedicels (in flower) >14 mm long	**30**
27	Pedicels (in flower) ≤7 mm long	**28**
–	Pedicels (in flower) >7 mm long	**29**
28	Sepals 5–7 mm long (persistent in fruit) – (The Colombian state of Antioquia)	**18. *C.magdalenae***
–	Sepals ca. 3 mm long (not persistent) – (The Peruvian state of Amazonas)	**34. *C.yamayakatense***
29	Leaf apex caudate (Fig. 7b). Pedicels (in flower) 10–14 mm long, sepals 3–4 mm long – (N Peru)	**15. *C.longicuspe***
–	Leaf apex acuminate (Fig. 7a). Pedicels (in flower) 12–20 mm long, sepals 1.5–3 mm long – (Panama)	**27. *C.panamense***
30	Closed flower buds broadly ovoid-triangular, remaining closed throughout development, with the petals not opening fully even at maturity (Fig. 7f) – (Amazonian Bolivia, Brazil and Peru)	**21. *C.monospermum***
–	Closed flower buds depressed ovoid, opening during development (Figs 7c–e)	**31**
31	Leaves, when dried, with a characteristic reddish tinge (particularly on the underside). Sepals 3–4(–5) mm long – (French Guiana)	**5. *C.brevipes***
–	Leaves drying grey- or brownish-green, only veins below with reddish tinge (or not). Sepals 1.5–3 mm long	**32**
32	Leaves sparsely covered with appressed whitish hairs particularly on veins below – (S Peru)	**10. *C.confusum***
–	Leaves glabrous on both sides	**33**
33	Veins on underside of leaves without reddish tinge. Pedicels 12–20 mm long (in flower). Sepals 1.5–3 mm long – (Panama)	**27. *C.panamense***
–	Veins on underside of leaves with reddish tinge. Pedicels 18–34 mm long (in flower). Sepals 2–3 mm long – (Bolivia)	**14. *C.leiophyllum***
34	Pedicels (in flower) ≤7 mm long – (The Colombian state of Antioquia)	**18. *C.magdalenae***
–	edicels (in flower) >7 mm long	**35**
35	Pedicels (in flower) 40–50(–70) mm long – (Amazonian Bolivia, Brazil and Peru)	**21. *C.monospermum***
–	Pedicels (in flower) ≤20 mm long	**36**
36	Leaves, when dried, with a characteristic reddish tinge (particularly on the underside). Flower buds opening widely in development – (French Guiana)	**5. *C.brevipes***
–	Leaves, when dried, greyish or brownish (olive)-green. Flower buds opening loosely or remaining closed in development	**37**
37	Leaves 10–27 cm long, apex caudate (Fig. 7b). Sepals 3–4 mm long, outer petals 10–12 mm long – (N Peru)	**15. *C.longicuspe***
–	Leaves 13–64 cm long, apex acuminate (Fig. 7a). Sepals 4–6 mm long, outer petals 11–29 mm long	**38**
38	Flower buds depressed ovoid, opening loosely in development, sepals and petals both drying blackish-brown – (Amazonian Colombia, Ecuador and Peru)	**19. *C.megalophyllum***
–	Flower buds broadly ovoid-triangular, remaining loosely closed in development, sepals drying lighter brown than petals – (The Peruvian states of Amazonas and Loreto)	**30. *C.peruvianum***
39	Sepals ≤3 mm long	**40**
–	Sepals >3 mm long	**46**
40	Outer petals >30 mm long – (Costa Rica)	**25. *C.osicola***
–	Outer petals <30 (mostly <22) mm long	**41**
41	Sepals ≤2 mm long	**42**
–	Sepals >2 mm long	**43**
42	Leaves 13–45 cm long. Sepals ca. 2 mm long, recurved, not persistent (The Brazilian state of Acre and C and S Peru)	**24. *C.oblongum***
–	Leaves 8–22 cm long. Sepals 1.5–3 mm long, not recurved, often persistent (Panama)	**27. *C.panamense***
43	Lower side of leaves sparsely covered with appressed whitish hairs, particularly on veins. Pedicels ≥20 mm long – (S Peru and Bolivia)	**10. *C.confusum***
–	Lower side of leaves mostly glabrous. Pedicels ≤20 mm long	**44**
44	Leaves, when dried, with a characteristic reddish tinge (particularly on the underside) – (French Guiana). Sepals 3–4 mm long	**5. *C.brevipes***
–	Leaves, when dried, greyish- or brownish (olive)-green. Sepals 1.5–3 mm long	**45**
45	Leaves 19–41 cm long. Sepals ca. 3 mm long, not persistent – (Pacific coast of Colombia)	**26. *C.pacificum***
–	Leaves 8–22 cm long. Sepals 1.5–3 mm long, often persistent – (Panama)	**27. *C.panamense***
46	Outer petals >30 mm long – (Costa Rica)	**25. *C.osicola***
–	Outer petals <30 mm long	**47**
47	Leaves, when dried, with a characteristic reddish tinge (particularly on the underside). Flower buds opening widely in development – (French Guiana)	**5. *C.brevipes***
–	Leaves, when dried, greyish- or brownish (olive)-green. Flower buds opening loosely or remaining closed in development	**38**
48	Monocarps <15 mm long	**49**
–	Monocarps ≥15 mm long	**63**
49	Stipes ≤15 mm long	**50**
–	Stipes >15 mm long	**59**
50	Monocarps globose	**51**
–	Monocarps ellipsoid	**52**
51	Leaves 20–28 cm long. Sepals usually 5–7 mm long (persistent in fruit) – (The Colombian state of Antioquia)	**18. *C.magdalenae***
–	Leaves 8–22 cm long. Sepals 1.5–3 mm long (often persistent in fruit) – (Panama)	**27. *C.panamense***
52	Monocarps longer than stipes	**53**
–	Monocarps shorter than stipes	**55**
53	Tree, 4–20 m tall. Leaves, when dried, with a characteristic reddish tinge (particularly on the underside) – (French Guiana)	**5. *C.brevipes***
–	Tree or shrub 1–12 m tall. Leaves, when dried, greyish- or brownish (olive)-green	**54**
54	Leaf apex caudate to acuminate. Pedicels (12-)22–73 mm long (in fruit), stipes 9–11 mm long – (Amazonian Bolivia, Brazil and Peru)	**21. *C.monospermum***
–	Leaf apex acuminate. Pedicels 8–15(–20) mm long (in fruit), stipes 12–14 mm long – (The Peruvian state of Amazonas)	**34. *C.yamayakatense***
55	Monocarps <12 mm long [compare to *C.confusum* and *C.monospermum*]	
–	Monocarps ≥12 mm long	**56**
56	Pedicels (in fruit) ≤20 mm long	**57**
–	Pedicels (in fruit) >20 mm long	**58**
57	Leaves 10–27 cm long, apex caudate. (Fig. 7b) – (N Peru)	**15. *C.longicuspe***
–	Leaves 13–57 cm long, apex acuminate (Fig. 7a) – (Amazonian Colombia, Ecuador and Peru)	**19. *C.megalophyllum***
58	Leaves 10–28(–34) cm long. Stipes 8–13 mm long, monocarps 8–12 mm long- (S Peru)	**10. *C.confusum***
–	Leaves 13–57 cm long. Stipes 10–30 mm long, monocarps 12–20 mm long – (Amazonian Colombia, Ecuador and Peru)	**19. *C.megalophyllum***
59	Monocarps globose – (Panama)	**27. *C.panamense***
–	Monocarps ellipsoid	**60**
60	Monocarps drying blackish, asymmetrical, the stipes thickening somewhat where they meet the monocarps – (Bolivia)	**14. *C.leiophyllum***
–	Monocarps drying light to dark brown or blackish, the stipes not markedly thickening where they meet the monocarps	**61**
61	Leaves 10–27 cm long, apex caudate (Fig. 7b). Pedicels (in fruit) 11–20 mm long – (N Peru)	**15. *C.longicuspe***
–	Leaves 13–57 cm long, apex acuminate (Fig. 7a). Pedicels (in fruit) 15–40 mm long	**62**
62	Number of monocarps 6–32 – (Amazonian Colombia, Ecuador and Peru)	**19. *C.megalophyllum***
–	Number of monocarps 20–43 – (Costa Rica)	**25. *C.osicola***
63	Monocarps longer than stipes	**64**
–	Monocarps shorter than stipes	**66**
64	Leaves, when dried, with a characteristic reddish tinge (particularly on the underside) – (French Guiana)	**5. *C.brevipes***
–	Leaves, when dried, greyish- or brownish (olive)-green	**65**
65	Pedicels 12–35(–55) by 1.5–3 mm (in fruit) – (The Brazilian state of Acre and C and S Peru)	**24. *C.oblongum***
–	Pedicels 22–35 by ca. 1.5 mm (in fruit) – (Pacific coast of Colombia)	**26. *C.pacificum***
66	Monocarps drying blackish, asymmetrical, the stipes thickening somewhat where they meet the monocarps – (Bolivia)	**14. *C.leiophyllum***
–	Monocarps drying light to dark brown or blackish, the stipes not markedly thickening where they meet the monocarps [compare to *C.megalophyllum*, *C.osicola*, *C.pacificum* and *C.peruvianum* (note widely differing distributions)]	
67	Flowering material available	**68**
–	Fruiting material available	**81**
68	Pedicels (in flower) <35 mm long	**69**
–	Pedicels (in flower) ≥35 mm long	**77**
69	Outer petals ≤15 mm long	**70**
–	Outer petals >15 mm long	**74**
70	Sepals ≥4 mm long – (Amazonian Colombia, Ecuador and Peru)	**19. *C.megalophyllum***
–	Sepals <4 mm long	**71**
71	Outer petals ≤6 mm long – (Central Peru)	**29. *C.pendulum***
–	Outer petals ≥11 mm long	**72**
72	Leaves glabrous above, sparsely covered with appressed whitish hairs particularly on veins below – (S Peru and Bolivia)	**10. *C.confusum***
–	Leaves glabrous on both sides	**73**
73	Leaves olive green, more greyish above – (Central Peru)	**4. *C.brachypodum***
–	Leaves brown/green with a reddish tinge on both sides (particularly on the veins on the underside) – (Bolivia)	**14. *C.leiophyllum***
74	Sepals ≥4 mm long – (Amazonian Colombia, Ecuador and Peru)	**19. *C.megalophyllum***
–	Sepals <4 mm long	**75**
75	Pedicels (in flower) ≤20 mm long	**65**
–	Pedicels (in flower) >20 mm long	**76**
76	Pedicels (in flower) 20–45(–70) mm long – (S Peru and Bolivia)	**10. *C.confusum***
–	Pedicels (in flower) 12–20 mm long – (Pacific coast of Colombia)	**26. *C.pacificum***
77	Outer petals ≤6 mm long – (Central Peru)	**29. *C.pendulum***
–	Outer petals ≥9 mm long	**78**
78	Leaf apex caudate to acuminate. Closed flower buds broadly ovoid-triangular, remaining closed in development – (Amazonian Bolivia, Brazil and Peru)	**21. *C.monospermum***
–	Leaf apex acuminate. Closed flower buds very broadly ovoid to globose, opening in development	**79**
79	Pedicels (in flower) 35–45 mm long, sepals 1.5–2 mm long – (N Venezuela)	**17. *C.macrocarpum***
–	Pedicels (in flower) 20–95 mm long, sepals 2.5–4 mm long	**80**
80	Petal venation not prominent – (S Peru and Bolivia)	**10. *C.confusum***
–	Petals venation prominent – (N Peru, Ecuador)	**28. *C.pedunculatum***
81	Stipes ≤2 mm long – (Central Peru)	**4. *C.brachypodum***
–	Stipes >2 mm long (usually >6 mm long)	**82**
82	Monocarps ≤12 mm long [compare to *C.confusum*, *C.megalophyllum*, *C.monospermum*, *C.pedunculatum* and *C.pendulum* (flowering material may be needed for a precise determination)]	
–	Monocarps >12 mm long	**83**
83	Monocarps longer than stipes	**84**
–	Monocarps shorter than stipes	**85**
84	Monocarps ≤17 mm long [compare to *C.oblongum*, *C.pacificum*, *C.pedunculatum* and *C.pendulum* (flowering material may be needed for a precise determination)]	
–	Monocarps >17 mm long [compare to *C.macrocarpum*, *C.oblongum* and *C.pacificum* (note widely differing distributions)]	
85	Leaf lamina 11–20 cm long. Inflorescence on main trunk. Monocarps ellipsoid, strongly asymmetrical (Fig. 7j) – (Pacific coast of Colombia)	**9. *C.chococola***
–	Leaf lamina 7–57 cm long. Inflorescence on leafy twigs, thicker branches or on the main trunk. Monocarps ellipsoid, slightly asymmetrical	**86**
86	Monocarps drying blackish, the stipes thickening somewhat where they meet the monocarps – (Bolivia)	**14. *C.leiophyllum***
–	Monocarps drying light to dark brown or blackish, the stipes not markedly thickening where they meet the monocarps [compare to *C.megalophyllum, C.pacificum*, *C.pedunculatum* and *C.pendulum* (flowering material may be needed for a precise determination)]	

### Synoptic Key

Species codes cited refer to those used in the identification list. Those cited under more than one lead are indicated in bold. Where a character state is unknown for a given species, the corresponding code is omitted.

Leaf axillary buds indument

a) Conspicuously hairy

dol-nov

b) Inconspicuous and glabrous

alt-ant-awa-bra-bre-bul-cau-cen-cho-con-dop-gra-lei-lon-lop-mac-mag-meg-mic-mon-nap-obl-osi-pac-pan-ped-pen-per-ste-ven-wes-yam

Leaf length

a) <25

**alt-ant-awa**-bra-**bre-bul-cau-cen-cho-con-dol-gra-lei-lon-mac-mag-meg-mic-mon-nap-obl-osi-pac-pan-ped-pen-yam**

b) 25–35

**alt-ant-awa-bre-bul-cau-con**-dop-**gra-lei-lon-mac-mag-meg-mic-mon-nap-nov-obl-osi-pac-ped-pen-ste-ven-wes**

c) >35

**bre**-cau-lop-meg-nap-nov-obl-osi-pac-per-ven-wes

Leaf base

a) Cordate, subcordate or rounded

bul-cen-**nov**-per-ven

b) Obtuse or acute

alt-ant-awa-bra-bre-cau-cho-con-dol-dop-gra-lei-lon-lop-mac-mag-meg-mic-mon-nap-**nov**-obl-osi -pac-pan-ped-pen-per-ste-wes-yam

Leaf apex

a) Acute or obtuse

ven

b) Acuminate

alt-ant-awa-bra-bre-bul-**cau**-cen-cho-con-dol-dop-**gra**-lei-**lon**-lop-mac-mag-meg-mic-**mon**-nap-**nov**-obl-osi-pac-pan-ped-pen-per-ste-wes-yam

c) Caudate

**cau**-**gra**-**lon**-**mon**-**nov**

Leaf appearance

a) Bullate

bul-(**meg**)

b) Not bullate

alt-ant-awa-bra-bre-cau-cen-cho-con-dol-dop-gra-lei-lon-lop-mac-mag-(**meg**)-mic-mon-nap-nov-obl-osi-pac-pan-ped-pen-per-ste-ven-wes-yam

Secondary veins

a) <18

alt-ant-awa-bra-bre-**bul**-cau-cen-cho-con-dol-dop-gra-lei-lon-lop-mac-mag-**meg**-mic-mon-nap-nov-**obl**-osi-pac-pan-ped-pen-ste-**ven**-wes-yam

b) ≥18

**bul**-**meg**-**obl**-per-**ven**

Inflorescence position

a) On main trunk

**ant**-**bul**-**cau**-cho-(**dop**)-**meg**-(**obl**)-**osi**-**ped**-ven

b) On thicker branches and leafless twigs

**ant**-**awa**-bre-**bul**-**cau**-**con**-**dol**-**dop**-lei-lop-**meg**-**mic**-**mon**-nap-**nov**-**obl**-**osi**-pac-**pan**-**ped**-**per**-ste-ven-**wes**-**yam**

c) On leafy twigs

**an**t-**awa**-bra-**bul**-cen-**con**-**dol**-gra-lon-mac-mag-**meg**-**mic**-**mon**-**nov**-**obl**-**pan**-**ped**-pen-**per**-**wes**-**yam**

Inflorescence

a) Branching

alt-cau-nap

b) Simple

**alt**-ant-awa-bra-bre-bul-**cau**-cen-cho-con-do-dop-gra-lei-lon-lop-mac-mag-meg-mic-mon-**nap**-nov-obl-osi-pac-pan-ped-pen-per-ste-ven-wes-yam

Pedicel length (in flower)

a) <10 mm

mag-(**mic**)-**obl**-yam

b) 10–20 mm

**ant**-bre-**cau**-**gra**-**lei**-lon-meg-**mic**-nov-**obl**-pac-pan-per-ven

c) 21–80 mm

**ant**-awa-bra-**cau**-con-dol-**gra**-**lei**-mac-(**meg**)-**mic**-mon-nap-ped-pen-ste

d) >80 mm

bul-dop-lop-(**ped**)

Pedicel length (in fruit)

a) <20 mm

**bre-cau**-cen-**gra**-**lei**-**lon**-**mag**-**meg**-mic-**nov**-**obl**-**osi**-pan-**per**-**ven**-wes-yam

b) 20–39 mm

alt-ant-**awa**-bre-**cau**-**cho**-**gra**-**lei**-**lon**-**mag**-**meg**-mic-**mon**-nap-**nov**-**obl**-**osi**-pac-**pan**-**per**-**ven**

c) 40–100 mm

ant-**awa**-bra-**cau**-**cho**-dol-mac-(**meg**)-**mon**-(**obl**)-**ped**-pen

d) >100 mm

bul-dop-lop-(**ped**)

Pedicel indument

a) Hairy

ant-awa-**bre**-bul-cau-cen-dol-gra-**lon**-lop-mic-nap-nov-obl-**pac**-**ped**-ste-wes

b) Glabrous

alt-bra-**bre**-cho-con-dop-lei-**lon**-mac-mag-meg-mon-osi-**pac**-pan-**ped**-pen-per-ven-yam

Flower buds

a) Opening widely

bre-cau-lei-nap-obl-pan

b) Opening loosely

ant-awa-dol-gra-lon-lop-mac-mag-meg-mic-nov-pac-ped-ste-ven-wes

c) Remaining closed

bul-mon-pen-per-yam

Closed flower bud shape

a) Depressed ovoid

alt-ant-awa-bra-bre-cau-con-dol-gra-lei-lon-lop-mac-mag-meg-mic-nap-nov-obl-pac-pan-ped-pen-ven-wes-yam

b) Broadly ovoid-triangular

bul-mon-per

Sepals length

a) ≤2.5 mm

ant-awa-bra-dop-lei-mac-(**mag**)-mon-nap-obl-pan-ped-pen-ste-ven-wes

b) 2.6–5 mm

bre-cau-con-dol-gra-lei-lon-lop-mag-meg-mic-mon-nap-pac-pan-ped-per-yam

c) 5.1–7.5

bul-mag-meg-**nov**

d) >7.5

nov

Sepals indument

a) Hairy

ant-awa-bul-cau-dol-gra-lop-mic-nap-nov-(**obl**)-ste-wes

b) Glabrous

alt-bra-bre-con-dop-lei-lon-mac-mag-meg-mon-**obl**-pac-pan-ped-pen-per-ven-yam

Sepals persistence

a) Always

mag

b) Occasionally and/or partially

bra-dol-dop-meg-nov-pan-wes

c) Never

ant-awa-bre-bul-cau-con-gra-lei-lon-lop-mac-mic-mon-nap-obl-osi-pac-ped-pen-per-ven-yam

Monocarp shape

a) Globose to transversely broadly ellipsoid

cau-mag-pan

b) Ellipsoid

alt-ant-awa-bra-bre-bul-cen-cho-con-dol-dop-gra-lei-lon-lop-mac-meg-mic-mon-nap-nov-obl-osi-pac-ped-pen-per-ven-wes-yam

c) Narrowly ellipsoid

dol

Monocarp symmetry

a) Strongly asymmetrical

ant-cho

b) Slightly asymmetrical

alt-awa-bra-bre-bul-cau-cen-con-dol-dop-gra-lei-lon-lop-mac-mag-meg-mic-mon-nap-nov-obl-osi-pac-pan-ped-pen-per-ven-wes-yam

Monocarp length

a) <12 mm

bra-**bre-cau**-**con**-**gra**-mic-mon-**pan**-pen

b) 12–18 mm

ant-**bre**-bul-**cau**-cen-cho-**con**-**gra**-lei-lon-mag-nap-nov-obl-osi-pac-**pan**-ped-pen-per-wes-yam

c) 18.1–24 mm

**alt-awa**-dop-lop-mac-meg-nap-nov-obl-per-ven-**wes**

d) >24 mm

**alt-awa**-dol

Monocarp indument

a) Hairy

bul-cau-cen-gra-mic-nov

b) (Appearing) glabrous

alt-ant-awa-bra-bre-cho-con-dol-dop-lei-lon-lop-mac-mag-meg-mon-nap-obl-osi-pac-pan-ped-pen-per-ven-wes-yam

Stipe length

a) <4 mm

bra-**nov**

b) 4–10 mm

**bre-cau**-cen-**con**-**gra**-**lon**-**mac**-mag-**meg**-**mic**-**mon**-**nov**-**obl**-**pac**-**pan**-**pen**-**wes**

c) 10.1–20 mm

alt-ant-awa-**bre**-bul-**cau**-cho-**con**-dol-**dop**-**gra**-**lei**-**lon**-**mac**-**meg**-**mic**-**mon**-nap-**obl**-osi-**pac**-**pan**-**ped**-**pen**-**per**-ven-**wes**-yam

d) 20.1–30 mm

**awa**-**cau**-**dop**-**lei**-lop-**meg**-nap-**pan**-**ped**-**per**

e) >30 mm

(**cau**)-**per**

Distribution

a) French Guiana

bre

b) N Venezuela

mac-ven

c) Central America (Costa Rica, Panama)

osi-pan-wes

d) Pacific coast of Colombia and Ecuador

awa-cho-lop-nov-pac-ste

e) The Colombian state of Antioquia

ant-dol-mag

f) Amazonian Brazil,

**cau-mic**-**mon**-**obl**

g) Amazonian Colombia

**gra**-**meg**-**mic**

h) Amazonian Ecuador

**alt-cau-gra**-**meg**-nap-**ped**

i) Peru

**alt**-bra-**bul-cau-cen-con**-**dop**-**gra**-**meg**-**mic**-**mon-obl**-**ped-pen-per-yam**

j) N Peru

alt-bul-cau-cen-gra-meg-mic-mon-ped-per-yam

k) The Peruvian state of Amazonas

bul-cen-per-yam

l) S and Central Peru

con-dop-mon-obl-pen-per

m) Bolivia

lei-**mon**

### 1. Cremastosperma alticola

Taxon classificationPlantaeMagnolialesAnnonaceae

Pirie & Chatrou sp. nov.

urn:lsid:ipni.org:names:60477506-2

Fig. 8 ; Map 2

#### Diagnosis.

Most similar to *C.pedunculatum*, from which it differs by the branching inflorescence, shorter pedicels and shorter stipes and the absence of indument on the pedicels. Differs from the only two other *Cremastosperma* species with branching inflorescences by the absence of indument on flowers and fruits and shape of the monocarps (compared to generally hairy *C.cauliflorum* with globose to transversely broadly ellipsoid monocarps) and stipes shorter than monocarps (much longer than monocarps in *C.napoense*).

#### Type.

ECUADOR. Zamora-Chinchipe: Palanda, Región de la Cordillera del Cóndor, sector sur, Parroquia San Francisco de Vergel, Cuenca alta del Río Vergel, Pica Sangola, 16 Mar 2005, *Quizhpe, W. et al. 1088* (holotype: MO! [MO-2985962]; isotypes: MO! [MO-2985963], LOJA, U! [U 0249915]).

#### Description.

*Tree* 6–20 m tall; young twigs and petioles glabrous to sparsely covered with appressed golden brown hairs 0.1–0.2 mm long. *Leaves*: petioles 5–9 by 1.5–2 mm; lamina elliptic to obovate, 17–27 by 6–7.5 cm (leaf index 2.7–2.9), chartaceous, patchy blackish-brown with darker primary vein, glabrous, base acute to obtuse, apex acuminate (acumen ca. 6 mm long), primary vein raised over entire of leaf length, ca. 2 mm wide at widest point, secondary veins 9–10, intersecondary veins 3–4, distance between from 5–10 mm at the base to 15–25 mm closer to the apex, angles with primary vein from 30–40° at the base to 40–50° closer to the apex, not branching, forming indistinct loops, smallest distance between loops and margin 2–3 mm, tertiary veins somewhat reticulate. *Inflorescence* of 1–2 flowers, branching, clustered in groups of up to 2, on leafy and leafless twigs; transition between short axillary shoot and pedicel unclear, combined structure 26–33 by ca. 1.5 mm at the base, ca. 4 mm at the apex (in fruit; only young buds seen), short axillary shoot and pedicels glabrous; 1 lower bract, broadly elliptic, ca. 2 by 1.5 mm, rounded, only present in early bud, glabrous, ciliate; upper bract directly subtending flower in bud, midway along pedicel in fruit, shallowly triamgular, ca. 2 by 3 mm, rounded, glabrous, ciliate; closed flower buds depressed ovoid, blackish-brown *in sicco*, sepals and petals glabrous and ciliate; mature flowers not seen, flowers reported as greenish-yellow *in vivo*. *Monocarps* 6–17, ellipsoid, symmetrical, 19–30 by 15–25 mm, no obvious apicule, black *in vivo*, blackish-brown *in sicco*; stipes 10–14 by 1.5 mm at the base increasing to ca. 4 mm at the apex; fruiting receptacle ellipsoid to transverse elipsoid, 6–12 mm diam.; monocarps, stipes and receptacle glabrous. *Seeds* ellipsoid, reddish-brown, pitted to grooved, 15–16 by 10–11 mm, raphe sunken, encircling seed longitudinally, ruminations spiniform.

#### Distribution.

Ecuador (Zamora-Chinchipe), Peru (Cajamarca)

#### Habitat and ecology.

In dense and secondary forest. At elevations of 1700 and 2200 m. Flowering: March; fruiting: March and June.

#### Notes.

Branching inflorescences are unusual in the genus, previously described in only two species: *C.cauliflorum* and *C.napoense*.

#### Etymology.

The epithet “alticola” refers to the high elevations at which the species has been found, which is unusual for the genus (and also the family in general).

#### Preliminary conservation status.

Neither of the only two known collections of *C.alticola* was collected in protected areas and one was reported to be found in close proximity to human habitation. Given this low area of occupancy and the likely ongoing decline in area, extent and/or quality of the habitat, we classify the species as Endangered [EN] (Table 1).

#### Other specimen examined.

**Peru.** Cajamarca: San Ignacio, Distr. San José de Lourdes, *Perea & Flores 2540* (AMAZ, HUT, L, MO, MOL, USM, WAG),

**Map 2.**
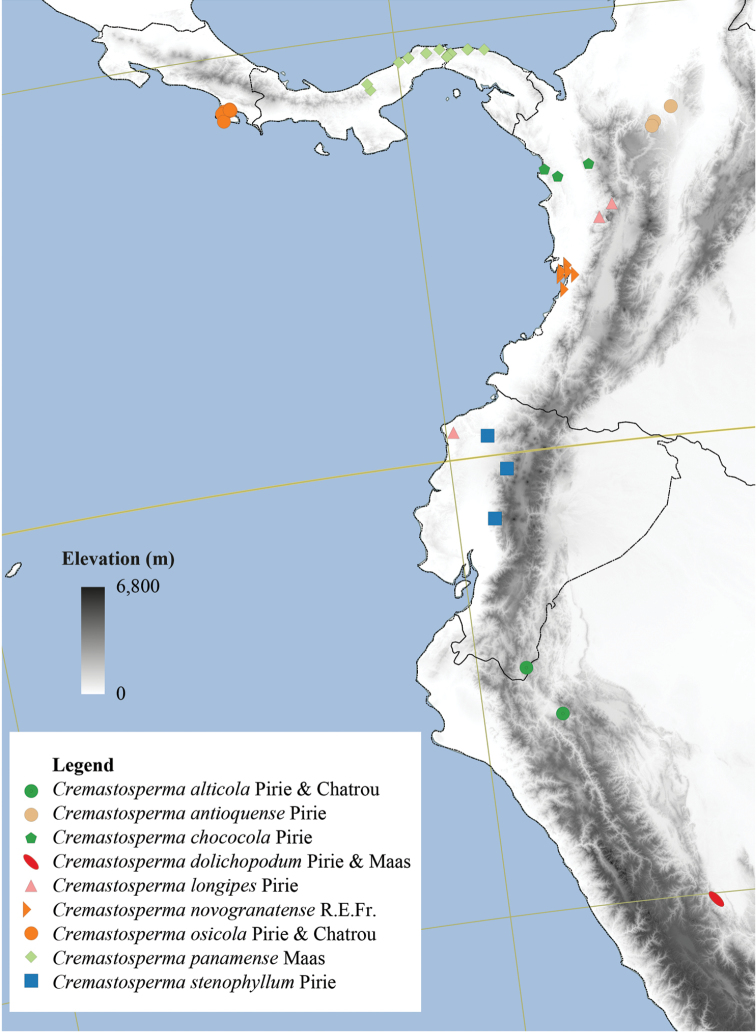
Distribution of *Cremastospermaalticola* Pirie & Chatrou, *C.antioquense* Pirie, *C.chococola* Pirie, *C.dolichopodum* Pirie & Maas, *C.longipes* Pirie, *C.novogranatense* R.E.Fr., *C.osicola* Pirie & Chatrou, *C.panamense* Maas and *C.stenophyllum* Pirie

**Figure 8.**
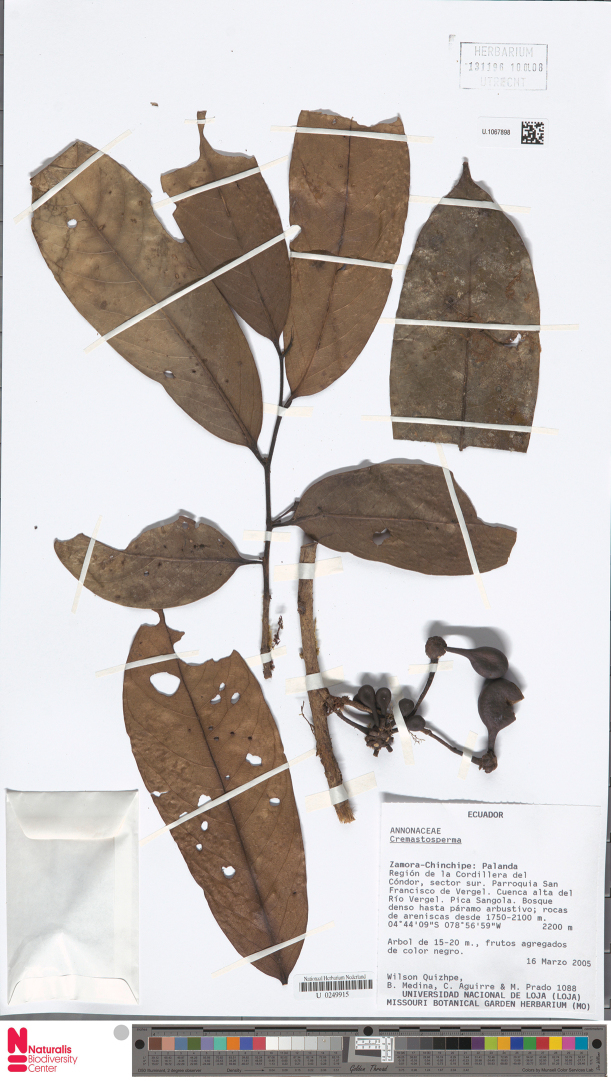
*Cremastospermaalticola* Pirie & Chatrou. Fruiting specimen (*Quizhpe et al. 1088*).

### 2. Cremastosperma antioquense

Taxon classificationPlantaeMagnolialesAnnonaceae

Pirie

Fig. 9 , Map 2

Cremastosperma antioquense Pirie, Blumea 50: 43, f. 1. 2005.

#### Type.

COLOMBIA, Antioquia: Mun. Anorí, Corregimiento Providencia, Buenos Aires, 4 km from Providencia, 500–700 m a.s.l., 10 Dec 1972, *Soejarto, D.D. 3586* (holotype: COL! [COL000334459]; isotypes: F! [V0054279F], GH! [GH00257162], HUA, MEDEL! [MEDEL000017], MO! [MO-047625]).

#### Description.

*Tree* ca. 5 m tall; young twigs and petioles sparsely covered with appressed brown hairs up to 0.2 mm long or glabrous. *Leaves*: petioles 7–10 by 2–3 mm; lamina elliptic, 16–27 by 6–9.5 cm (index 2.3–2.8), chartaceous, drying to a mosaic of brown and lighter green on both sides, glabrous on both sides, base obtuse, apex acuminate (acumen 10–15 mm long), primary vein grooved in the basal half, 1–1.5 mm wide at widest point, secondary veins 8–11, intersecondary veins occasional, distance between from 10 mm at the base to 50 mm closer to the apex, angles with primary vein from 50° at the base to 70° closer to the apex, forming distinct loops, smallest distance between loops and margin 3–4 mm, tertiary veins more or less percurrent. *Inflorescence* of single flowers, axillary on leafy twigs or from main trunk, then solitary or clustered in groups of at least two on brachyblasts; peduncles ca. 2 by 1.5 mm (in flower), 2–3 by 1.5–2 mm (in fruit); pedicels 20–28 by ca. 1 mm at the base, 1.5–2 mm at the apex (in flower), 20–40 by ca. 2 mm at the base, ca. 3 mm at the apex (in fruit), peduncles and pedicels sparsely covered with appressed whitish-golden hairs to 0.2 mm long; 2 lower bracts, deltate, ca. 1 mm long, obtuse, soon falling off; upper bract attached around halfway along pedicel, deltate, ca. 1 mm long, obtuse, outer side of upper and lower bracts rather densely to densely covered with appressed whitish-golden hairs to 0.2 mm long; closed flower buds not seen; flowers light green, stamens and carpels yellowish or pinkish *in vivo*, petals dark brown, contrasting to lighter colour of sepals and pedicels *in sicco*; sepals fused at base, deltate, appressed, 2–2.5 by 2–2.5 mm, acute, soon falling off, sparsely to rather densely covered with appressed whitish-golden hairs to 0.2 mm long; outer petals elliptic, ca. 12 by 8 mm, inner petals elliptic, 10–12 by 5–6 mm, outer side of outer and inner petals rather densely covered with appressed whitish-golden hairs to 0.2 mm long; receptacle depressed ovoid; androecium 5–7 mm diam., stamens ca. 1 mm long, connective appendage 0.5–0.7 mm wide, glabrous; gynoecium 1–1.5 mm diam., carpels ca. 1.5 mm long, glabrous. *Monocarps* ca. 10, ellipsoid to broadly so, strongly asymmetrical, 13–14 by 11 mm, orange to deep red, maturing to black *in vivo*, dark reddish-brown *in sicco*, with an excentric apicule; stipes orange to deep red *in vivo*, ca. 20 by 1.5 mm; fruiting receptacle depressed ovoid, ca. 6 mm diam; monocarps, stipes and receptacle glabrous. *Seeds* ellipsoid, reddish-brown with dark pits each surrounded by a raised rim, ca. 12 by 9 mm, raphe sunken, regular.

#### Distribution.

Colombia (Antioquia).

#### Habitat and ecology.

Primary forest. At elevations of 500–700 m. Flowering: February, fruiting: December.

#### Notes.

The distinctive strongly asymmetrical monocarps of *Cremastospermaantioquense* could only be confused with those of *C.chococola*,and collections of both species display cauliflory (though not exclusively so in *C.antioquense*) with inflorescences inserted on similar brachyblasts. However, *C.chococola* can easily be distinguished from *C.antioquense* by its somewhat smaller, narrowly elliptic leaves with typical pinkish-brown colour on the underside (no such colour contrast in the brown/green-drying leaves of *C.antioquense*) and by the absence of hairs on the pedicels. The flowers of *C.antioquense* are superficially similar to those of *C.awaense*, particularly in the dimensions of the sepals and petals and lengths of the pedicels. However, petals of *C.antioquense* are uniformly covered in indument in contrast to the distinctive indument patterning on those of *C.awaense* and the fruits of the two species are more distinct: in contrast to *C.awaense*, the monocarps of *C.antioquense* are smaller, shorter than the stipes, strongly asymmetrical and entirely glabrous. In addition, none of the collections of *C.awaense* display cauliflory, a condition found in both of the two collections of *C.antioquense*.

#### Preliminary conservation status.

None of the only three known collections of *C.antioquense* was found in protected areas. Given the low area of occupancy and a likely ongoing decline in area, extent and/or quality of the habitat, we propose to classify the species as Endangered [EN] (Table 1).

#### Other specimens examined.

**COLOMBIA. Antioquia**: Providencia, 7°30'N, 74°40'W, 500–700 m a.s.l., 12 Feb 1971, *Soejarto 2798* (COL, GH, HUA); Anorí, Vereda “La Esperanza”, 7°11'03"N, 75°01'53"W, 8 Nov 1999, *Tuberquia et al. 1416* (COL).

**Figure 9.**
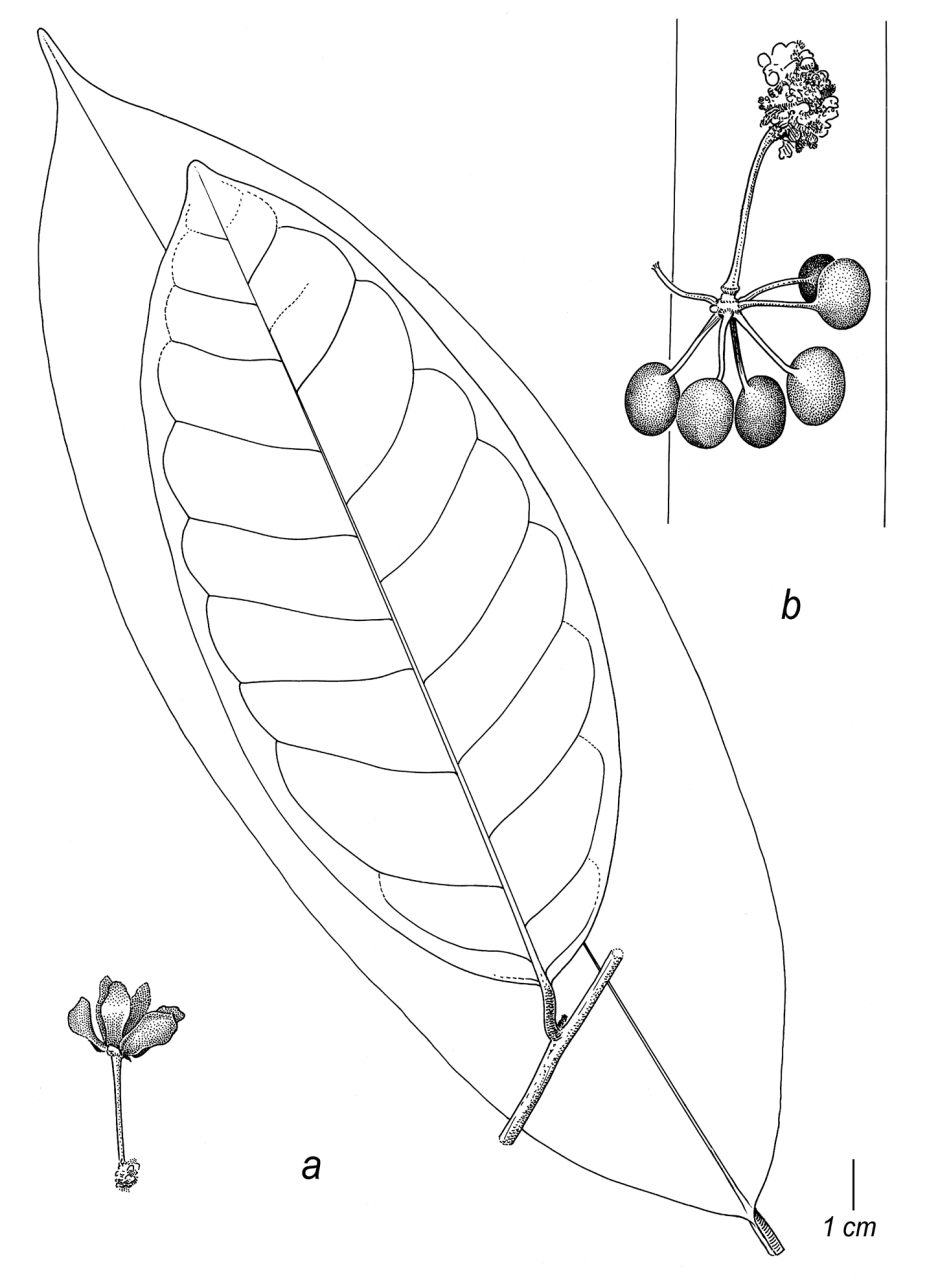
*Cremastospermaantioquense* Pirie. **a** leaf and flower **b** fruit (**a***Soejarto 2798***b***Soejarto 3586*).

### 3. Cremastosperma awaense

Taxon classificationPlantaeMagnolialesAnnonaceae

Pirie

Fig. 10 , Map 3

Cremastosperma awaense Pirie, Blumea 50: 45, f. 2. 2005.

#### Type.

ECUADOR. Carchi: Maldonado, parish of Tobar Donoso, Ethnic Reserve Awá, Sabalera, 900 m a.s.l., 22 Nov 1992, *Aulestia, C. 842* (holotype: QCNE! [QCNE87829]; isotypes: MO! [MO-1609731], U! [U0012222]), US! [US00901537].

#### Description.

*Tree* 4–15(-20) m tall, 8–25 cm diam.; young twigs and petioles sparsely to rather densely covered with appressed golden hairs to 0.3 mm long. *Leaves*: petioles 4–11(–15) by 1.5–3 mm; lamina elliptic to slightly obovate or narrowly so, 17–33 by 5.5–13 cm (index 2.2–3.6), chartaceous, brown/grey green above, darker below, veins on underside dark brown, glabrous above, veins sparsely to rather densely covered with appressed golden hairs to 0.3 mm long below, base obtuse to acute, apex acuminate (acumen 10–20 mm long), primary vein 1–3 mm wide at widest point, secondary veins 7–11, intersecondary veins occasional, distance between from 10 mm at the base to up to 60 mm closer to the apex, angles with primary vein from 45–50° at the base to 55–60° closer to the apex, forming loops in the apical half, smallest distance between loops and margin 1–3 mm, tertiary veins slightly reticulate. *Inflorescence* of single, solitary flowers, axillary on leafy or leafless twigs; peduncles ca. 1.5 by 1 mm (in flower), 1.5–3 by 1–2 mm (in fruit); pedicels 27–28 by ca. 1 mm (in flower), 35–60 by 1–2 mm (in fruit), peduncles and pedicels rather densely to densely covered with appressed golden hairs to 0.3 mm long; single lower bract, broadly elliptic, 1–2 by 1–1.5 mm, obtuse, soon falling off, outer side densely covered with appressed golden hairs to 0.3 mm long; upper bract attached around midway along the pedicel, broadly elliptic, 1–2.5 by 1–2 mm, obtuse, rather densely to densely covered with appressed golden hairs to 0.3 mm long; closed flower buds depressed ovoid; flowers green or cream *in vivo*, blackish *in sicco*; sepals free, deltate, reflexed (appressed in bud), 2–2.5 by 2–2.5 mm, obtuse, soon falling off, outer side rather densely to densely covered with appressed golden hairs to 0.3 mm long; outer petals elliptic to broadly elliptic, 10–15 by 8–9 mm, inner petals elliptic, 10–15 by 5–6 mm, sparsely to rather densely covered with appressed golden hairs to 0.2 mm long on the outer side, denser at the base and in a band leading from the base to the apex of the petals; stamens 1–1.5 mm long, connective appendage ca. 1 mm wide; gynoecium ca. 2 mm diam., carpels 30–40, 1–2 mm long, sparsely covered with golden, <0.1 mm long hairs. *Monocarps* 10–12(–20), ellipsoid, slightly asymmetrical, 22–28 by 12–17 mm, brown *in sicco*, with an excentric apicule or rarely a nipple-like protuberance; stipes 11–24 by 1–1.5 mm; fruiting receptacle depressed ovoid, 3.5–8 mm diam. monocarps, stipes and receptacle very sparsely to sparsely covered with appressed white hairs <0.1 mm long. *Seeds* ellipsoid, asymmetrical, yellow-orange with shallow pits, ca. 19 by 11 mm, raphe sunken, regular.

#### Distribution.

Pacific coast of Colombia (Chocó, Nariño) and Ecuador (Carchi, Esmeraldas).

#### Habitat and ecology.

Primary humid to premontane tropical forest. At elevations of 0–2000 m. Flowering: January, September and November; fruiting: January, February and June to September.

#### Vernacular names.

Colombia: Guasca negra (*Romero-Castañeda 3369*). Ecuador: Cargadera negra (*Tipaz et al. 1718*), Castaña negro (*Aulestia, C. et al. 1842*), Huasca negra (*Quelal et al. 191*), Teuug teiug (*Tipaz et al. 1428*).

#### Note.

*Cremastospermaawaense* can be distinguished by the unique pattern of indument on the outer sides of the petals; denser at base and in a line leading to the petal apex. The sparse indument of very short (<0.1mm) hairs on the monocarps and stipes are not visible without magnification and the fruits appear glabrous. This character is also exhibited by some specimens of *C.westrae*. *C.awaense* can easily be distinguished from both *C.westrae* and the geographically closer *C.stenophyllum* Pirie on the basis of the length of the pedicel. The pedicels of *C.westrae* are shorter (not exceeding 17 mm) and those of *C.stenophyllum* longer (ca. 45 mm in comparison to 27–28 mm in flower).

#### Preliminary conservation status.

Although the species is distributed across a moderately wide area (EOO >20,000), *C.awaense* is rather rarely collected (AOO <500 km^2^) and half of the specimens known to us are from within just one ethnic reserve in Ecuador which reportedly does not confer protected status to the biota. Decline in non-protected areas may lead to a considerable reduction in EOO and hence we assign the category Near Threatened [NT] (Table 1).

#### Selected specimens examined.

**COLOMBIA. Chocó**: Termales, between Jobi and Arusi, 5°37'N, 77°25'W, 5–50 m a.s.l., 31 Jan 1995, *Betancur et al. 6043* (COL, HUA, U, US). **ECUADOR. Carchi**: Reserva Etnica Awá-Camumbí, 0°53'N, 78°16'W, 1700–1900 m a.s.l., 20 Jul 1991, *Quelal et al. 191* (U); Tulcán, 0°53'N, 78°20'W, 1600 m a.s.l., 20 Sep 1991, *Rubio et al. 2181* (MO); Reserva Etnica Awá, sector Sabalera, 1°00'N, 78°24'W, 650–1000 m a.s.l., 19 Jun 1992, *Tipaz et al. 1428* (U); Reserva Etnica Awá, 0°53'N, 78°25'W, 1800 m a.s.l., 17 Aug 1992, *Tipaz et al. 1718* (U); Lita-Alto Tambo Road, ca. 20 km past Lita, 0°55'N, 78°30'W, 550 m a.s.l., 26 Jun 1991, *Van der Werff et al. 12045* (QCNE, U). **Esmeraldas**: Reserva Etnica Awá, 1°08'N, 78°33'W, 200 m a.s.l., 21 Sep 1992, *C. Aulestia et al. 637* (U); Bravito, 0°35'N, 79°02'W, 600 m a.s.l., 9 Sep 1998, *X. Cornejo* & *Bonifaz 6451* (U); Lita-San Lorenzo road, 1°05'N, 78°40'W, 300–500 m a.s.l., 12 May 1990, *Gentry et al. 70042* (U); Eloy Elfaro, 0°49'N, 78°45'W, 250 m a.s.l., 23 Oct 1993, *Tirado et al. 591* (MO, US); Río Santiago, 0°49'S, 78°54'W, 200 m a.s.l., 17 Jul 1994, *Tirado et al. 1083* (U).

**Map 3.**
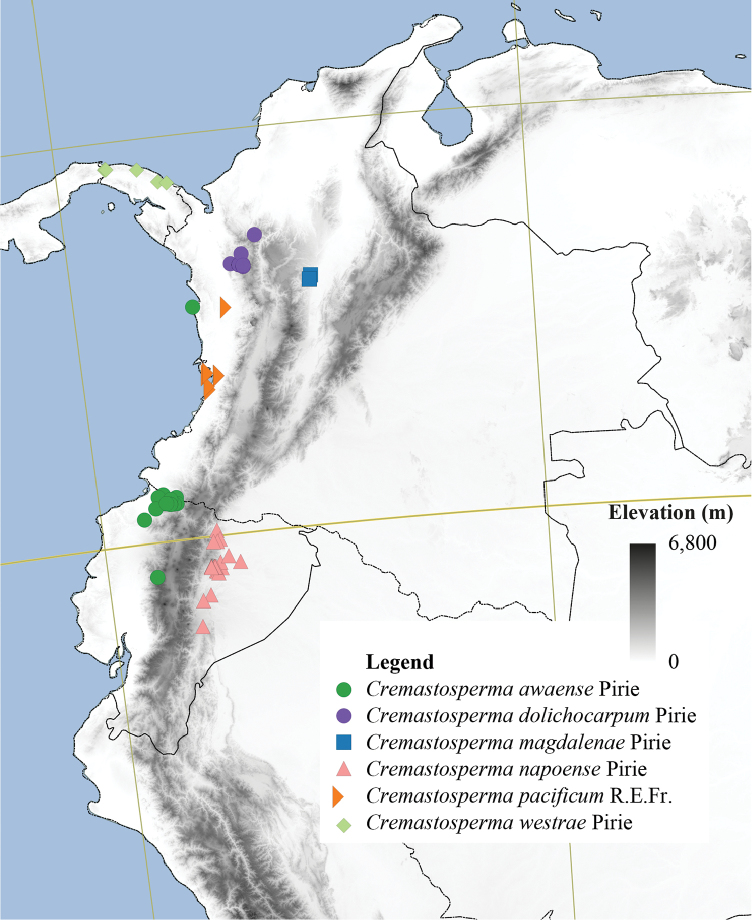
Distribution of *Cremastospermaawaense* Pirie, *C.dolichocarpum* Pirie, *C.magdalenae* Pirie, *C.napoense* Pirie, *C.pacificum* R.E.Fr. and *C.westrae* Pirie

**Figure 10.**
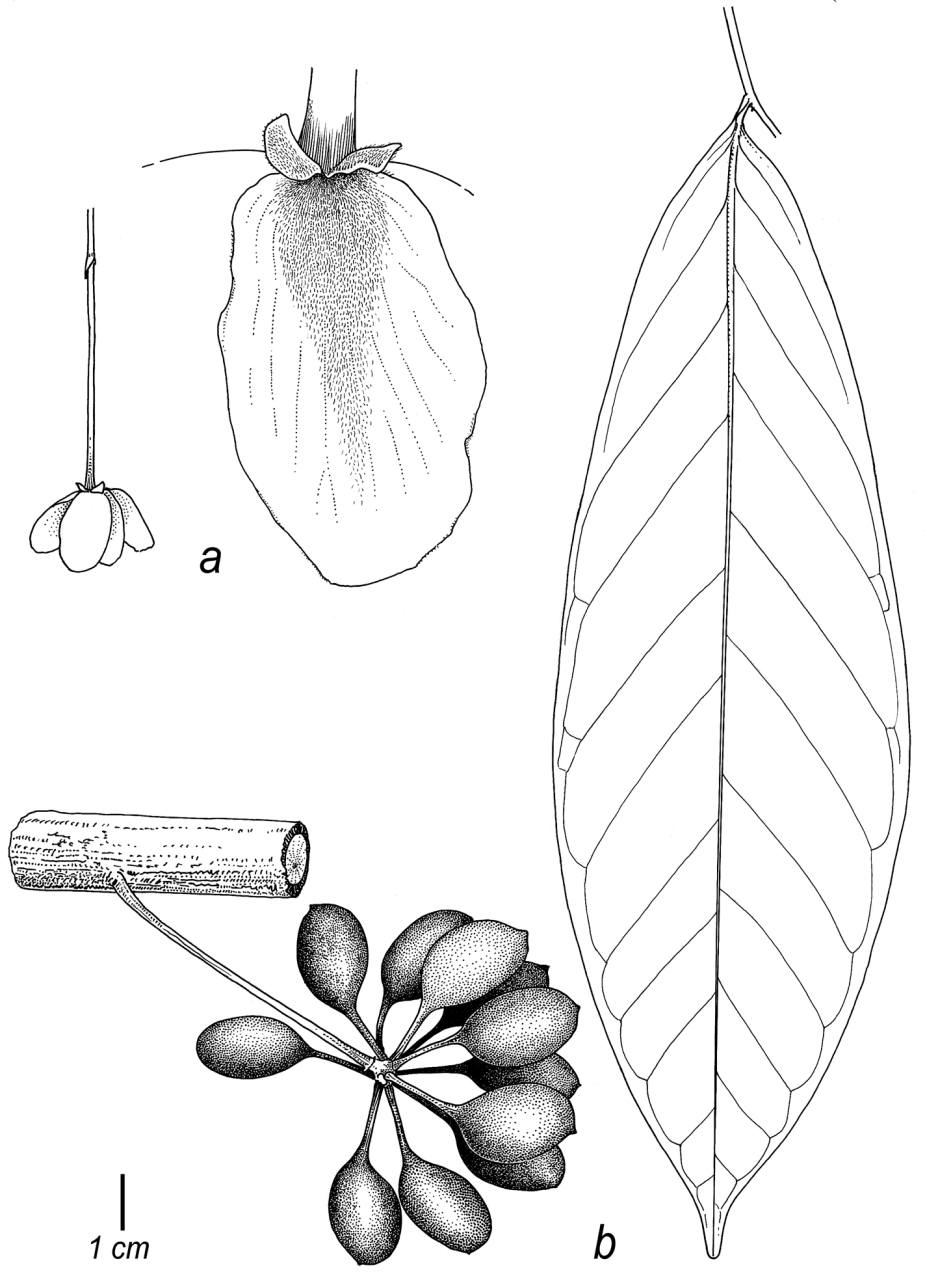
*Cremastospermaawaense* Pirie. **a** flower **b** leaf and fruit (**a***Aulestia 842***b***Van der Werff 12045*).

### 4. Cremastosperma brachypodum

Taxon classificationPlantaeMagnolialesAnnonaceae

Pirie & Chatrou sp. nov.

urn:lsid:ipni.org:names:60477507-2

Fig. 11 , Map 8

#### Diagnosis.

Most similar to *C.pendulum*, *C.confusum* and *C.monospermum* (all species also found in central and southern Peru, with glabrous flowers and fruit and smallish leaves), from which it differs in the unusually short stipes; further differs from *C.monospermum* in the shape of the flower bud (depressed ovoid, compared to broadly ovoid-triangular) and from *C.monospermum* and *C.pendulum* in the shorter pedicels.

#### Type.

PERU, San Martín: Huinguillo, 3 Mar 1962, *Woytkowski, F. 7128* (holotype: MO! [MO-047731]; isotypes: GH, UC).

#### Description.

*Tree* ca. 8 m tall; young twigs and petioles glabrous. *Leaves*: petioles 4–7 by 1–2 mm; lamina elliptic to obovate, 11–17 by 4.5–7 cm (leaf index 2.3–2.4), chartaceous, olive green, more greyish above, glabrous, base acute to obtuse, apex acuminate (acumen 8–10 mm long), primary vein raised over entire leaf length, ca. 1 mm wide at widest point, glabrous, secondary veins 5–6, intersecondary veins ca. 1–2, distance between varying along length (15 mm to ca. 25 mm), angles with primary vein from ca.70° at the base to ca.60° closer to the apex, not branching, forming distinct loops, smallest distance between loops and margin 3–4 mm, tertiary veins reticulate. *Inflorescence* of single flowers, axillary; short axillary shoot, ca. 1 by 0.5 mm (in flower), ca. 2 by 1 mm (in fruit); pedicels 21–28 by 1 mm at the base, 2.5 mm at the apex (in flower), ca. 44 by 1–3 mm at the base, ca. 3 mm at the apex (in fruit), short axillary shoot and pedicels glabrous; 2 lower bracts, soon falling off; upper bract attached at between one third and half of the pedicel length, deltate, ca. 1 mm long by ca. 1 mm diam., apex rounded, ciliate; closed flower buds depressed ovoid; flowers creamy white *in vivo*, dark brown *in sicco*; sepals connate for ca. 1 mm, deltate, somewhat recurved, 2–2.5 by 2.5–3 mm, rounded, persistent, glabrous; outer petals ovate, 11–13 by 8–9 mm, inner petals elliptic to ovate, 8–12 by 4–5 mm, glabrous, ciliate; receptacle ovoid, apex concave; androecium ca. 3 mm diam., stamens ca. 100, 1.5–2 mm long, connective appendage ca. 1 mm wide; gynoecium ca. 2 mm diam., carpels ca. 15, glabrous. *Monocarps* ca. 13, ellipsoid, slightly asymmetrical, 7–9 by 6–7 mm, without apicule, blackish-brown *in sicco*; stipes ca. 2 by ca. 2 mm; fruiting receptacle elipsoid, 6 mm diam.; monocarps, stipes and receptacle glabrous. *Seeds* not seen.

#### Distribution.

Peru (San Martín).

#### Habitat and ecology.

Forest. At an elevation of ca. 500 m. Flowering and fruiting: March.

#### Notes.

*Cremastospermabrachypodum*, known only from the type, is distinctive within *Cremastosperma* due to the unusual shortness of the stipes, but otherwise typical of species of the southern/montane clade in the absence of indument on all parts.

#### Etymology.

The specific epithet “*brachypodum*” is derived from the Greek brachy (short) and podum (-stalked), referring to the short stipes.

#### Preliminary conservation status.

This new species is only known from a single collection dating back to 1962 in a non-protected, rural area. Critically endangered [CR] (Table 1).

**Figure 11.**
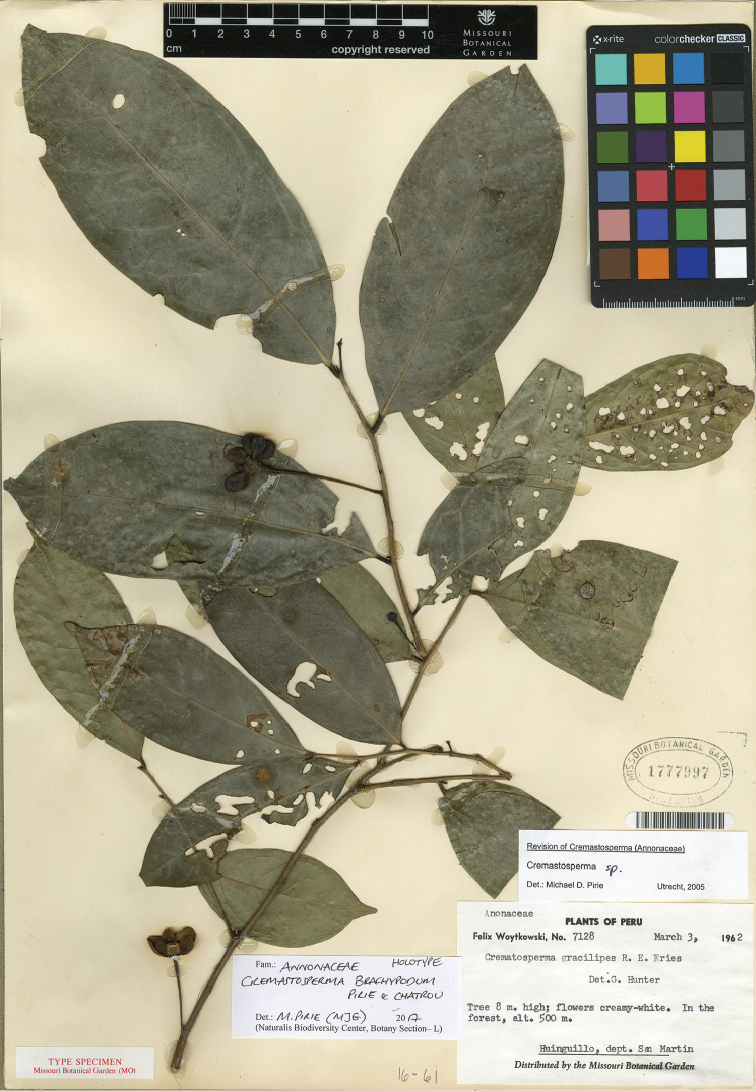
*Cremastospermabrachypodum* Pirie & Chatrou. Flowering and fruiting specimen (*Woytkowski 7128*).

### 5. Cremastosperma brevipes

Taxon classificationPlantaeMagnolialesAnnonaceae

(DC.) R.E.Fr.

**Map 4.**
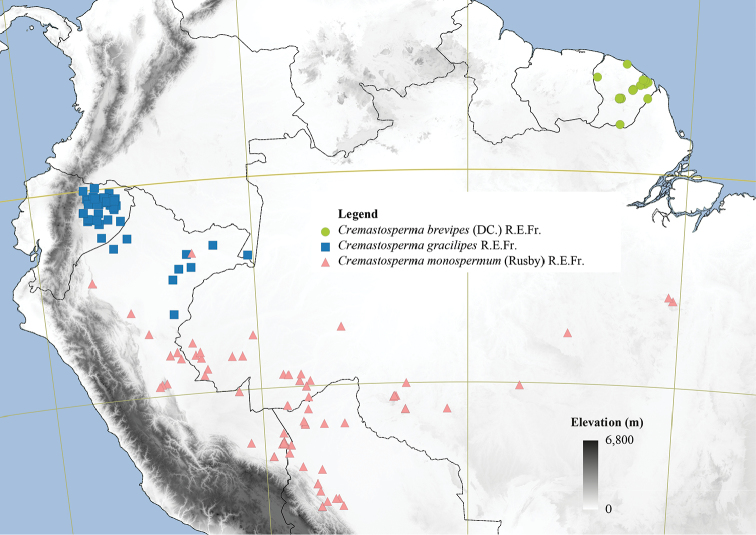
Distribution of *Cremastospermabrevipes* (DC.) R.E.Fr.; *C.gracilipes* R.E.Fr.; *C.monospermum* (Rusby) R.E.Fr.

**Figure 12.**
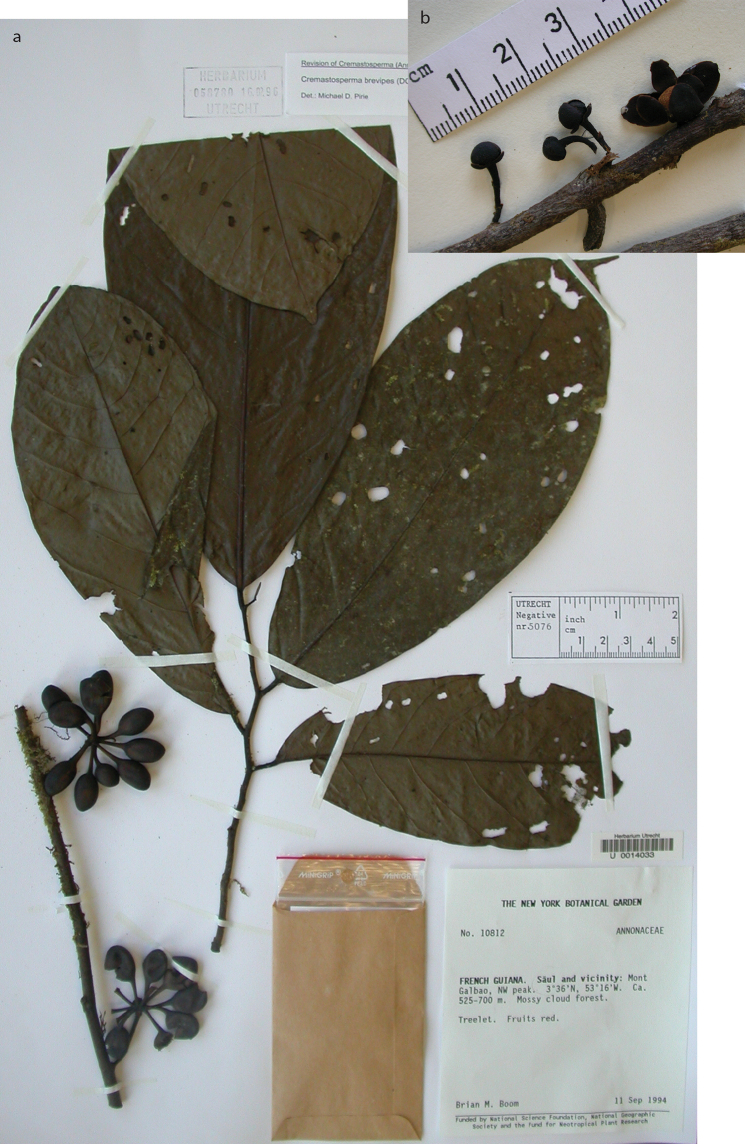
*Cremastospermabrevipes* (DC.) R.E.Fr. **a** fruiting specimen **b** flower buds (**a***Boom 10812***b***Prévost 3446*).

**Figure 13.**
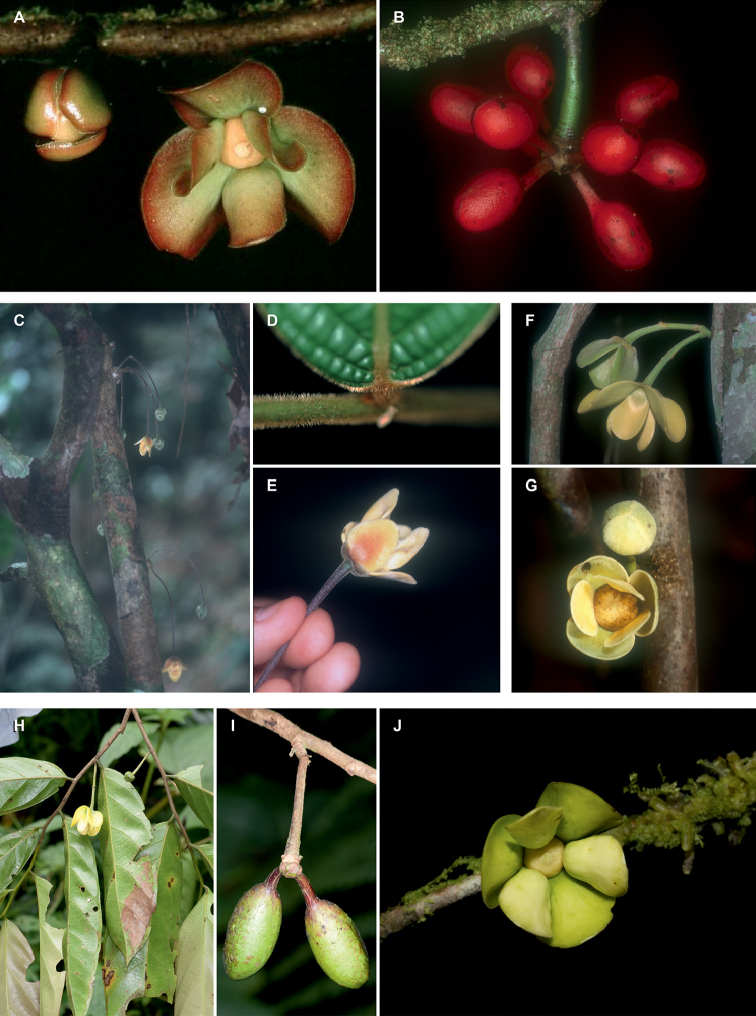
**a–b***Cremastospermabrevipes* (DC.) R.E.Fr. **a** flowers (*Maas et al. 8064*; photo PJMM) **b** fruit (*Mori et al 22721*; photo Scott Mori) **c–e***C.bullatum* Pirie **c, e** Flowering specimen (*Pirie et al. 94*; photos **c** MDP **e** Robin van Velzen) **d** leaf base showing bullate corrugations of the lamina and long golden indument (Pirie et al. 71; photo: MDP) **f, g***C.cauliflorum* R.E.Fr. Flowering specimens (**f***Maas et al. 9029*, photo PJMM **g***Chatrou et al. 224*, photo LWC) **h–j***C.dolichocarpum* Pirie. Flowering and fruiting specimen (*Pedraza et al. 2146*; photos: María F. González).

Figs 12 , 13 , Map 4

Cremastosperma brevipes (DC.) R.E.Fr., Acta Horti Bergiani 12: 538. 1939.
Guatteria brevipes DC. in Dunal, Monogr. Annonac.: 126. 1817.

#### Type.

FRENCH GUIANA, without location *Martin s.n.* (lectotype: G! [designated in Pirie, Kankainen & Maas, 2005], isotypes: BM, K! (uncertain whether this specimens represents an isotype) [K000485530]).

*Cremastospermapoiteaui* (Diels) R.E.Fr., Acta Horti Bergiani 10: 328. 1931.

*Guatteriapoiteaui* Diels, Notizbl. Bot. Gart. Berlin-Dahlem 11: 74. 1931.

#### Type.

FRENCH GUIANA, without location, 1819–1821, *Poiteau, A. s.n.* (holotype: G! [G00237255 G00237256]; isotypes: B! [B 10 0242372], F! [F932330], S! [S-R-7016]).

#### Description.

*Tree* 4–20 m tall, 3–20 cm diam.; young twigs and petioles glabrous to sparsely covered with appressed white/yellowish hairs to 0.2 mm long. *Leaves*: petioles 4–9(–12) by 1.5–4 mm; lamina elliptic to obovate or narrowly so, 18–39 by 7–15 cm (index 2–3.5), chartaceous, brown/green with a reddish tinge on both sides (particularly on the veins on the underside), darker above, glabrous on both sides, base obtuse to rounded, rarely acute, apex acuminate (acumen 5–20 mm long), primary vein 1.5–3 mm wide at widest point, secondary veins 9–15, intersecondary veins 0–2, distance between from 5–10 mm at the base to 35 mm closer to the apex, angles with primary vein from 45–60° at the base to 55–70° closer to the apex, not branching, forming mostly distinct loops, smallest distance between loops and margin 1–3 mm, tertiary veins percurrent. *Inflorescence* of single flowers, solitary or clustered in groups of 2, on leafless twigs; peduncles 1–2 by 1–2 mm (in flower), 2–5 by ca. 2 mm (in fruit), sparsely to rather densely covered with appressed white/yellowish ca. 0.1 mm long hairs; pedicels 15–20 by 1–2 mm at the base (in flower), 18–23 by ca. 2 mm (in fruit), sparsely covered with appressed white/yellowish hairs ca. 0.1 mm long or glabrous; 2 lower bracts, deltate, basal to 0.2 mm long, apical 0.3–0.5 mm long, obtuse, mostly persistent, rather densely covered with appressed white/yellowish hairs ca. 0.1 mm long; upper bract mostly attached midway along pedicel, deltate to broadly ovate, 1–2 by ca. 1 mm, obtuse or emarginate, sparsely covered with appressed white/yellowish hairs ca. 0.1 mm long; closed flower buds depressed ovoid, opening in development; flowers green, sometimes tinged with red around margins of petals or creamy yellow *in vivo*, reddish or dark brown *in sicco*, sepals and petals glabrous; sepals connate for 0.5–1 mm, broadly ovate, recurved, rarely appressed, 3–4[-5] by 3.5–5 mm, obtuse, soon falling off; outer petals elliptic, 12–22 by 7–12 mm, rounded, inner petals narrowly obovate to narrowly elliptic, 10–24 by 4–7 mm, obtuse; androecium ca. 6 mm diam.; stamens 1.4–1.6 mm long, connective appendage 0.5–0.7 mm wide; gynoecium ca. 1 mm diam.; carpels ca. 25, ca. 2.5 mm long, glabrous. *Monocarps* 7–17, ellipsoid, slightly asymmetrical, 11–17 by 9–11 mm, green maturing to red, reddish-brown, dark purple or black *in vivo*, blackish or reddish-brown *in sicco*, with an excentric apicule; stipes green maturing to red *in vivo*, 7–14 by 1–1.5(3) mm; fruiting receptacle depressed ovoid, 4–8 mm diam., monocarps, stipes and receptacle glabrous. *Seeds* broadly ellipsoid to globose, yellowish or orange-brown, slightly pitted, ca. 8 by 7–8 mm, raphe raised within a sunken groove, somewhat irregular.

#### Distribution.

French Guiana, region of Saül and Nouragues.

#### Habitat and Ecology.

Primary moist forest. At elevations of 200–800 m. Flowering: February, March, May and October; fruiting: May – October, December and January.

#### Vernacular names.

French Guiana: Apélému (Wayapi; *Grenand 1509*, *Jacqemin 2349*), Maman yawé (Créole; *Moretti 12*).

#### Notes.

*Cremastospermabrevipes* is the only species of the genus found in the Guianas. The leaves, when dried, have a characteristic reddish tinge (particularly on the underside). This species is similar to *C.venezuelanum*, but differing in particular by the acuminate as opposed to acute or obtuse leaf apex of *C.venezuelanum*, smaller sepals and monocarps and shorter stipes. Its use as fish bait has been reported. As of many Annonaceae, the bark of *C.brevipes* has been described as aromatic: on collection of *Riera 668*, a peppery smell is reported.

#### Preliminary conservation status.

*C.brevipes* has been collected regularly, occurring over a fairly wide area, including the protected area of Les Nouragues. Least concern [LC] (Table 1).

#### Selected Specimens Examined

**. FRENCH GUIANA** Rivière Arataye, Saut Pararé, 4°02'N, 52°42'W, 3 Dec 1985, *Barrier 5166* (CAY, L, P, U); Saül, Mont Galbao, 3°36'N, 53°16'W, 525–700 m a.s.l., 11 Sep 1994, *Boom 10812* (CAY, NY, U); Mont Inéri, 4°22'N, 52°10'W, 10 m a.s.l., 9 Sep 1997, *Cremers 15331* (U); Trésor Reserve, Creek 4, middle part, 4°35'N, 52°16'W, *Ek et al. 1615* (U); Régina, Montagne Tortue, 4°18'N, 52°22'W, 200–450 m a.s.l., 17 Jun 1988, *Feuillet et al. 10239* (U); Piste de Saint-Elie, 5°20'N, 53°00'W, 29 Nov 1987, *De Granville 10176* (CAY, P, U, US); Approuague, 4°28'N, 52°02'W, 28 Jul 1997, *Hequet 432* (P, U); Pedra Alice, River Oiapoque, 3°40'N, 52°01'W, 17 Sep 1960, *Irwin et al. 47562* (NY, U); Route de Bélizon, Les Eaux Claires-Saül, 3°37'N, 53°12'W, 200 m a.s.l., 9 Feb 1993, *Maas et al. 8064* (MO, U); Saül, Piste de Galbao, 4°36'89"N, 54°16'25"W, 28 Aug 2003, *Munzinger* & *Heuret 1818* (P); Crique Cacao, 2°20'N, 53°12'W, 2 May 1987, *Prévost* & *Sabatier 2284* (CAY, U); Montagne de Kaw, 4°33'N, 52°10'W, 21 Feb 1998, *Prévost 3446* (NY, U); Les Nouragues, 4°03'N, 52°42'W, 8 Oct 1992, *Riera 1564* (P); Les Nouragues, basin of River Approuague, 4°04'57"N, 52°40'39"W, 89–140 m a.s.l., Oct 2001, *Scharf 76* (U).

### 6. Cremastosperma bullatum

Taxon classificationPlantaeMagnolialesAnnonaceae

Pirie

**Map 5.**
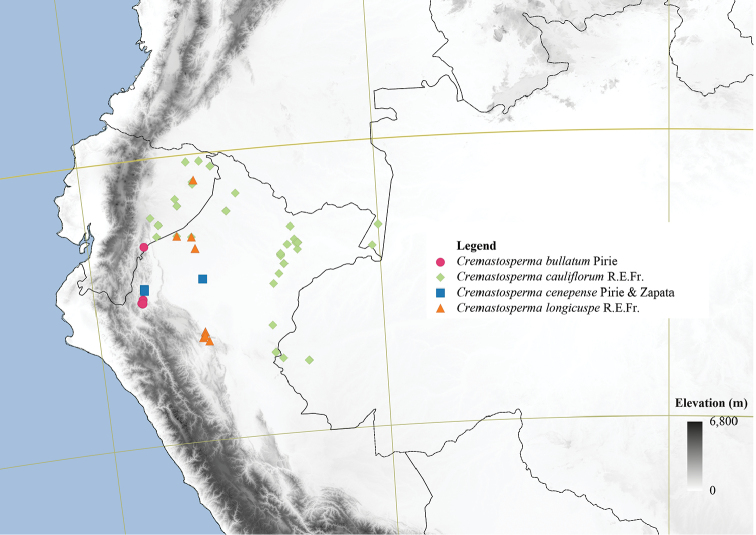
Distribution of *Cremastospermabullatum* Pirie; *C.cauliflorum* R.E.Fr.; *C.cenepense* Pirie & Zapata; and *C.longicuspe* R.E.Fr.

Figs 12 , 13 , Map 5

Cremastosperma bullatum Pirie, Arnaldoa 11: 8 f. 2, 3–5. 2004.

#### Type.

PERU, Amazonas: Bagua, Distr. Imaza, community Yamayakat, trail to Putuim, 360 m a.s.l., 22 Nov 2003, *Pirie, M.D. et al. 71* (holotype: U! [two sheets U0121238, U0121239]; isotypes: AAU!, AMAZ!, CUZ!, E! [E00268265], F! [V0047939F], HAO!, HUT!, K! [K000580475], MO! [MO-1459050], MOL!, NY! [NY00689082], US! [US00901687], USM! [USM000035], WU! [WU0038419]).

#### Description.

*Tree* 2–10 m tall; young twigs and petioles densely covered with mainly erect golden hairs up to 1 mm long. *Leaves*: petioles 3–7 mm by 2.5–3 mm; lamina elliptic or narrowly so to slightly obovate, 17–28 by 6–11 cm (index 2.4–3.5), chartaceous, mid-brown, occasionally slightly grey above (immature leaves drying black), sparsely covered with mainly erect golden hairs up to 1 mm long or glabrous above, densely so on edge of lamina and on all veins below, base rounded to subcordate, apex acuminate (acumen 5–20 mm long), primary, secondary and tertiary veins sunken in depressions in leaf surface, primary vein 1.5–2 mm wide at widest point, densely covered with mainly erect golden hairs up to 1 mm long above and below, secondary veins 15–20 (intersecondary veins rare), distance between from 6 mm at the base to 16 mm closer to the apex, angles with primary vein consistently around 60–70°, occasionally branching, forming distinct loops, smallest distance between loops and margin 1–1.5 mm; tertiary veins mostly percurrent. *Inflorescences* of single, successively produced, flowers, axillary on leafy branches, on leafless branches and produced from the main trunk (then on brachyblasts); peduncles and pedicels sparsely to rather densely covered with mainly erect golden hairs up to 1 mm long, peduncles 17–20 by 1–1.5 mm (in flower), 18–25 by 1.5 mm (in fruit); pedicels up to 120 by 1 mm at the base (in flower), 110–150 by 1.5 mm (in fruit); bracts densely covered with mainly erect golden hairs up to 1 mm long, single lower bract, elliptic to ovate, ca. 2.5 by 1 mm, acute, persistent or falling off; upper bract within central third of pedicel length, elliptic to ovate, 2–3 by 1–2 mm, acute; flower buds depressed ovoid, developing to ovoid before opening; flowers green, maturing to yellow with a basal orange patch on the outside of the outer petals *in vivo*, golden brown *in sicco*, outer sides and apical portion of the inner sides of petals and outer sides of sepals densely covered in appressed golden hairs up to 1 mm long, inner sides otherwise glabrous; sepals basally connate, deltate, 5–7 by 6 mm, acute, soon falling off, outer petals broadly ovate, ca. 18 by 15 mm, inner petals ovate, concave, ca. 25 by 12 mm; androecium ca. 5 mm diam., stamens 1–1.5 mm long, connective appendage ca. 0.5 mm wide; gynoecium [ca. 2.5] mm diam., glabrous. Monocarps 8–10, dark brown *in sicco*, ellipsoid, slightly asymmetrical, ca. 15 by 11 mm, often with an excentric apicule; stipes 14–16 by 1.5 mm; fruiting receptacle 5–6 mm diam., monocarps, stipes and receptacle sparsely to moderately densely covered with erect golden hairs up to 0.2 mm long. *Seeds* ellipsoid, orange-brown, shallowly pitted, ca.13 by 10 mm, raphe raised, regular.

#### Distribution.

Ecuador (Morona-Santiago) and Peru (Amazonas).

#### Habitat and Ecology.

Primary forest, on red clay. At elevations of 300–500 m. Flowering: February and November; fruiting: November and June.

#### Notes.

*Cremastospermabullatum* can easily be distinguished from all other species of *Cremastosperma* by any one of the number of unique and striking characteristics it displays. The leaf blade has a corrugated (bullate) appearance, both in the field and when pressed, which is due to the deeply sunken nature of the primary, secondary and tertiary venation. The indument present on many of its parts is far longer than in any other species in the genus and, also uniquely in the genus, densely inserted in a halo-like formation around the leaf margin. Other notable characteristics are the unusually long pedicel, the orange colouring of the base of the outer petals of mature flowers, the inner petals considerably longer than the outer petals and the rounded to subcordate shape of the leaf base.

#### Preliminary conservation status.

*Cremastospermabullatum* is known from a small number of locations, within a limited area of northern Peru and adjacent Ecuador outside of protected areas. Endangered [EN] (Table 1).

#### Selected Specimens Examined.

**ECUADOR. Morona-Santiago**: Región de la Cordillera del Cóndor, 3°05'13"S, 78°04'23"W, 380 m a.s.l., 1 Jun 2006, *Wisum* & *Kajekai 446* (US). **PERU. Amazonas**: Bagua, Yamayakat, 4°55'S, 78°19'W, 320 m a.s.l., 20 Jan 1996, *Jaramillo et al. 942* (MO, U); Bagua, Yamayakat, trail to Putuim, 5°03'09"S, 78°20'58"W, 343 m a.s.l., 22 Nov 2003, *Pirie et al. 66* (HAO, U, USM); Bagua, Putuim, 5°01'44"S, 78°22'43"W, 339–359 m a.s.l., 25 Nov 2003, *Pirie et al. 94* (AMAZ, CUZ, HAO, HUT, MO, U, USM).

### 7. Cremastosperma cauliflorum

Taxon classificationPlantaeMagnolialesAnnonaceae

R.E.Fr.

Figs 2b, e , 13 , 15 , Map 5

Cremastosperma cauliflorum R.E.Fr. Acta Horti Bergiani 10: 330. 1931.

#### Type.

PERU, Loreto: Mishuyacu, near Iquitos, 100 m a.s.l., Feb-Mar 1930, *Klug, G. 902* (holotype: B! [B 10 0242371]; isotypes: F! [F0054580F], NY! [NY00025860], S! [S-R-6959], US! [US00104263]).

#### Description.

*Tree* 2–20 m tall, 4–25 cm diam.; young twigs and petioles glabrous to rather densely covered with appressed or erect golden hairs to 0.5 mm long. *Leaves*: petioles 4–12(–16) by 2–4(–6) mm; lamina elliptic to obovate or narrowly so, (14–)20–61 by 5–14(–22) cm (index 2.3–3.7), chartaceous, olive/brown green above, darker below, glabrous above except for base of primary vein sparsely covered with appressed or erect hairs to 0.3 mm long, base, primary and secondary veins sparsely to rather densely covered with appressed or erect golden hairs to 0.5 mm long below, base acute to obtuse, apex acuminate (acumen 5–45 mm long), primary vein verrucose (particularly at the base), deeply grooved for most of length, 1.5–3.5(–5) mm wide at widest point, secondary veins (6–)10–17, occasionally 1–2 intersecondary veins, distance between from 4 mm at the base to up to 40 mm closer to the apex, angles with primary vein from 45–70° at the base to 45–60° closer to the apex, not branching, forming mostly distinct loops, smallest distance between loops and margin 1–5 mm, tertiary veins percurrent. *Inflorescence* of 1–5 flowers, branching, solitary or clustered in groups of up to 7, on thick leafless twigs or on main trunk (then often on brachyblasts); peduncles 3–12(–15) by 1–1.5(–3) mm (in flower), 3–15 by 1–3 mm (in fruit); pedicels 10–45 by 1–3 mm at the base (in flower), 15–45 by 1–3 mm (in fruit), peduncles and pedicels rather densely to densely covered with mainly erect golden hairs ca. 0.3 mm long, often with hairs more densely covering the articulation point between shoot and pedicel; single lower bract (from the axil of which short shoots develop bearing new flowers), deltate, 1.5–2 mm long, acute, soon falling off, densely covered with mostly appressed golden hairs to 0.3 mm long; upper bract attached around midway along pedicel, broadly to very broadly ovate or deltate, 2–4 mm long, obtuse or acute, outer side densely covered with appressed or erect golden hairs to 0.3 mm long; closed flower buds depressed ovoid, opening in development; flowers (pale) green, creamy white, greenish-yellow or yellow *in vivo*, brownish-yellow or brown with orange, dark brown or black base *in sicco*, outer side of sepals and petals densely covered with erect or appressed golden hairs to 0.4 mm long, inner side of sepals and petals sparsely to rather densely covered with erect hairs to 0.4 mm long or glabrous, base glabrous; sepals free, broadly to very broadly ovate-deltate, mostly recurved, 3–5 by 4–6 mm, obtuse, soon falling off; outer petals elliptic to broadly elliptic, 10–25(–32) by 9–17 mm, inner petals elliptic, 11–21(–32) by 6–11 mm; androecium 7–10 mm diam., stamens 1.5–2 mm long, connective appendage 0.7–1 mm wide; gynoecium 2–3 mm diam., carpels ca. 40, 2–2.3 mm long, sparsely to rather densely covered with mostly appressed golden hairs to 0.2 mm long. *Monocarps* 9–41, globose to transversely broadly ellipsoid, slightly asymmetrical, 8–13 by 10–14 mm, green maturing to orange, red, brown and black *in vivo*, blackish-brown or brown *in sicco*, sometimes with an apicule at or near the apex; stipes 7–23(–32) by 1–2 mm; fruiting receptacle depressed ovoid, 4–11 mm diam; monocarps, stipes and receptacle rather densely covered with erect golden hairs to 0.2 mm long. *Seeds* broadly ellipsoid to globose, orange, pitted, 9–10 by 9–10 mm, raphe sunken, regular.

#### Distribution.

Amazonian Colombia (Amazonas, Putamayo), Ecuador (Morona-Santiago, Napo, Pastaza, Sucumbíos), Peru (Loreto) and Brazil (Acre, Amazonas).

#### Habitat and ecology.

Moist primary forest, mostly non-inundated areas, on clayey or lateritic soil or white sand. At elevations of 100–500 m. Flowering: June, August, October-February; fruiting: May-February.

#### Vernacular names.

Colombia: Espintana blanca (*Raffauf 102*); Jiobo ñaatraje duceju (*J. Murillo 565*). Ecuador: Mantach (Shuar; *Warush Juwa RBAE 119*), Moncapatahue (Huaorani; M. Aulestia 3238), Nanguehue (*M. Aulestia 3395*), Piton (Quech; *Cazalet et al. 7528*), Uñetahue (Huaorani; *Rubio 812*), Uñitague (Huaorani; *Espinosa 399*). Peru: Bara (*Rimachi 480*), Bara caspi (*McDaniel 20761*, *Rimachi 2397*), Espintana (*Cheta 6/173*, *Rimachi 2397*), Mantaach (Achual Jivaro; *W.H. Lewis 12049*).

#### Notes.

*Cremastospermacauliflorum* is one of only three species of the genus displaying a branched inflorescence. It can be discerned from *C.napoense* and *C.alticola* by the presence of indument on the monocarps and stipes and by the greater length and density of hairs on the inflorescences. The wood is aromatic, flowers reported as vanilla scented.

#### Preliminary conservation status.

*Cremastospermacauliflorum* is one of the more widespread and abundant species of the genus, found in protected areas in Colombia and Ecuador, as well as in Northern Peru. Least concern [LC] (Table 1).

#### Selected Specimens Examined.

**BRAZIL. Acre**: Mun. Mancio Lima, Rio Moa, 7°25'S, 73°38'W, 12 Oct 1989, *Cid Ferreira et al. 10019* (NY); Cruzeiro do Sul-Boa Fé road, km 12, 7°34'S, 72°44'W, 2001, *Maas et al. 9029* (U); Mun. Cruzeiro do Sul, BR-307, 7°34'S, 72°45'W, 2001, *Maas et al. 9251* (U). **COLOMBIA. Amazonas**: Tarapaca, 3°02'S, 70°00'W, 100 m a.s.l., 30 Jun 1992, *Rudas et al. 4514* (MO); Parque Nacional Amacayacu, 3°45'S, 70°15'W, 100 m a.s.l., 8 Aug 1989, *Vásquez et al. 12655* (MO). **Putamayo**: Puerto Leguízamo, Puerto Leguízamo-La Tagua road, 0°92'00"S, 74°46'40"W, 19 Jan 2000, *Suárez 1331* (COL). **ECUADOR. Morona-Santiago**: Taisha, 2°23'S, 77°30'W, 500 m a.s.l., 14 Jun 1980, *Brandbyge et al. 31854* (AAU, U); El Centro Shuar Pampants, 2°47'S, 77°36'W, 300 m a.s.l., 11 Sep 1985, *Warush Juwa RBAE 119* (U); Cordillera de Cutucú, 2°06'48"S, 77°44'39"W, 610 m a.s.l., 16 Jul 2007, *Wisum* & *Kajekai 1165* (WAG). **Napo**: Reserva Etnica Huaorani, 0°59'S, 76°12'W, 235 m a.s.l., 18 Jan 1995, *Aulestia* & *Omehuat 3238* (QCNE); Río Wai si ayá, 0°15'S, 76°21'W, 300 m a.s.l., 13 Aug 1981, *Brandbyge et al. 33503* (AAU, MO, NY, U); Río Aguarico, E of mouth of Río Cuyabeno, 0°16'S, 75°54'W, 200 m a.s.l., 20 Feb 1980, *Holm-Nielsen et al. 21501* (AAU, K, MO, U); Cuyabeno Wildlife Reserve, 0°29'S, 75°32'W, 230 m a.s.l., 25 Sep 1991, *Palacios 7602* (U). **Pastaza**: Pozo petrolero ‘Danta 2’ de UNOCAL, 1°47'S, 76°48'W, 365 m a.s.l., 1 Oct 1990, *Espinoza* & *Coba 399* (MO, U); Pozo petrolero ‘Ramirez’, 1°32'S, 76°51'W, 300 m a.s.l., 21 Feb 1990, *Zak 5272* (U). **PERU. Loreto**: Yanamono Explorama Reserve, 3°27'S, 72°51'W, 100–150 m a.s.l., 31 Dec 1998, *Chatrou et al. 224* (MOL, U); Allpahuayo-IIAP, 3°50'S, 73°25'W, 150 m a.s.l., 2 Jan 1999, *Chatrou et al. 233* (L, MOL); Prov. Requena (locality unknown), 4°50'S, 73°45'W, 170 m a.s.l., 8 Aug 1985, *Cheta 6/ 173* (K); Prov. Maynas, NW of Zona Protectado Pucacuro, 2°08'13"S, 75°08'58"W, 160–270 m a.s.l., 26 Aug 2006, *Dávila et al. 2784* (L); Nauta-Iquitos road, 4°30'S, 73°32'W, 140 m a.s.l., 27 Jun 1979, *C. Díaz 1229* (F, MO, U); Caserío Gamitana, Reserva del Río Mazán, 3°30'S, 73°10'W, 116 m a.s.l., 21 Jun 1990, *Grández et al. 1591* (LPB, MO, U); Prov. Maynas, NW of Area de Conservación Ampiyacu, 2°52'40"S, 73°00'46"W, 150–160 m a.s.l., 16 Oct 2009, *Huamantupa et al. 12928* (L); Las Amazonas, ExplorNapo Camp, 3°20'S, 72°55'W, 100–140 m a.s.l., 15 Feb 1991, *Pipoly et al. 12982* (MO, U); Prov. Requena, Quebrada Yanayacu-Río Tapiche, 6°15'49"S, 73°54'31"W, 140–180 m a.s.l., 16 Oct 2014, *Ríos et al. 4491* (F, L); Andoas, 2°55'S, 76°25'W, 210 m a.s.l., 5 Jun 1981, *Vásquez* & *N. Jaramillo 1938* (F, MO, U); Maniti, Recreo, 3°42'S, 72°50'W, 115 m a.s.l., 14 May 1988, *Vásquez 10637* (F, MOL, NY, U); Iquitos-Nauta road, 4°10'S, 73°20'W, 150 m a.s.l., 14 Dec 1988, *Vásquez* & *N. Jaramillo 11423* (MO, U, USM); Puerto Almendras, 3°48'S, 73°25'W, 122 m a.s.l., 20 Jun 1989, *Vásquez* & *T. Soto 12358* (MO, U, USM).

**Figure 14.**
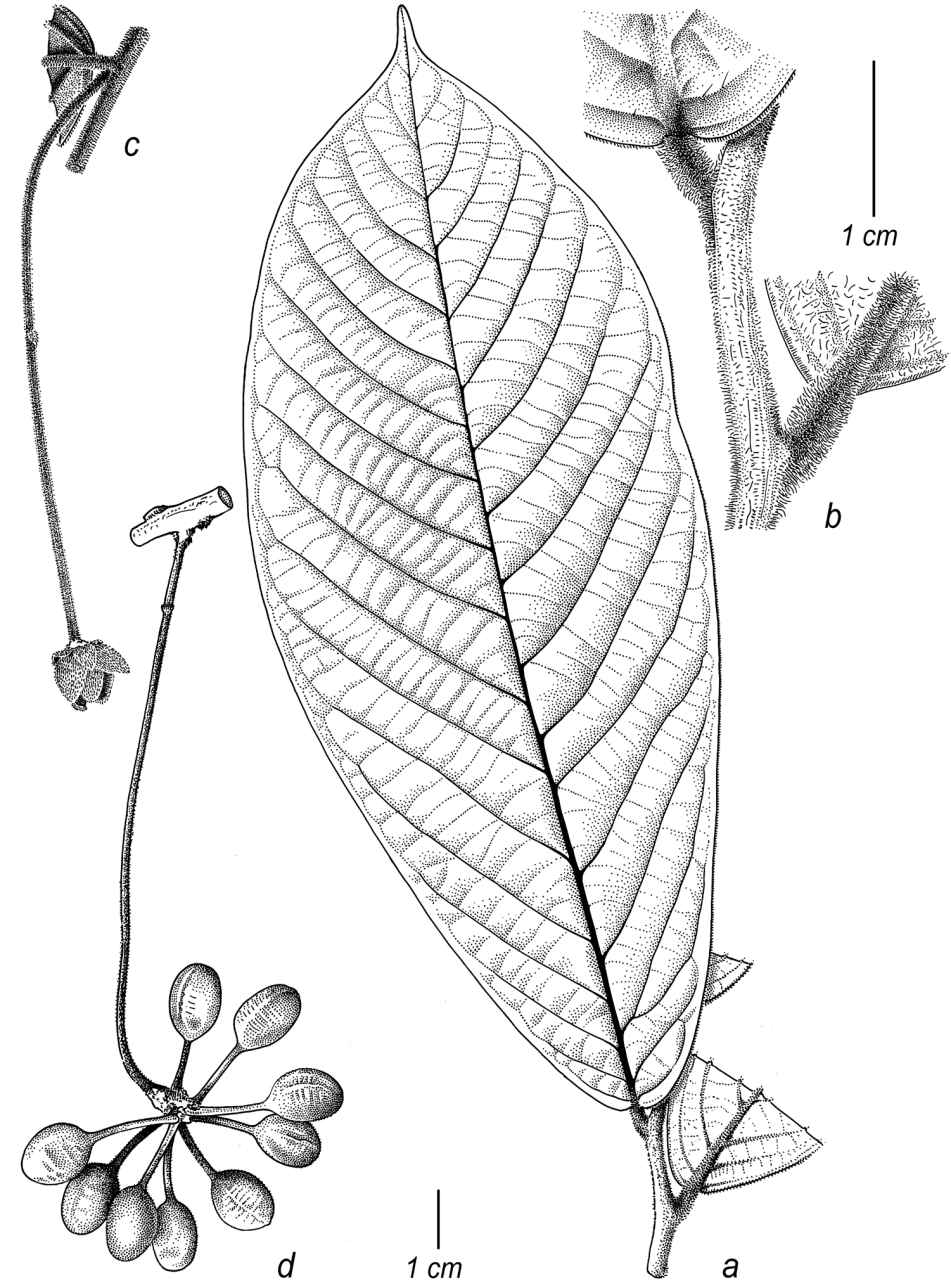
*Cremastospermabullatum* Pirie **a** leaf **b** leaf base **c** flower **d** fruit (**a–c***Vásquez et al. 24891***d***Jaramillo, N. et al. 972*).

**Figure 15.**
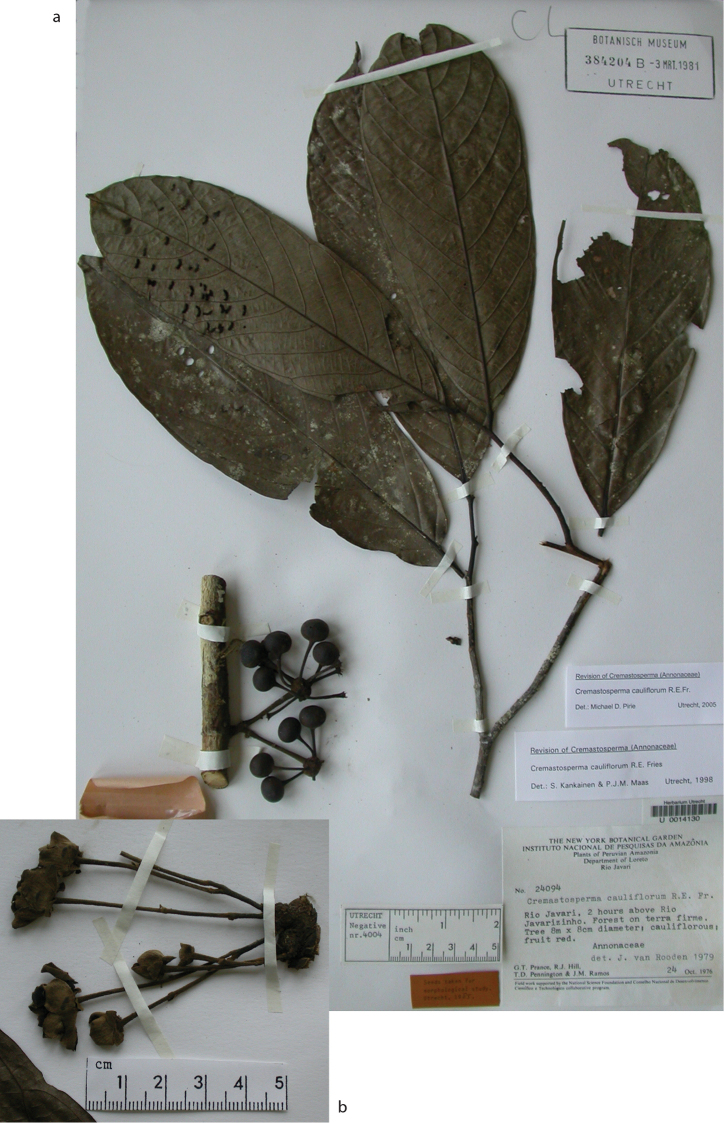
*Cremastospermacauliflorum* R.E.Fr. **a** fruiting specimen **b** inflorescence (**a***Prance et al. 24094***b***Vasquez & N. Jaramillo 11423*).

### 8. Cremastosperma cenepense

Taxon classificationPlantaeMagnolialesAnnonaceae

Pirie & Zapata

Fig. 16 , Map 5

Cremastosperma cenepense Pirie & Zapata, Arnaldoa 11: 13, f. 2, 9. 2004.

#### Type.

PERU, Amazonas: prov. Condorcanqui, Río Cenepa region, community Mamayaque, 11 Aug 1997, *Rojas, R. et al. 269* (holotype: U! (barcode U0123477]; isotypes: AMAZ, HUT, MO [MO-1664376], USM).

#### Description.

*Tree* ca. 10 m tall; young twigs and petioles sparsely (axillary buds densely) covered with appressed golden hairs ca. 0.1 mm long. *Leaves*: petioles 4–7 by 1–2 mm; lamina elliptic to narrowly so, 12–22 by 4–8 cm (index 2.7–3), chartaceous, grey-yellow green above, light brown or yellowish-green below, glabrous on both sides, base cordate to subcordate, apex acuminate (acumen 8–10 mm long), primary vein 1–1.5 mm wide at widest point, secondary veins 7–12, intersecondary veins occasional, distance between from 2–5 mm at the base to 15–25(35) mm closer to the apex, angles with primary vein from 80–90° at the base to 50–60° closer to the apex, forming distinct loops, smallest distance between loops and margin 2–5 mm, tertiary veins more or less percurrent. *Inflorescence* of single, solitary flowers, axillary on leafy twigs; peduncles ca. 2 by 2 mm (in fruit); pedicels ca. 8 by 2 mm at the base (in fruit), peduncles and pedicels sparsely covered with appressed golden hairs ca. 0.1 mm long; 2 lower bracts, soon falling off; upper bract attached midway along pedicel, soon falling off; closed flower buds and flowers not observed. *Monocarps* 8–10, blackish-brown *in sicco*, ellipsoid, slightly asymmetric, 14–15 by 9–11 mm, with an excentric apicule; stipes 7–8 mm by ca. 1.5 mm; fruiting receptacle 4–7 mm diam.; monocarps, stipes and receptacle rather densely covered with appressed golden hairs ca. 0.1 mm long. *Seeds* ellipsoid, golden brown shallowly wrinkled (immature), ca. 12 by 7 mm, raphe sunken, regular.

#### Distribution.

Peru (Amazonas, in the area of the Cenepa River, a tributary of the Marañon River).

#### Habitat and Ecology.

Primary forest. At elevations of 250–400 m. Flowering: not recorded; fruiting: July and August.

#### Vernacular names.

Peru: Yais (Amarun; *R. Rojas et al. 0255*, *0269*)

#### Notes.

*Cremastospermacenepense* is most likely to be confused with *C.yamayakatense* and *C.gracilipes*, which are more commonly collected in northern Peru and also characterised by relatively small leaves and fruits. It differs in the shape of the leaf base (cordate or subcordate as opposed to acute in *C.yamayakatense* and *C.gracilipes*), the indument on the fruits (rather dense as opposed to almost always absent in *C.yamayakatense*) and lengths of the pedicel (shorter than that of *C.gracilipes*) and stipes (shorter than those of *C.yamayakatense*).

#### Preliminary conservation status.

Three of the only four known collections of *C.cenepense* are from more or less the same locality and none was found in protected areas. Given the low area of occupancy and a likely ongoing decline in area, extent and/or quality of the habitat, we propose to classify the species as Endangered [EN] (Table 1).

#### Other specimen examined.

**PERU. Amazonas**: Río Cenepa region, Quebrada Nahem, 780 ft a.s.l., 15 July 1974, *Kayap 1078* (MO, U); Río Cenepa region, community Mamayaque, 4°34'S, 78°14'W, 400 m a.s.l., 9 Aug 1997, *R. Rojas et al. 0 255* (U); Río Cenepa region, community Mamayaque, 4°37'08"S, 78°13'46"W, 400 m a.s.l., 18 Aug 1997, *Rojas et al. 0351* (MO).

**Figure 16.**
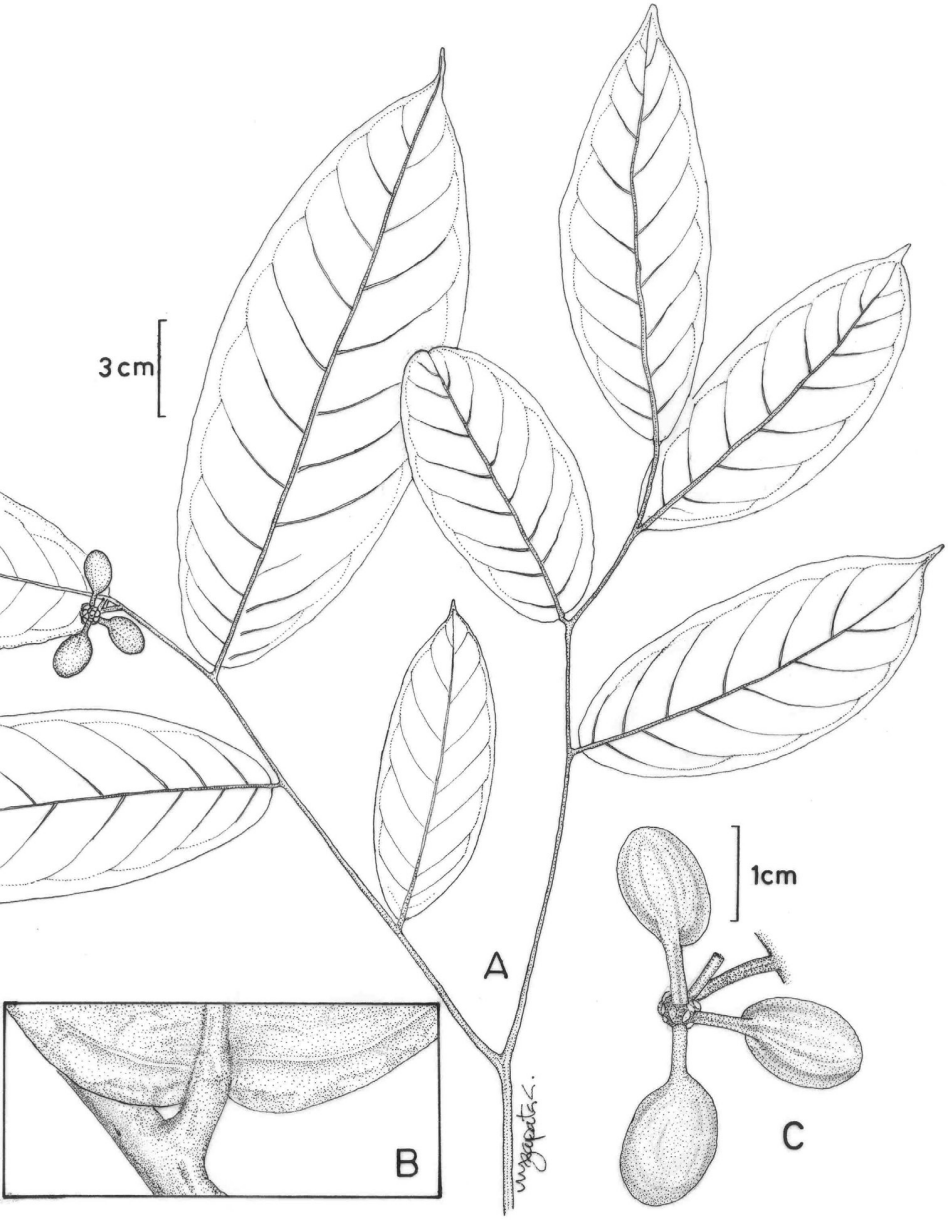
*Cremastospermacenepense* Pirie & Zapata. **a** fruiting twig **b** leaf base **c** fruit (**a, c***Rojas 269*; **b***Kayap 1078*).

### 9. Cremastosperma chococola

Taxon classificationPlantaeMagnolialesAnnonaceae

Pirie

Fig. 17 , Map 2

Cremastosperma chococola Pirie, Blumea 50: 47, f. 3. 2005.

#### Type.

COLOMBIA, Chocó: Alto de Buey, 500–1200 m a.s.l., 8 Jan 1973, *Gentry, A.H. & Forero, E. 7286* (holotype: MO! [MO-047629]; isotype: COL! [COL000214771]).

#### Description.

*Tree* ca. 5 m tall; young twigs and petioles glabrous. *Leaves*: petioles 5–8 by 1.5–2 mm; lamina narrowly elliptic, 11–20 by 4–5.5 cm (index 3.7–4), chartaceous, dark/olive brown, shiny above, lighter pinkish-brown, matt below, glabrous above and below, base acute to cuneate, apex acuminate (acumen 7–10 mm long), primary vein ca. 1 mm wide at widest point, verrucose below, secondary veins 8–10, no intersecondary veins, distance between from 5 mm at the base to 30 mm closer to the apex, angles with primary vein from ca. 60° at the base to 60–70° closer to the apex, forming distinct loops, smallest distance between loops and margin 2.5–3.5 mm, tertiary veins reticulate. *Inflorescence* of single flowers, solitary or clustered in groups of at least two, on brachyblasts on the main trunk; peduncles, 2–3 by 1–1.5 mm (in fruit); pedicels 38–42 by 1 mm at the base, 1 mm at the apex (in fruit), peduncles and pedicels glabrous; lower bract(s) not observed; upper bract attached within basal half of pedicel, ovate, ca. 1 by 0.7 mm, obtuse, glabrous; flowers not observed. *Monocarps* 10–13, ellipsoid, strongly asymmetrical (stipes inserted within basal half of longest axis), 13–14 by 10–11 mm, with an excentric, to 0.2 mm long, apicule, green maturing through red to dark blue *in vivo*, dark brown *in sicco*; stipes 15–18 by ca. 1 mm increasing to 1.5 diam. when mature; fruiting receptacle depressed ovoid, 4–5 mm diam.; monocarps, stipes and receptacle glabrous. *Seeds* ellipsoid, orange-brown, pitted, 9–11 by 6–8 mm, raphe sunken, regular.

#### Distribution.

Pacific coast of Colombia (Chocó).

#### Habitat and Ecology.

Tropical wet forest. At elevations of 0–1200 m. Flowering: not recorded; fruiting: January and June.

#### Notes.

The strongly asymmetric monocarps of *Cremastospermachococola* resemble those of *C.antioquense*: see above for distinctions.

#### Preliminary conservation status.

Only three collections of *C.chococola* are known to us, from different localities but within a restricted region and only one of which is in a protected area. Endangered [EN] (Table 1).

#### Other specimens examined.

**COLOMBIA. Chocó**: Punta Lanas, 1 September 1989, *Espina et al. 3188* (HUA, MO); Parque Nacional Utría, 6°20'N, 77°20'W, 0–100 m a.s.l., 5 Jun 1990, *F. García* & *Agualimpia 390* (MO).

**Figure 17.**
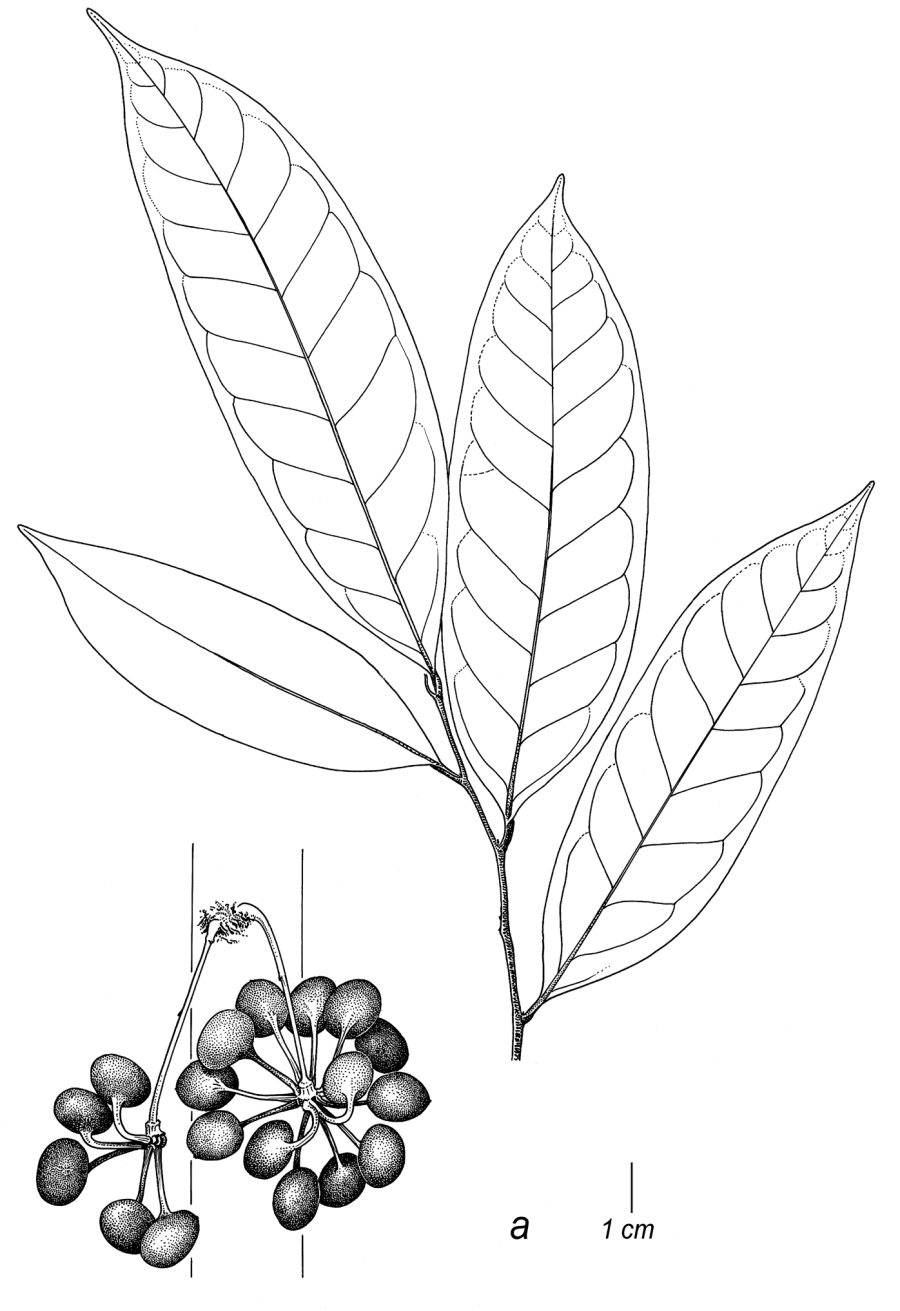
*Cremastospermachococola* Pirie. **a** leaf and fruit (*Gentry & Forero 7286*).

### 10. Cremastosperma confusum

Taxon classificationPlantaeMagnolialesAnnonaceae

Pirie sp. nov.

urn:lsid:ipni.org:names:60477508-2

Fig. 18 , Map 6

#### Diagnosis.

*Cremastospermaconfusum* is most similar to *C.monospermum*, which produces effectively indistinguishable small fruits on slender pedicels. *C.confusum* differs from *C.monospermum* in the flower buds, which open in development, unlike in *C.monospermum* in which flower buds remain closed with a characteristic roughly triangular shape. The flowers of *C.confusum* are more similar to those of *C.leiophyllum*, from which it differs in the shape and colour of the monocarps, which, unlike those of *C.leiophyllum*, do not dry black and are not asymmetrical.

#### Type.

PERU, Madre de Dios: Tambopata, Explorer’s Inn, near the confluence of Río Tambopata and Río La Torre, 39 km SW of Puerto Maldonado, along the Big Tree Trail, 21 Jan 1989, *Smith, S.F. et al. 1578* (holotype: USM; isotypes: U! [U0012270], NY).

#### Description.

*Tree* or *shrub* 3–8 m tall, 3–20 cm diam.; young twigs and petioles sparsely covered with appressed whitish hairs to 0.3 mm long. *Leaves*: petioles 7–10 by 1.5–3 mm; lamina elliptic to obovate or narrowly so, 10–28(–34) by 7–10(–12) cm (index 1.9–3.5), chartaceous to coriaceous, green (or greenish-brown), darker above, lighter or more brown with darker or reddish veins below, glabrous above, sparsely covered with appressed whitish hairs to 0.3 mm long particularly on veins below, base acute to obtuse (rarely rounded), apex acuminate (acumen 6–20 mm long), primary vein 1.5–3 mm wide at widest point, secondary veins 7–10, intersecondary veins 0–1, distance between from 11–20 mm at the base to 12–32 mm closer to the apex, angles with primary vein from 70–80° at the base to 40–50° closer to the apex, rarely branching, mostly forming distinct loops, smallest distance between loops and margin 2–5 mm, tertiary veins mostly percurrent. *Inflorescence* of single (very rarely branching) flowers, solitary, axillary on leafy or leafless twigs or thicker branches; peduncles (1-)3–5 by 1–2 mm (in flower), 3–9 by ca. 2 mm (in fruit), rather densely covered with appressed to erect whitish hairs to 0.2 mm long; pedicels 20–45(–70) by ca. 1 mm at the base (in flower), 22–80 by 1–2.5 mm (in fruit), pink, purple or reddish *in vivo*, glabrous; 2 lower bracts of unequal dimensions, basal lower bract depressed ovate, ca. 0.5 by 1 mm, obtuse, sometimes persistent, densely covered with appressed to erect whitish hairs to 0.2 mm long, apical lower bract elliptic, ca. 1.5 by 1 mm, obtuse, sometimes persistent, sparsely to rather densely covered with appressed to erect whitish hairs to 0.2 mm long; upper bract attached around midway along pedicel, (broadly) ovate, 1.5–2 by ca. 1–1.5 mm, obtuse or rounded, persistent, sparsely covered with appressed to erect whitish hairs to 0.2 mm long; closed flower buds depressed ovoid, opening in development; flowers green maturing to yellow or white and yellowish at base *in vivo*, dark to yellowish light brown sometimes tinged with red, darker at the base *in sicco*, sepals and petals glabrous; sepals free, deltate to triangular, appressed to recurved, ca. 3 by 2–3 mm, acute or obtuse, soon falling off or sometimes briefly persistent; outer petals elliptic, 14–17 by 6–8 mm, inner petals elliptic to narrowly so, 16–17 by 5–7 mm; androecium ca. 6 mm diam., connective appendage to 0.8 mm wide; gynoecium ca. 1.5 mm diam., carpels glabrous. *Monocarps* 10–25(–32), ellipsoid (broadly so when immature), slightly asymmetrical, 8–12 by 6–8 mm, green maturing to greenish-purple and brown *in vivo*, light to dark brown or blackish *in sicco*, with an excentric apicule; stipes 8–13 by 2 mm; fruiting receptacle 4–10 mm diam.; monocarps, stipes and receptacle glabrous. *Seeds* ellipsoid, light brown, shallowly pitted, ca. 11 by 6 mm, raphe sunken, regular.

#### Distribution.

Bolivia (La Paz), Peru (Cuzco, Madre de Dios).

#### Habitat and ecology.

Primary and secondary moist and wet forest, occasionally on floodplains. At elevations of 210–670 m. Flowering: August – December; fruiting: January – May, August and October.

#### Notes.

A number of the specimens of *Cremastospermaconfusum* have in the past been identified as *C.leiophyllum* or *C.monospermum*, two well known species found relatively nearby in Bolivia and widespread across Bolivia, Brazil and Peru, respectively. The distributions of *C.confusum* and *C.leiophyllum* do not overlap, but in the area of Madre de Dios and adjacent La Paz, Bolivia, *C.monospermum* and *C.confusum* may both occur and, in this area in particular, non-flowering specimens of the two are often not discernible. These species are closely related as well as morphologically similar and further data to test their boundaries and the potential for gene-flow between species/populations are warranted. Variation in the size, shape and texture of leaves and length of pedicel of *C.confusum* is relatively wide, with specimens collected in Cuzco in particular exhibiting larger leaves.

#### Etymology.

The species is named *C.confusum* because of the past confusion caused by its similarities to different other nearby species and the lack of unambiguous diagnostic characters for the fruiting material.

#### Preliminary conservation status.

*Cremastospermaconfusum*, found in southern Peru and the Las Paz region in northern Bolivia, has a fairly wide EOO and is not uncommon. It is also found within protected areas. Least concern [LC] (Table 1).

#### Selected specimens examined.

**BOLIVIA. La Paz**: Ixiamas, 13°08'34"S, 68°03'59"W, 18 October 2009, *Couvreur 159* (L, NY); Ixiamas, 13°00'39"S, 68°04'17"W, 29 October 2009, *Couvreur 262* (L, NY); Ixiamas, 13°59'16"S, 67°48'24"W, 29 October 2009, *Couvreur 267* (L, NY). **PERU. Cuzco**: Camisea, Campamento San Martín-C, 11°47'08"S, 72°41'57"W, 467 m a.s.l., 12 January 1997, *Acevedo-Rodríguez et al. 8635* (MO, P, U, USM); Prov. Cuzco (locality unknown), 11°47'00"S, 72°41'57"W, 467 m a.s.l., 26 Jan 1997, *Acevedo-Rodríguez et al. 9132* (USM); La Convención, Manguyari, 12°47'S, 72°40'W, 670 m a.s.l., 3 Feb 1989, *Núñez Vargas et al. 10146* (MO, U); Camanti, 13°13'S, 70°45'W, 643 m a.s.l., 18 Feb 1991, *Núñez Vargas 12951* (U, USM); La Convención, Distr. Echarati, 11°41'S, 73°00'W, 350 m a.s.l., 18 Apr 1998, *Núñez Vargas, et al. 21737* (USM); Camanti, Maniri, 13°17'S, 70°48'W, 720 m a.s.l., 15 Oct 1990, *Timaná 1010* (MO); Camanti, 13°17'19"S, 70°46'27"W, 26 Feb 2007, *Valenzuela Gamarra, L et al. 8643* (CUZ, HUT, MO, USM). **Madre de Dios**: Parque Nacional Manu, Cocha Cashu, 11°52'S, 71°22'W, 29 Sep 1976, *Foster* & *Terborgh 5083* (US); Parque Nacional Manu, Tayakome, 11°41'S, 71°36'W, 350–400 m a.s.l., 28 Sep 1986, *Foster* & *Achille 11495* (U, USM); Tambopata Reserve, Río Tambopata, 12°50'S, 69°17'W, 250 m a.s.l., 5 Mar 1981, *Gentry et al. 32009* (MO); Shintuya-Salvación road, 12°40'S, 71°15'W, 500 m a.s.l., 14 May 1984, *Knapp* & *Mallet 6450* (F, NY, US); Las Piedras, 12°36'23"S, 69°04'54"W, 17 Aug 2004, *Suclli* & *Huamantupa 1943* (MO); Tambopata Reserve, Explorer’s Inn, 12°47'S, 69°41'W, 270 m a.s.l., 21 Sep 1998, *Vásquez Chávez et al. 25605* (L, MO, MOL); Tambopata Reserve, 12°15'S, 69°17'W, 260 m a.s.l., 21 Nov 1984, *Young* & *Stratton 219* (MO, U). **Puno**: Ridge between Río Candamo and Río Guacamayo, 13°30'S, 69°50'W, 400–600 m a.s.l., 22 May 1992, *Gentry et al. 76947* (MO).

**Map 6.**
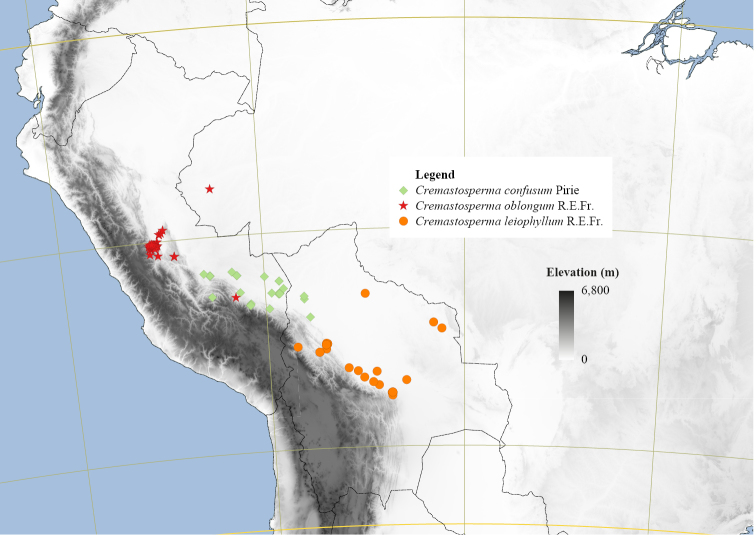
Distribution of *Cremastospermaconfusum* Pirie, *C.leiophyllum* R.E.Fr.; and *C.oblongum* R.E.Fr.

**Figure 18.**
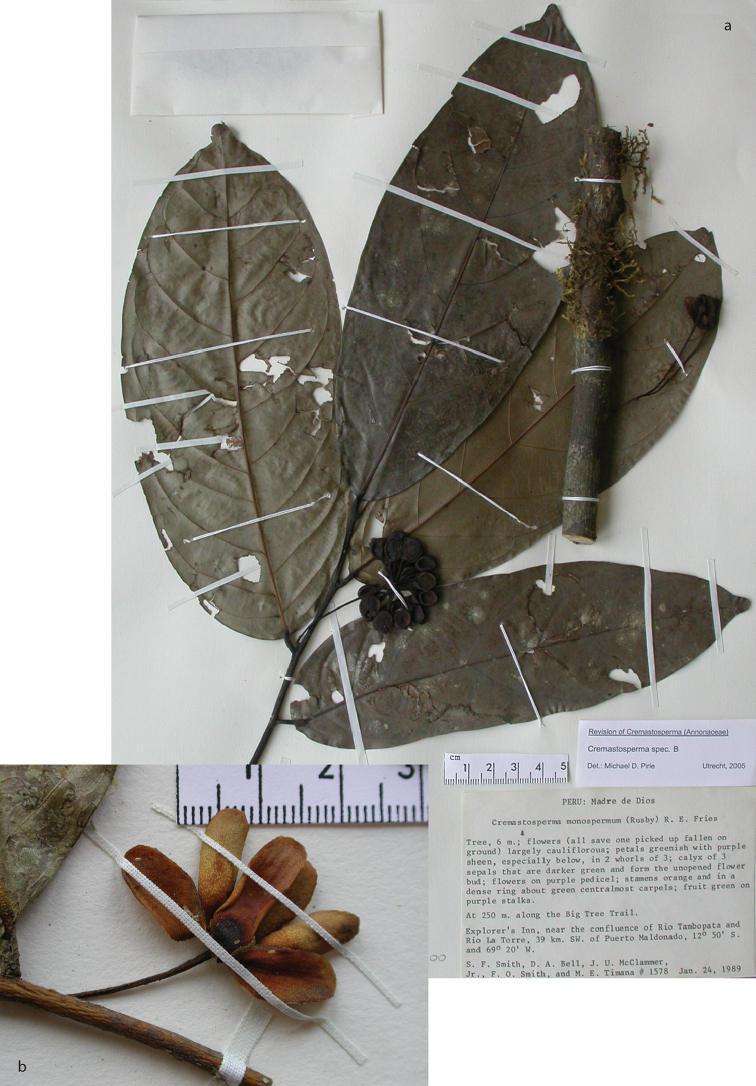
*Cremastospermaconfusum* Pirie. **a** fruiting and flowering specimen **b** flower (**a***Smith, S. et al. 1578***b***Smith, S. et al. 794*).

### 11. Cremastosperma dolichocarpum

Taxon classificationPlantaeMagnolialesAnnonaceae

Pirie

**Figure 19.**
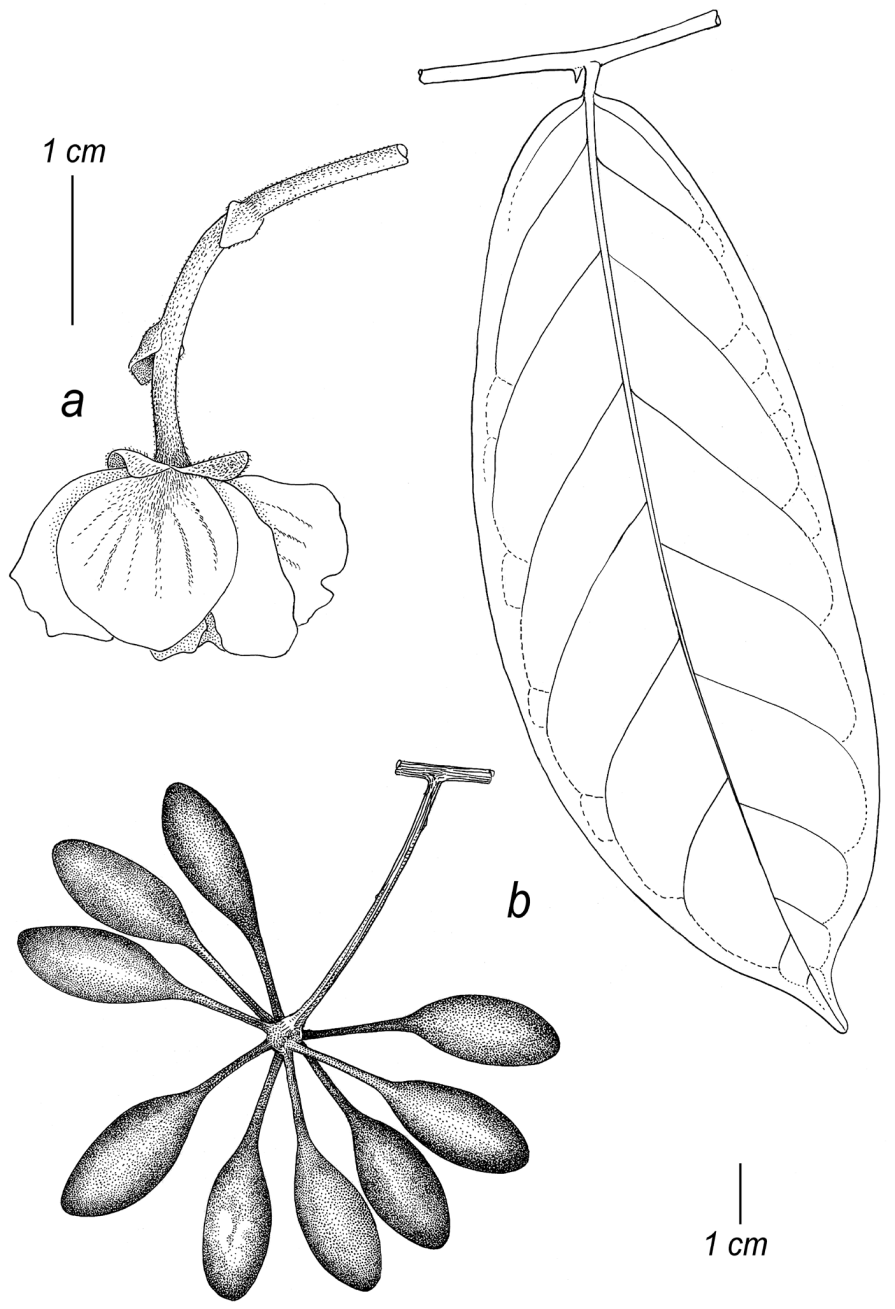
*Cremastospermadolichocarpum* Pirie. **a** flower **b** leaf and fruit (**a***Callejas 3110***b***Sánchez 415*).

Figs 13 , 19 , Map 3

Cremastosperma dolichocarpum Pirie, Blumea 50: 49, f. 4. 2005.

#### Type.

COLOMBIA, Antioquia: Frontino, Nutibara, upper watershed of Río Cuevas, 15 Jul 1986. *Sánchez, D. et al. 415* (holotype: U [U0012253]; isotypes: COL! [COL000221555], MEDEL! [MEDEL000018, MEDEL000019]).

#### Description.

*Tree* 6–18 m tall, 15–22 cm diam.; young twigs and petioles sparsely covered with white-yellow appressed hairs 0.3–0.5 mm long. *Leaves*: petioles 3–6 by 1.5–2 mm, often with warts extending up primary vein; axillary buds densely covered with white-yellow appressed hairs 0.3–0.5 mm long; lamina narrowly elliptic to elliptic, 14–24.5 by 6–10 cm (index 1.6–3.1), chartaceous to subcoriaceous, mid-dark brown above, lighter below, glabrous above, sparsely covered with white-yellow appressed hairs 0.3–0.5 mm long (particularly on veins) below, base obtuse-acute (narrowly cuneate), apex acuminate (acumen 5–15 mm long), primary vein not conspicuously grooved, 1–2 mm wide at widest point, secondary veins (5–)7–9(–11), intersecondary veins occasional, distance between from 5 mm at the base to 30 mm closer to the apex, angles with primary vein 40–50° at the base to 50–70° closer to the apex, not branching, forming distinct loops in the apical half-two thirds, smallest distance between loops and margin 1.5–4 mm, tertiary veins percurrent with significant reticulation. *Inflorescences* of single flowers solitary or clustered in groups of 2 (or more), produced from leafy twigs or leafless branches; peduncles of two internodes, the second 1.2–4 by 1 mm (in flower), approx. 2 by 2 mm (in fruit); pedicels 28–47 by 1 mm at the base, 1–1.5 mm at the apex (in flower), 40–55 by 1.5–2 mm at the base, 2–2.5 mm at the apex (in fruit); peduncles and pedicels rather densely covered with white-yellow appressed hairs 0.3–0.5 mm long; two lower bracts (one on each internode), the apical one persisting later into flowering, 1–1.5 by 0.7–1 mm, broadly ovate, obtuse, soon falling off, rather densely covered with white-yellow appressed hairs 0.3–0.5 mm long; upper bract broadly to narrowly ovate, 1–3.5 by 1–2 mm, obtuse, persistent, densely covered with white-yellow appressed hairs 0.3–0.5 mm long; closed flower buds depressed ovoid; flowers green maturing to yellow *in vivo*, brown outside and black inside *in sicco*; sepals free, ovate, appressed, 3–4 mm long, obtuse, occasionally persistent on less mature fruit, densely covered with white-yellow appressed hairs 0.3–0.5 mm long; outer petals ovate to broadly so, 10–15 by 9–11 mm, inner petals ovate, 10–16 by 7–8 mm densely covered with white-yellow appressed hairs 0.3–0.5 mm long; receptacle ovoid to depressed ovoid; androecium 3–5 mm diam., stamens 1–1.2 mm long, connective appendage ca. 1 mm wide; gynoecium 1.5–2 mm diam., carpels 0.5–0.6 mm long, glabrous. *Monocarps* 10–20 (fully ripe fruit not observed), black *in sicco*, ellipsoid or narrowly so, 27–28 by 11–12 mm, with an excentric apicule (obvious only in immature fruit); stipes 17–19 by 1.5–2 mm; fruiting receptacle broadly ovoid, 4–5 mm diam.; monocarps and stipes sparsely covered with golden appressed hairs <0.1mm long or glabrous, receptacle often sparsely covered with white-yellow appressed hairs 0.3–0.5 mm long. *Seeds* ellipsoid to narrowly so, ca. 17 by 7 mm, dark brown and wrinkled, raphe sunken, encircling seed diagonally.

#### Distribution.

Colombia (Antioquia, northern and western foothills of the Cordillera Occidental; one specimen potentially representing the species from Riseralda [*Betancur* & *al. 33011*]).

#### Habitat and ecology.

Forest, at elevations of 1200–1500 m. Flowering: May and December; fruiting: May, July and December.

#### Notes.

*Cremastospermadolichocarpum* can be distinguished from other species of *Cremastosperma* by the unique long-ellipsoid monocarps after which the species is named and identified even when sterile by the conspicuous axillary buds with dense indument.

#### Preliminary conservation status.

Most of the collections of *Cremastospermadolichocarpum* are from two national parks (Parque Nacional Las Orquídeas and Parque Nacional Paramillo), but overall they represent a small number of populations within a restricted area. Endangered [EN] (Table 1).

#### Selected specimens examined.

**COLOMBIA. Antioquia**: Parque Nacional Las Orquídeas, 6°32'N, 76°14'W, 1450 m a.s.l., 13 Aug 1993, *Cogollo et al. 6345* (MO); Parque Nacional Paramillo, Río San Jorge, 7°15'N, 75°55'W, 1560 m a.s.l., 3 Mar 1993, *Gentry et al. 79039* (U); Urrao, 6°29'N, 76°14'W, 1300 m a.s.l., 11 Dec 1992, *Pipoly et al. 16951* (MO).

### 12. Cremastosperma dolichopodum

Taxon classificationPlantaeMagnolialesAnnonaceae

Pirie & Maas sp. nov.

urn:lsid:ipni.org:names:60477509-2

Fig. 20 , Map 2

#### Diagnosis.

Differs from most species of the genus in the greater length of the pedicels (ca. 170 mm in flower; 250 mm in fruit). Compared to *C.longipes* (), differs by the habit (shrub ca. 3m tall, compared with a tree >4.5 m tall), smaller leaves (up to 35 cm long, compared with 35–60 cm long) and lack of indument on all parts.

#### Type.

PERU, Pasco: Oxapampa, Distr. Palcazú, Communidad Nativa Alto Lagarto – Villa Progreso. Reserva Comunal Yanesha, 500 m a.s.l., 9 Feb 2009, *Rojas, R. 6486* (holotype: USM; isotypes: HOXA, L! [L4318001], MO! [MO-2967424]).

#### Description.

*Shrub* ca. 3 m tall; young twigs and petioles sparsely covered with appressed hairs 0.1–0.2 mm long when very young, soon completely glabrous. *Leaves*: petioles 5–12 by 2–3 mm; lamina narrowly oblong-elliptic to oblong-elliptic, 22–38 by 11–16 cm (index 2–3), coriaceous, greyish above, brownish below, upper side glabrous, lower side glabrous except for some scattered hairs along primary nerve, base obtuse, apex acuminate (acumen ca. 10 mm long), extreme tip obtuse, primary vein raised over all of its length, 2–3 mm wide at widest point, glabrous, secondary veins 10–14, intersecondary veins absent, distance between from 8–10 mm at the base to 40 mm in the centre to 10 mm closer to the apex, angles with primary vein from 50–55° at the base to 45–50° closer to the apex, not branching, forming distinct loops, smallest distance between loops and margin 1–4 mm, tertiary veins percurrent. *Inflorescence* of single flowers, on older branches or potentially cauliflorous; combined short axillary shoot and pedicel 170–260 by 0.5–1 mm at the base, 2 mm at the apex (in flower), 250–260 mm by 0.5–1 mm at the base, ca. 2 mm at the apex (in fruit), short axillary shoot and pedicels glabrous; 1 lower bract, broadly triangular, ca. 1.5 by 2 mm, obtuse, persistent, glabrous but margins densely covered with brown hairs ca. 1 mm long; upper bract attached at ca. 1/3 from base, broadly triangular, ca. 1.5 by 2 mm, obtuse, glabrous, ciliate; closed flower buds not seen; flowers yellow tinged red *in vivo*, dark brown *in sicco*, glabrous; sepals free, depressed ovate, patent to reflexed, ca. 2 by 3.5 mm, obtuse, persistent, glabrous but margins covered with hairs ca. 0.1 mm long; petals grooved at base, outer petals narrowly elliptic to narrowly obovate, 25–30 by 7–10 mm, inner petals narrowly elliptic to narrowly obovate, 7–10 by 3–4 mm, glabrous; receptacle broadly oblongoid; intact androecium not observed, stamens 2–2.5 mm long, connective appendage ca. 1× 0.2 mm wide; gynoecium ca. 4 mm diam., carpels ca. 25, ca. 1 mm long, glabrous. *Monocarps* ca. 3, ellipsoid, obtuse, symmetrical, 20–22 by 15 mm, no obvious apicule, distally green to proximally reddish *in vivo*, blackish *in sicco*; stipes 19–21 by 1 to 2 mm subtending monocarp; fruiting receptacle oblongoid, ca. 5 mm diam., glabrous; monocarps, stipes and receptacle glabrous. *Seeds* not seen.

#### Distribution.

Peru (Pasco).

#### Habitat and ecology.

Primary forest remnants. At an elevation of ca. 500 m. Flowering and fruiting: February.

#### Notes.

*Cremastospermadolichopodum* is only known from two specimens collected at the same locality, but the length of the pedicel – a generally reliable character within the genus – greatly exceeds that of any similar known species.

#### Etymology.

The epithet “dolichopodum”, from the Greek dolicho (long) and podum (-stalked) refers to the unusual length of the pedicel.

#### Preliminary conservation status.

Of the three known collections of *Cremastospermadolichopodum*, two are from the same locality, within a protected area, the third from a roadside remnant of primary forest within the same region. Given the extent of collections of other species in the region (with regular documentation of the much earlier described and more widely distributed *C.oblongum* and *C.pendulum*) *C.dolichopodum* must be naturally rare as well of restricted distribution. Endangered [EN] (Table 1).

#### Other specimens examined.

**PERU. Pasco**: Pozuzo, Alto Lagarto to Pozuzo Alto Victoria road, 10°07'09"S, 75°29'25"W, 1500 m a.s.l., 29 June 2008, *Rojas* & *Ortíz 5758* (HOXA, MO, USM); Palcazú, Communidad Nativa Alto Lagarto, 10°08'00"S, 75°22'06"W, 500 m a.s.l., 9 Sep 2009, *Rojas* & *Ortíz 6485* (HOXA, L, MO, USM).

**Figure 20.**
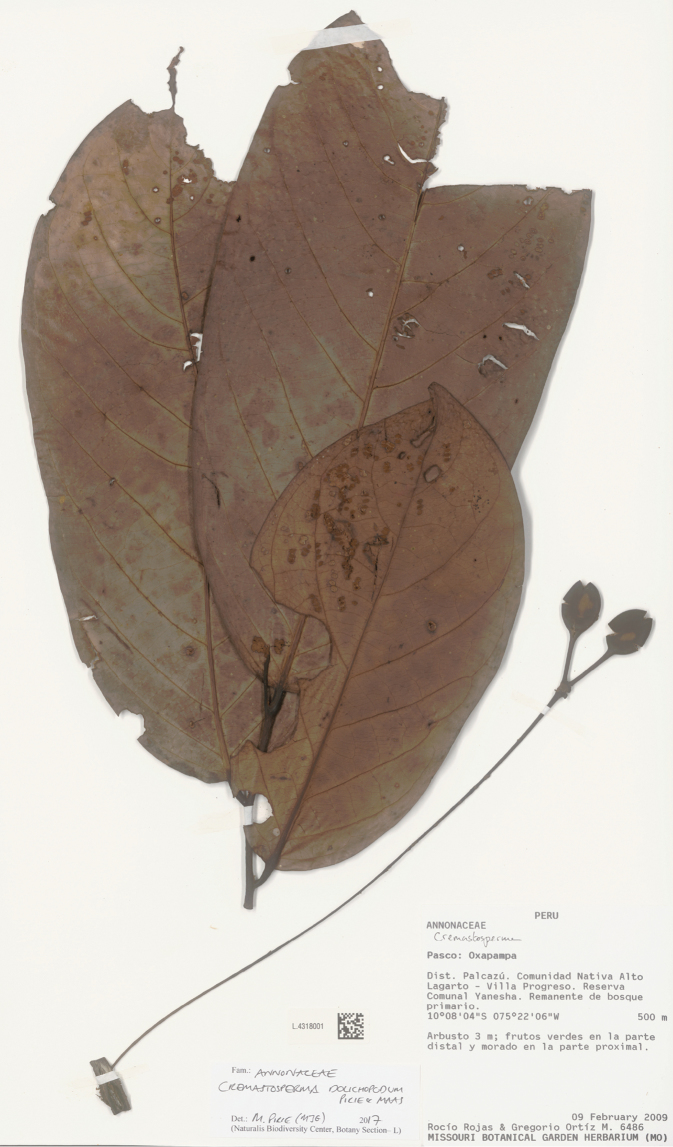
*Cremastospermadolichopodum* Pirie & Maas. Fruiting specimen (*Rojas & Ortíz 6486*).

### 13. Cremastosperma gracilipes

Taxon classificationPlantaeMagnolialesAnnonaceae

R.E.Fr.

**Figure 21.**
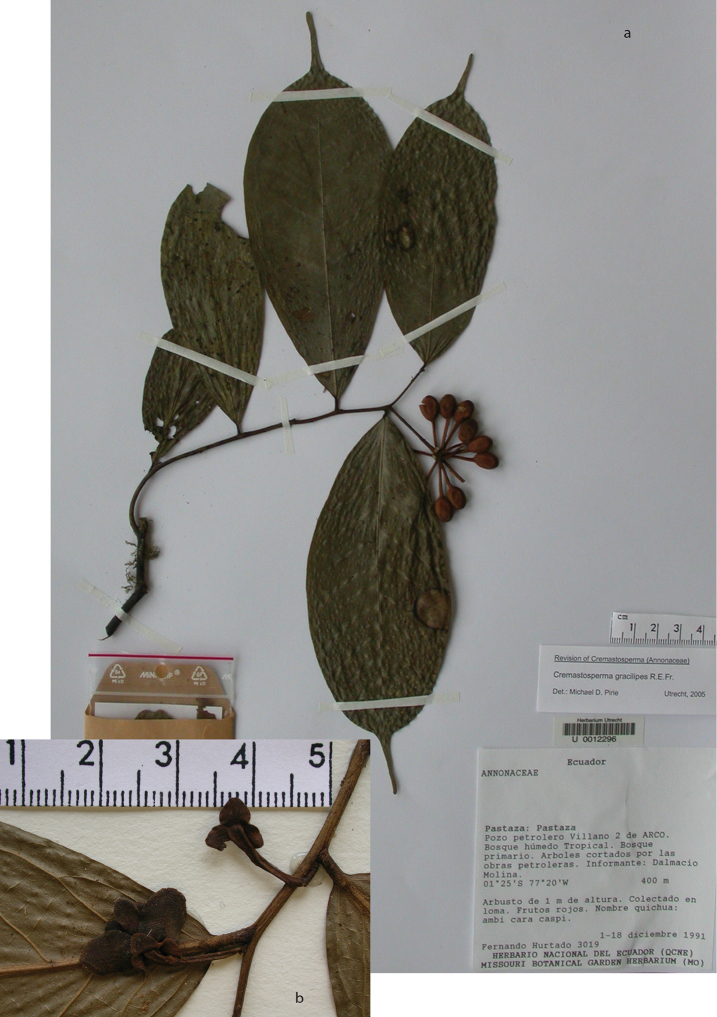
*Cremastospermagracilipes* R.E.Fr. **a** fruiting specimen **b** flower (**a***Hurtado 3019***b***Palacios 1651*).

Figs 1d , 21 , Map 4

Cremastosperma gracilipes R.E.Fr. Acta Horti Bergiani 10: 325. t. 26.

#### Type.

PERU, Ost-Peru, Regenwald von Ost-Peru: Stromgebiet des Marañon von Iquitos aufwärtsbis zur Santiago Mündung am Pongo de Manseriche, oberer Marañon; unterhalb des Pongo de Manseriche, flutfreier Hochwald, 155 m a.s.l., 13 Dec 1924, *Tessmann, G. 4748* (holotype: B! [B 10 0242370]; isotype: S! [S-R-6960]).

#### Description.

*Tree* or *shrub* 0.5–10 m tall; young twigs and petioles glabrous to sparsely covered with appressed brown hairs to 0.4 mm long. *Leaves*: petioles 2–8 by 1–2.5 mm; lamina elliptic to obovate or narrowly so, 11–28 by 3–10 cm (index 2–4(–4.7)), chartaceous, (pale) greyish- or brownish-green on both sides, often more greyish above, glabrous on both sides, base acute to obtuse or rounded, apex caudate (cusp 10–35 mm long), primary vein lightly grooved for basal third, 1–1.5 mm wide at widest point, more or less verrucose below, secondary veins 8–17, often 1–3 intersecondary veins, distance between from ca. 5 mm at the base to up to 25(-30) mm closer to the apex, angles with primary vein rather variable, from 45–80° at the base to 60–80° closer to the apex, forming distinct loops, smallest distance between loops and margin 2–6.5 mm, tertiary veins percurrent. *Inflorescence* of single, solitary flowers, on leafy twigs; peduncles 1–4 by ca. 1 mm (in flower), 2–5 by 1.5–2 mm (in fruit); pedicels (12–)15–25 by 1 mm at the base (in flower), 14–30 by 1–1.5 mm (in fruit), peduncles and pedicels rather densely covered with more or less erect brown hairs 0.2 mm long; 2 lower bracts, deltate, ca. 1 mm long, soon falling off, rather densely covered with more or less erect brown hairs 0.2 mm long; upper bract attached around midway along the pedicel, ovate or broadly so, 1–3 by ca. 1 mm, obtuse or acute, outer side sparsely to rather densely covered with appressed or erect whitish hairs to 0.2 mm long; closed flower buds depressed ovoid, opening loosely in development; flowers green to greenish-yellow, pale yellow or cream *in vivo*, dark brown with a lighter brown calyx *in sicco*, outer sides of sepals and petals sparsely to rather densely covered with erect or appressed whitish hairs to 0.2 mm long, inner sides glabrous to sparsely covered with appressed whitish hairs to 0.2 mm long (or inner petals papillate); sepals free, broadly ovate to deltate, recurved, 3–4 by 2.5–4 mm, obtuse, soon falling off; outer petals (broadly) elliptic to ovate, 9–15 by 7–12 mm, inner petals elliptic, obovate or narrowly so, 8–16 by 4–7 mm; androecium ca. 5 mm diam., stamens 1.2–1.5 mm long, connective appendage 0.7–0.8 mm wide; gynoecium ca. 2 mm diam., carpels ca. 25, ca. 2.2 mm long, sparsely covered with erect golden hairs 0.1 mm long. *Monocarps* 3–23, ellipsoid, slightly asymmetrical, 10–15 by 7–9 mm, with an excentric apicule, green maturing to pink or yellow through to red, purple and black *in vivo*, reddish or dark brown *in sicco*; stipes green maturing to pink or yellow to red *in vivo*, 7–17 by 1–1.5 mm, increasing to 3 mm diam. when mature; fruiting receptacle 3–8 mm diam.; monocarps, stipes and receptacle sparsely to rather densely covered with erect whitish hairs 0.1 mm long. *Seeds* ellipsoid, orange-brown, shallowly pitted, 5–8 by 3.5–6 mm, raphe sunken, regular.

#### Distribution.

Amazonian Colombia (Amazonas, Caquetá, Putamayo), Ecuador (Napo, Pastaza) and Peru (Loreto).

#### Habitat and ecology.

Primary forest, but also secondary, inundated and non-inundated forest. At elevations of 100–500 m. Flowering: January and April – August; fruiting: throughout the year.

#### Vernacular names.

Ecuador: Ambi cara caspi (*Hurtado 3019*); Ansuelo caspi muyo (*Lawesson et al. 39560*), Ayacara (*Whitmore 871*), Daycabome (Huaorani; *M. Aulestia et al. 1726*), Muncapatamo (Huaorani; *Espinoza et al. 578*).

#### Notes.

*Cremastospermagracilipes* most closely resembles *C.microcarpum*. The hairs on the flowers are shorter and less dense, which results in their drying a darker brown. The leaves are further generally distinctive in the shape of the apex (markedly caudate with an often long drip-tip) and in the green colour they consistently retain on drying. However, none of these characteristics is easy to define objectively or usefully and, although the geographic distributions of the two species are somewhat different (with *C.microcarpum* extending further into lowland Amazonia), there is apparent overlap. The species are closely related and further data to test their boundaries and the potential for gene-flow between species/populations is warranted. The leaves of *C.longicuspe* are similar to those of *C.gracilipes*, but in contrast to *C.gracilipes*, both flowers and fruit are entirely glabrous.

#### Preliminary conservation status.

*Cremastospermagracilipes* is one of the more widespread and abundant species of the genus, including occurrences in protected areas in Colombia and Ecuador, as well as in Peru. Least concern [LC] (Table 1).

#### Selected Specimens Examined.

**COLOMBIA. Amazonas**: Parque Nacional Amacayacu, 3°45'S, 70°15'W, 100 m a.s.l., 9 Aug 1989, *Vásquez et al. 12675* (U). **ECUADOR. Napo**: Yasuní Forest Reserve, 0°40'S, 76°28'W, 240–310 m a.s.l., 27 Jun 1995, *Acevedo-Rodríguez 7543* (US); Añangu, Río Napo, 0°30'S, 76°25'W, 300 m a.s.l., 9 Apr 1982, *Balslev 2418* (QCA); Comuna San José de Payamino, 0°30'S, 77°18'W, 300 m a.s.l., 1 Dec 1983, *Balslev et al. 4634* (AAU, NY); Punto Aguarico, 0°05'N, 76°59'W, 300 m a.s.l., 6 Apr 1980, *Brandbyge et al. 30472* (AAU, MO, NY, U, WU); San Pablo de Los Secoyas, 0°15'S, 76°21'W, 300 m a.s.l., 7 Aug 1980, *Brandbyge et al. 32589* (AAU, U); Reserva Biológica Jatun Sacha, 1°04'S, 77°36'W, 450 m a.s.l., 17 Jan 1987, *Cerón 702* (MO, U); El Chuncho Floristic Reserve, 0°30'S, 77°01'W, 250 m a.s.l., 1 Oct 1987, *Cerón et al. 2321* (MO); El Coca-Los Sachas Road, 0°25'S, 76°55'W, 250 m a.s.l., 8 Oct 1987, *Cerón* & *Neill 2428* (MO, U); Parque Nacional Sumaco, 0°24'S, 77°23'W, 540 m a.s.l., 6 Nov 1996, *Dik 1732* (MO); La Joya de los Sachas, 0°25'S, 76°37'W, 250 m a.s.l., 14 Sep 1992, *Gudiño* & *Grefa 1787* (MO); San José de Payamino, 40 km W of Coca, 0°30'S, 77°20'W, 300–600 m a.s.l., 23 Apr 1984, *Irvine 817* (F); Parque Nacional Yasuní, Añangu, 0°31'S, 76°23'W, 270 m a.s.l., 18 Feb 1983, *Luna et al. 9057* (NY, U); Reserva Etnica Huaorani, 0°39'45"S, 76°40'00"W, 300 m a.s.l., *Naranjo* & *B. Freire 493* (MO); Via Loreto, 4 km W of Río Payamino, 1°28'S, 77°02'W, 250 m a.s.l., 3 Aug 1986, *Neill et al. 7195* (MO); Río Tiputini, 0°43'S, 76°57'W, 300 m a.s.l., 21 Jul 1991, *Ollgaard et al. 99056* (AAU); Parque Nacional Yasuní, 0°26'S, 76°35'W, 250 m a.s.l., 7 Jun 1994, *Pitman 195* (U); Comunidad Kichwa El Eden, 0°31'35"S, 76°05'40"W, 200 m a.s.l., *D. Reyes* & *Carillo 691* (MO); Estación Científica Yasuní, 0°38'S, 76°30'W, 200–300 m a.s.l., 27 Aug 1995, *Romoleroux* & *Grefa 1834* (U); Sector Huashito, 0°20'S, 77°05'W, 250 m a.s.l., 3 Nov 1989, *Rubio 320* (MO); Río Pacuno, Bimbino, 0°40'S, 77°20'W, 300 m a.s.l., 19 Oct 1960, *Whitmore 737* (U); INIAP-Payamino Experimental Station, 0°26'S, 77°01'W, 250 m a.s.l., 3 Sep 1986, *Zaruma 581* (AAU, MO, NY, U). **Pastaza**: Pandanuque, S of oil well Villano 2 de ARCO, 1°28'S, 77°27'W, 550 m a.s.l., 30 Aug 1997, *Alvarez et al. 2405* (MO); Pozo petrolero ‘Namoyacu’ de UNOCAL, 1°40'S, 76°57'W, 290 m a.s.l., 13 Nov 1990, *Espinoza et al. 578* (MO); Pozo petrolero ‘Masaramu’ de UNOCAL, 0°44'S, 76°52'W, 400 m a.s.l., 1 May 1990, *Gudiño 310* (MO); Pozo petrolero Villano 2 de ARCO, 1°25'S, 77°20'W, 400 m a.s.l., 1 Dec 1991, *F. Hurtado 3019* (U); Lorocachi, 1°38'S, 75°58'W, 200 m a.s.l., 24 May 1980, *J. Jaramillo et al. 30773* (AAU, U); Río Acaro or Challuayacu, 1°23'S, 77°25'W, 360 m a.s.l., 19 Jan 1998, *Neill et al. 11095* (MO); Río Pastaza, 2°20'S, 76°55'W, 285 m a.s.l., 24 Jul 1980, *Ollgaard et al. 35223* (AAU); Río Curaray, 1°30'S, 76°32'W, 230 m a.s.l., 3 Sep 1985, *Palacios* & *Neill 783* (MO, U). **PERU. Loreto**: Oleoducto Secundario road betw. Bartra 1 & 4, 2°30'S, 75°45'W, 200 m a.s.l., 15 Sep 1979, *C. Díaz* & *N. Jaramillo 1411* (U); Prov. Maynas, NE of Comun Serafin Filomeno, 4°08'S, 72°55'W, 120–130 m a.s.l., 7 May 1991, *Grández et al. 2488* (MO); Distr. Nauta, “20 Enero”, 4°39'12"S, 73°49'18"W, 150 m a.s.l., 22 Jun 2006, *Huamantupa* & *N. Smith 7746* (MO, WAG); Río Ampiyacu, Pebas and vicinity, 3°10'S, 71°49'W, 4 May 1977, *Plowman et al. 7248* (GH); Prov. Requena, Distr. Tapiche, Quebrada Yanayacu, 6°15'49"S, 73°54'31"W, 15 Oct 2014, *Ríos et al. 4422* (F); Andoas, 2°55'S, 76°25'W, 180 m a.s.l., 9 Sep 1983, *Vásquez 4423* (MO); Yanamono Explorama Reserve, 3°30'S, 73°05'W, 108 m a.s.l., 25 Oct 1989, *Vásquez* & *N. Jaramillo 13007* (MO, U); Allpahuayo-IIAP, 4°10'S, 73°30'W, 150–180 m a.s.l., 11 Jul 1991, *Vásquez et al. 17376* (MO).

### 14. Cremastosperma leiophyllum

Taxon classificationPlantaeMagnolialesAnnonaceae

R.E.Fr.

**Figure 22.**
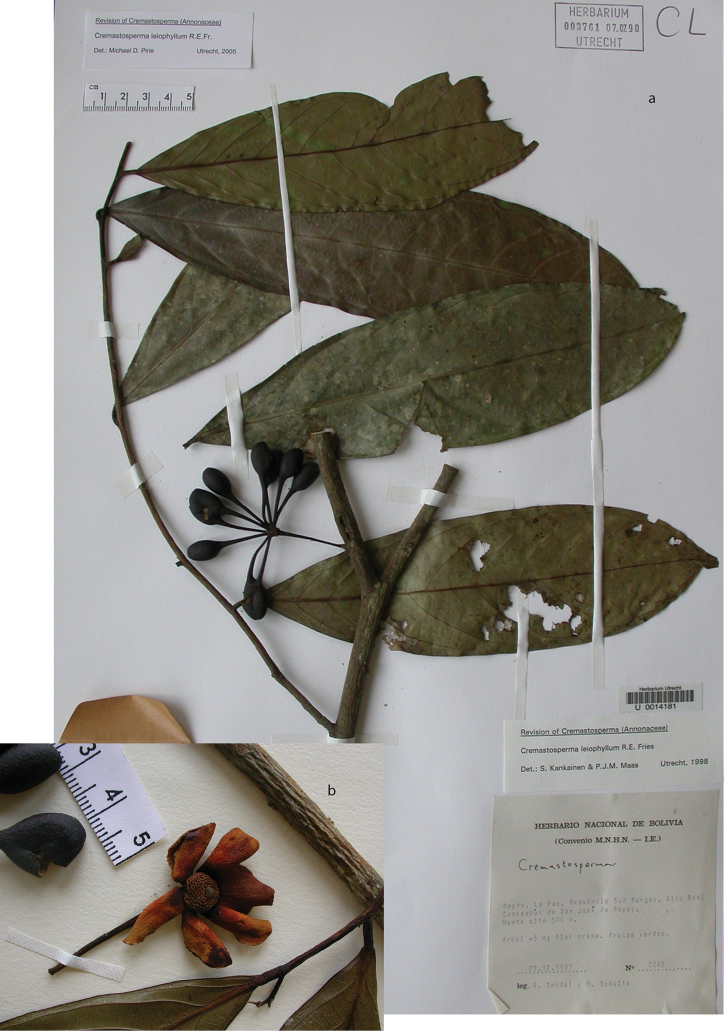
*Cremastospermaleiophyllum* R.E.Fr. **a** fruiting specimen **b** flower (**a, b***Seidel & Schulte 2265*)

**Figure 23.**
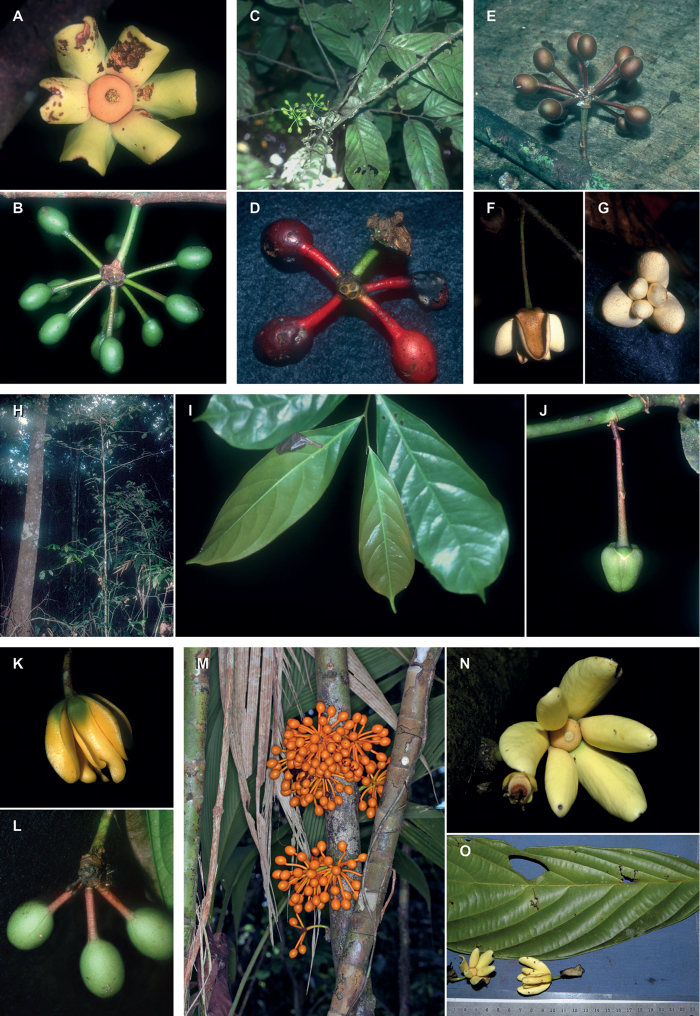
**a, b***Cremastospermaleiophyllum* R.E.Fr. **a** flower **b** fruit (*Pirie et al. 2*; photo LWC) **c, d***Cremastospermamegalophyllum* R.E.Fr. **c** habit (Maas et al. 8577, photo: PJMM), fruit (*Maas et al. 8595*, photo PJMM) **e–g***C.microcarpum* R.E.Fr. **e** fruit (*Maas et al. 6281*; photo PJMM) **f, g** flower (*Maas et al 8222*; photo PJMM) **h–j***C.monospermum* (Rusby) R.E.Fr. **h** habit **i** leaves **j** flower bud (*Pirie et al. 5*; photos LWC) **k, l***C.oblongum* R.E.Fr. **k** flower **l** fruit (*Maas et al. 9148*; photos PJMM) **m–o***C.osicola*. **m** fruit **n** flower **o** leaf and flower with scale bar (photos Reinaldo Aguilar).

Figs 22 , 23 , Map 6

Cremastosperma leiophyllum R.E.Fr., Acta Horti Bergiani 10: 328. 1931.
Guatteria leiophylla Diels, Annonaceae Novae. Notizbl. Bot. Gart. Berlin-Dahlem 11: 77. 1931. Non (Donn. Smith) Saff.

#### Type.

BOLIVIA, La Paz: Mapiri, San Carlos, 850 m a.s.l., 2 Dec 1926, *Buchtien, O. 705* (holotype: B! [B 10 0242369]; isotypes: HBG, MO! [MO-0477531], S! [S-R-7017], US).

*Annonanitida* Ruiz & Pav., Anales Instit. Bot. Cavanilles 17: 429, t. 488. 1959. Non Martius (1841), nom. nud.

*Guatteriarusbyi* J.F.Macbr., Publ. Field Columbian Mus., Bot Ser. 4: 171. 1929.

*Guatterialucida* Rusby, Mem. New York Bot. Gard. 7: 245. 1927. Non C. Presl.

#### Type.

BOLIVIA, Beni: Covendo 630 m a.s.l., 26 Aug 1921. *White, O.E. 913* (holotype: NY! [NY00026024]).

#### Description.

*Shrub* or *tree* 3–20 m tall, 3–18 cm diam.; young twigs and petioles glabrous. *Leaves*: petioles 4–12 by 1–3(–4) mm; lamina obovate to elliptic or narrowly so, 12–28 by 4–9(–12) cm (index 2–3.9), chartaceous, often green or greenish-brown above and below, more greyish above with darker or reddish veins, glabrous on both sides, base acute to obtuse, apex acuminate (acumen 5–15 mm long), primary vein 1.5–3 mm wide at widest point, verrucose, secondary veins 7–13, intersecondary veins 1–6, distance between from 14–24 mm at the base to 10–25 mm closer to the apex, angles with primary vein from 60–80° at the base to 40–50° closer to the apex, not branching forming distinct loops, smallest distance between loops and margin 2–4(–6) mm, tertiary veins percurrent. *Inflorescence* of single flowers, solitary or clustered in groups of up to 4, on older, leafless twigs; peduncles 1–2 by 1–2 mm (in flower), 2–4 by 1.5–3 mm (in fruit), sparsely covered with appressed golden <0.1 mm long hairs or glabrous; pedicels 18–34 by 1–1.5 mm at the base (in flower), 18–34(–43) by 1–3 mm (in fruit), glabrous; 1–3 lower bracts, depressed ovate, ca. 0.5 by 1 mm, obtuse, soon falling off, glabrous; upper bract attached around midway along pedicel, ovate to broadly so, ca. 1.5 by 1 mm, obtuse, glabrous; closed flower buds depressed ovoid, opening early in development; flowers green maturing to yellow or creamy yellow *in vivo*, dark yellow, reddish-brown or dark brown *in sicco*; sepals free, very broadly ovate-triangular, recurved, 2–3 by 2–3 mm, obtuse, soon falling off, sepals and petals glabrous; outer petals elliptic, 12–15 by 8–11 mm, rounded, inner petals elliptic, 13–15 by 6–8 mm; androecium ca. 7 mm diam., pinkish *in vivo*, stamens 1.4–1.8 mm long, connective appendage ca. 0.8 mm wide; gynoecium ca. 2 mm diam., carpels 2–2.2 mm long, glabrous. *Monocarps*, stipes and receptacle glabrous, monocarps 6–30, ellipsoid, asymmetrical, 14–17 by 8–9 mm, green maturing to yellow, orange-red and red *in vivo*, black (reddish-brown when immature) *in sicco*, with an excentric apicule; stipes 16–26 by 1–1.5 mm; fruiting receptacle 4–9 mm diam. *Seeds* ellipsoid, light brown, pitted ca. 12 by 7 mm, raphe sunken, regular.

#### Distribution.

Bolivia (Beni, Cochabamba, La Paz, Santa Cruz).

#### Habitat and ecology.

Mostly in primary wet or moist forest, also in mildly disturbed areas, often on slopes or terraces, on sandstone soils. At elevations of 200–1000 m. Flowering: February, May, July through September, November and December; fruiting: February through August, November and December.

#### Vernacular names.

Chocolatillo (*Meneces & Terceros 395*), Chocolatillo Negro (*D.N. Smith et al. 14006, 14058*), Eye (*Youras; Naessany 106*), Quií-quií (*Hinojosa & Seidel 1313*).

#### Notes.

*Cremastospermaleiophyllum* is the most southerly distributed species of the genus and. of the two found in Bolivia, the only endemic. Bud development in *C.leiophyllum* is open (as opposed to that of *C.monospermum*). It most closely resembles *C. spec B* (which is not found in Bolivia), from which it can best be distinguished by the characteristic shape (asymmetrical, the stipes thickening somewhat where they meet the monocarps) and colour (blackish) of the mature fruits when dried.

#### Preliminary conservation status.

Although a regional endemic, *Cremastospermaleiophyllum* has been collected regularly over a faily broad area of Bolivia including two national parks. Least concern [LC] (Table 1).

#### Selected specimens examined.

**BOLIVIA. Beni**: Serrania del Pilón Lajas, Yucumo, 15°15'S, 67°00'W, 850–900 m a.s.l., 3 May 1991, *Killeen et al. 3080* (USZ); Serranía del Pilón Lajas, Yucumo, 15°13'S, 67°03'W, 760–870 m a.s.l., 19 May 1989, *D.N. Smith et al. 13253* (LPB, MO, U, USZ). **Cochabamba**: Puerto Aurora, Región del Chapare, 16°50'S, 65°10'W, 28 Jun 1989, *Naessany 106* (LPB, U); Río Blanco, 17°12'S, 64°25'W, 240 m a.s.l., 31 May 2000, *Seidel 3543* (LPB). **La Paz**: San Carlos, Sarampiuni, 13°00'S, 65°00'W, 500 m a.s.l., 12 Mar 1927, *Buchtien 706* (B, S); Río Quiquibey, 15°29'S, 67°04'W, 1000 m a.s.l., 11 Nov 1990, *M.A. Lewis 37979* (MO, U); Alto Beni, Santa Ana, 15°37'S, 67°25'W, 500 m a.s.l., 28 Jun 1990, *Seidel et al. 2878* (LPB, U); Consata, 15°20'S, 68°31'W, 1300 m a.s.l., 15 Dec 1981, *Solomon et al. 6606* (LPB, MO, U). **Pando**: Campamento Gomero Pingo de Oro, 11°31'36"S, 69°06'11"W, 21 Oct 1999, *Paniagua 2119* (F). **Santa Cruz**: Velasco, N of Puerto Frey, 14°39'50"S, 61°09'33"W, 210 m a.s.l., 22 Sep 1995, *P.F. Foster 230* (WAG); Ayacucho Forest Reserve, 17°00'S, 63°00'W, 240 m a.s.l., 14 Apr 1976, *Meneces* & *Terceros 395* (MO, NY); Choré Forest Reserve, Río Ibabo, 16°35'S, 64°31'W, 180 m a.s.l., 16 Aug 1990, *Neill* & *Quevedo 9334* (LPB, MO, U, USZ); Parque Nacional Amboró, 17°33'S, 63°44'W, 360 m a.s.l., 15 Nov 1991, *I.G. Vargas et al. 1113* (F, LPB, NY, USZ).

### 15. Cremastosperma longicuspe

Taxon classificationPlantaeMagnolialesAnnonaceae

R.E.Fr.

Fig. 24 , Map 5

Cremastosperma longicuspe R.E.Fr., Acta Horti Bergiani 12: 203. 1934.

#### Type.

PERU, Loreto: Maynas, anno 1831. *Poeppig, E.F. s.n.* (lectotype (designated in Pirie, Kankainen & Maas, 2005): LE; isotype: S! [S-R-6962]).

*Cremastospermakillipii* R.E.Fr., Kongl. Svenska Vetenskapsakad. Handl. 24: 3, pl. 1 a-b. 1948.

#### Type.

PERU, Loreto: Yurimaguas, lower Río Huallaga, 135 m a.s.l., Aug-Sep 1929, *Killip, E.P. & Smith, A.C 29020* (holotype: US! [US00104264], isotype: S! [S-R-6961]).

#### Description.

*Tree* or *shrub* 1.5–20 m tall; young twigs and petioles sparsely covered with appressed whitish or golden hairs to 0.2 mm long. *Leaves*: petioles 4–14 by 1–3 mm; lamina elliptic, obovate or narrowly so, 10–27 by 3–11 cm (index 1.5–5), chartaceous, green or greyish-green above, green or brownish-green below, glabrous above, very sparsely covered with appressed whitish hairs to 0.2 mm long particularly on veins below, base acute, rarely obtuse, apex caudate (cusp 20–35 mm long), primary vein 1–2 mm wide at widest point, secondary veins 7–15, intersecondary veins 0–1(–4), distance between from 7–15 mm at the base to 9–18 mm closer to the apex, angles with primary vein from 60–80° at the base to 40–50° closer to the apex, rarely branching, forming distinct loops, smallest distance between loops and margin 2–4 mm, tertiary veins more or less percurrent. *Inflorescence* of single flowers solitary (or clustered in groups of 2), on leafy twigs; peduncles 2–5 by ca. 1 mm (in flower), 4–10 by 1–2 mm (in fruit), sparsely to rather densely covered with appressed golden hairs to 0.2 mm long; pedicels 10–14 by 1–1.5 mm at the base (in flower), 11–20 by 1–2 mm (in fruit), sparsely covered with appressed golden hairs to 0.2 mm long or glabrous; 2 lower bracts of unequal dimensions, basal lower bract deltate, ca. 0.5 by 0.5 mm, acute, soon falling off, apical lower bract narrowly elliptic, ca. 1.5 by 0.5 mm, rounded, soon falling off, lower bracts sparsely covered with appressed golden hairs to 0.1 mm long or glabrous; upper bract attached near base or midway along pedicel, ovate, ca. 2 by ca. 1 mm, acute, sparsely covered with appressed golden hairs to 0.1 mm long or glabrous; closed flower buds not seen, buds opening loosely in development; flowers yellowish *in vivo*, brown *in sicco*; sepals free, deltate, appressed or recurved, 3–4 by 3–4 mm, acute, soon falling off, sepals and petals glabrous; outer petals broadly elliptic, 10–12 by 9–12 mm, inner petals broadly elliptic, ca. 11 by 10 mm; androecium not seen; gynoecium not seen. *Monocarps* 6–13(–36), ellipsoid, slightly asymmetrical, 12–13 by 8–10 mm, white, red, deep red or deep purple *in vivo*, reddish-brown to dark brown or black *in sicco*, with an excentric apicule when unripe; stipes 10–19 by 2 mm; fruiting receptacle 4–5(–9) mm diam.; monocarps, stipes and receptacle glabrous. *Seeds* ellipsoid, orange or reddish-brown, 8–9 by 5–6 mm, raphe sunken, regular.

#### Distribution.

Ecuador (Napo); Peru (Loreto, San Martín), most collections found in the basin of the Río Huallaga.

#### Habitat and ecology.

Primary tropical wet forest, on sandy or white sand soil. At elevations of 140–200 m. Flowering: August-October; fruiting: February-June, August, September and November.

#### Vernacular names.

Ecuador: Moncapatahue (Huaorani; *M. Aulestia & Gonti 2072*). Peru: Anonilla (*J. Ruíz et al. 1748*).

#### Notes.

*Cremastospermalongicuspe* most closely resembles *C.gracilipes*, particularly in the shape of the leaf. However, in contrast to *C.gracilipes*, the fruits and flowers are glabrous. From the limited material available, the flower buds appear to be loosely, rather than widely open. In describing *C.killipii*, Fries (1948) noted its similarity to *C.longicuspe*. The leaves of the type specimen of *C.killipii* are unusually broad, but leaf and other characters otherwise fall within the variation found in *C.longicuspe*, including the notable caudate apex.

#### Preliminary conservation status.

*Cremastospermalongicuspe* is known from a scattering of collections across a reasonably wide area; none within protected areas and with no new collections of the species since September 2000. Given the low AOO and ongoing decline in habitats in the region, it is assigned: Vulnerable [VU] (Table 1).

#### Selected Specimens Examined.

**ECUADOR. Napo**: Reserva Etnica Huaorani, 0°55'S, 76°09'W, 250 m a.s.l., 20 Mar 1994, *Aulestia* & *Gonti 2072* (QCNE, U). **PERU. Loreto**: Río Huasaga, 3°20'S, 76°20'W, 185 m a.s.l., *Lewis et al. 11166* (USM); Puranchim, Río Sinchiyacu, 2°50'S, 76°55'W, 200 m a.s.l., 21 Nov 1986, *Lewis 12186* (MO, USM); Andoas, 2°55'S, 76°25'W, 180 m a.s.l., 3 Nov 1983, *Vásquez* & *N. Jaramillo 4559* (MO, U). **San Martín**: Santa Rosa de Davidcillo, Tarapoto-Yurimaguas road, 6°16'S, 76°17'W, 200 m a.s.l., 22 Apr 1986, *Knapp* & *Mallet 7178* (F, U, USM); Shapaja-Chazuta road, Río Huallaga, 6°36'S, 76°10'W, 250–300 m a.s.l., 4 Aug 1986, *Knapp 7864* (F, MO, NY, U, USM); Tarapoto-Yurimaguas Road, km 46, 6°24'S, 76°18'W, 350 m a.s.l., 30 Aug 1986, *Knapp* & *Mallet 8203* (USM).

**Figure 24.**
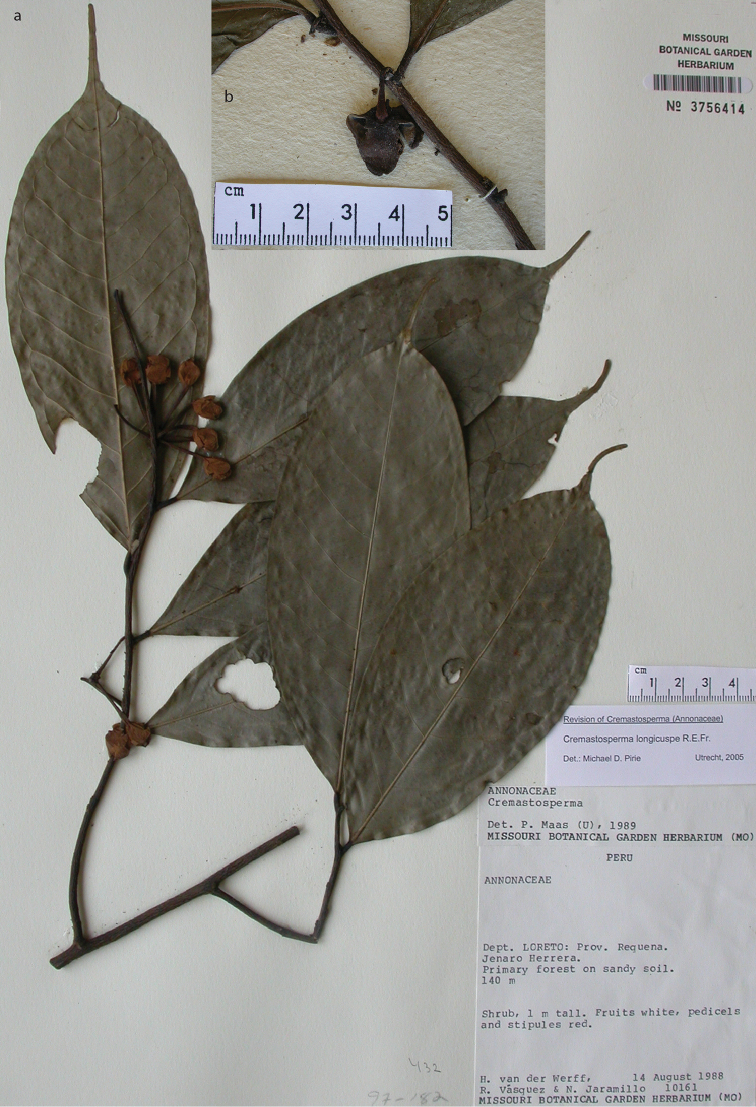
*Cremastospermalongicuspe* R.E.Fr. **a** fruiting specimen **b** flower (**a***van der Werff 10161***b***Poeppig s.n.*).

### 16. Cremastosperma longipes

Taxon classificationPlantaeMagnolialesAnnonaceae

Pirie

Fig. 25 , Map 2

Cremastosperma longipes Pirie, Blumea 50: 51, f. 5. 2005.

#### Type.

COLOMBIA, Chocó: San José del Palmar, mouth of Río Torito (tributary of Río Hábita), west slope, 3 Mar 1980, *Forero, E. et al. 6576* (holotype: COL! [COL000334460]; isotype: MO! [MO-047638]).

#### Description.

*Tree* 4.5–15 m tall; young twigs and petioles black, verrucose, sparsely to rather densely covered with white-golden appressed hairs ca. 0.4 mm long. *Leaves*: petioles 10–15 mm long, 2.5–7 mm diam.; lamina narrowly elliptic to elliptic, 35–60 by 10–25 cm (leaf index 2.3–3), chartacous to subcoriacous, olive/dark brown above, lighter below, glabrous above, sparsely covered with white-golden appressed hairs ca. 0.4 mm long on veins below (densely so developing leaves), base acute, apex acuminate (acumen 10–15 mm long), primary vein deeply grooved in basal half, 2–6 mm wide at widest point; secondary veins 10–16, intersecondary veins rare, distance between from 10 mm at the base to 80 mm closer to the apex, angles with primary vein 45° at the base to 60–70° closer to the apex, not branching, forming distinct loops in the apical half to third of the leaf, smallest distance between loops and margin 3–4 mm; tertiary veins mainly percurrent. *Inflorescences* of single, pendulous flowers, produced from leafless branches; peduncles 5–8 by ca. 1 mm (in flower), ca. 4 by 2 mm (in fruit); pedicels 90 (less mature) – 210 by 1 mm at the base, 1.5 at the apex (in flower), ca. 240 by 2 mm at the base, 3 mm at the apex (in fruit); peduncles and pedicels sparsely to rather densely covered with white-golden appressed hairs ca. 0.4 mm long; single lower bract, broadly elliptic, 1–2 by ca. 1 mm, acute, soon falling off, densely covered with white-golden appressed hairs ca. 0.4 mm long; upper bract attached on lower half of pedicel, elliptic, 1.5–3 by ca. 1 mm, acute, densely covered with white-golden appressed hairs ca. 0.4 mm long; closed flower buds not seen; flowers green (immature) *in vivo*, medium brown *in sicco*; sepals free, triangular to broadly trullate, appressed, 3–4.5 mm long, acute, soon falling off, rather densely to densely covered with white-golden appressed hairs ca. 0.4 mm long; outer petals elliptic, ca. 22 by 12 mm, inner petals narrowly elliptic, ca. 22 by 6 mm, sparsely to rather densely covered with white-golden appressed hairs ca. 0.4 mm long; stamens ca. 1.2 mm long, connective appendage ca. 1 mm wide. *Monocarps* ca. 20, black *in sicco*, ellipsoid, slightly asymmetrical, ca. 20 by 12 mm, without an apicule; stipes ca. 25 by 2 mm; fruiting receptacle ovoid, 8 mm diam.; monocarps and stipes glabrous, receptacle sparsely covered with white-golden appressed hairs ca. 0.4 mm long. *Seeds* ellipsoid, 18–20 by 8–9 mm, orange/brown, with many shallow pits, raphe slightly raised, encircling seed longitudinally.

#### Distribution.

Pacific coast of Colombia (Chocó, Riseralda) and Ecuador (Esmeraldas).

#### Habitat and ecology.

Humid lowland to premontane forest. At elevations of 280–1400 m. Flowering: January and March; fruiting: September.

#### Notes.

*Cremastospermalongipes* can easily be distinguished from most other species of the genus by the exceptional length of the pedicel, after which the species is named. The flowers and fruits of most species of *Cremastosperma* are borne on pedicels less than 50 mm long, with rare exceptions such as *C.pedunculatum* and *C.bullatum* never exceeding 150 mm in length, significantly shorter than those of *C.longipes*. The only species with pedicels of a comparable length is the newly described *C.dolichopodum*, which differs from *C.longipes* in the lack of indument on the flowers and receptacle. In addition, leaves of *C.longipes* are unusually large, equalling the maximum dimensions observed in *C.megalophyllum*, a more densely collected species from Amazonian Colombia, Ecuador and Peru.

#### Preliminary conservation status.

*Cremastospermalongipes* is only known from three collections from widely spaced localities outside of protected areas. Vulnerable [VU] (Table 1).

#### Other specimens examined.

**COLOMBIA. Riseralda**: Geguadas-Puerto de Oro road, 800–1400 m a.s.l., 13 Sep 1991, *J.L. Fernández et al. 8872* (COL). **ECUADOR. Esmeraldas**: Fila de Bilsa, 0°37'N, 79°51'W, 280 m a.s.l., 30 Jan 1991, *Gentry et al. 72995* (F, MO).

**Figure 25.**
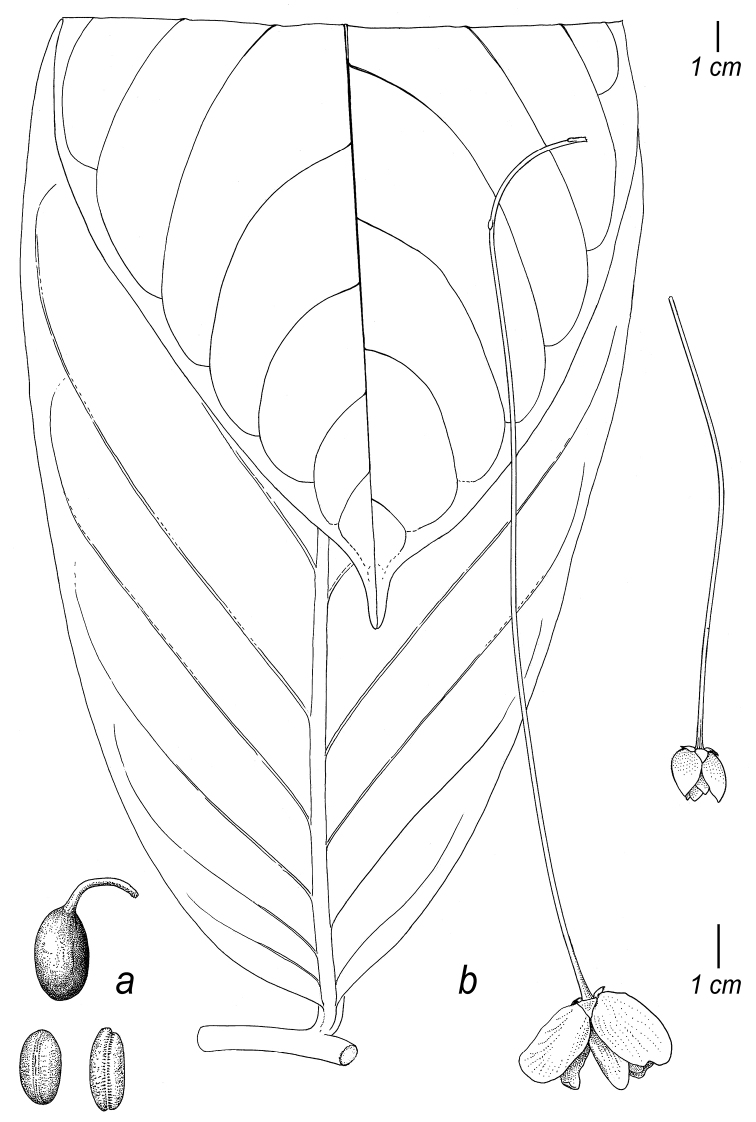
*Cremastospermalongipes* Pirie. **a** monocarp (with stipe) and seeds **b** leaf, flower and flower bud (**a***Fernández 8872***b***Forero et al. 6576*).

### 17. Cremastosperma macrocarpum

Taxon classificationPlantaeMagnolialesAnnonaceae

Maas

**Figure 26.**
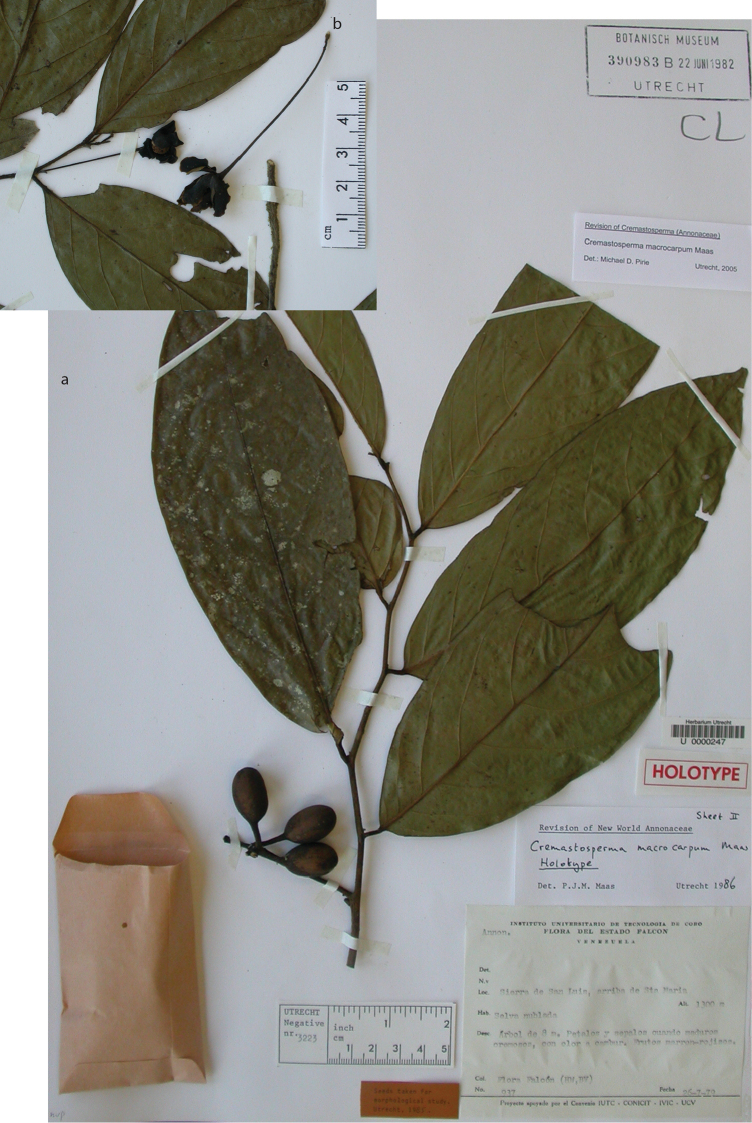
*Cremastospermamacrocarpum* Maas. **a** fruiting specimen **b** flower (**a, b***van der Werff & Vera, Flora Falcón 937*).

Figs 2d , 26 , Map 9

Cremastosperma macrocarpum Maas, Proc. Kon. Ned. Akad. Wetensch. Ser. C 89: 253, f. 2, 3b. 1986.

#### Type.

VENEZUELA, Falcón: Sierra de San Luis, above Santa María, 1300 m, 26 Jul 1979, *van der Werff, H.H. & Vera, B., Flora Falcón 937* (holotype: U! [U0000246]).

#### Description.

*Tree* 5–10 m tall; young twigs and petioles glabrous. *Leaves*: petioles 3–10 by 1–3 mm; lamina narrowly elliptic to elliptic, 10–30 by 4–9(12) cm (index 2.3–3.7), chartaceous, green, brownish-green or brown on both sides, darker above, glabrous on both sides, base obtuse to rounded, apex acuminate (acumen 5–10 mm long), primary vein grooved in basal half, 1–2 mm wide at widest point, verrucose, secondary veins 6–12, intersecondary veins 1–2, distance between from 7–15 mm at the base to 7–23 mm closer to the apex, angles with primary vein from 60–80° at the base to 45–60° closer to the apex, not branching, forming mostly distinct loops, smallest distance between loops and margin 1–5 mm, tertiary veins mostly percurrent. *Inflorescence* of single flowers, on leafy twigs; peduncles ca. 1 by 0.5–1 mm (in flower), 1–2 by 1–2 mm (in fruit), sparsely covered in appressed golden hairs to 0.1 mm long or glabrous; pedicels 35–45 by 0.5–1 mm at the base up to 2.5 mm at the apex (in flower), 40–65 by 1–1.5 mm at the base up to 5 mm at the apex (in fruit), glabrous; single lower bract, depressed ovate, ca. 0.5 by 1 mm, rounded, soon falling off, rather densely covered with appressed golden hairs to 0.1 mm long; upper bract at or near base of pedicel, ovate, ca. 1.5 by 0.8 mm, obtuse, glabrous; closed flower buds not seen; flowers pale greenish-yellow with green base or cream-coloured *in vivo*, black *in sicco*, sepals and petals glabrous; sepals free, broadly to depressed ovate, recurved, 1.5–2 by 1.5–2 mm, obtuse, soon falling off; outer petals ovate, 11–14 by 8–9 mm, obtuse, inner petals obovate, 15–16 by ca. 7 mm, obtuse; androecium ca. 6.5 mm diam., gynoecium not seen. *Monocarps* 7–18, ellipsoid, slightly asymmetrical, 18–24 by 12–14 mm, very small strongly excentric apicule, green maturing to yellowish-brown, brown or purple-black *in vivo*, reddish-brown or dark brown *in sicco*; stipes 7–14 by ca. 2 mm; fruiting receptacle 5–8 mm diam.; monocarps, stipes and receptacle glabrous. *Seeds* ellipsoid, orange-brown, shallowly pitted, ca. 20 by 8 mm, raphe sunken, regular.

#### Distribution.

Venezuela (Carabobo, Falcón, Yaracuy).

#### Habitat and ecology.

Primary or secondary evergreen cloud forest. At elevations of 700–1500 m. Flowering: July; fruiting: March, May-July, October and December.

#### Notes.

One of only two species of *Cremastosperma* found in Venezuela, *C.macrocarpum* can most easily be distinguished from *C.venezuelanum* by its smaller leaves (10–30 cm as opposed to 30–53 cm long) and longer pedicels (40–65 mm as opposed to 16–22 mm in fruit).

#### Preliminary conservation status.

*Cremastospermamacrocarpum* is known from seven collections representing five localities outside of protected areas, the most recent having been collected in 1991. Endangered [EN] (Table 1).

#### Selected specimens examined.

**VENEZUELA. Carabobo**: Río Morón valley, 10°17'N, 68°10'W, 700–1100 m a.s.l., 3 May 1991, *W. Diaz* & *Niño 274* (NY, U). **Falcón**: Cerro Galicia, 1500 m a.s.l., 10 Jun 1978, *T. Ruíz Z. et al. 3499* (U). **Yaracuy**: Río Carabobo, 10°26'N, 68°49'W, 800–1200 m a.s.l., 31 Mar 1980, *Liesner* & *A. González 9763* (MO, U); San Felipe, 10°15'N, 68°29'W, 1200 m a.s.l., 7 Dec 1980, *Steyermark 123804* (U).

### 18. Cremastosperma magdalenae

Taxon classificationPlantaeMagnolialesAnnonaceae

Pirie

Fig. 27 , Map 3

Cremastosperma magdalenae Pirie, Blumea 50: 53, f. 6. 2005.

#### Type.

COLOMBIA, Antioquia: San Luis, Medellín-Bogotá highway, 8.1 km E of bridge over Río Caldera, 13 Mar 1983, *Escobar, L.A. de & Folsom, J.P. 3309* (holotype: NY!, 2 sheets [NY00759135, NY00759134]; isotypes: HUA! [HUA0000058], U! [U0012249]).

#### Description.

*Tree* 3–7 m tall; young twigs glabrous. *Leaves*: petioles 6–14 mm long, 2–3 mm diam.; lamina narrowly elliptic to elliptic, 20–28 by 7–9 cm (index 2.5–3.1), chartaceous to subcoriaceous, olive to more lime green or brown above, darker below, glabrous on both sides, base obtuse to acute, apex acuminate (acumen 5–10 mm long), primary vein deeply grooved in basal 1/2 – 3/4, occasionally verrucose below, ca. 2 mm wide at widest point, glabrous, secondary veins 9–14, often 2 or 3 intersecondary veins, distance between from 4–5 mm at the base to 20–35 mm closer to the apex, angles with primary vein from 40–50° at the base to 70–80° closer to the apex, occasionally branching, occasionally forming more or less indistinct loops in the apical half, smallest distance between loops and margin 2–4 mm, tertiary veins rather reticulate. *Inflorescences* of single flowers, solitary or clustered in groups of two, axillary on leafy twigs; peduncles 2–3 by ca. 2 mm (in fruit); pedicels ca. 7 by 1 mm at the base, 1.5 mm at the apex (in flower), 16–20 by 1.5–2 mm at the base, ca. 3 mm at the apex (in fruit), peduncles and pedicels glabrous, two lower bracts, the apical one depressed triangular, ca. 1.5 by 2 mm, acute, persistent, glabrous, upper bract attached around midway along pedicel, broadly ovate to deltate, 0.5–2.5 by 1–2.5 mm, actute to obtuse, glabrous; closed flower buds not seen; flowers *in vivo* immature light green, black *in sicco*, sepals and petals glabrous; sepals fused for basal 1 mm, broadly to very broadly ovate, appressed, (2–) 5–7 by (2–)–5 mm, acute, mostly persistent, outer petals elliptic, ca. 12 by 7 mm, inner petals narrowly elliptic, ca. 12 by 5 m; androecium 2.5–2.7 mm diam., stamens ca. 0.7 mm long, connective appendage 0.3–0.4 mm wide. *Monocarps* 20–30, globose, symmetrical, 12–13 by 12–13 mm, green maturing to red *in vivo*, black *in sicco*, with a slightly excentric, 0.25 mm long apicule; stipes (immature) 9–10 by 1–1.5 mm; fruiting receptacle depressed ovoid, 7–9 mm diam. (only immature fruits seen); monocarps, stipes and receptacle glabrous. *Seeds* globose, shallowly pitted with a papery outer layer, ca. 13 by 11 mm, orange-brown, raphe neither raised nor sunken, regular.

#### Distribution.

Colombia (Antioquia, west side of the Magdalena valley).

#### Habitat and ecology.

Disturbed primary or secondary forest. At elevations of 670–1200 m. Flowering and fruiting: March.

#### Note.

*Cremastospermamagdalenae* can be distinguished from other species of the genus by the combination of globose monocarps and large sepals which mostly persist into fruiting (one slightly differing collection, *Cárdenas 2899*, displays immature fuits with smaller sepals only persistent on one of the two duplicates studied). Also noteworthy are the relatively short pedicels and the absence of indument on all parts. The absence of hairs on fruits and flowers reveals the blackish colour typical of specimens of *Cremastosperma* upon drying. Both *C.panamense* and *C.pacificum* (a species found on the Pacific coast of Colombia) also lack indument, but, amongst other differences, the sepals of both species are much smaller and do not persist into fruiting.

#### Preliminary conservation status.

*Cremastospermamagdalenae* is known from just four collections representing similar non-protected localities, three close to the highway between Medellín and Bogotá. Endangered [EN] (Table 1).

#### Other specimens examined

**. COLOMBIA. Antioquia**: Al Prodigio, 6°06'N, 74°48'W, 350 m a.s.l., 26 Jun 1990, *Cárdenas et al. 2899* (COL, MO); Mun. San Luis, Medellín-Bogotá road, Río Samaná, 6°00'N, 74°50'W, 670 m a.s.l., 19 Mar 1982, *Hernandez 251* (COL, HUA); Mun. San Luis, Medellín-Bogotá road, Río Caldera, 6°00'N, 74°50'W, 1000 m a.s.l., 13 Mar 1983, *Juncosa et al. 736* (MO).

**Figure 27.**
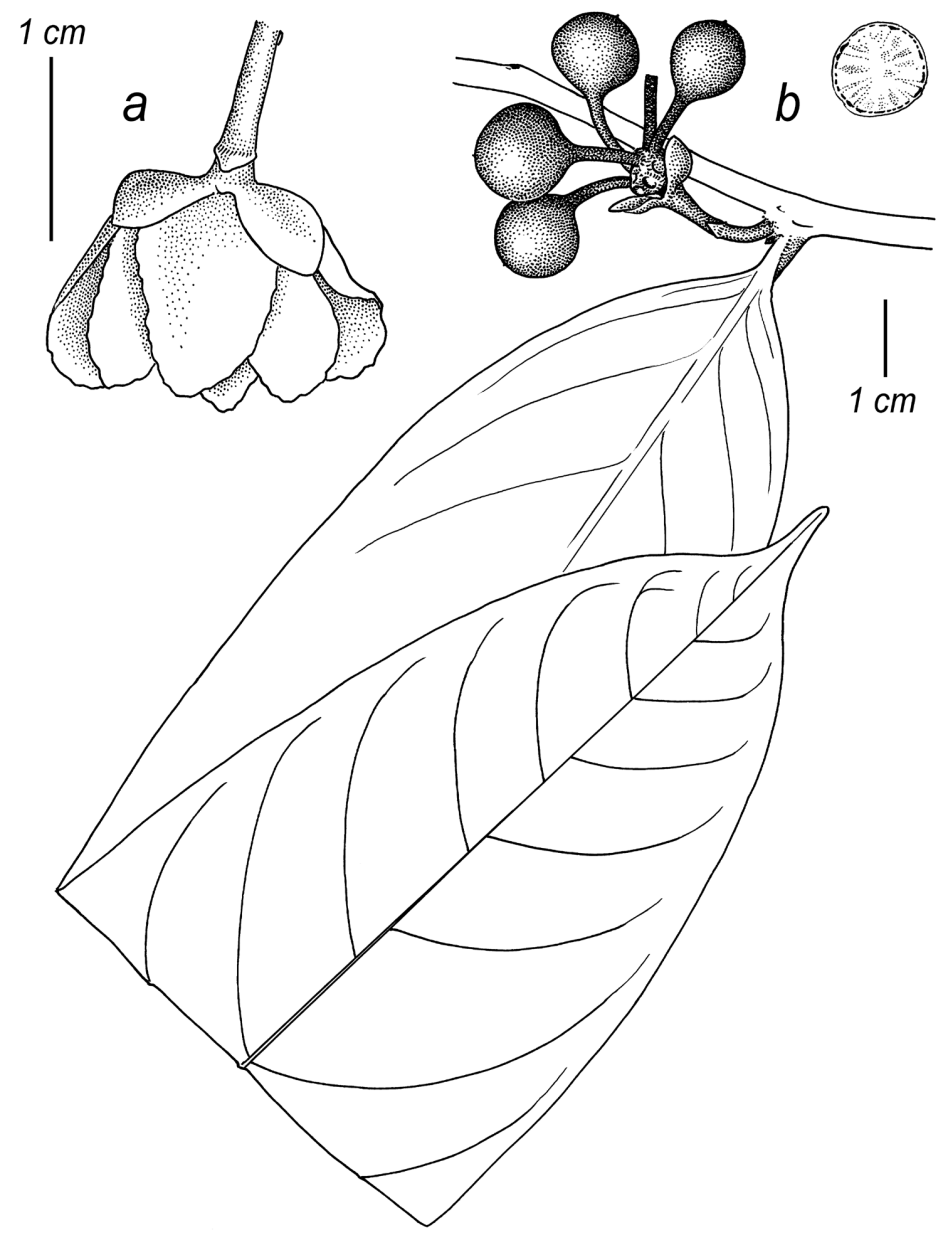
*Cremastospermamagdalenae* Pirie. **a** flower **b** fruiting twig and cross section of seed (**a***Hernandez 251***b***Escobar & Folsom 3309*).

### 19. Cremastosperma megalophyllum

Taxon classificationPlantaeMagnolialesAnnonaceae

R.E.Fr.

**Map 7.**
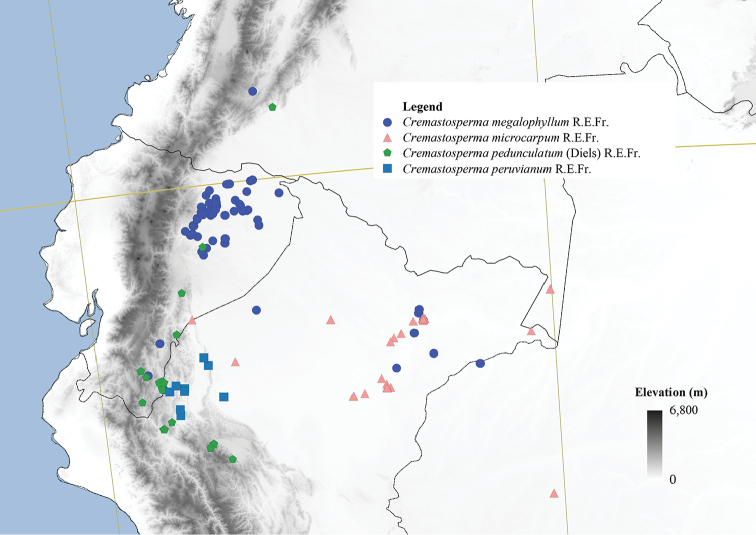
Distribution of *Cremastospermamegalophyllum* R.E.Fr.; *C.microcarpum* R.E.Fr.; *C.pedunculatum* (Diels) R.E.Fr.; and *C.peruvianum* R.E.Fr.

**Figure 28.**
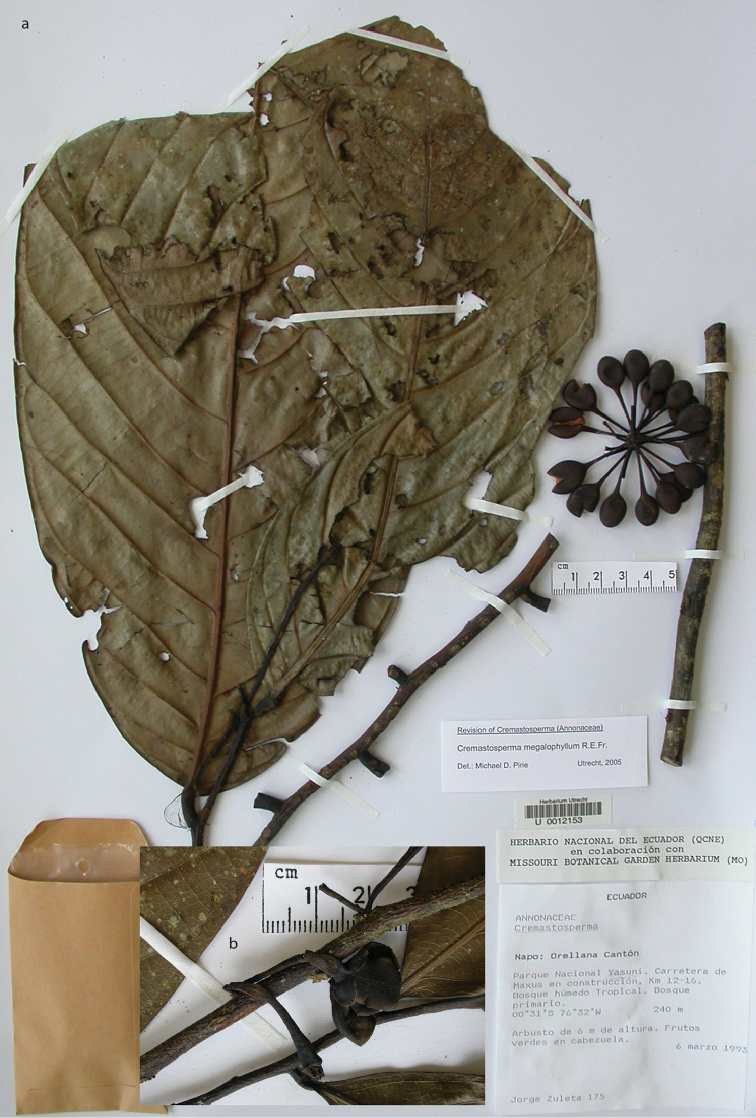
*Cremastospermamegalophyllum* R.E.Fr. **a** fruiting specimen **b** flower buds (**a***Zuleta 175***b***Palacios 3270*).

Figs 2a , 23 , 28 , Map 7

Cremastosperma megalophyllum R.E.Fr., Acta Horti Bergiani 10: 329. 1931.

#### Type.

PERU, Loreto: Boca de Pebas, 23 Oct 1927, *Ducke, A. RB19620* (holotype: S! [S-R-6963]; isotypes: B! [B 10 0242368], RB! [RB00534080, RB00534176]).

#### Description.

*Tree* or *shrub* 3–15 m tall, 2–15(–35) cm diam.; young twigs and petioles glabrous. *Leaves*: petioles 5–22 by 1.5–9 mm; lamina obovate to elliptic or narrowly so, 13–57 by 3–26 cm (index 2–5.1), chartaceous to coriaceous, shiny on both sides, secondary veins often impressed above (giving slightly bullate appearance), (dark) greyish-green or brown above, more brown or green below, glabrous on both sides, base obtuse to rounded, rarely acute or decurrent, apex acuminate (acumen (5-)10–30 mm long), primary vein conspicuously grooved in basal half, 1–5 mm wide at widest point, secondary veins (5–)8–21, intersecondary veins often 1–2(–3), distance between from 5–25 mm at the base to 9–35(–43) mm closer to the apex, angles with primary vein from (30–)45–70(–80)° at the base to (30–)40–75° closer to the apex, rarely branching, forming distinct loops, smallest distance between loops and margin 1–5 mm, tertiary veins percurrent. *Inflorescence* of single flowers, solitary or clustered in groups of up to 3, on leafy or leafless twigs or on the main trunk; short axillary shoot, 4–7(–8) by 0.7–1 mm (in flower), 4–10 by 1.5–4 mm (in fruit), glabrous or sparsely covered with whitish appressed hairs to 0.1 mm long; pedicels 10–20(–32) by ca. 1 mm at the base, up to 2(–2.5) mm at the apex (in flower), 15–30(–40) by 1.5–3 mm at the base, up to 4 mm at the apex (in fruit), glabrous; 3 lower bracts, deltate, ca. 1 by 1 mm, acute, soon falling off, sparsely covered with whitish appressed hairs to 0.1 mm long or glabrous; upper bract attached in apical half of pedicel, broadly ovate, 1–3 by 1–2 mm, obtuse, glabrous; closed flower buds depressed ovoid, opening loosely in development; flowers green maturing to yellow *in vivo*, black *in sicco*, sepals and petals glabrous; sepals free or connate for 1 mm, broadly ovate to triangular, appressed, open and conspicuous whilst petals still closed in young buds, 4–6 by 4–6 mm, obtuse to acute, sometimes briefly or partly persistent; outer petals broadly elliptic, 11–18 by 9–15 mm, obtuse, inner petals obovate, 10–16 by 5–7 mm, obtuse; androecium diam. unknown, stamens 1.3–1.8 mm long, connective appendage 0.7–1 mm wide; gynoecium diam. unknown, carpels 1.5–2 mm long. *Monocarps* 6–32, ellipsoid to broadly ellipsoid, (slightly) asymmetrical, 12–20 by 9–14 mm, green maturing to yellow, orange, purple and black *in vivo*, reddish or dark brown or black *in sicco*, often with an excentric apicule; stipes 10–30 by 1–2 mm; fruiting receptacle 3–12 mm diam.; monocarps, stipes and receptacle glabrous. *Seeds* broadly ellipsoid, reddish-orange, pitted, ca. 12 by 9–10 mm, raphe raised (more so when seeds immature), regular.

#### Distribution.

Amazonian Colombia (Caquetá, Putamayo), Ecuador (Morona-Santiago, Napo, Pastaza, Sucumbíos, Zamora-Chinchipe) and Peru (Amazonas, Loreto).

#### Habitat and ecology.

Primary and secondary premontane or lowland rainforest, sometimes inundated, on red (oxisols/lateritic) or sometimes volcanic soils. At elevations of 100–1200 m. Flowering: April-June, September-January; fruiting: throughout the year.

#### Vernacular names.

Ecuador: Ayacara (Quichua; *A. Alvarado 298*), Cucha casa caspi (*Hurtado 3005*), Caramoyu (*Whitmore 854*), Ichilla cara caspi (small bark tree; *D. Irvine 374*), Lynshtimoia (Quechna; *Whitmore 717*), Mandachi (Shuar; *W. Palacios 10278*), Oñetahue (the plant itself), Oñetahuemo (the fruit) (Huaorani; *J.S. Miller et al. 794*), Tara caspi (Quichua; *Grijalva & Grefa 305*) or T’zinytala (Quichua; *W. Palacios 10278*). Peru: Bara (*Rimachi Y. 1027*).

#### Notes.

Despite its name, the size of leaves of *Cremastospermamegalophyllum* varies from large to relatively small with respect to those of other species of the genus. This variation is also apparent in the size of the fruits, which are similar to those of *C.napoense*, but which in contrast are never borne on a branching inflorescence. *C.megalophyllum* is best distinguished by the shape of the glabrous, black-drying flowers: the large sepals open earlier and to a greater extent than the petals (in contrast to those of *C.napoense*, bud development of which is open from an early stage and the lighter colour (particularly of the pedicel) of which indicates the presence of indument).

#### Preliminary conservation status.

*Cremastospermamegalophyllum* is one of the more widespread and abundant species of the genus, including occurrences in protected areas in Ecuador. Least concern [LC] (Table 1).

#### Selected specimens examined.

**COLOMBIA. Caquetá**: Mocoa, 2°00'N, 76°00'W, 4 May 1899, *Sprague 351* (K). **ECUADOR. Morona-Santiago**: Misión Salesiana-Shuar, 3°25'S, 78°35'W, 800 m a.s.l., 8 Jun 1986, *Zaruma* & *Arguello 518* (MO, U). **Napo**: Yasuní Forest Reserve, 0°40'S, 76°22'W, 200 m a.s.l., 25 Jun 1995, *Acevedo-Rodríguez et al. 7523* (US); Hollín-Loreto road, foothills of Volcán Sumaco, 0°44'S, 77°28'W, 600 m a.s.l., 26 Jun 1989, *Alvarado 298* (AAU, GB, MO, NY, U); San Pablo de Los Secoyas, 0°15'S, 76°21'W, 300 m a.s.l., 6 Aug 1980, *Brandbyge et al. 32566* (AAU, U); Dureno, Comunidad Indigena Cofan, 0°02'S, 76°42'W, 350 m a.s.l., 22 Sep 1986, *Cerón 404* (AAU, MO, U); Reserva Biológica Jatun Sacha, 1°04'S, 77°36'W, 450 m a.s.l., 17 Jan 1987, *Cerón 612* (MO, QAME, U); Galeras Cordillera, E of Río Pusuno, 0°52'55"S, 77°34'15"W, 900–1200 m a.s.l., 19 Jun 2009, *Couvreur et al. 130* (L); La Joya de los Sachas, 0°25'S, 76°37'W, 220 m a.s.l., 23 Nov 1992, *A. Grijalva* & *Grefa 305* (U); Río Suno, W of Río Napo, 0°42'S, 77°10'W, 400 m a.s.l., 23 Jun 1968, *Holm-Nielsen et al. 903* (AAU); San José de Payamino, 40 km W of Coca, 0°30'S, 77°20'W, 300–600 m a.s.l., 3 Nov 1983, *Irvine 374* (F); Reserva Etnica Huaorani, 0°39'45"S, 76°40'00"W, 300 m a.s.l., *Naranjo* & *B. Freire 399* (MO); Cañón de los Monos, 15 km N of Coca, 0°20'S, 77°01'W, 250 m a.s.l., 5 Apr 1985, *Neill et al. 6359* (MO, QAME, U); Coca-Loreto road. 10 km W of Río Payamino, 0°35'S, 77°10'W, 275 m a.s.l., 10 Oct 1987, *Neill et al. 7883* (MO, U); INIAP-Payamino Experimental Station, 0°25'S, 77°10'W, 250 m a.s.l., 18 Nov 1991, *Neill et al. 10005* (MO); Coca-Loreto road, 8 km after Loreto, 0°35'S, 77°20'W, 800 m a.s.l., 8 Jun 1987, *Palacios 1620* (MO, U); Río Payamino, road to Loreto, 0°26'S, 77°06'W, 300 m a.s.l., 15 Dec 1987, *Palacios 2311* (MO, U); Puerto de Misahuallí, 1°04'S, 77°37'W, 450 m a.s.l., 7 Sep 1988, *Palacios 2937* (U); El Chuncho Floristic Reserve, 0°25'S, 77°01'W, 250 m a.s.l., 23 May 1993, *Palacios 10663* (U); Via Los Zorros, Sol Naciente, 0°40'S, 77°07'W, 250 m a.s.l., Aug 1993, *Palacios 11016* (U); Río Napo, 20 km W of Coca, 0°35'S, 77°03'W, 300 m a.s.l., 22 Apr 1985, *Stein et al. 2577* (AAU, MO, NY, U); Río Tutapishco, 0°36'S, 77°22'W, 480 m a.s.l., 27 Jan 1996, *H. Vargas* & *Cerda 671* (U); Parque Nacional Sumaco, 0°08'S, 77°08'W, 400 m a.s.l., 30 Sep 1996, *H. Vargas* & *Alvarado 1025* (MO); Estación Científica Yasuní, 0°38'S, 76°30'W, 200–300 m a.s.l., 24 Apr 2002, *Villa et al. 1499* (F, U); Ridge W of Hac. Cotapino, parallel to Río Cotapino, 0°40'S, 77°20'W, 360 m a.s.l., 18 Oct 1960, *Whitmore 717* (K); Payamino-Loreto road, 4–6 km from R Areas, 0°26'S, 77°02'W, 250 m a.s.l., 13 Sep 1986, *Zaruma 657* (MO); Parque Nacional Yasuní, 0°31'S, 76°32'W, 240 m a.s.l., 6 Mar 1993, *Zuleta 175* (U). **Pastaza**: Pozo petrolero ‘Masaramu’ de UNOCAL, 0°44'S, 76°52'W, 390 m a.s.l., 1 May 1990, *Espinoza 129* (MO, NY, U); Río Landayacu, 1°34'S, 77°25'W, 580 m a.s.l., 25 Nov 1990, *Gudiño 1123* (U); Curaray, NE of Destacamento, 1°21'S, 76°56'W, 250 m a.s.l., 19 Mar 1980, *Holm-Nielsen 22082* (AAU, U); Pozo petrolero Villano 2 de ARCO, 1°25'S, 77°20'W, 400 m a.s.l., 1 Dec 1991, *F. Hurtado 3005* (U); Río Tigüeno, 1°16'S, 77°11'W, 200 m a.s.l., 4 Jun 1995, *J.S. Miller et al. 794* (U); Pozo Petrolero Villano 2, 1°29'S, 77°27'W, 500 m a.s.l., 24 Jul 1992, *Palacios 10278* (U); Vía Auca, 115 km S of Coca, 6 km S of Río Tigüino, 1°15'S, 76°55'W, 320 m a.s.l., 31 Mar 1989, *Zak* & *Rubio 4202* (MO, U). **Sucumbíos**: Rio Aguarico, Zabalo village, 0°21'24"S, 75°39'56"W, 22 Nov 1998, *Aguinda et al. 458* (F); Campo Bermejo 6 Norte, 0°14'S, 77°13'W, 1050 m a.s.l., 23 Mar 1990, *Cerón et al. 9324* (U); Reserva Cuyabeno, 0°00'S, 76°12'W, 265 m a.s.l., 1 Apr 1988, *R. Valencia et al. 67352* (AAU, U); Dureno, 0°02'07"S, 76°45'17"W, 250 m a.s.l., 31 May 2007, *Vriesendorp 428* (L). **Zamora-Chinchipe**: Jamboe Bajo, 4°05'S, 78°55'W, 1100 m a.s.l., 3 Nov 1996, *Clark et al. 3158* (U); Cordillera del Condór, 4°26'14"S, 78°37'12"W, 900–1400 m a.s.l., 19 Jun 2005, *Quizhpe et al. 1505* (MO, U). **PERU. Amazonas**: Atalaia do Norte. Río Javari, 4°33'S, 71°40'W, 2 Jan 1989, *Cid Ferreira et al. 9959* (NY). **Loreto**: Río Marañón, San Rafael, 3°46'S, 73°03'W, 11 Sep 1989, *Daly et al. 6186* (MO, NY, U); Alto Amazonas, 2°55'S, 76°25'W, 210 m a.s.l., 6 Jun 1981, *Vásquez* & *N. Jaramillo 1969* (MO); Miraflores, Quebreda Tamshiyacu, 4°15'S, 72°40'W, 200 m a.s.l., 24 Feb 1986, *Vásquez* & *N. Jaramillo 7237* (MO, U); Yanamono Explorama Reserve, 3°30'S, 72°50'W, 106 m a.s.l., 28 Sep 1988, *Vásquez 11076* (F, U); Iquitos-Nauta road, 4°30'S, 73°30'W, 130 m a.s.l., 8 Nov 1988, *Vásquez et al. 11192* (MO, U); Las Amazonas, ExplorNapo Camp, 3°15'S, 72°54'W, 140 m a.s.l., 27 Jun 1991, *Vásquez et al. 16885* (MO, U).

### 20. Cremastosperma microcarpum

Taxon classificationPlantaeMagnolialesAnnonaceae

R.E.Fr.

**Figure 29.**
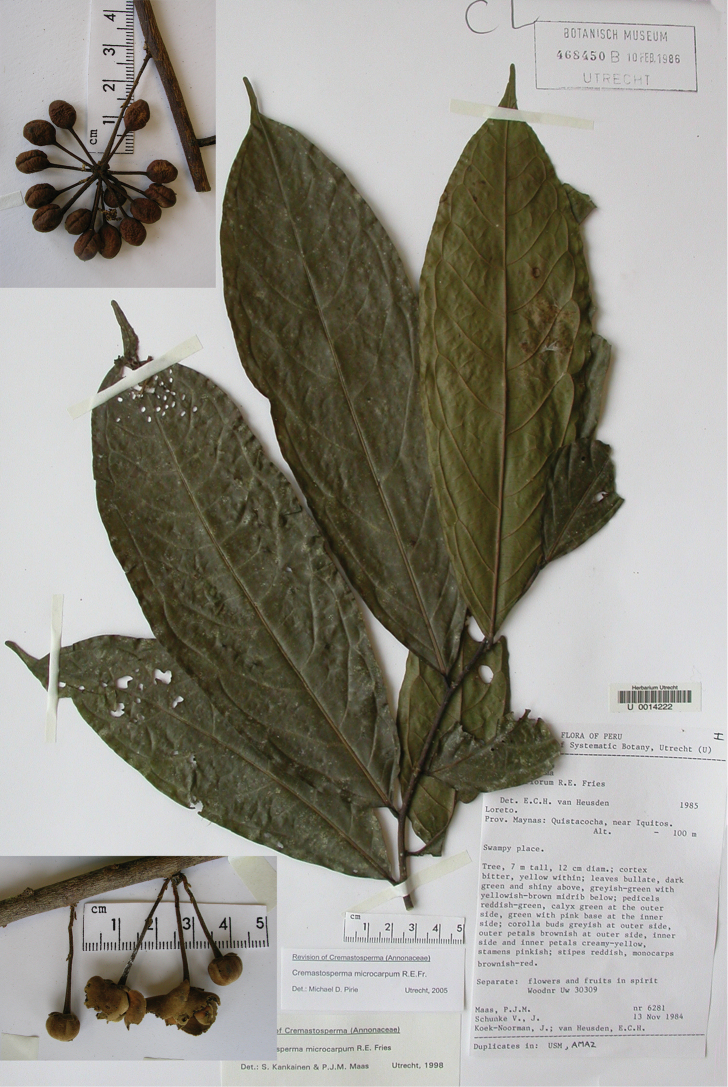
*Cremastospermamicrocarpum* R.E.Fr. **a** leaves **b** flowers and flower buds **c** fruit (**a, b***Maas et al. 6281***c***Vásquez & Jaramillo 9350*).

Figs 1a,g , 2c , 23 , 29 , Map 7

Cremastosperma microcarpum R.E.Fr., Acta Horti Bergiani 12: 559. 1939.

#### Type.

BRAZIL, Amazonas: Mun. Humaitá, Rio Madeira, near Tres Casas, 18 Sep 1934, *Krukoff, B.A. 6151* (holotype: S! [S-R-6964]; isotypes: A! [00039263], F! [V0054581F], G! [G00237254], GB! [GB-0047034], K! [K000485529], MICH! [MICH1286075], MO! [MO-047716], NY! [NY00025862], U! [U0000248], US! [US00811272, US00901600]).

#### Description.

*Tree* or *shrub* 2–20 m tall, 2.5–18.5 cm diam.; young twigs and petioles glabrous to rather densely covered with appressed and/or erect white or golden hairs 0.3–0.4 mm long. *Leaves*: petioles 2–12 by 1.5–3 mm; lamina narrowly elliptic to elliptic or narrowly obovate, 13–31 by 4–12 cm (index 1.8–3.8(–4.8)), chartaceous, green, greyish- or brownish-green or brown on both sides, shiny above, venation below often yellowish, glabrous above, glabrous to rather densely covered with appressed or erect white hairs to 0.2 mm long at the base and on primary and secondary veins below, base acute to obtuse, rarely rounded or narrowly cuneate, apex acuminate (acumen 10–40 mm long), primary vein 1–2 mm wide at widest point, more or less verrucose on both sides, lightly grooved for around half of length, secondary veins 7–15, often 1–4 intersecondary veins, distance between from ca. 5 mm at the base to ca. 20 mm closer to the apex, angles with primary vein mostly from 45–60° at the base to 60–80° closer to the apex, not branching, forming distinct loops, smallest distance between loops and margin 2–7 mm, tertiary veins percurrent. *Inflorescence* of single flowers, solitary or clustered in groups of up to 3, on leafy or leafless twigs; peduncles (3–)5–15 by ca. 1 mm (in flower), 4–15 by 1–2 mm (in fruit); pedicels (5–)12–24 by ca. 1 mm at the base (in flower), 10–25 by 1–2 mm (in fruit), peduncles and pedicels sparsely to rather densely covered with appressed or erect whitish hairs to 0.3 mm long; 1 to several lower bract(s), the basal-most small and scale-like, those more apical mostly (long) elliptic, occasionally leaf-like, 2–6(–60) by ca. 1 mm, acute, soon falling off, rather densely covered with appressed white hairs to 0.3 mm long; upper bract attached mostly on the basal half of the pedicel, ovate to deltate, 1.5–2.5 by 1–1.5 mm, obtuse or acute, sparsely to rather densely covered with appressed or erect golden hairs to 0.3 mm long; closed flower buds very broadly to depressed ovoid, opening loosely in development; flowers green, maturing to brown, (pale) yellow, cream or white outside, cream or yellow inside, sepals green or dark brown outside, green with a pink base inside *in vivo*, pale (orange-) brown or brown with dark or reddish-brown base *in sicco*, sepals and petals rather densely to densely covered with appressed or erect golden hairs (whitish close to the edges) to 0.3 mm long; sepals free or connate for 0.5 mm, broadly ovate to deltate, not reflexed, 3–4[-6] by 2.5–4[-6] mm, obtuse, soon falling off; outer petals ovate to very broadly ovate, rounded, 11–18[-19] by 10–17 mm, inner petals elliptic to (narrowly) obovate, obtuse, 10–16[-22] by 5–8[-10] mm; androecium ca. 7 mm diam., stamens 1.3–1.5 mm long, connective appendage 0.6–0.8 mm wide; gynoecium ca. 1 mm diam., carpels 2–2.5[-2.9] long, sparsely covered with erect whitish hairs to 0.1 mm long. *Monocarps* (8-)17–33, ellipsoid to broadly ellipsoid, asymmetrical, 8–11 by 6–8 mm, often with an oblique longitudinal groove corresponding to the seed raphe, green maturing to pink or orange through purple or brownish-red, brown and black *in vivo*, dark or reddish-brown *in sicco*, with an excentric apicule, monocarps, stipes and receptacle sparsely to rather densely covered with erect whitish hairs ca. 0.1 mm long; stipes 8–16 by 1 mm; fruiting receptacle 4–8 mm diam. *Seeds* broadly ellipsoid, orange brown, pitted, 6–8 by 5–6 mm, raphe sunken, somewhat irregular.

#### Distribution.

Amazonian Colombia (Amazonas), Ecuador (Morona-Santiago), Peru (Amazonas, Loreto) and Brazil (Amazonas).

#### Habitat and ecology.

Forest inundated by white (várzea) or black (tahuampa) water, on yellowish, lateritic soil. At elevations of 80–200 m. Flowering: March, July and September; fruiting: throughout the year.

#### Vernacular names.

Peru: Bara (*McDaniel et al. 17020*), Barra caspi (*McDaniel et al. 20677*), churú yais (*F. Dominguez 52, 59*), Hicojilla (*Schunke V. 6412*), Icoja (*M.E. Mathias et al. 5510*), Yais (Huambisa; *Huashikat 321, 493*), Zorro Caspi Blanco (*Freitas 8*).

#### Notes.

*Cremastospermamicrocarpum* resembles most closely *C.gracilipes*, from which it differs in the denser, longer hairs on the flowers and the generally acuminate as opposed to caudate leaf apex (but see discussion under that species). The hairy flower resembles somewhat those of *C.cauliflorum*, but which cannot be confused as *C.microcarpum* never exhibits a branching inflorescence. In addition, the monocarps of *C.cauliflorum* are larger than those of *C.microcarpum* and characteristically globose to transversely broadly ellipsoid as opposed to ellipsoid.

#### Preliminary conservation status.

*Cremastospermamicrocarpum* is one of the more widespread and abundant species of the genus, including occurrences in protected areas in Colombia. Least concern [LC] (Table 1).

#### Selected specimens examined.

**BRAZIL. Amazonas**: Rio Embira, 7°30'S, 70°15'W, 10 Jun 1933, *Krukoff 4748* (F, G, K, MICH, MO, NY, S, U). **COLOMBIA. Amazonas**: Parque Nacional Amacayacu, 3°01'S, 70°02'W, 100 m a.s.l., 21 Jun 1991, *Rudas et al. 2260* (MO). **ECUADOR. Morona-Santiago**: Santiago-Río Morona road, E of Santiago, 2°58'24"S, 77°49'36"W, 322 m a.s.l., 10 Jul 2004, *Croat 90749* (MO, U). **PERU. Amazonas**: Río Santiago valley, Quebrada Caterpiza, 4°00'S, 77°00'W, 200 m a.s.l., 3 Sep 1979, *Huashikat 321* (MO). **Loreto**: Yanamono Explorama Reserve, 3°27'S, 72°51'W, 100–150 m a.s.l., 30 Dec 1998, *Chatrou et al. 208* (MOL, U); Nauta-Parimari, 5°00'S, 74°15'W, 90 m a.s.l., 1993, *Del Carpio 2213* (MO); Jenaro Herrera, 4°55'S, 73°40'W, 125 m a.s.l., 26 Oct 1988, *Freitas 8* (U); Mishana, Río Nanay, 3°50'S, 73°30'W, 130 m a.s.l., 26 Feb 1979, *Gentry* & *Aronson 25111* (U, USM); Quistacocha, near Iquitos, 3°45'S, 73°20'W, 140 m a.s.l., 13 Mar 1981, *Gentry et al. 32153* (MO, NY, U, USM); Río Samiria, Flor de Yarina, 5°02'S, 74°30'W, 140–160 m a.s.l., 4 Aug 1982, *Gentry et al. 38085* (MO, U); Reserva Nacional Pacaya-Samiria, 3°18'S, 74°50'W, 130 m a.s.l., 22 Oct 1990, *Grández* & *N. Jaramillo 2032* (MO); Braga, near Caño Supay, Río Ucayali, 4°55'S, 73°44'W, 110 m a.s.l., 27 Oct 1994, *Maas et al. 8222* (L, U, USM, WU); Mishana, Río Nanay, Quebreda San Pedro, 3°55'S, 73°35'W, 130 m a.s.l., 26 Sep 1986, *Vásquez* & *N. Jaramillo 8010* (MO, U); Mariscal Castilla, Caballo cocha, 3°55'S, 70°30'W, 106 m a.s.l., 14 Jul 1987, *Vásquez* & *N. Jaramillo 9350* (F, MO, U, USM); Sapuena, Jenaro Herrera, 4°50'S, 73°45'W, 170 m a.s.l., 17 Nov 1987, *Vásquez* & *N. Jaramillo 10088* (MO, U, USM).

### 21. Cremastosperma monospermum

Taxon classificationPlantaeMagnolialesAnnonaceae

(Rusby) R.E.Fr.

**Figure 30.**
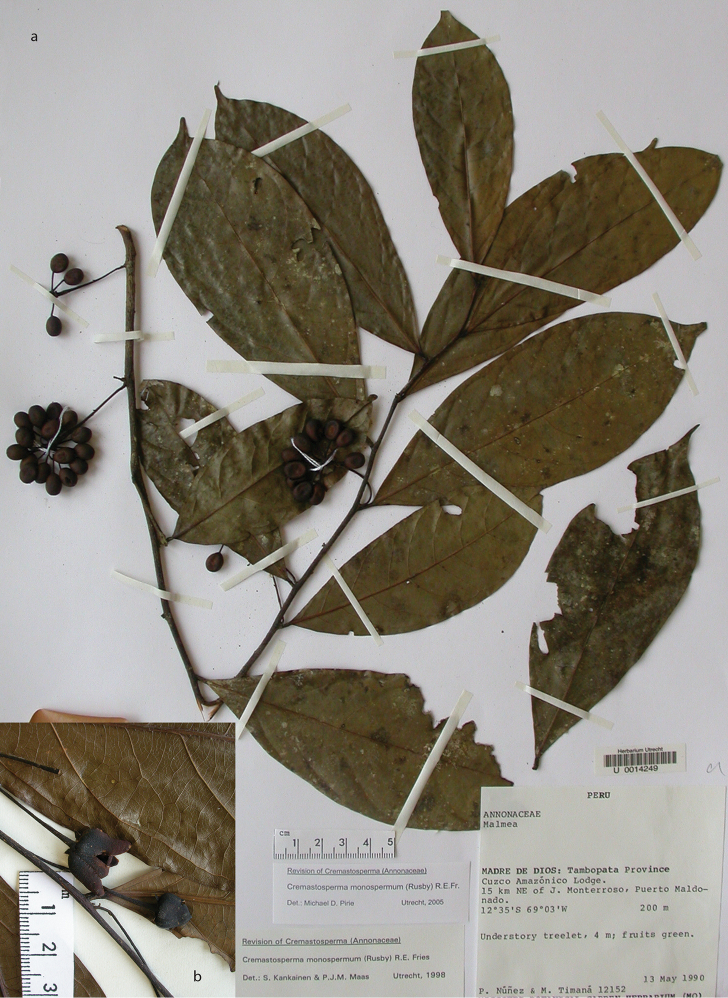
*Cremastospermamonospermum* (Rusby) R.E.Fr. **a** fruiting specimen **b** flower buds (**a***Nuñez & Timaná 12152***b***Cid Ferreira et al. 6301*).

Figs 1c , 23 , 30 , Map 4

Cremastosperma monospermum (Rusby) R.E.Fr., Acta Horti Bergiani 10: 193. 1931.
Cymbopetalum monospermum Rusby, Bull. New York Bot. Gard. 6: 505 (“Symbopetalum”). 1910.

#### Type.

BOLIVIA, La Paz: San Buenaventura, 470 m a.s.l., 12 Nov 1901, *Williams, R.S. 670* (holotype: NY [NY00025876]; isotype: K! [K000485528]).

*Cremastospermajuruense* R.E.Fr., Acta Horti Bergiani 12: 282. 1937.

#### Type.

BRAZIL, Amazonas: Basin of Rio Juruá; near mouth of Rio Embira (tributary of Rio Tarauacá), 6 Jun 1933, *Krukoff, B.A. 4697* (holotype: NY! [NY00025861]; isotype: S! [S-R-7933]).

Cremastospermamonospermum(Rusby)R.E.Fr.var.brachypodum R.E.Fr., Acta Horti Bergiani 12: 559. 1939.

#### Type.

BRAZIL, Pará: Boa Vista on the Tapajós River, 5 May 1929; *Dahlgren & Sella 162* (holotype: S! [S-R-7011]; isotypes: B! [B 10 0242367], F! [V0054582F]).

#### Description.

*Tree* or *shrub*, 1–12 m tall, 4–10 cm diam.; young twigs and petioles glabrous. *Leaves*: petioles 5–10 by 1–3 mm; lamina obovate, elliptic or narrowly so, (8-)10–35 by 4–12 cm (index 2–3.5), chartaceous, green to brown, darker above, veins often reddish below, glabrous on both sides or rarely sparsely covered with appressed whitish to 0.4 mm long hairs on primary vein below, base acute to obtuse, rarely narrowly cuneate, mostly decurrent, apex acuminate to caudate (acumen/cusp 5–30 mm long), primary vein 1–2 mm wide at widest point, secondary veins 6–10, intersecondary veins 0–3, distance between from 10–18 mm at the base to 12–24 mm closer to the apex, angles with primary vein from 70–80° at the base to 40–50° closer to the apex, rarely branching, forming mostly distinct loops, smallest distance between loops and margin 2–6 mm, tertiary veins mostly reticulate. *Inflorescence* of single flowers solitary (or clustered in groups of 2), on leafy or leafless twigs; peduncles 2–10 by 0.5–1 mm (in flower), 2–10 by 1.5–2 mm (in fruit), sparsely covered with appressed to erect golden to 0.1 mm long hairs or glabrous; pedicels 40–50(-70) by 0.5–1 mm at the base (in flower), (12-)22–73 by 1–1.5 mm (in fruit), green or reddish *in vivo*, glabrous; 2 lower bracts, deltate, ca. 0.8 by 0.8 mm (occasionally large and leafy), obtuse, soon falling off, sparsely covered with appressed golden 0.1 mm long hairs or glabrous; upper bract mostly attached around half way along pedicel, narrowly ovate or deltate, 1–2.5 by ca. 1 mm, acute, obtuse, rounded or truncate, outer side sparsely covered with appressed golden 0.1 mm long hairs or glabrous; closed flower buds broadly ovoid-triangular, remaining closed (or nearly so) throughout development; flowers green, maturing to creamy yellow, yellow or orange *in vivo*, dark or reddish-brown or black *in sicco*; sepals and petals glabrous, sepals free or connate for ca. 1 mm, broadly ovate or deltate, appressed, patent or recurved, 2–4 by 2–3 mm, acute or obtuse, mostly soon falling off; outer petals ovate, 9–14 by 6–8 mm, inner petals elliptic to ovate or narrowly so, 10–13 by 4–5 mm; androecium not seen; gynoecium not seen. *Monocarps* 10–29, ellipsoid to broadly so, slightly asymmetrical, 9–11 by 7–8 mm, green maturing to pink, maroon, red or (blue-) black *in vivo*, brown, dark or reddish-brown or black *in sicco*, with an excentric apicule; stipes (6-)8–15 by 1 mm; fruiting receptacle 3–8 mm diam., monocarps, stipes and receptacle glabrous. *Seeds* broadly ovoid, reddish-brown, pitted, pits appear black with raised rim, 8–10 by 6–7 mm, raphe sunken, regular.

#### Distribution.

Bolivia (Beni, La Paz, Pando), Brazil (Acre, Pará, Rondônia) and widespread across Peru.

#### Habitat and ecology.

Primary and secondary lowland forest, occasionally on poorly drained soils or brown latosols. At elevations of 200–500 m. Flowering: April, July – December; fruiting: throughout the year.

#### Vernacular names.

Bolivia: Yohisi (Chacobo; *Boom 5039*), huabu midha (Bourdy 1828). Peru: Ayacbara (Schunke V. 2554).

#### Notes.

*Cremastospermamonospermum* is the most widespread species of the genus – the only one found both along the eastern foothills of the Andes as far south as Bolivia and across Brazil south of the Amazon. It is best distinguished by the shape of the flower bud: roughly triangular with an obtuse apex, apparently remaining closed throughout development, with the petals not opening fully even at maturity. In most other species of the genus, including the otherwise very similar *C.confusum* (see notes under that species), the flower bud opens during development. *Cremastospermapendulum* and *C.yamayakatense* also exhibit glabrous, closed flower buds, but the shape of both is depressed ovoid. The latter also has a short, sturdy pedicel, very different to *C.monospermum*, the flower of which is borne on a slender and often long (though rather variable) pedicel.

The authors do not consider it useful to recognise sub-specific taxa within *C.monospermum*. The variation in stipe length and thickness represented by the type of var. brachypodum, described by Fries (1939), falls within that of the species as a whole and is therefore synonymised here.

#### Preliminary conservation status.

*Cremastospermamonospermum* is the most widespread and abundant species of the genus, including occurrences in protected areas in Brazil and Peru. Least concern [LC] (Table 1).

#### Selected specimens examined.

**BOLIVIA. Beni**: Chácobo, 11°45'S, 66°02'W, 200 m a.s.l., 15 Apr 1984, *Boom 5039* (K, LPB, MO, NY, U); Río Beni above Río Qui, 14°48'S, 67°23'W, 320 m a.s.l., 22 May 1990, *Daly et al. 6573* (LPB, MO, NY, U); Río Beni, Rurrenabaque, 14°28'S, 67°30'W, 320 m a.s.l., 25 May 1990, *Daly et al. 6630* (NY, U); San Borja, 15°10'S, 66°28'W, 250 m a.s.l., 23 Nov 1988, *R.B. Foster et al. 12549* (F, LPB, U); Bosque de Chimanes, 15°30'S, 66°15'W, 250 m a.s.l., 27 Oct 1989, *R.B. Foster* & *Terceros 13422* (U); Prov. José Ballivián, Carinavi-San Borja road, 15°17'S, 67°04'W, 850–900 m a.s.l., 1 Nov 1989, *D.N. Smith* & *V. Garcia 13834* (LPB); Bosque de Producción Permanente Chimanes, 15°10'S, 66°37'W, 260 m a.s.l., 25 Aug 1990, *D.N. Smith et al. 14216* (LPB, MO). **La Paz**: Luisita, 13°50'S, 67°15'W, 180 m a.s.l., 27 Feb 1984, *Beck* & *Haase 10092* (LPB, MO, U); Santa Fé, 13°40'S, 68°12'W, 250 m a.s.l., 5 Aug 1995, *DeWalt et al. 662* (LPB, U, WU); Puerto Muscoso, 13°01'S, 68°50'W, 190 m a.s.l., 25 Jun 1995, *Helme* & *Kruger 742* (LPB, MO, U); Alto Beni, Sapecho, 15°30'S, 67°20'W, 550 m a.s.l., 3 Apr 1989, *Seidel 2674* (LPB, U). **Pando**: Manuripi, 11°40'S, 68°00'W, 18 Aug 1990, *G. Gonzales 53* (LPB); Puerto América, 11°35'S, 68°03'W, 180 m a.s.l., 9 Sep 1995, *Jardim 2408* (U); Agua Clara, 11°44'20"S, 67°02'40"W, 13 Nov 2001, *Pirie et al. 4* (U); Puerto Rico, road to Cobija, 11°03'25"S, 67°47'46"W, 175 m a.s.l., 15 Nov 2001, *Pirie et al. 5* (U). **BRAZIL. Acre**: Fazenda Bom Sossego, 7°40'S, 73°09'W, 27 Sep 1985, *Campbell et al. 9026* (U); Seringal Porongaba, 10°51'S, 68°48'W, 23 May 1991, *Daly et al. 6686* (MO, NY, U); Mun. Rio Branco, BR 317, 10°30'S, 67°45'W, 8 Jun 1991, *Daly et al. 6909* (MO, NY, U); Reserva Extrativista do Alto Jurua, 8°55'S, 72°31'W, 11 Mar 1992, *Daly et al. 7347* (MO, NY, U); Mun. Porto Acre, 9°45'S, 67°38'W, 3 Nov 1993, *Daly et al. 8043* (NY, U); Mun. Cruzeiro do Sul, 8°13'41"S, 73°02'22"W, 11 May 2003, *Daly et al. 11719* (NY); Mun. Tarauacá, Seringal Universo, 8°25'S, 71°19'W, 15 Jun 1995, *Figueiredo et al. 853* (U); Rio Muru, Seringal Vitoria Velha, 8°26'S, 70°49'W, 20 Jun 1995, *Figueiredo et al. 906* (U); Mun. Sena Madureira, Rio Macaua, 9°24'S, 68°54'W, 3 Apr 1994, *L. Lima et al. 582* (NY); Rio Branco-Porto Acre road, 10°00'S, 67°50'W, 11 Oct 1980, *Nelson 680* (U); Riozinho do Andirá, colocação Curitiba, 9°43'45"S, 68°08'53"W, 6 Jun 1998, *A.R.S. Oliveira et al. 488* (WAG); Porto Walter, Rio Juruá Mirim, 8°07'S, 72°49'W, 31 May 1994, *Silveira et al. 767* (NY, U); Tarauacá, 9°25'45"S, 68°40'00"W, 18 Jun 2006, *Silveira et al. 3796* (L); Mun. Tarauacá, Rio Liberdade, 7°18'49"S, 66°01'46"W, 18 Jun 2006, *Silveira et al. 3811* (L); Floresta Estadual do Antimary, 9°25'45"S, 68°04'00"W, 8 Jul 2006, *Silveira et al. 4171* (L). **Mato Grosso**: Mun. Alta Floresta, 10°07'S, 57°30'W, 29 Sep 1985, *Cid Ferreira et al. 6301* (F, GH, K, MICH, NY, U, US). **Pará**: Itaituba, 7°40'S, 55°15'W, 14 May 1983, *M.N. Silva 322* (MO, NY, U, US); Serra dos Carajás, 5°49'S, 50°32'W, 225–250 m a.s.l., 15 Jun 1982, *Sperling et al. 6198* (GH, K, MO, NY, U). **Rondônia**: Mun. Costa Marques, Chapada dos Parecís, 11°12'S, 62°63'W, 14 Jun 1984, *Cid Ferreira et al. 4507* (MO, NY, U); Espigão de Oeste, 11°12'S, 60°61'W, 20 Jun 1984, *Cid Ferreira et al. 4673* (F, GH, MO, NY, U, US); Ariquemes, 10°00'S, 62°59'W, 200 m a.s.l., 15 Mar 1987, *Nee 34402* (U); Campo Novo, 10°38'S, 63°37'W, 300 m a.s.l., 23 Apr 1987, *Nee 34992* (MO); Porto Velho, Campo Novo, road to Ariquemes, 10°35'S, 63°30'W, 300 m a.s.l., 25 Apr 1987, *Nee 35033* (U); Ariquemes, Mineração Mibrasa, 10°35'S, 63°35'W, 15 May 1982, *Teixeira 480* (MO, U). **PERU. Huánuco**: Pucallpa, western Sira mountain, 9°28'S, 74°47'W, 680 m a.s.l., 7 Apr 1988, *Wallnöfer 112 7488* (U). **Loreto**: Quebrada Shesha, Río Abujao, 8°20'S, 73°45'W, 14 Dec 1978, *C. Díaz et al. 825* (NY, U); Shucushayacu, Río Huallaga, 6°01'S, 75°55'W, 180 m a.s.l., 11 Oct 1985, *Gentry et al. 52233* (MO, U); Pongo de Manseriche, 4°26'S, 77°34'W, 650 m a.s.l., 25 Nov 1997, *R. Rojas et al. 666* (F, MO); Canchahuayo, Río Ucayali, 7°05'S, 75°10'W, 500 m a.s.l., 25 Nov 1985, *Vásquez et al. 6932* (F, U); Yanamono Explorama Reserve, 3°28'S, 72°50'W, 106 m a.s.l., 3 Oct 1989, *Vásquez* & *N. Jaramillo 12829* (MO, MOL, U). **Madre de Dios**: Tambopata, Río Heath, 12°39'S, 68°44'W, 210 m a.s.l., 22 May 1996, *M. Aguilar* & *Castro 764* (U, USM); Cuzco Amazónico, 15 km ENE of Puerto Maldonado, 12°35'S, 69°05'W, 200 m a.s.l., 14 Dec 1989, *Gentry et al. 68663* (MO, USM); Inkaterra Ecological Reserve, 12°35'S, 69°09'W, 190 m a.s.l., 30 Aug 2006, *Monteagudo et al. 12749* (WAG); Tambopata, 12°33'S, 69°03'W, 200 m a.s.l., 27 May 1989, *Núñez et al. 10589* (MO); Cuzco Amazónico Lodge, 12°35'S, 69°03'W, 200 m a.s.l., 13 May 1990, *Núñez* & *Timaná 12152* (U); Collpa de Blanquillo, Río Madre de Dios, 12°27'S, 70°41'W, 200 m a.s.l., 11 May 1995, *Núñez 16303* (USM); Cuzco Amazónico, 12°05'S, 69°03'W, 200 m a.s.l., 21 Jun 1989, *Phillips et al. 545* (MO, U); Las Piedras, Cusco Amazónico, 12°29'S, 69°03'W, 200 m a.s.l., 25 Feb 1991, *Timaná 1537* (MO); Puerto Maldonado, Cusco Amazónico, 12°32'S, 69°03'W, 220 m a.s.l., 20 May 2003, *L. Valencia* & *Suclli 2205* (U). **Ucayali**: Peru-Brazil border, Quebrada Sapallal, 8°02'S, 73°55'W, 260 m a.s.l., 19 Jun 1987, *Gentry* & *C. Diaz 58434* (USM); Prov. Purus, Distr. Purus, Río Caranja, 10°04'S, 71°06'W, 325 m a.s.l., 17 Jul 1998, *Graham 605* (F); Prov. Coronel Portillo, Iparia, el Sira, 9°25'57"S, 74°32'47"W, 350–400 m a.s.l., 21 Sep 2007, *Graham 4706* (L); Prov. Coronel Portillo, Calleria, 8°09'13"S, 74°15'48"W, 150–175 m a.s.l., 26 Feb 2003, *Schunke V. 15223* (F).

### 22. Cremastosperma napoense

Taxon classificationPlantaeMagnolialesAnnonaceae

Pirie

Fig. 31 , Map 3

Cremastosperma napoense Pirie, Blumea 50: 54, f. 7. 2005.

#### Type.

ECUADOR, Napo: Cantón Archidonia, foothills south of Volcano Sumaco, km 50 on Hollín – Loreto road, community Huahua Sumaco, 3 May 1989, *Alvarado, A. 267* (holotype: U! [U0012198]; isotypes: AAU!, MO! [MO-047571], QCNE).

#### Description.

*Tree* 5–20 m tall, 10–15 cm diam.; young twigs and petioles rather densely covered with appressed whitish-golden hairs to 0.2 mm long. *Leaves*: petioles 8–12(-18) by 3–4 mm; lamina narrowly elliptic to elliptic, 17–42 by 7–13 cm (index 1.8–3.9), chartaceous, olive green or brown on both sides, venation darker below, glabrous above, rather densely covered with appressed whitish-golden hairs to 0.2 mm long on veins below, base acute, apex acute to acuminate (acumen 5–15 mm long), primary vein grooved over entire leaf length, verrucose at the base, 3–4 mm wide at widest point, secondary veins (8-)10–15, occasionally 1 or 2 intersecondary veins, distance between from 4–9 mm at the base, 20–50(-60) mm in the centre to 10–30 mm closer to the apex, angles with primary vein from 40–50° at the base to 70–80° closer to the apex, occasionally branching, forming more or less distinct loops in the apical half, tertiary veins percurrent. *Inflorescence* of 1–8 flowers, branching, solitary or clustered in groups of 2, on leafless twigs and branches; peduncles 7–22 by 1–1.5 mm (in flower), 10–22 by 2.5–3 mm (in fruit); pedicels 25–38 by ca. 1 mm at the base, 1.5–2 mm at the apex (in flower), 25–38 by 2–3 mm at the base, 2–4 mm at the apex (in fruit), peduncles and pedicels rather densely to densely covered with appressed whitish to golden hairs to 0.2 mm long; single lower bract, soon falling off; upper bract attached around halfway along the pedicel, broadly to depressed elliptic or broadly to depressed ovate, 1–2 by 1.5–1.8 mm, obtuse, outer side densely covered with appressed whitish-golden hairs to 0.2 mm long; closed flower buds depressed ovoid, opening early in development; flowers green, maturing to greenish-yellow or cream *in vivo*, brown *in sicco*; sepals fused for first 0.5 mm, deltate, appressed (basal 1 mm of sepals and petals reflexed), 2.5–3 by ca. 3 mm, acute or obtuse, soon falling off, inner side glabrous, outer side rather densely to densely covered with appressed whitish-golden hairs to 0.2 mm long; outer petals elliptic, 8–15 by 5–9 mm, inner petals elliptic, 12–14 by 5–7 mm, inner side of inner and outer petals glabrous, most of outer side of outer petals rather densely to densely covered with appressed whitish-golden hairs to 0.2 mm long (towards the margins and apex glabrous), outer side of inner petals largely glabrous but with a narrow, dense, sometimes branching band of appressed, whitish-golden, to 0.2 mm long hairs extending from the base to halfway towards the apex; receptacle depressed ovoid; androecium ca. 6 mm diam., stamens 1–1.5 mm long, connective appendage 0.5–0.8 mm wide; gynoecium ca. 2 mm diam., carpels up to ca. 40, ca. 1.5 mm long, glabrous or sparsely covered with appressed, whitish-golden, to 0.2 mm long hairs. *Monocarps* 16–37, ellipsoid, asymmetrical, 12–20 by 10–13 mm, green maturing to dark purple or black *in vivo*, black *in sicco*; stipes 20–30 by 2–3 mm; fruiting receptacle depressed ovoid, 7–12 mm diam., monocarps, stipes and receptacle glabrous. *Seeds* ellipsoid, brown, lightly furrowed (not pitted), ca. 13 by 9 mm, raphe neither sunken nor raised, encircling seed longitudinally.

#### Distribution.

Ecuador (Napo, one collection in Pastaza).

#### Habitat and ecology.

Primary pluvial premontane forest, often on volcanic soils but also reported growing on limestone. At elevations of 600–1300 m. Flowering: September, November, December and February; fruiting: August to December, March to May.

#### Vernacular names.

Ecuador: Ayacara (Quichua; *Alvarado 267*, *Cerón & Hurtado 6663, Hurtado & Alvarado 322, 896, 941, Hurtado et al. 2096*), Naguan (*T.D. Pennington et al. 12266*).

#### Notes.

The characteristic pattern of indument on the inner petals of *Cremastospermanapoense* appears to be unique for the genus. The species can be further distinguished by the combination of a branching inflorescence and glabrous fruits. The only other species in the genus with such an inflorescence are *C.alticola* and *C.cauliflorum*. *Cremastospermaalticola* differs in the much shorter stipes and larger monocarps. *C.cauliflorum* differs both in the presence of brown indument on the (characteristic globose to transversely broadly elliptic) monocarps and in the dense covering of much longer hairs on the flowers.

#### Preliminary conservation status.

*Cremastospermanapoense* is represented by a moderate number of collections spread across an area that, due to its limited size, would qualify as Vulnerable; however it includes sites wthin a national park that might ameliorate habitat decline. Near Threatened [NT] (Table 1).

#### Selected specimens examined.

**ECUADOR. Napo**: Parque Nacional Sumaco, 0°44'S, 77°34'W, 1100 m a.s.l., 1 May 1991, *Alvarado 427* (U); Hollín-Loreto road, foothills of Volcán Sumaco, 0°38'S, 77°27'W, 1000 m a.s.l., 29 Apr 1989, *Cerón* & *F. Hurtado 6663* (U); Reserva de Biosfera Sumaco, 0°49'39"S, 77°33'47"W, 1160 m a.s.l., 26 Feb 2003, *Cevallos 55* (MO); Hollín-Loreto road, 0°43'S, 77°40'W, 1230 m a.s.l., 10 Nov 1988, *F. Hurtado* & *Alvarado 896* (U); Codo Bajo, 0°30'S, 77°15'W, 660 m a.s.l., 18 Sep 1990, *J. Jaramillo et al. 12840* (AAU); Gonzalo Pizarro, Río Tigre, Lumbaqui-Reventador rd, 0°05'S, 77°24'W, 900–1100 m a.s.l., 19 Feb 1987, *Neill* & *Palacios 7649* (MO, U); Hollín-Loreto-Coca road, 0°40'S, 77°00'W, 1200 m a.s.l., 11 Dec 1987, *Neill et al. 8089* (AAU, GB, K, MO, NY, QCNE, U); Volcán Sumaco, 5 km E of Huamaní, 0°44'S, 77°35'W, 1100 m a.s.l., 19 Oct 1989, *Neill* & *Palacios 9088* (U); Cantón El Chaco, Río Granadillo, 0°08'S, 77°28'W, 1300 m a.s.l., 13 Sep 1990, *Palacios 5485* (U). **Pastaza**: Puyo, Colonia Bolívar, 1°23'S, 77°45'W, 1000 m a.s.l., 15 Dec 1997, *Neill 11048* (U). **Sucumbíos**: Sinangoe Station, Cucocco beach camp, 0°07'49"S, 77°33'20"W, 8 Aug 2001, *Aguinda et al. 1581* (F); Cofán de Sinangüe, 0°08'N, 77°27'W, 700–800 m a.s.l., 4 Dec 1992, *Cerón 20815* (MO, U).

**Figure 31.**
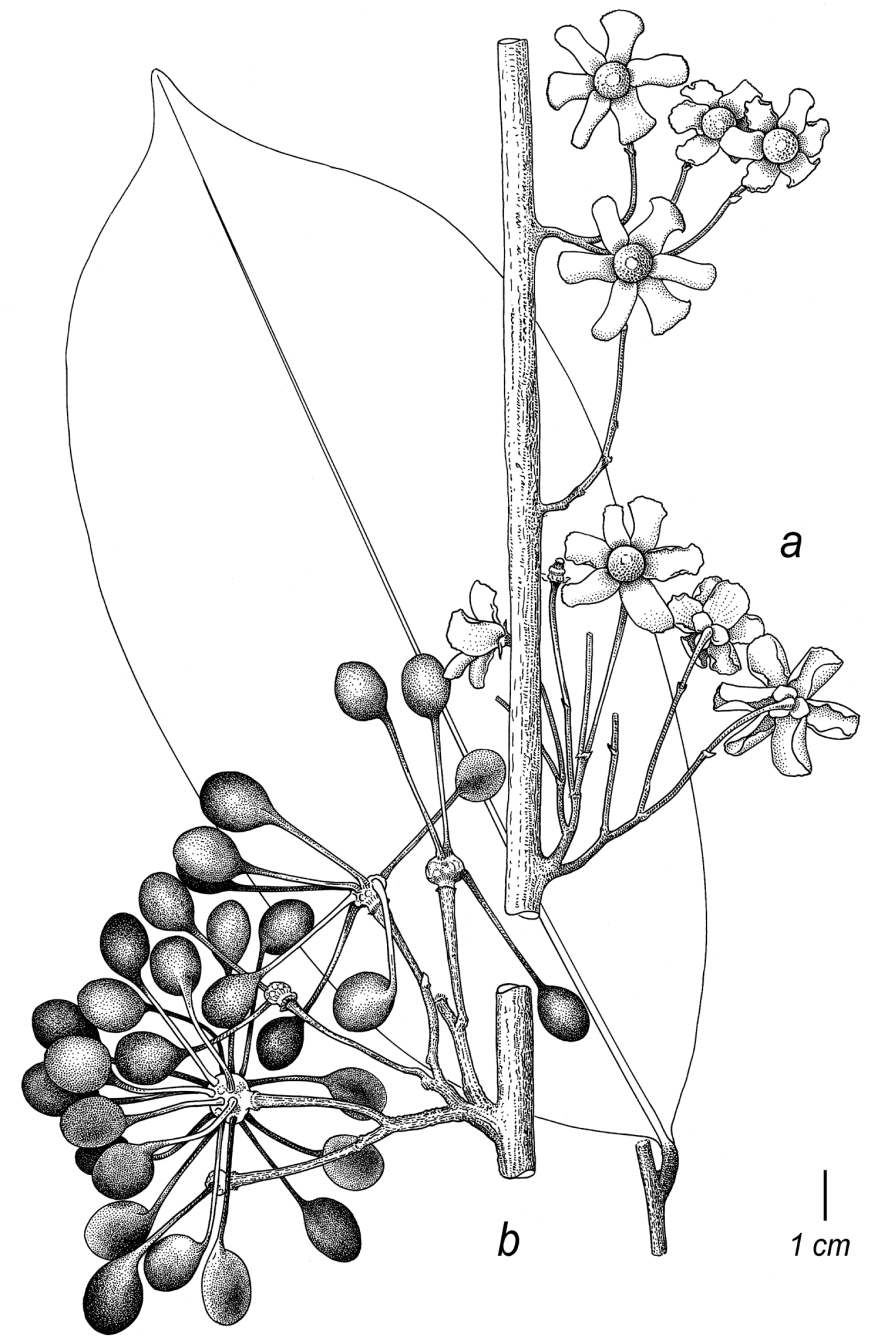
*Cremastospermanapoense* Pirie. **a** inflorescences **b** infrutescence and leaf (**a***Cerón 2986***b***Alvarado 267*).

### 23. Cremastosperma novogranatense

Taxon classificationPlantaeMagnolialesAnnonaceae

R.E.Fr.

Fig. 32 , Map 2

Cremastosperma novogranatense R.E.Fr., Ark. Bot. 1: 329. 1950.

#### Type.

COLOMBIA, Valle del Cauca: Costa del Pacifico, Río Cajambre, Silva, 5–80 m a.s.l., 5–15 May 1944, *Cuatrecasas, J. 17573* (holotype: S! [two sheets S-R-6965, S10-19867]; isotypes: F! [V0054583F, V0054584F], US! [US00811252, US00104265], VALLE! [VALLE000050, VALLE000051]).

#### Description.

*Tree* 8–20 m tall, 5–13 cm diam.; young twigs and petioles densely covered with appressed or erect golden to whitish hairs to 0.4 mm long. *Leaves*: petioles (6-)10–20 by 3–5 mm; lamina elliptic to obovate (24-)32–50 by 11–21 cm (index 2.2–2.8), chartaceous, pale brownish-green to greyish-green above, pale brownish-green below, glabrous above, veins sparsely to rather densely covered with appressed golden to whitish hairs to 0.5 mm long below, base obtuse to rounded, apex acuminate (acumen 15–40 mm long), primary vein 2–4 mm wide at widest point, secondary veins 10–13, intersecondary veins 1–3, distance between from 4 mm at the base to up to 40 mm closer to the apex, angles with primary vein from 50–70° at the base to 45–55° closer to the apex, not branching forming mostly distinct loops, smallest distance between loops and margin 2–7 mm, tertiary veins percurrent. *Inflorescence* of single flowers, solitary (or clustered in groups of 2), on leafy or leafless twigs; peduncles 2–3 by ca. 2 mm (in flower), 2–4 by 2.5–3 mm (in fruit); pedicels 10–20 by ca. 2 mm (in flower), 13–27 by 2.5–3 mm (in fruit), peduncles and pedicels sparsely to densely covered with appressed golden hairs to 0.4 mm long; single lower bract, ovate, ca. 2 by 1.5 mm, acute, often persistent, outer sides of bracts, of sepals and of petals densely covered with appressed golden hairs to 0.6 mm long; upper bract in the lower half of the pedicel, broadly to depressed ovate, 2–3.5 by 1.5–2 mm, acute; closed flower buds globose; flowers pale green, sepals light brownish-green *in vivo*, outer side of sepals and petals dark yellow or greyish-green, inner side reddish-brown or dark brown *in sicco*; sepals free, ovate to broadly ovate, appressed or patent, 7–10 by 7–8 mm, obtuse, often persistent, with prominent venation; outer petals elliptic, 17–29 by 10–16 mm, obtuse, inner petals narrowly elliptic, 28–33 by 9–12 mm, obtuse; androecium not seen; stamens 1.6–1.9 mm long, connective appendage 0.5–0.8 mm wide; gynoecium not seen. *Monocarps* 3–14, ellipsoid to broadly ellipsoid, asymmetrical, 16–22 by 10–13 mm, yellow, orange, red or pale brown *in vivo*, blackish-brown to black *in sicco*, with an excentric apicule; stipes 1.5–4 by 2 mm; fruiting receptacle 4–10 mm diam.; monocarps, stipes and receptacle sparsely to rather densely covered with appressed brown hairs to 0.2 mm long. *Seeds* ellipsoid, yellowish-brown, very shallowly pitted, ca. 16 by 10 mm, raphe sunken, irregular.

#### Distribution.

Pacific coast of Colombia (Valle del Cauca).

#### Habitat and ecology.

Rainforest. At elevations of 0–130 m. Flowering: September and October; fruiting: May, September.

#### Notes.

*Cremastospermanovogranatense* can be distinguished by its almost astipitate monocarps and by the large and densely hairy flowers with unusually large, often persistent, sepals. It is most similar to *C.westrae*, the sepals of which are much smaller and indument in general of shorter, less dense hairs.

#### Preliminary conservation status.

*Cremastospermanovogranatense* is represented by just seven collections within a small area without protected status. Endangered [EN] (Table 1).

#### Selected specimens examined.

**COLOMBIA. Valle del Cauca**: Río Calima, Chocó region, 20–40 m a.s.l., 24 May 1946, *Cuatrecasas 21288* (F, P, S, US); Río Calima, Quebrada de López, 20–40 m a.s.l., 23 Sep 1961, *Cuatrecasas et al. 26031* (US); Córdoba, 3°49'41"N, 76°52'10"W, 130 m a.s.l., 16 Oct 1996, *Devia et al. 5335* (MO); Bajo Calima, 3°50'N, 77°10'W, 50 m a.s.l., 7 May 1987, *Faber-Langendoen* & *Renteria 476* (U); Bajo Calima, Juanchaco Palmeras, 3°56'N, 77°08'W, 50 m a.s.l., 18 Apr 1987, *Gentry et al. 57078* (MO, U); Bajo Calima, 3°55'N, 77°00'W, 100 m a.s.l., 24 Sep 1987, *Monsalve 1816* (MO).

**Figure 32.**
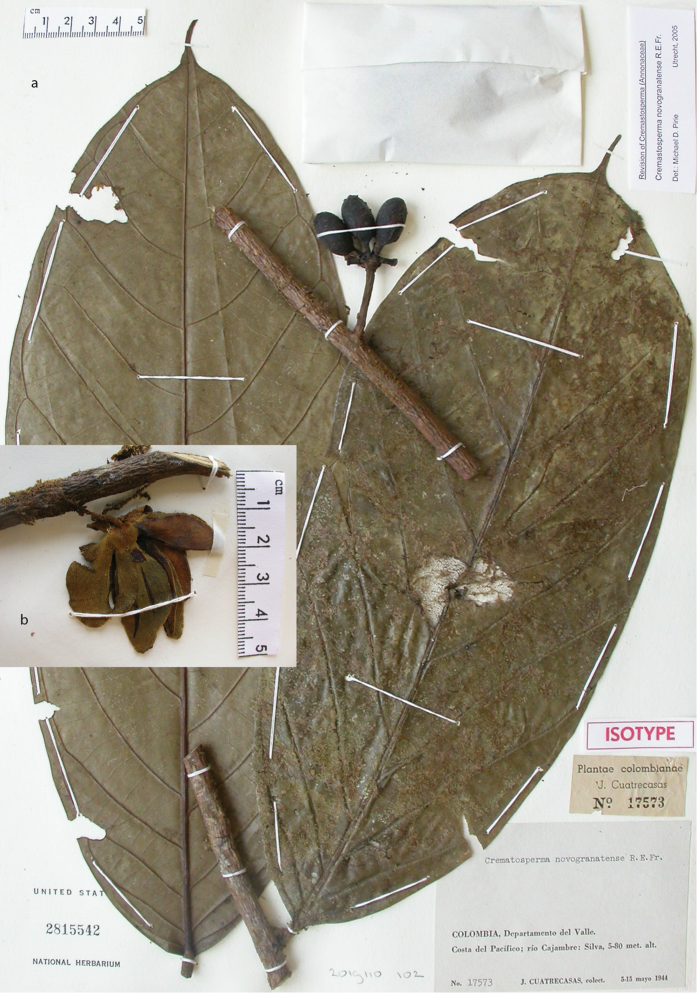
*Cremastospermanovogranatense* R.E.Fr. **a** fruiting specimen **b** flower (**a***Cuatrecasas 17573***b***Cuatrecasas & Willard 26031*).

### 24. Cremastosperma oblongum

Taxon classificationPlantaeMagnolialesAnnonaceae

R.E.Fr.

**Figure 33.**
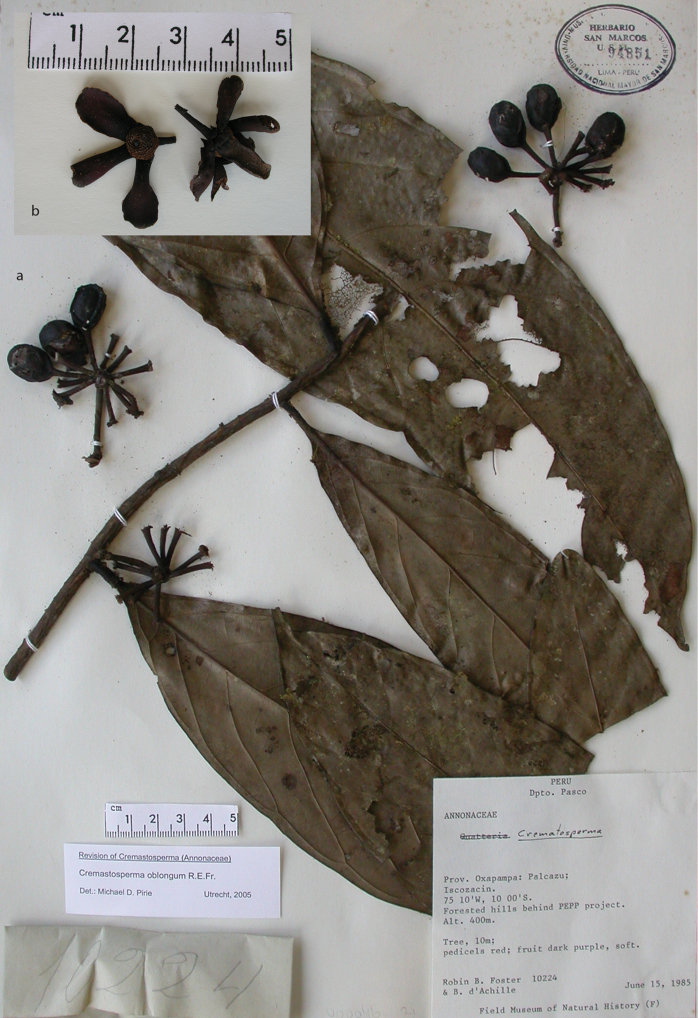
*Cremastospermaoblongum* R.E.Fr. **a** fruiting specimen **b** flower (**a***Foster & d’Achille 10224*; **b***Maas et al. 4592*).

Figs 23 , 33 , Map 6

Cremastosperma oblongum R.E.Fr., Konl. Svenska Vetenskapsakad. Handl. 24: 4. 1948.

#### Type.

PERU, Junín: Río Pinedo, N of La Merced, 30 May 1929, *Killip, E.P. & Smith, A.C. 23622* (holotype: US! [US00104266]; isotypes: NY! [NY00025863], S! [S-R-6966]).

#### Description.

*Tree* or *shrub* 2–17 m tall, 6–15 cm diam.; young twigs and petioles sparsely covered with appressed golden hairs to 0.4 mm long. *Leaves*: petioles 5–16 by 2–4 mm; lamina elliptic, obovate or narrowly so, 13–45 by 5–12 cm (index 2.5–4), coriaceous, shiny grey-green or brown above, dull brown below, glabrous above, sparsely covered with appressed golden hairs to 0.4 mm long on veins below, base acute, rarely cordate, apex acuminate (acumen (5–)10–20 mm long), primary vein grooved in basal half, 2–4 mm wide at widest point, secondary veins 8–19, intersecondary veins often 1(–2), distance between from 8–24 mm at the base to 12–27 mm closer to the apex, angles with primary vein from 40–70° at the base to 20–60° closer to the apex, sometimes branching, forming distinct loops, smallest distance between loops and margin 2–5 mm, tertiary veins mostly percurrent. *Inflorescence* of single flowers clustered in groups of up to three, on leafy or leafless twigs (occasionally on main stem, then on brachyblasts); peduncles ca. 2 by 1 mm (in flower), 2–5 by 1.5–3 mm (in fruit); pedicels 7–18 by ca. 1 mm at the base (in flower), 12–35(–80) by 1.5–3 mm (in fruit), peduncles and pedicels sparsely covered with appressed whitish hairs to 0.1 mm long; 2 or 3 lower bracts, deltate, 0.5–1 by 0.5–1 mm, obtuse, soon falling off or persistent, sparsely covered with appressed whitish hairs to 0.1 mm long; upper bract attached in basal half of pedicel, shallowly triangular, ca. 1 by ca. 2 mm, obtuse, persistent, sparsely covered with appressed whitish hairs to 0.1 mm long; closed flower buds not seen; flowers green maturing to cream, yellow or orange/yellow *in vivo*, black *in sicco*; sepals free, deltate, often recurved, ca. 2 by 2 mm, acute, soon falling off, sparsely covered with appressed whitish hairs <0.1 mm long or glabrous; petals glabrous, outer petals obovate to narrowly so, ca. 16 by 6–8 mm, inner petals elliptic to narrowly so, 15–16 by 4–6 mm; androecium ca. 8 mm diam., stamens 1–1.5 mm long, connective appendage 0.5–0.8 mm wide; gynoecium ca. 1.5 mm diam., carpels sparsely covered with erect golden hairs <0.1 mm long. *Monocarps* 6–20, ellipsoid, asymmetrical, 16–20 by 10–14 mm, green maturing through orange or red to black *in vivo*, brown or black *in sicco*, with an excentric apicule; stipes green maturing to red *in vivo*, 9–17 by 2–3 mm; fruiting receptacle 7–12 mm diam.; monocarps, stipes and receptacle glabrous. *Seeds* ellipsoid, reddish-brown, pitted, ca. 13 by 8 mm, raphe raised, regular.

#### Distribution.

Central and southern Peru (Cuzco, Huánuco, Junín, Loreto, Madre de Dios, Pasco, San Martín and Ucayali) and adjacent Brazil (Acre).

#### Habitat and ecology.

Primary, often upland, rainforest, on white sands, brown latosols and limestone soils. At elevations of 100–1800 m. Flowering: September, October, December; fruiting: March-July, October-January.

#### Vernacular names.

Peru: Bara caspi, Carahuasca (*Bulnes 502*), Carahuasca amarilla (*Tello 1241*), Hicoja (*Schunke V. 5829*), Palo blanco, Tortuga blanca (*Tello 165*), Ts’ntonimaski (*D.N. Smith 6850*), Yana huasca (*Williams, Ll 7423*).

#### Notes.

*Cremastospermaoblongum* is best discerned from the most similar other species on the basis of floral characters: the sepals are small and recurved (unlike *C.megalophyllum*) and borne on short pedicels, whilst bud development is open (not the case in *C.yamayakatense*). A similar species that also occurs in Central Peru is *C.dolichopodum*, which can be distinguished by the much greater length of the pedicels (both in flower and fruit). Two further undetermined collections from this region (*Valenzuela 13205* and *Vásquez 35950*) are also similar, but differ in the presence of indument on the sepals and receptacles. The leaves of *C.oblongum* are quite distinctive: rather leathery with a greyish colour on the upper side, with secondary veins forming conspicuous loops and often narrowly elliptic. Fruiting specimens display more variation – particularly in the length of the pedicel. Cauliflorous specimens from the Peruvian department of Pasco (e.g. *D.N. Smith 6613* and *6850*; *Monteagudo et al. 11777*) have longer pedicels; the fruits otherwise resemble somewhat those of *C.dolichopodum* (the pedicels of which are several times longer).

#### Preliminary conservation status.

*Cremastospermaoblongum* is only found in southern Peru, but within its fairly wide EOO, it is not uncommon, including within protected areas. Least concern [LC] (Table 1).

#### Selected specimens examined.

**BRAZIL. Acre**: Unidade de Assentamento Santa Luzia, 7°45'S, 72°22'W, 2001, *Maas et al. 9148* (L, U). **PERU. Huánuco**: Selva Central, Dantas, Llullapichis, 9°40'S, 75°02'W, 280 m a.s.l., 29 Jan 1986, *Bulnes 502* (U); Selva Central, Dantas, Yuyapichis, 9°40'S, 75°02'W, 270 m a.s.l., 16 Dec 1989, *Flores* & *Tello 165* (G); Pucallpa, western Sira mountain, 9°29'S, 74°50'W, 300–360 m a.s.l., 28 Sep 1988, *Wallnöfer 117 28988* (U). **Loreto**: Panguana, SE of Pucallpa, junction of Río Pachitea, 9°37'S, 74°56'W, 260 m a.s.l., 25 Sep 1985, *Morawetz* & *Wallnöfer 110 25985* (U, USM). **Pasco**: Palcazú, Iscozacin, 10°00'S, 75°10'W, 400 m a.s.l., 15 Jun 1985, *R.B. Foster* & *Achille 10224* (F, MOL, U, USM); Huampal, 10°15'00"S, 75°13'26"W, 1100–1250 m a.s.l., *Monteagudo et al. 4933* (HOXA); Distr. Iscozacín, 10°09'S, 75°18'W, 23 Sep 1986, *Pariona* & *J. Ruíz 983* (MO); Parque Nacional Yanachaga-Chemillen, 10°10'50"S, 75°34'26"W, 900–1200 m a.s.l., 3 Nov 2003, *Pirie et al. 7* (HOXA, U, USM); Palcazú, 10°08'00"S, 75°22'06"W, 500 m a.s.l., 31 Mar 2009, *R. Rojas et al. 6598* (HOXA, MO, USM); Cordillera de San Matias, 10°11'S, 75°12'W, 680–850 m a.s.l., 21 Jun 1982, *D.N. Smith 2018* (MO); Gran Pajonal, 10°45'S, 74°23'W, 1200 m a.s.l., 30 Mar 1984, *D.N. Smith 6613* (MO, U); Palcazú, Ataz, 10°09'20"S, 75°19'45"W, 652 m a.s.l., 22 May 2009, *Valenzuela et al. 12782* (HOXA, HUT, MO, USM); Palcazú, Cerro Ozuz, 10°19'00"S, 75°17'30"W, 850–1010 m a.s.l., 10 Sep 2005, *Vilca 411* (WAG).

### 25. Cremastosperma osicola

Taxon classificationPlantaeMagnolialesAnnonaceae

Pirie & Chatrou sp. nov.

urn:lsid:ipni.org:names:60477510-2

**Figure 34.**
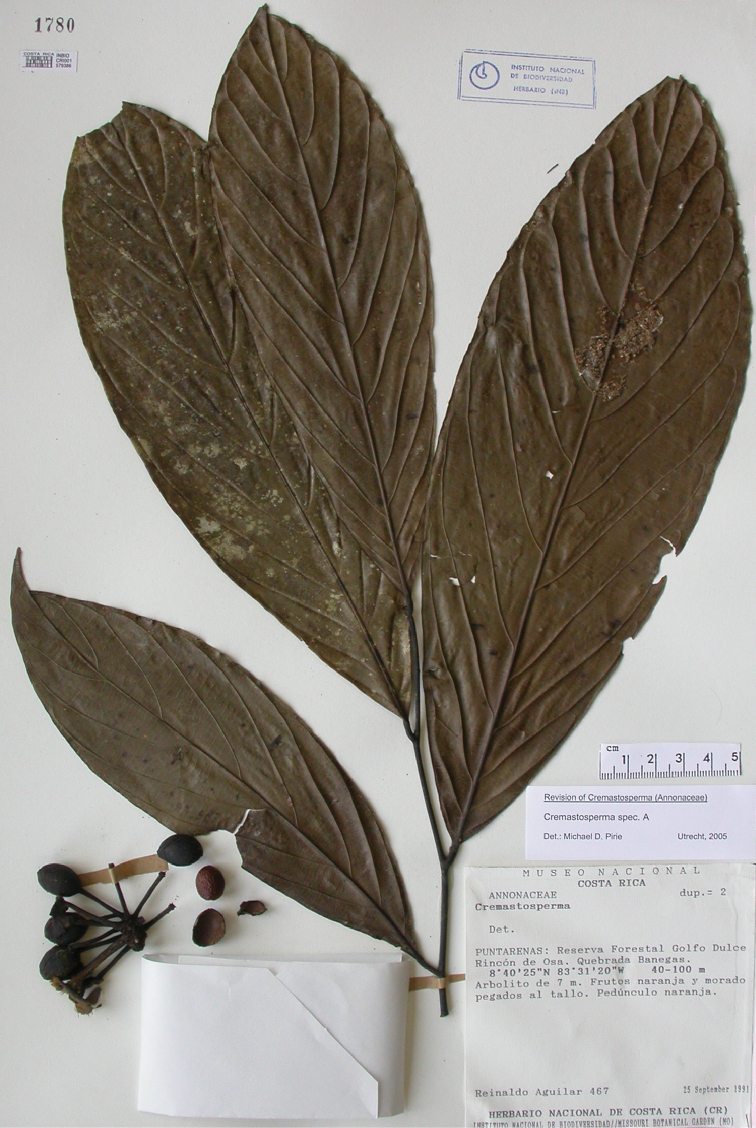
*Cremastospermaosicola* Pirie & Chatrou fruiting specimen (*Aguilar 467*).

Figs 23 , 34 , Map 2

#### Diagnosis.

Differs from *C.megalophyllum*, *C.pacificum* and *C.yamayakatense* in the fully open bud development; from *C.brevipes, C.magdalenae*, *C.novogranatense*, *C.pacificum* and *C.westrae* in the stipes being longer than the monocarps and (except *C.magdalenae*) in the usually much larger number of monocarps (20–40 as opposed to mostly fewer than 20); from *C.westrae* and *C.novogranatense* in the absence of indument on the fruits; from *C.panamense* in the greater size of the fruits and the shape of the monocarps (ellipsoid, compared to more or less globose in *C.panamense*); and from all of the above except *C.novograntense* in the much larger flowers.

#### Type.

COSTA RICA, Puntarenas: Cantón Osa, Distr. Sierpe, Los Mogos, Bahia de Chal, Entrada San Juan, 9 Sep 2011, *Chatrou, L.W. et al. 707* (holotype: CR!; isotypes: K!, MO!, P!, U!).

#### Description.

*Tree* 3–7 m tall, 5–7 cm diam.; young twigs and petioles very sparsely covered with appressed whitish hairs to 0.2 mm long or glabrous. *Leaves*: petioles 7–15(–20) by 2–4 mm; lamina obovate to elliptic or narrowly so, 21–44 by 5–15 cm (index 2.6–3.4), chartaceous, green or brown above and below, veins darker below, glabrous above, very sparsely covered with appressed whitish hairs to 0.2 mm long, especially on veins or glabrous below, base acute or obtuse, apex acuminate (acumen 10–20 mm long), primary vein shallowly grooved near base, 1.5–2 mm wide at widest point, secondary veins 7–10, intersecondary veins 0–1, distance between from 10–13 mm at the base to 25–45 mm closer to the centre, angles with primary vein from 45–60° at the base to 70–80° closer to the centre, sometimes branching, forming distinct loops in apical half of leaf, smallest distance between loops and margin 2–3 mm, tertiary veins mostly percurrent. *Inflorescence* of single flowers solitary on leafless twigs or clustered in groups on brachyblasts on the main stem; peduncles 4 by 4 mm (in fruit); pedicels 18–23 by 3 mm at the base, 3–4 mm at the apex (in fruit), peduncles and pedicels glabrous; lower bract(s), upper bract and closed flower buds not seen, flowers (observed from photo: Fig. 21 o) cream in vivo; sepals not seen; petals indument not seen, outer petals elliptic, ca. 31 mm long, inner petals elliptic, ca. 27 by 9 mm; androecium ca. 7 mm diam., stamens not seen; gynoecium ca. 2 mm diam., carpels not seen. *Monocarps* 20–43, ellipsoid (broadly so in immature specimens), slightly asymmetrical, 11–17 by 11–12 mm, green maturing to yellowish, orange and purple *in vivo*, black *in sicco*, with a small excentric apicule, monocarps, stipes and receptacle glabrous; stipes 17–30 by 1.5–2 mm; fruiting receptacle ca. 14 mm diam. *Seeds* ellipsoid, reddish-brown, shallowly pitted, ca. 12 by 10 mm, raphe sunken, regular.

#### Distribution.

Costa Rica (Puntarenas, Osa peninsula).

#### Habitat and ecology.

Tropical wet forest. At elevations of 40–300 m. Fruiting: July and September.

**Notes.** The distribution of *Cremastospermaosicola* in Costa Rica is the furthest north into Central America of any species of the genus. The species is most similar to *C.pacificum*, from the Pacific coast of Colombia, from which it can be discerned with flowering material by the much larger flowers (outer petals ca. 31 mm compared to ca. 16 mm in *C.pacificum*) and with fruiting material by the length of the stipes exceeding that of the monocarps.

#### Preliminary conservation status.

*Cremastospermaosicola* is known from a small number of populations within a region sufficiently small to qualify it as Endangered, but within a protected area (Golfo Dulce Forest Reserve). Near Threatened [NT] (Table 1).

#### Other specimens examined.

**COSTA RICA. Puntarenas**: Osa, Golfo Dulce Forest Reserve, 8°40'N, 83°31'W, 40–100 m a.s.l., 25 Sep 1991, *R.Aguilar 467* (INB, MO); Cantón de Osa, 8°43'20"N, 83°26'40"W, 20–100 m a.s.l., 13 Nov 1995, *R. Aguilar et al. 4371* (INB); Osa, Bahia Chal-La Palma road, 8°44'N, 83°26'W, 80 m a.s.l., 29 Nov 1998, *Chatrou et al. 103* (U); Osa, Rancho Quemado, near Rincón, 8°42'N, 83°33'W, 300 m a.s.l., 11 Jan 1993, *Gentry et al. 78657* (F, INB, MO); Osa, Parque Nacional Corcovado Cerro Brujo, 8°39'00"N, 83°35'50"W, 617 m a.s.l., 19 Jul 1990, *G. Herrera et al. 3972* (INB); Osa, Parque Nacional Corcovado Sirena, 8°28'N, 83°35'W, 1 50 m a.s.l., 20 Jul 1989, *Kernan 1224* (U); Osa, Golfo Dulce Forest Reserve, 8°42'N, 83°25'W, 100 m a.s.l., 2 Jul 1984, *Schatz et al. 1002* (MO, U, WIS); Osa, Golfo Dulce Forest Reserve, 8°43'N, 83°26'W, 20 m a.s.l., 24 Jul 1995, *Zamora* & *R. Aguilar 2312* (INB, U).

### 26. Cremastosperma pacificum

Taxon classificationPlantaeMagnolialesAnnonaceae

R.E.Fr.

Fig. 35 , Map 3

Cremastosperma pacificum R.E.Fr., Ark. Bot. 1: 330. 1950.

#### Type.

COLOMBIA, Valle del Cauca: Costa del Pacífico, Río Cajambre, Silva, 5–80 m a.s.l., 5–15 May 1944, *Cuatrecasas, J. 17463* (holotype: S! [two sheets S10-19876, S10-19873]; isotypes: F! [V0074281F, V0047735F], US! [US00104268, US00104267]), VALLE! [VALLE000049].

#### Description.

*Tree* 3–15 m tall, 2.5–25 cm diam.; young twigs and petioles sparsely to rather densely covered with appressed golden hairs to 0.4 mm long or glabrous. *Leaves*: petioles 8–16 by 2–4 mm; lamina elliptic to obovate, or narrowly so, 19–41 by 9–16 cm (index 2.1–3.1), chartaceous to slightly coriaceous, brown, brownish-green, or greyish-green and shiny above, brown, pale brown or greenish-brown below, glabrous above, glabrous or sparsely to densely covered with appressed golden hairs to 0.4 mm long particularly on veins below, base acute, apex acuminate (acumen 10–20 mm long), primary vein shallowly grooved at base, 2–3 mm wide at widest point, secondary veins 7–12, intersecondary veins occasionally 1–2, distance between from 8 mm at the base to up to 55 mm closer to the apex, angles with primary vein from 30–50° at the base to 50–70° closer to the apex, not branching, often forming distinct loops for the apical third, smallest distance between loops and margin 1.5–2 mm, tertiary veins percurrent. *Inflorescence* of single flowers, on leafless twigs; peduncles ca. 1 by 1 mm (in flower), 2–3 by ca. 2 mm (in fruit), rather densely to densely covered with appressed golden or whitish hairs to 0.2 mm long; pedicels 12–20 by ca. 1 mm (in flower), 22–35 by 1.5 mm (in fruit), longitudinally furrowed, glabrous or sparsely to rather densely covered with appressed gold or whitish hairs to 0.2 mm long; single lower bract, deltate, 1–1.5 by 1 mm, obtuse or acute, occasionally persistent, densely covered with appressed gold or whitish hairs 0.2 mm long; upper bract in the lower half of the pedicel, deltate, 1–1.5 by 1–1.5 mm, rounded, outer side sparsely to rather densely covered with appressed gold or whitish hairs to 0.2 mm long or glabrous; closed flower buds not seen; flowers pale greenish-yellow or pale green *in vivo*, black or dark brown *in sicco*, sepals and petals glabrous; sepals free, very broadly ovate, 3 by 3–3.5 mm, obtuse, soon falling off; outer petals elliptic to broadly elliptic, ca. 16 by 11–12 mm, inner petals obovate, ca. 15 by 7 mm, obtuse; stamens ca. 1.5 mm long, connective appendage ca. 0.6 mm wide; gynoecium not seen. *Monocarps* (2–)7–21, ellipsoid, slightly asymmetrical, 15–18 by 10–12 mm, green (immature) *in vivo*, black or dark brown *in sicco*, with an excentric apicule, monocarps, stipes and receptacle glabrous; stipes 10–18 by 1 mm; fruiting receptacle depressed ovoid, 3–9 mm diam. *Seeds* ellipsoid, yellow, furrowed and slightly pitted, ca. 10 by 8 mm, raphe raised within sunken groove, regular.

#### Distribution.

Pacific coast of Colombia (Chocó, Valle del Cauca).

#### Habitat and ecology.

Tropical wet and pluvial forest, reported as growing on yellow clay with alluvial substrate. At elevations of 5–100 m. Flowering: December and August; fruiting: April and May.

#### Notes.

*Cremastospermapacificum* appears similar to a number of other species characterised by the absence of (visible) indument on flowers and fruits, most notably *C.magdalenae*, *C.megalophyllumC.osicola*, *C.panamense* and *C.yamayakatense*. The sepals of *C.magdalenae* and *C.megalophyllum* are much larger (4–7 mm long, as opposed to up to 3 mm in *C.pacificum*). From the limited floral material available, bud development in *C.pacificum* would not appear to be open, unlike the fully open bud development of *C.osicola* and *C.panamense* and the closed bud shape is similar to that of *C.yamayakatense*. In contrast to both *C.yamayakatense* and *C.panamense*, the monocarps of *C.pacificum* are relatively large (>15 mm long, as opposed to up to 14 mm). The monocarps of *C.pacificum* are around the same length as the stipes, as opposed to stipes longer than monocarps in *C.osicola*.

#### Preliminary conservation status.

*Cremastospermapacificum* is known from a small number of collections, but scattered across a fairly wide area. As with a number of other species with apparently widely disjunct distributions in extra-Amazonian Colombia (particularly *C.novogranatense* and *C.longipes*), it is possible that both EOO and AOO are currently underestimated compared to other species as a result of the lack of recent collections. Nevertheless, following a precautionary approach, until demonstrated otherwise, we consider *C.pacificum* Endangered [EN] (Table 1).

#### Selected specimens examined.

**COLOMBIA. Chocó**: Yuto-Lloró road, 2 km from Ferry, 7 Aug 1982, *D. Sánchez et al. 323* (U). **Valle del Cauca**: San Cipriano, Escalarete Natural Reserve, 100 m a.s.l., 26 Mar 1993, *Devia et al. 3762* (COL); Bajo Calima, 3°53'N, 77°10'W, 50 m a.s.l., 7 Jul 1987, *Faber-Langendoen 1208* (U); Bajo Calima, 3°36'N, 77°08'W, 50 m a.s.l., 4 Dec 1981, *Gentry 35298* (MO, U); Bajo Calima, 3°56'N, 77°08'W, 50 m a.s.l., 10 Dec 1981, *Gentry 35562* (MO, U).

**Figure 35.**
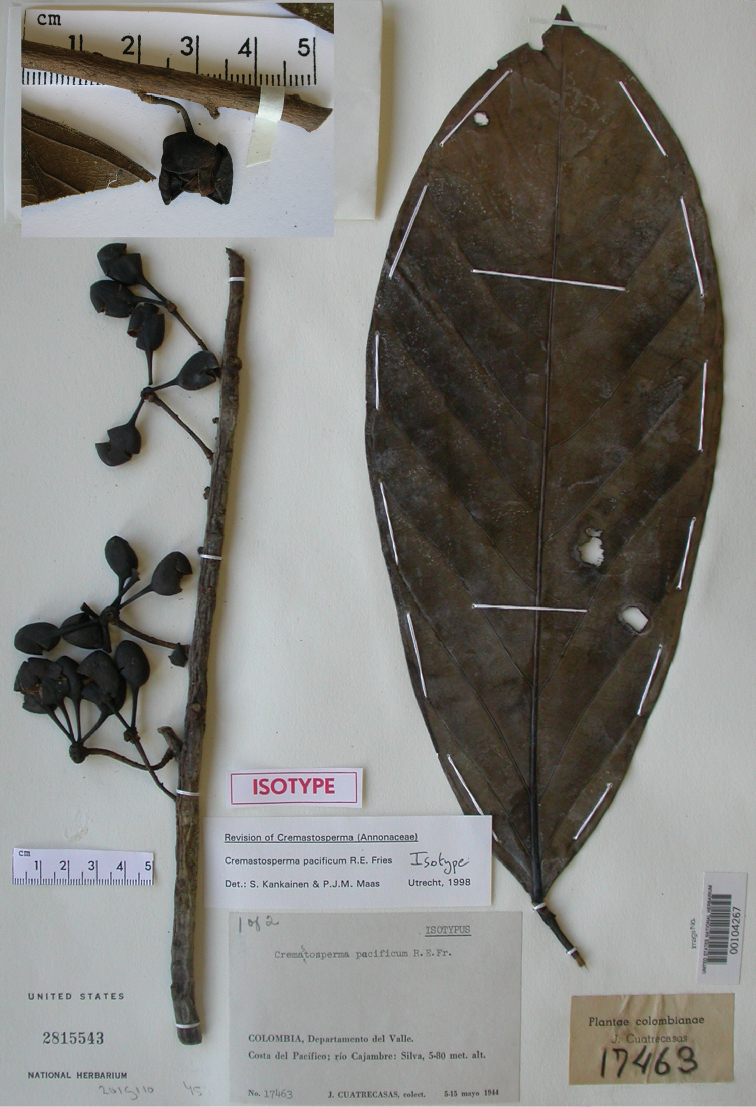
*Cremastospermapacificum* R.E.Fr. **a** fruiting specimen **b** flower (**a***Cuatrecasas 17463***b***Sánchez et al. 323*).

### 27. Cremastosperma panamense

Taxon classificationPlantaeMagnolialesAnnonaceae

Maas

**Figure 36.**
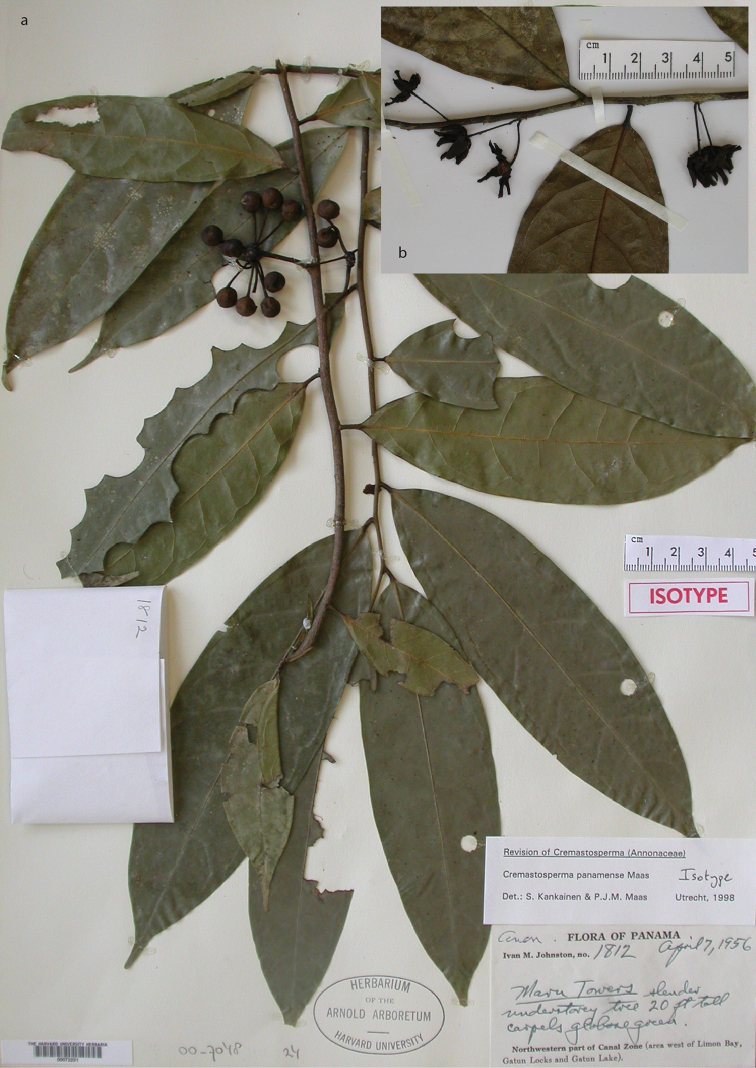
*Cremastospermapanamense* Maas. **a** fruiting specimen **b** flowers (**a***Johnston 1812***b***Perez 832*).

Figs 36a, b , 7 , Map 2

Cremastosperma panamense Maas, Proc. Kon. Ned. Akad. Wetensch. Ser. C 89: 254, f. 5 & 6.1986.

#### Type.

PANAMA. Canal Zone: NW part of Canal Zone, area W of Limon Bay, Gatun Locks and Gatun Lake, Maru Towers, 7 Apr 1956, *Johnston, I.M. 1812* (holotype: MO! [MO-047502]; isotypes: A! [00072231], MICH! [MICH1256197]).

#### Description.

*Tree* or *shrub* 1.5–7(–20) m tall, 3–10 cm diam.; young twigs and petioles glabrous. *Leaves*: petioles 2–10 by 1–2.5 mm, caniculate above, verrucose or furrowed; lamina narrowly elliptic to elliptic, 8–22 by 2–7 cm (index 2.7–4.7), chartaceous, green, brownish-green or greyish-green (or brown) above, (pale) green, (pale) brownish-green (or brown) below, shiny on both sides, glabrous on both sides, base acute to obtuse, decurrent, rarely narrowly cuneate, apex acuminate (acumen 5–25 mm long), primary vein occasionally shallowly grooved at the base, 1–1.5 mm wide at widest point, verrucose below, secondary veins 5–10, intersecondary veins 1–4, distance between from 5–20 mm at the base to 15–25 mm closer to the apex, angles with primary vein from 35–55(–70)° at the base to 60–75° closer to the apex, not branching, forming mostly distinct loops, smallest distance between loops and margin 2–5 mm, tertiary veins largely reticulate. *Inflorescence* of single, solitary flowers, on leafy or leafless twigs; peduncles 1–3 by 0.5–1 mm (in flower), 1–3 by 1.5–2 mm (in fruit), sparsely covered with erect whitish to golden hairs to 0.1 mm long; pedicels 12–20 by 0.5–0.8 mm at the base (in flower), 13–22 by 1–2 mm (in fruit), glabrous; (1–)2–3(-several) lower bract(s), deltate, ca. 0.5 by 0.5 mm, obtuse, mostly soon falling off, sparsely covered with erect whitish to golden hairs to 0.1 mm long; upper bract halfway along the pedicel, ovate to depressed ovate, 1–1.5 by 0.7–1mm, acute, obtuse or emarginate, glabrous; closed flower buds very broadly ovoid, opening in development; flowers green when immature, maturing to white, cream, or (pale) yellow *in vivo*, yellow brown, dark brown or blackish brown *in sicco*, sepals and petals glabrous; sepals free, broadly to very broadly ovate, appressed or patent, 1.5–3 by 1.5–3 mm, obtuse, soon falling off or persistent; outer petals narrowly ovate to narrowly elliptic, 7–18 by 4–6 mm, obtuse, inner petals narrowly ovate to narrowly elliptic, 10–25 by 3–5 mm, obtuse; androecium diam. unknown, stamens 1.2–1.5 mm long, connective appendage 0.7–0.9 mm wide; gynoecium diam. unknown, carpels ca. 35, 2 mm long, glabrous. *Monocarps* (2–)8–30, more or less globose, slightly asymmetrical, 8–13 by 7–11 mm, green maturing to yellow, orange, red or black *in vivo*, pale brown, reddish-brown or brown *in sicco*, with an excentric apicule; stipes 7–21 by 1–1.5(–3) mm; fruiting receptacle 3–8 mm diam., monocarps, stipes and receptacle glabrous. *Seeds* broadly ellipsoid, globose or transversally ellipsoid, light brown, pitted, 7–12 by 6–10 mm, raphe sunken, regular.

#### Distribution.

Panama (Coclé, Colón, Panamá and San Blas).

#### Habitat and ecology.

Evergreen tropical wet forest, cloud forest or low swampy places or in disturbed areas. At elevations of 0–800 m. Flowering: April, May, July and August; fruiting: throughout the year.

#### Vernacular names.

Panama: Palo santo (Panamanian name), Sate wawa (Kuna; *Nevers 4898*), Waras gid (Kuna; *Nevers 6513)*.

#### Notes.

*Cremastospermapanamense* appears similar to *C.magdalenae*, but can be distinguished by the smaller size of the sepals, which additionally persist less frequently into fruiting. *C.pacificum* and *C.chococola* are both geographically close (Pacific coast of Colombia) and share the characters of glabrous pedicels (flowers) and fruits. However, the pedicels of *C.chococola* are considerably longer and the shape of the larger monocarps of *C.pacificum* (ellipsoid as opposed to roughly globose), as well as the larger leaves, allow easy distinction in both cases. The colour of the relatively small leaves (drying consistently green), the relatively large distance between the loops of their secondary veins and the margin and the reticulate and indistinct nature of tertiary and quaternary venation of *C.panamense* are also distinctive.

#### Preliminary conservation status.

*Cremastospermapanamense* has been described as common in the San Blas region of Panama. It has been collected fairly frequently near roads in the surrounding region, but not within protected areas and with an EOO that would be sufficiently small as to qualify as Vulnerable. Given its apparent local abundance but restricted distribution: Near Threatened [NT] (Table 1).

#### Selected specimens examined.

**PANAMA. Coclé**: Caño Blanco del Norte, Cano Sucio, 8°42'N, 80°36'W, 200–400 m a.s.l., 3 Oct 1983, *Davidse et al. 23634* (MO). **Colón**: Santa Rita Ridge road, 9°20'N, 79°46'W, 300 m a.s.l., 16 Jun 1994, *Galdames et al. 1162* (US); Parque Nacional Lorenzo, road to Sherman Crane, 9°16'N, 79°58'W, 150 m a.s.l., 7 Jun 2004, *Maas et al. 9559* (U); Teck Cominco Petaquilla mining concession, 8°50'58"N, 80°38'53"W, 180 m a.s.l., 25 Jun 2008, *McPherson 20670* (WAG). **Panama**: El Llano-Cartí road, 9°15'N, 79°00'W, 350 m a.s.l., 22 Aug 1986, *McPherson 9957* (MO, U). **San Blas**: Mountain range opposite Nargana island, 9°22'N, 78°34'W, 65 m a.s.l., 12 Aug 1994, *Galdames et al. 1585* (NY); Yannuadi, 9°22'N, 78°35'W, 50–100 m a.s.l., 23 Oct 1992, *H. Herrera et al. 1231* (MO); Nusagandi, Sendero Wedar, 9°18'N, 78°58'W, 300–400 m a.s.l., 19 Jul 1985, *McDonagh et al. 181* (MO); Nusagandi, 9°19'N, 78°15'W, 300 m a.s.l., 31 Jul 1984, *De Nevers et al. 3601* (MO); Lower Río Cangandí, 9°27'N, 79°08'W, 0–20 m a.s.l., 14 Jan 1985, *De Nevers* & *H. Herrera 4571* (MO, U); Río Cangandi, 9°24'N, 79°24'W, 0–100 m a.s.l., 17 Feb 1985, *De Nevers et al. 4898* (MO, U).

**Figure 37.**
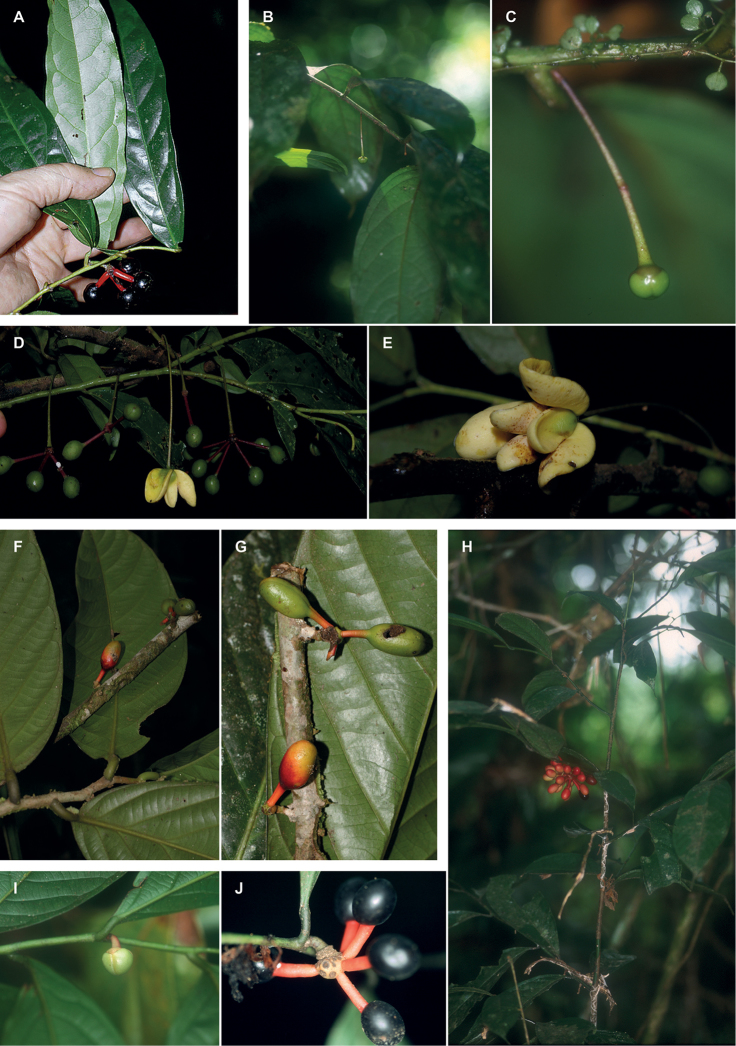
**a***Cremastospermapanamense* Maas. Fruiting specimen (photo: Robin Foster) **b–e***C.pendulum* (Ruiz & Pav.) R.E.Fr. **b** habit **c** flower bud (Pirie et al. 33; photos MDP) **d** flowering and fruiting branch **e** flower (*Vásquez et al. 34206*, photos Rodolfo Vásquez) **f, g***C.westrae* Pirie fruiting specimen (photos Robin Foster) **h–j***C.yamayakatense* Pirie **h** habit, fruiting specimen (*Pirie et al. 57*) **i** flower bud (*Pirie et al. 58*) **j** mature fruit (*Pirie et al. 60*; photos MDP).

### 28. Cremastosperma pedunculatum

Taxon classificationPlantaeMagnolialesAnnonaceae

(Diels) R.E.Fr.

Fig. 38 , Map 7

Cremastosperma pedunculatum (Diels) R.E.Fr., Acta Horti Bergiani 10: 48. 1930.
Aberemoa pedunculata Diels, Bot. Jahrb. Syst. 37: 409. 1906.

#### Type.

PERU, San Martín: Moyobamba, 1100–1200 m a.s.l., 1906, *Weberbauer, A. 4558* (holotype: B! [B 10 0242366]; isotypes: F, G! [G00237252], S! [S-R-7025]).

#### Description.

*Tree* 4–15 m tall, 8–15 cm diam.; young twigs and petioles glabrous. *Leaves*: petioles 4–15 by 1–3 mm; lamina elliptic to obovate or narrowly so, 12–27 by 4–10 cm (index 2–3.6), chartaceous, (dark) greyish-brown above, blackish-brown with darker veins below, glabrous above, glabrous to sparsely covered with appressed golden hairs to 0.6 mm long, particularly on veins below, base acute to rounded, decurrent, apex acuminate (acumen 5–20 mm long), primary vein 1.5–2.5 mm wide at widest point, secondary veins 7–13, intersecondary veins often 1–3, distance between from 5 mm at the base to 15–20 mm closer to the apex, angles with primary vein inconsistent, 40–60° at the base and closer to the apex, not branching, forming distinct loops, smallest distance between loops and margin 1–4 mm, tertiary veins more or less percurrent. *Inflorescence* of single flowers clustered in groups of up to 2, on leafy or leafless twigs or main trunk; peduncles 2–10 by ca. 1 mm (in flower), 5–15 by ca. 1.5 mm (in fruit), sparsely to rather densely covered with appressed golden or whitish hairs 0.1–0.4 mm long; pedicels (30–)35–75(–95) by ca. 1 mm at the base, to 3 mm at the apex (in flower), (30–)55–85(–110) by 1–1.5 mm at the base, to 3 mm at the apex (in fruit), sparsely to rather densely (at the base) covered with appressed golden or whitish hairs 0.1- 0.4 mm long or glabrous; 1-several lower bracts, elliptic, ca. 1.5 by 1 mm, acute, soon falling off, sparsely to rather densely covered with appressed golden or whitish hairs 0.1- 0.4 mm long; upper bract attached in variable position on pedicel, ovate to very broadly ovate, 1–2.5 by 1–2 mm, acute, obtuse or rounded, sparsely to rather densely covered with appressed golden or whitish hairs 0.1- 0.4 mm long or glabrous; closed flower buds very broadly ovoid to globose, opening loosely in development; flowers green, maturing to green-violet, yellow or pale cream-yellow, inner petals with purple base *in vivo*, dark brown or reddish-brown *in sicco*, sepals and petals glabrous; sepals free or connate for ca. 1 mm, broadly to very broadly ovate or broadly ovate-triangular, appressed, patent or recurved, 2.5–4 by 2.5–4 mm, obtuse, mostly persistent; outer petals elliptic to broadly elliptic, 11–17 by 7–13 mm, inner petals elliptic, obovate or narrowly so, 11–19 by 4–8 mm, obtuse or rounded, petals with prominent venation; androecium ca. 7 mm diam., stamens 1.3–1.8 mm long, connective appendage 0.5–0.8 mm wide; gynoecium ca. 1.5 mm diam., carpels 2–2.2 mm long. *Monocarps* 3–27, ellipsoid to broadly ellipsoid, asymmetrical, 12–17 by 10–12 mm, green (immature) *in vivo*, black, dark brown or reddish-brown *in sicco*, with an excentric apicule; stipes 11–21 by 1.5–2 mm; fruiting receptacle 4–10 mm diam.; monocarps, stipes and receptacle glabrous. *Seeds* ellipsoid to broadly ellipsoid, reddish-brown, pitted, ca. 10 by 7 mm, raphe sunken, regular.

#### Distribution.

Ecuador (Zamora-Chinchipe), Peru (San Martín, Cajamarca). Two collections of less certain affinity have been made further north in Ecuador (Pastaza, Morona-Santiago) and one in Colombia (Caquetá).

#### Habitat and ecology.

Premontane and montane primary and secondary forest, sometimes inundated, mainly on soils with calcareous bedrock. At elevations of 850–1800 m (except the single specimen collected in Pastaza (Ecuador) at 360 m). Flowering: July, October-December; fruiting February, July, October and December.

#### Notes.

The pedicels of *Cremastospermapedunculatum* are unusually long in the genus, similar to those of *C.bullatum* (distinguished by the bullate appearance of the leaves and dense, long, indument on most parts) and only exceeded by *C.longipes* (from the western side of the Andes and with larger leaves) and *C.dolichopodum* (from further south in Peru). *Cremastospermapedunculatum* is otherwise similar to *C.alticola*, which also occurs at higher elevations in northern Peru and Ecuador (albeit much less frequently collected), differing from *C.alticola* by the presence of indument on the (longer) pedicels, lack of branching inflorescences and stipes as long as or longer than monocarps.

The holotype of *Guatteriasocialis* J.F.Macbr., *C. Schunke 395*, was determined by Diels (1931) as *C.pedunculatum* although he deliberately omitted placing *G.socialis* in synonymy under *C.pedunculatum*, citing differences in the reported growth form. The type is reported by the collector to be a liana. This has not been recorded for any other collections of *C.pedunculatum* (although it is reported for the type specimen of *C.oblongum*). In addition, the collection was made in the central Peruvian department of Junin, much further south than the known distribution of *C.pedunculatum* (in northern Peru and Ecuador). A photo of the holotype (not the specimen itself) was made available to the authors. The collection appears to be of a *Cremastosperma* (the leaves with a raised primary vein), but includes only immature buds. Although Maas et al. (Maas, Mennega & Westra 1994; Maas et al. 2015) have listed *G.socialis* as a taxonomic synonym of *C.pedunculatum*, we consider the available evidence insufficient to assign this specimen to a particular species in *Cremastosperma*.

#### Preliminary conservation status.

*Cremastospermapedunculatum* occurs over a relatively wide area including within protected areas in Ecuador. Least concern [LC] (Table 1).

#### Selected specimens examined.

**COLOMBIA. Caquetá**: Florencia, 1°36'N, 75°37'W, 1400–1440 m a.s.l., 10 Nov 1993, *J.G. Ramírez et al. 4881* (MO). **ECUADOR. Morona-Santiago**: Cordillera de Huaracayo, Río Coangos, 3°15'44"S, 78°12'01"W, 1380 m a.s.l., 25 Mar 2001, *Neill* & *Manzanares 13201* (U); E of Macas, 2°21'S, 77°59'W, 700–1400 m a.s.l., 24 Aug 1996, *Stahl et al. 2931* (AAU). **Pastaza**: Río Acaro, 1°23'S, 77°25'W, 360 m a.s.l., 19 Jan 1998, *Neill et al. 11065* (AAU, MO, QCNE, WAG). **Santiago-Zamora**: without precise locality, 2°40'S, 78°00'W, 450–550 m a.s.l., 17 Nov 1944, *Camp E 1311* (NY). **Zamora-Chinchipe**: San Francisco Scientific Station, 3°58'S, 79°04'W, 1900 m a.s.l., 21 Nov 1998, *X. Cornejo* & *Bonifaz 6702* (AAU, U); Nangaritza, 4°15'S, 78°39'W, 1000 m a.s.l., 12 Mar 2017, *X. Cornejo et al. 9000* (L); Río Nangaritza, Shaimi, 4°19'S, 78°40'W, 985 m a.s.l., 6 Nov 2004, *Homeier et al. 1489* (MO); Parque Nacional Podocarpus, Bombuscaro entrance, 4°07'S, 78°58'W, 1050 m a.s.l., 27 Jan 2009, *Homeier et al. 4173* (WAG); Río Nangaritza, 4°20'S, 78°40'W, 1000 m a.s.l., 7 Dec 1990, *Neill 9589* (MO, QCNE, U); Río Nangaritza, Miazi, 4°16'S, 78°42'W, 930 m a.s.l., 26 Oct 1991, *Palacios et al. 8646* (COL, F, QCNE, U); Cordillera del Condór, 4°16'54"S, 78°36'00"W, 900 m a.s.l., 27 Jul 2003, *Quizhpe et al. 671* (MO, U); Parque Nacional Podocarpus, 4°39'50"S, 79°07'00"W, 1050–1250 m a.s.l., 12 Jul 1990, *Rome et al. 1014* (MO, QCNE, U). **PERU. Cajamarca**: San Ignacio, 5°18'30"S, 78°43'00"W, 1350 m a.s.l., 23 Jul 1997, *Campos et al. 4268* (USM); Distr. Huarango, Caserio Nuevo Mundo, 5°10'S, 78°32'W, 1500–1600 m a.s.l., 21 Jul 1997, *E. Rodríguez* & *P. Reyes 1758* (F, HUT, U). **San Martín**: Prov. Ríoja, Venceremos, Rioja-Pomacocha road, 5°45'S, 77°40'W, 1850 m a.s.l., 11 Feb 1984, *Gentry et al. 45355* (MO); Prov. Ríoja, Pedro Ruíz-Moyobamba road, 5°50'S, 77°45'W, 1770–2150 m a.s.l., 5 Aug 1983, *D.N. Smith* & *S. Vasquez 4611* (MO, U); Prov. Ríoja (locality unknown), 6°08'S, 77°18'W, 1500–1640 m a.s.l., 2 Jul 1998, *I. Sánchez et al. 9615* (F).

**Figure 38.**
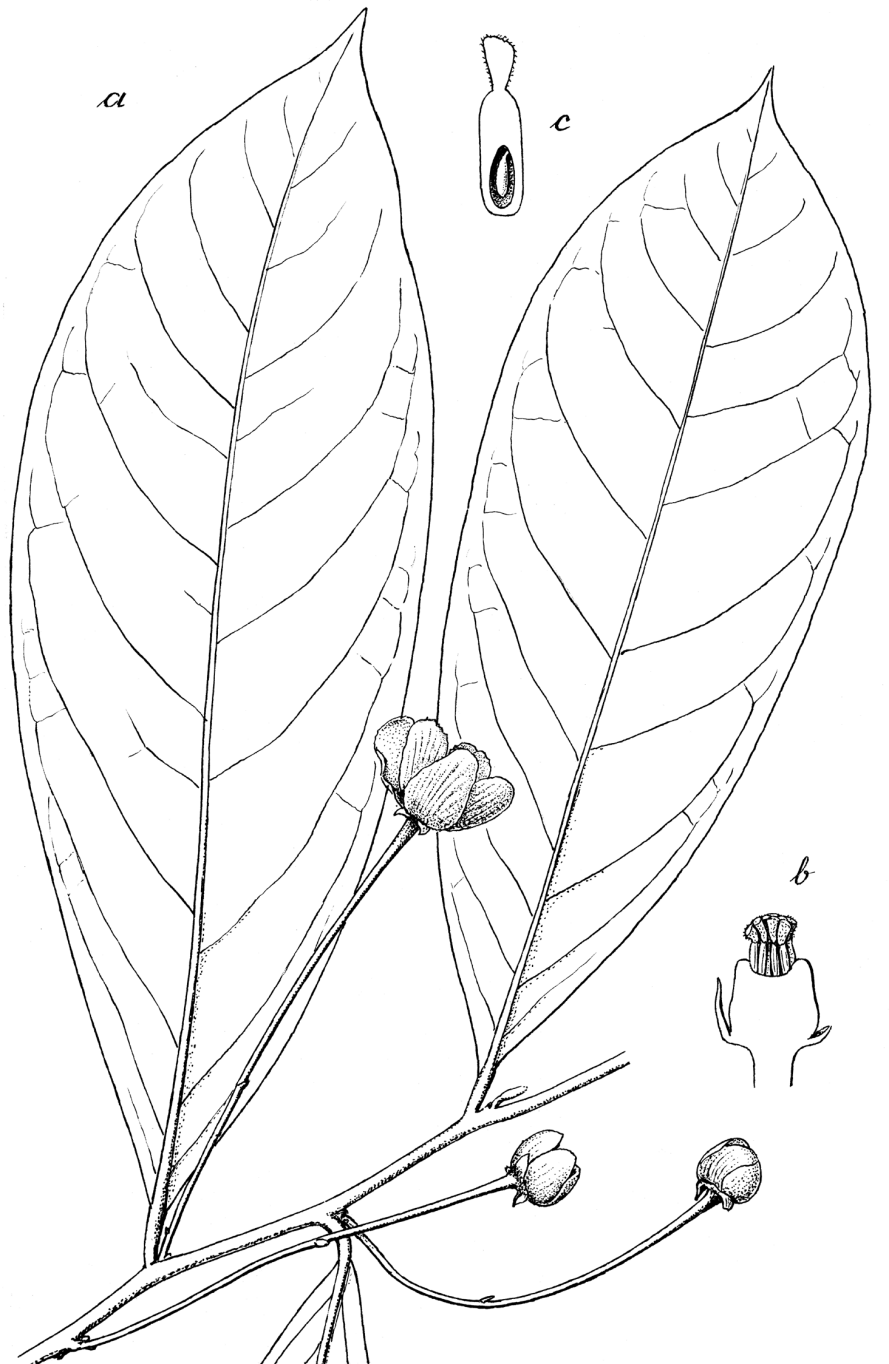
*Cremastospermapedunculatum* (Diels) R.E.Fr. **a** flowering twig **b** receptacle, illustrating insertion of carpels **c** carpel with single apical/lateral ovule (**a–c***Weberbauer 4558*, reproduced from Fries (1930).

### 29. Cremastosperma pendulum

Taxon classificationPlantaeMagnolialesAnnonaceae

(Ruiz & Pav.) R.E.Fr.

**Map 8.**
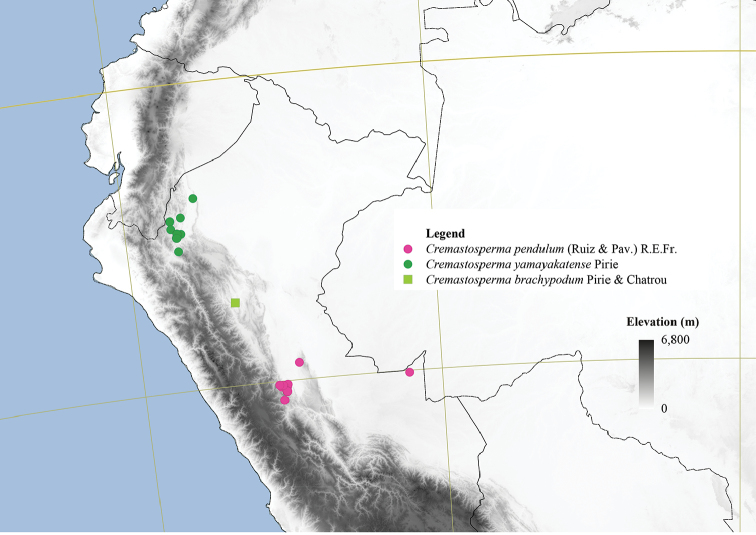
Distribution of *Cremastospermabrachypodum* Pirie & Chatrou, *C.pendulum* (Ruiz & Pav.) R.E.Fr.; and *C.yamayakatense* Pirie.

**Figure 39.**
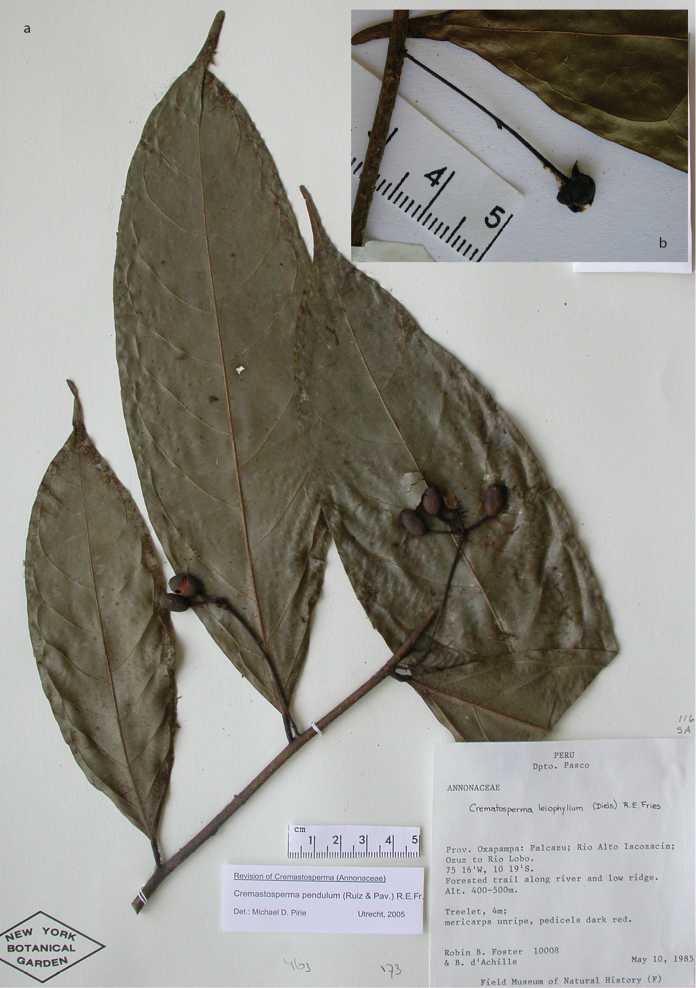
*Cremastospermapendulum* (Ruiz & Pav.) R.E.Fr. **a** fruiting specimen **b** flower bud (**a***Foster & d’Achille 10008***b***Foster 9842*).

Figs 36 , 39 , Map 8

Cremastosperma pendulum (Ruiz & Pav.) R.E.Fr., Acta Horti Bergiani 10: 325. 1931.
Guatteria pendula Ruiz & Pav., Syst. Veg. Fl. Peruv. Chil.: 146. 1798.

#### Type.

PERU, without location, without date, *Pavón, J.A. s.n.* (holotype: G! [G00237253]).

#### Description.

*Tree* or *shrub* 4–10 m tall; young twigs and petioles glabrous. *Leaves*: petioles (3–)6–13 by 1–3 mm; lamina narrowly elliptic to elliptic, 7–30 by 3.5–10 cm (index 2.9–4.2), chartaceous, drying green (darker above), glabrous above, sparsely covered with appressed whitish hairs to 0.2 mm long particularly on veins or glabrous below, base acute, apex acuminate (acumen 15–25 mm long), primary vein 1–2 mm wide at widest point, deeply grooved in basal half, secondary veins 7–10, intersecondary veins often 1(–2), distance between from 9–16 mm at the base to 11–25 mm closer to the apex, angles with primary vein from 50–60° at the base to 45–50° closer to the apex, sometimes branching, forming distinct loops, smallest distance between loops and margin 2–4 mm, tertiary veins more or less percurrent. *Inflorescence* of single, solitary flowers; peduncles 0.5–1.5 by ca. 0.5 mm (in flower), 3–5 by 0.8–1 mm (in fruit), sparsely covered with appressed whitish hairs <0.1 mm long or glabrous; pedicels 28–70 by 0.3–0.5 mm at the base, to 1 mm at the apex (in flower), 50–70 by 0.5–1 mm at the base, to 1.5 mm at the apex (in fruit), green or purple *in vivo*, glabrous; 1 or 2 lower bracts, deltate, ca. 0.5 by 0.5 mm, acute, soon falling off, rather densely covered with appressed whitish hairs <0.1 mm long; upper bract attached halfway along pedicel, elliptic or deltate, 0.5–1 by 0.5–1 mm, acute or obtuse, sparsely covered with appressed whitish hairs <0.1 mm long or glabrous; closed flower buds depressed ovoid, remaining closed (or nearly so) throughout development; flowers green maturing to yellow *in vivo*, black *in sicco*, sepals and petals glabrous; sepals fused for basal 0.5 mm, elliptic, appressed (particularly in bud) or recurved, ca. 2 by 1.5 mm, obtuse, soon falling off; outer petals ovate, 5–6 by 3–4 mm, inner petals ovate, ca. 3.5 by 2 mm; androecium ca. 4 mm diam., connective appendage 0.5–0.8 mm wide; gynoecium ca. 1 mm diam., glabrous. *Monocarps* 6–11, ellipsoid, slightly asymmetrical, 10–13 by 7–9 mm, monocarps and stipes green maturing to dark reddish-brown *in vivo*, medium to dark brown *in sicco*, with an excentric apicule; stipes 9–15 by 1–2 mm; fruiting receptacle depressed ovoid, 3–6 mm diam., monocarps, stipes and receptacle glabrous. *Seeds* ellipsoid, reddish-brown, pitted, ca. 13 by 7 mm, raphe sunken, regular.

#### Distribution.

Peru (Huánuco, Pasco, Ucayali).

#### Habitat and ecology.

Primary and secondary tropical lowland and upland forest, on brown and red latosols. At elevations of 180–500 m. Flowering: January, August, September and November; fruiting: May, July and August.

#### Vernacular names.

Peru: Tortuga (*McDaniel et al. 2570*).

#### Notes.

*Cremastospermapendulum* can be distinguished by its long thin pedicel in combination with the shape of the small flower buds which remain closed throughout development: it differs from *C.yamayakatense* in the greater length of pedicel and from *C.monospermum* in the depressed ovoid rather than triangular flower bud.

#### Preliminary conservation status.

*Cremastospermapendulum* has an EOO that exceeds the minimum for Vulnerable status, but a low AOO representing relatively sparse distribution across the range (albeit some in protected areas). Near Threatened [NT] (Table 1).

#### Selected specimens examined.

**PERU. Huánuco**: Pucallpa, western Sira mountain, 9°27'S, 74°46'W, 500 m a.s.l., 9 Aug 1988, *Morawetz* & *Wallnöfer 13 9888* (U). **Pasco**: Palcazú, Iscozacin, 10°12'S, 75°15'W, 380 m a.s.l., 24 Jan 1984, *R.B. Foster 9482* (F, MO, NY, U, USM); Palcazú, Río Iscozacin, Ozuz to Río Lobo, 10°19'S, 75°16'W, 400–500 m a.s.l., 10 May 1985, *R.B. Foster* & *Achille 10008* (F, LPB, MOL, U, USM); Parque Nacional Yanachaga-Chemillen, 10°19'20"S, 75°15'56"W, 368 m a.s.l., 8 Nov 2003, *Pirie et al. 33* (HOXA, MOL, U, USM); Palcazú, Ataz-Quebrada Ataz, 10°10'13"S, 75°18'55"W, 26 May 2009, *Valenzuela et al. 13014* (HOXA, HUT, MO, MOL, USM); Palcazú, Ataz-Quebreda Ataz, 10°10'10"S, 75°18'55"W, 392 m a.s.l., 26 May 2009, *Valenzuela et al. 13016* (HUT, MO, MOL, USM); Palcazú, Bosque de Protección San Matias, 10°36'40"S, 75°22'29"W, 1902 m a.s.l., 13 Oct 2008, *Vásquez 34206* (MO, WAG); San Francisco de Chuchurros, 10°07'S, 75°13'W, 300 m a.s.l., 6 Jul 2003, *Van der Werff et al. 18081* (U). **Ucayali**: Prov. Purus, Distr. Purus, Río Caranja, 10°04'S, 71°06'W, 325 m a.s.l., 22 Jul 1998, *Graham 635* (F, U).

### 30. Cremastosperma peruvianum

Taxon classificationPlantaeMagnolialesAnnonaceae

R.E.Fr.

Fig. 40 , Map 7

Cremastosperma peruvianum R.E.Fr., Acta Horti Bergiani 12: 204. 1934.

#### Type.

PERU, Loreto: upper Río Marañon, Pongo de Manseriche, 160 m a.s.l., 1 Oct 1924, *Tessmann. G. 4176* (holotype: B! [B 10 0242364]; isotype: S! [S-R-6968]).

#### Description.

*Tree* 2–7 m tall; young twigs and petioles glabrous. *Leaves*: petioles 3–15 by 4–8 mm, verrucose or transversally furrowed; lamina elliptic to obovate or narrowly so, 38–64 by 11–17(–24) cm (index 2.7–4.4), chartaceous or coriaceous, pale olive or brownish-green on both sides, glabrous, base obtuse, rounded or cordate, often asymmetrical, apex acuminate (acumen 15–35 mm long), primary vein deeply grooved in basal half, 3–5 mm wide at widest point, glabrous, secondary veins 20–26, intersecondary veins 1–2, distance between from 2–10 mm at the base, 20–40 mm in the centre, 10–20 mm closer to the apex, angles with primary vein from 55–65° at the base to 70–80° closer to the apex, not branching, forming mostly distinct loops, smallest distance between loops and margin 2–4 mm, tertiary veins percurrent. *Inflorescence* of single solitary flowers, on leafy or leafless twigs; short axillary shoot, 1–1.5 by 1–1.5 mm (in flower), ca. 3 by ca. 2.5 mm (in fruit), sparsely covered with appressed or erect golden hairs <0.1 mm long or glabrous; pedicels 18–20 by ca. 1 mm at the base (in flower), 20–25 by ca. 2 mm (in fruit), red *in vivo*, glabrous; 2 lower bracts, elliptic, ca. 1 by 0.5 mm, obtuse, soon falling off, sparsely covered with appressed golden hairs <0.1 mm long; upper bract attached near the base of the pedicel, (broadly) ovate, 1.5–3 by ca. 1.5 mm, rounded, glabrous; closed flower buds broadly ovoid-triangular, remaining loosely closed in development; flowers green, maturing to bright yellow (green at the base) outside, brown inside *in vivo*, orange or yellowish-brown (sepals and bracts lighter) *in sicco*, sepals and petals glabrous; sepals free, broadly ovate, appressed, ca. 4 by 3.5 mm, obtuse, soon falling off; outer petals elliptic to narrowly obovate, 15–29 by 8–13 mm, obtuse, inner petals narrowly elliptic to narrowly obovate, (13-)21–25 by 4–8 mm; androecium diam. unknown, stamens 1.5–2 mm long, connective appendage 0.6–0.8 mm wide; gynoecium diam. unknown, carpels ca. 2 mm long. *Monocarps* 3–20, ellipsoid, asymmetrical, 16–19 by 12–13 mm, green maturing to yellow, purple or black *in vivo*, reddish-brown, dark brown or black *in sicco*, without apparent apicule; stipes 20–40 by 2 mm; fruiting receptacle 4–9 mm diam.; monocarps, stipes and receptacle glabrous. *Seeds* broadly ellipsoid, reddish-brown, pitted, ca. 11 by 10 mm, raphe sunken, regular.

#### Distribution.

Peru (Amazonas, Loreto).

#### Habitat and ecology.

Primary, non-inundated forest, on white sand or red clay. At elevations of 170–400 m. Flowering: September and October; fruiting: April, June – August and October.

#### Vernacular names.

Peru: Achuana (*Kayap 631*), Chiwanim (*Ancuash 262, 1517, Berlin 2079, Knapp et al. 7645*), Yáis (*Huashikat, V 577*).

#### Notes.

*Cremastospermaperuvianum* can be distinguished from other species of the genus by its long, relatively narrow leaves (often with cordate base) and long stipes. The flower resembles that of *C.monospermum* in shape, but is larger, with relatively larger sepals and borne on a shorter, thicker pedicel.

#### Preliminary conservation status.

*Cremastospermaperuvianum* is uncommon within a relatively small range, not within protected areas. Vulnerable [VU] (Table 1).

#### Selected specimens examined.

**PERU. Amazonas**: Río Cenepa, Huampami, 4°30'S, 78°30'W, 200–250 m a.s.l., 15 Aug 1978, *Ancuash 1517* (U); Distr. Santiago, 4°00'56"S, 77°35'19"W, 275 m a.s.l., 10 Aug 2011, *Huamantupa et al. 15601* (L, MO); Río Santiago valley, Quebrada Caterpiza, 200 m a.s.l., 4 Sep 1979, *Huashikat 357* (MO); Bagua, Putuim, 4°55'S, 78°19'W, 480 m a.s.l., 19 Jun 1996, *E. Rodríguez et al. 1112* (HUT, U, USM); Río Cenepa region, community Aguaruna Pagki-Suwa, 4°31'35"S, 78°10'34"W, 289 m a.s.l., 24 Jan 1997, *E. Rodríguez et al. 22253* (HUT); Río Comaina, 4°23'S, 78°21'W, 800 m a.s.l., 21 Aug 1994, *Vásquez et al. 18990* (U). **Loreto**: Yanayacu, 6 km W of Sarameriza, Río Marañón, 4°45'S, 77°20'W, 170 m a.s.l., 8 Jun 1986, *Knapp et al. 7645* (U).

**Figure 40.**
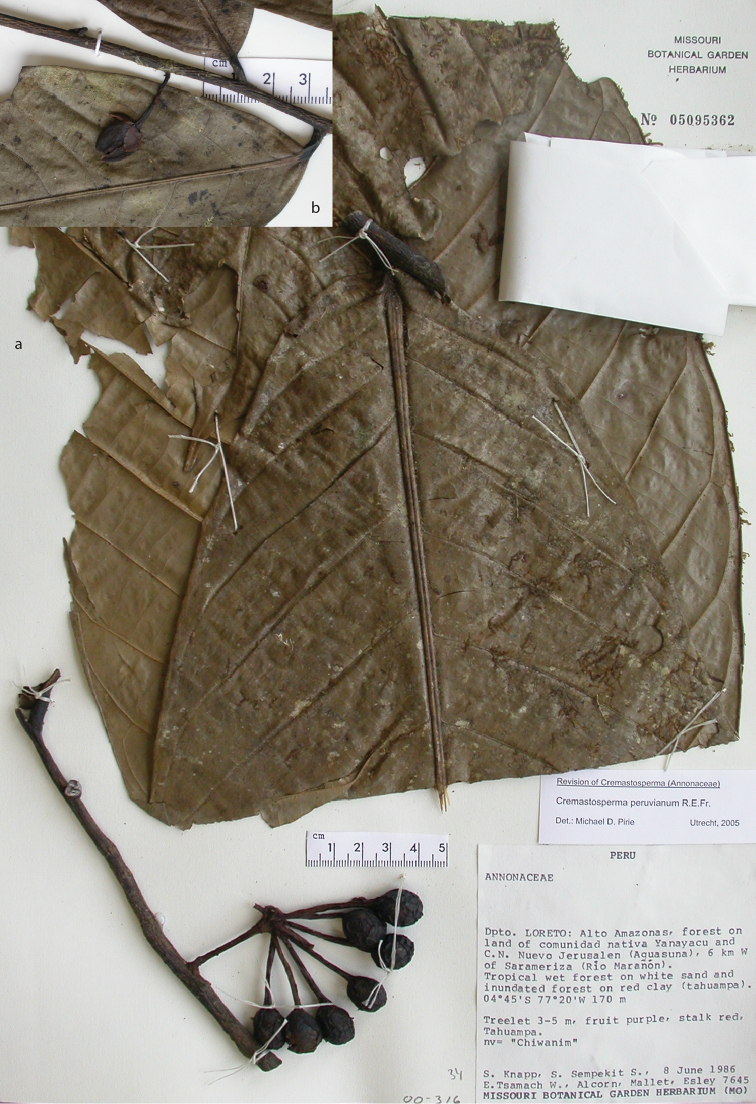
*Cremastospermaperuvianum* R.E.Fr. **a** fruiting specimen **b** flower bud (**a***Knapp et al. 7645***b***Díaz et al. 8225*).

### 31. Cremastosperma stenophyllum

Taxon classificationPlantaeMagnolialesAnnonaceae

Pirie

Fig. 41 , Map 2

Cremastosperma stenophyllum Pirie, Blumea 50: 56, f. 8. 2005.

#### Type.

ECUADOR, Pichincha: ‘Tinalandia’, km 112 on the road to Santo Domingo de los Colorados from Quito, 500–1000 m a.s.l., 15 Jan 1984, *Knapp, S. & Mallet, J. 6159* (holotype: QCNE [QCNE4139]; isotype: QCA! [QCA5625]).

#### Description.

*Tree* 5–10 m tall, up to 20 cm diam.; young twigs and petioles rather densely covered with appressed golden hairs to 0.2 mm long. *Leaves*: petioles 5–12 by 2–2.5 mm; lamina narrowly elliptic to elliptic, 25–33 by 6.5–11.5 cm (index 2.9–4.3), chartaceous, greyish-green above, green below, sparsely covered with appressed yellowish-white hairs to 0.2 mm long below and on veins above, base acute, apex acuminate (acumen 20–25 mm long), primary vein 1–1.5 mm wide at widest point, verrucose, secondary veins 8–10, intersecondary veins occasional, distance between from 5 mm at the base to 50 mm closer to the apex, angles with primary vein from 45–55° at the base to 70–80° closer to the apex, not branching, not forming loops, tertiary veins with some reticulation. *Inflorescence* of single flowers on leafy twigs or on brachyblasts on thicker twigs or branches; peduncle 1.5–3 by ca. 1 mm (in flower), ca. 6 by ca. 2 mm (in fruit); pedicels 45–55 by ca. 1 mm (in flower), ca. 62 by ca. 1.5 mm (in fruit), peduncles and pedicels and outer side of bracts (densely), sepals (densely) and petals (sparsely to rather densely) covered with appressed yellowish-white hairs to 0.2 mm long; 2 lower bracts, deltate, ca. 1 mm long, obtuse; upper bract attached on basal half of pedicel, ovate, ca. 1.5 by 0.8 mm, acute; closed flower buds not seen; flowers green, maturing to yellow *in vivo*, light brown with dark brown patches at the base of the petals *in sicco*; sepals deltate, 2 mm long, obtuse; outer petals elliptic, ca. 18 by 8 mm, inner petals elliptic, ca. 18 mm long (diam. unknown); androecium and gynoecium not seen. *Monocarps* ca. 4, ellipsoid, slightly asymmetrical, ca. 25 by ca. 12 mm, black *in sicco*, with a strongly excentric apicule; stipes ca. 8 by ca. 2.5 mm; fruiting receptacle ca. 4 mm diam.; monocarps, stipes and receptacle with occasional appressed white hairs <0.1 mm long. *Seeds* not seen.

#### Distribution.

Ecuador (Bolívar, Pichincha).

#### Habitat and ecology.

Secondary vegetation with primary elements. At elevations of 500–1200 m. Flowering: January; fruiting: not recorded.

#### Vernacular names.

Ecuador: Molinillo (*Acosta Solís 6429*).

#### Notes.

Only three collections of *Cremastospermastenophyllum*, one of which is sterile, have been observed by the authors. However, these are consistently distinct from all other species of the genus. *C.stenophyllum* can be distinguished even when sterile by the narrowly (or nearly so) elliptic long acuminate leaves. The flowers and fruits resemble those of *C.awaense* Pirie, but both the pedicel and leaf acumen are longer and *C.stenophyllum* also lacks the distinctive pattern of indument on the petals of *C.awaense*: the hairs are instead evenly distributed on the outer surface.

#### Conservation status.

We have seen one further specimen since *Cremastospermastenophyllum* was described, bringing the total to three, representing highly fragmented populations, none of which is in a protected area. Endangered [EN] (Table 1).

#### Other specimens examined.

**ECUADOR. Bolívar**: Valle de Limón, 800–1200 m a.s.l., 16 Oct 1943, *Acosta Solís 6429* (F); **Esmeraldas**: Quinindé Cantón, Cristobal Colón, 0°27'N, 79°09'W, 700 m a.s.l., 20 Oct 2008, *Palacios 16384* (ECUAMZ, MO, QCNE).

**Figure 41.**
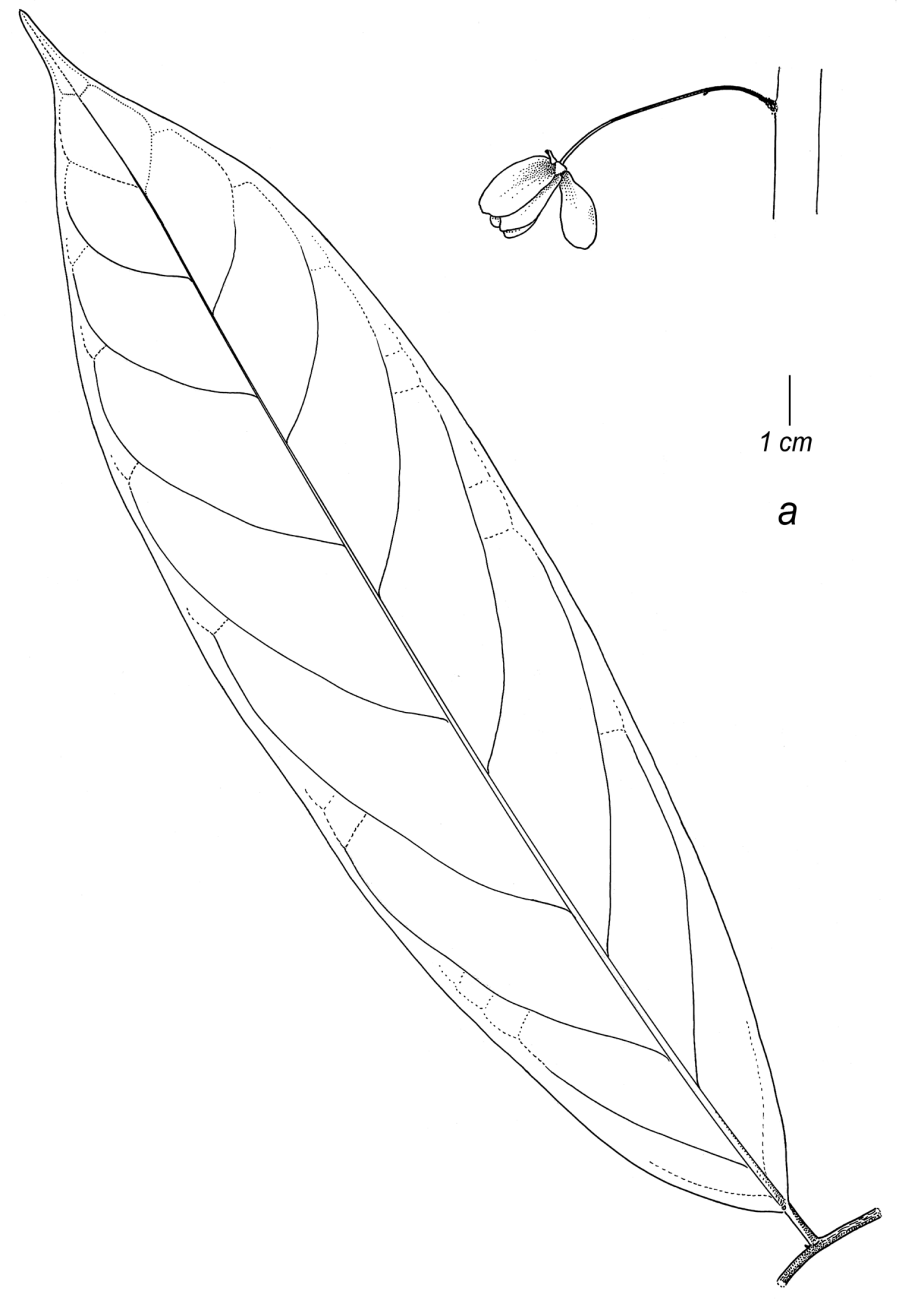
*Cremastospermastenophyllum* Pirie. **a** flower and leaf (*Knapp & Mallet 6159*).

### 32. Cremastosperma venezuelanum

Taxon classificationPlantaeMagnolialesAnnonaceae

Pirie

Fig. 42 , Map 9

Cremastosperma venezuelanum Pirie, Blumea 50: 34, f. 1. 2005.

#### Type.

VENEZUELA, Carababo: Autonomo Mora, Watershed of Río Morón, 3 May 1991, *Diaz, W. & Niño, M. 231* (holotype: U! [U0012254]; isotype: NY! [NY00759136]).

#### Description.

*Tree* 7–10 m tall; young twigs and petioles glabrous. *Leaves*: petioles 10–20 mm long, 3–4 mm diam.; lamina narrowly elliptic, 30–53 by 9–15 cm (index 3–3.6), chartacous to subcoriacous, shiny, dark brown above, olive green/greyish/light to dark brown below, glabrous on both sides, base cordate (rarely rounded), apex obtuse to acute, primary vein 2.5–5 mm wide at the widest point, secondary veins 15–30, intersecondary veins occasional, distance between from 12 mm at the base to 40 mm closer to the apex, angles with primary vein from 90° at the base to 60–50° closer to the apex, rarely branching, forming more or less indistinct loops, smallest distance between loops and margin 2–3 mm; tertiary veins showing some reticulation. *Inflorescence* of single flowers clustered in groups of up to 6, produced from leafless branches or from the main trunk; peduncles ca. 2 by 1 mm (in flower), 3–5 by 1.5–3 mm (in fruit); pedicels ca. 11 by 1 mm at the base (in flower), 16–22 by 1.5–2 mm (in fruit), glabrous; one lower bract, very broadly triangular, 0.5–1 mm long, obtuse, persistent, glabrous; upper bract attachment variable within central 80% of length, ca. 1 by 1.5 mm, depressed triangular, obtuse, glabrous; closed flower buds not seen; flowers dark brown to black *in sicco*, sepals and petals glabrous; sepals free, depressed triangular, reflexed, ca. 1 by 1.5 mm, acute to obtuse, persistent on less mature fruits; outer petals elliptic, ca. 18 by 10 mm, inner petals narrowly elliptic, ca. 21 by 6 mm; androecium ca. 4 mm diam., stamens ca. 1 mm long, connective ca. 0.4 mm wide; gynoecium ca. 1.8 mm diam., carpels 0.6–0.7 mm long, glabrous. *Monocarps* 20–35, ellipsoid, asymmetrical, 17–20 by 12–13 mm, black *in sicco*, with a strongly excentric apicule; stipes 15–22 by 1.5–2 mm; fruiting receptacle 5–12 mm diam.; monocarps, stipes and receptacle glabrous. *Seeds* ellipsoid, orange-brown, shallowly pitted, 15–17 by 13–16 mm, raphe raised, regular.

#### Distribution.

Venezuela (Aragua, Carabobo).

#### Habitat and ecology.

Understorey of primary, moist, evergreen forest. At elevations of 350–1100 m. Fruiting: April and May; flowering: August.

#### Note.

*Cremastospermavenezuelanum* is best distinguished from other species of *Cremastosperma* by its distinctive acute to obtuse leaf apex (as opposed to acuminate or caudate in other species). The combination of cordate (rarely rounded) leaf base, the large angles of the secondary with the primary veins near the base of the leaves and the lack of indument on any parts are also unique. Only one other species of *Cremastosperma* has been collected in Venezuela, *C macrocarpum* Maas, which has longer pedicels and larger monocarps with shorter, thicker stipes.

#### Preliminary conservation status.

*Cremastospermavenezuelanum* is known from only four collections and its EOO and AOO would both qualify as Endangered; however it has been found within the bounds of a national park (Parque Nacional Henri Pittier, Venezuela). Near Threatened [NT] (Table 1).

#### Selected specimens examined.

**VENEZUELA. Aragua**: Parque Nacional Henri Pittier, Maracay-Ocumare, 10°21'N, 67°43'W, 740 m a.s.l., 1 Apr 1990, *Edwards et al. 397* (NY); Parque Nacional Dos Riitos, 10°35'N, 68°00'W, 600 m a.s.l., 19 May 1943, *Killip* & *Lasser 37752* (NY, S). **Carababo**: Río San Gián, S of Borburata, 10°10'N, 68°05'W, 350–500 m a.s.l., 7 Aug 1965, *Steyermark 94314* (NY, US).

**Map 9.**
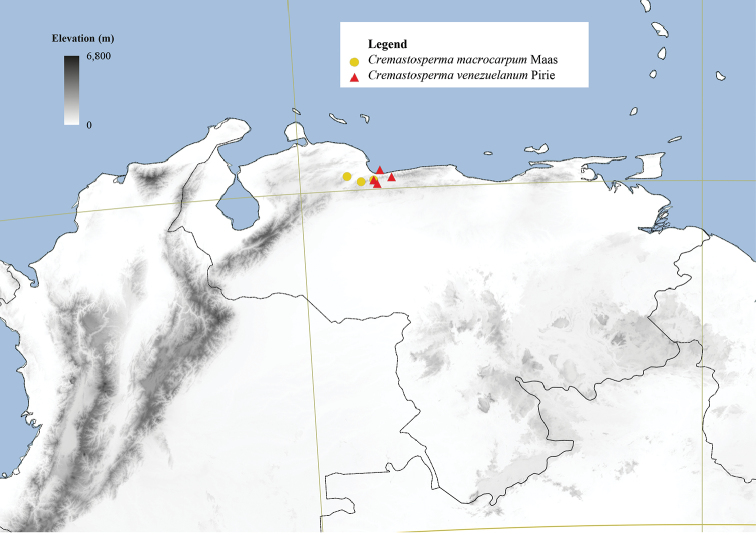
Distribution of *C.macrocarpum* Maas; and *C.venezuelanum* Pirie.

**Figure 42.**
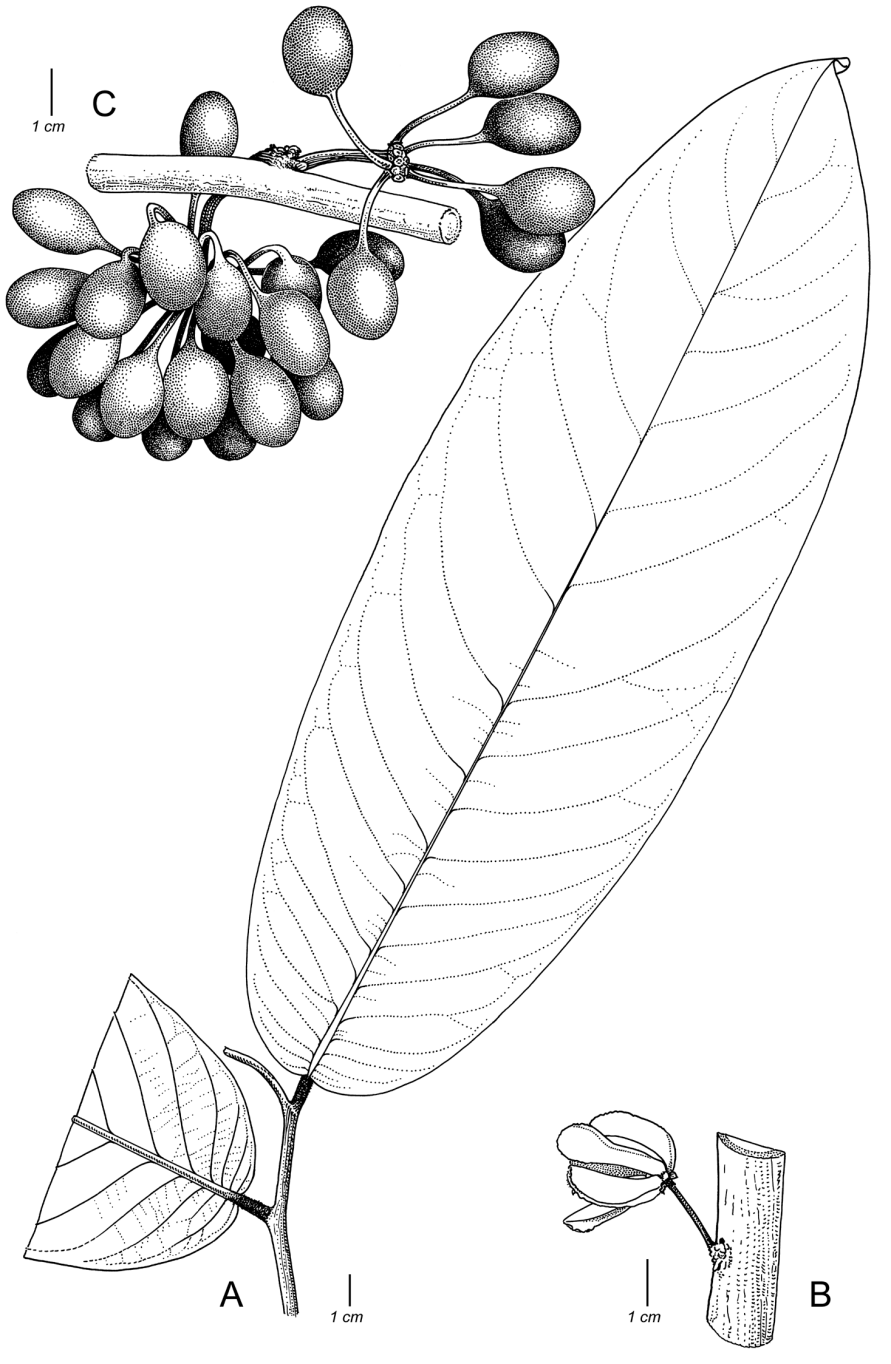
*Cremastospermavenezuelanum* Pirie. **a** leaves **b** flower **c** immature fruit (**a, c***Edwards 397***b***Steyermark 94314*).

### 33. Cremastosperma westrae

Taxon classificationPlantaeMagnolialesAnnonaceae

Pirie

**Figure 43.**
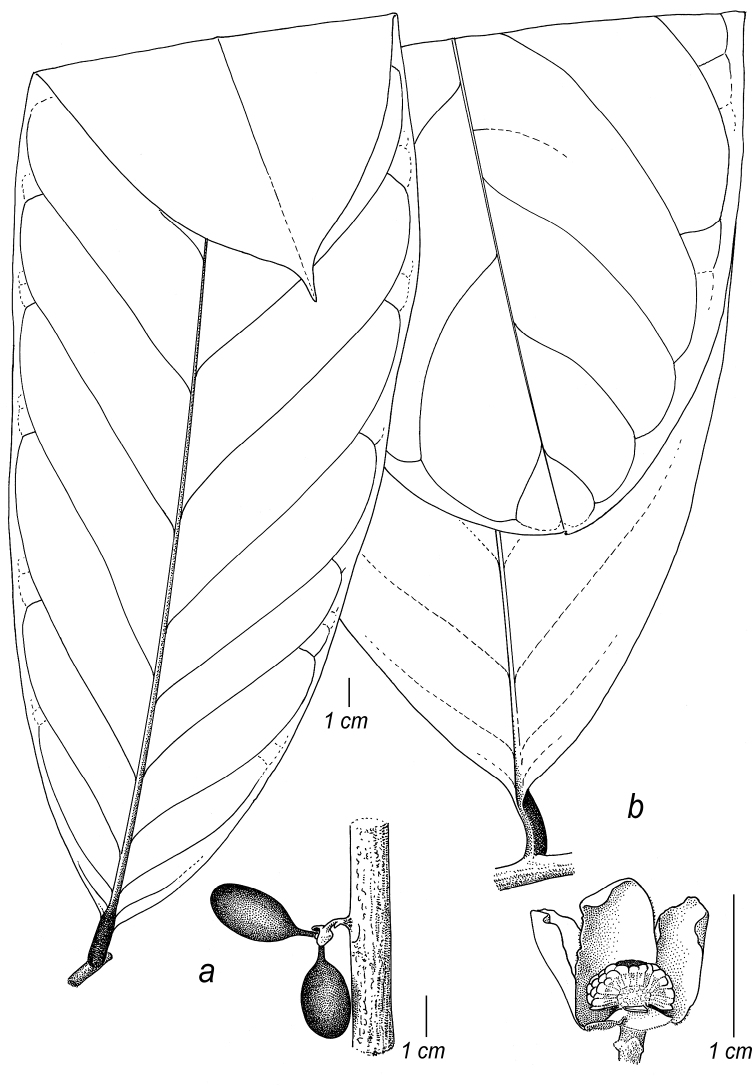
*Cremastospermawestrae* Pirie. **a** fruit and leaf (acuminate apex) **b** flower and leaf (apex broken off) (**a***de Nevers 4475***b***Liesner 682*).

Figs 37 , 43 , Map 3

Cremastosperma westrae Pirie, Blumea 50: 58, f. 9. 2005.

#### Type.

PANAMA, Panamá: road from El Llano to Carti-Tupile, 12 miles above Pan-American Highway, 200–500 m a.s.l., 13 Mar 1973, *Liesner, R.L. 682* (holotype: MO [MO-047498]).

#### Description.

*Tree* or *shrub* 4–8 m tall; young twigs and petioles sparsely to densely covered with appressed brown hairs ca. 0.1 mm long. *Leaves*: petioles 6–20 by 3–6 mm; lamina narrowly elliptic to slightly obovate or narrowly so, (15–)30–50 by (8–)12–20 cm (index 1.6–3), chartaceous to coriaceous, dark to olive green or brown above, lighter below, glabrous above, sparsely covered with appressed whitish hairs 0.1 mm long on veins below, base acute to rounded, apex acuminate (acumen 10–20 mm long), primary vein 2–5 mm wide at widest point, secondary veins 8–12, no intersecondary veins, distance between from 5–10 mm at the base to 40–60 mm closer to the apex, angles with primary vein from 50–80° at the base to 45–60° closer to the apex, not branching, forming more or less distinct loops in apical half, smallest distance between loops and margin 2–5 mm, tertiary veins mostly percurrent. *Inflorescence* of single, solitary flowers, on leafy or leafless twigs; peduncles, 2–3 by 2–3 mm (in fruit); pedicels 6–17 by 2–3 mm (in fruit), peduncles and pedicels rather densely to densely covered with erect whitish hairs 0.1 mm long; single lower bract, soon falling off; upper bract attached in the basal half of the pedicel, depressed ovate, ca. 1.5 by 2 mm, rounded, outer side densely covered with appressed brown hairs 0.2 mm long; closed flower buds depressed ovoid, flowers yellow *in vivo*, black with yellow indument *in sicco*; sepals free or fused for basal 0.5 mm, deltate, appressed, 2.5–3 by 2.5–3 mm, rounded, often persistent, densely covered (outside, sparsely inside) with appressed brown hairs 0.2 mm long; outer petals elliptic to slightly ovate, ca. 9 by 5 mm, rather densely to densely covered (outside, sparsely inside) with appressed brown hairs ca. 0.2 mm long, inner petals elliptic, ca. 8 by 4 mm, rather densely to densely (towards the apex outside, sparsely inside and at base) covered with appressed brown hairs 0.2 mm long; androecium ca. 6 mm diam., stamens ca. 1.3 mm long, connective appendage ca. 0.7 mm wide; gynoecium ca. 1 mm diam., carpels sparsely covered with erect brown hairs <0.1 mm long. *Monocarps* 6–10, ellipsoid, slightly asymmetrical, 18–22 by 10–12 mm, green, maturing to yellow, orange, red or black *in vivo*, reddish- to blackish-brown *in sicco*, with a small excentric apicule; stipes 4–14 by 1–2 mm; fruiting receptacle depressed ovoid, 3–6 mm diam.; monocarps and stipes sparsely to rather densely covered with erect whitish hairs <0.1 mm long or glabrous, receptacle densely covered with erect whitish hairs <0.1 mm long. *Seeds* ellipsoid, reddish-brown, surface wrinkled and slightly pitted, ca. 16 by 10 mm, raphe slightly sunken, encircling seed longitudinally.

#### Distribution.

Panama (Darién, Panamá, San Blas).

#### Habitat and ecology.

Primary seasonal evergreen forest on red clay. At elevations of 50–600 m. Flowering: March; fruiting: January, March, July, November and December.

#### Notes.

*Cremastospermawestrae* is most similar to *C.novogranatense*: it differs in having longer stipes, smaller sepals and less dense, shorter hairs on the petals. The shape of the fruits of *C.pacificum* bears a resemblance to those of *C.westrae*. A clear distinction can be made due to the presence of indument on flowers and fruits in *C.westrae*: those of *C.pacificum* are glabrous.

#### Preliminary conservation status.

*Cremastospermawestrae* is known from just six localities and although these are similar, both in restricted extent and lack of protection to those of *C.panamense*, it is apparently considerably rarer. Endangered [EN] (Table 1).

#### Selected specimens examined.

**PANAMA. Darien**: Camp Summit, adjacent Darién-San Blas border, 1000–1200 m a.s.l., 18 Dec 1967, *Oliver et al. 3681* (MO). **San Blas**: Cordillera de San Blas, 9°13'N, 78°16'W, 50 m a.s.l., 8 Nov 1991, *H. Herrera et al. 1122* (U); Trail along Continental Divide, 9°20'N, 78°56'W, 400 m a.s.l., 23 Jul 1986, *McDonagh et al. 291* (MO); Continental Divide trail W of El Llano-Cartí road, 9°19'N, 78°55'W, 350 m a.s.l., 9 Jan 1985, *De Nevers* & *H. Herrera 4475* (U); Aila Tiwar, Río Acla, 8°48'N, 77°40'W, 25–100 m a.s.l., 23 Mar 1979, *Sugden 613* (MO).

### 34. Cremastosperma yamayakatense

Taxon classificationPlantaeMagnolialesAnnonaceae

Pirie

**Figure 44.**
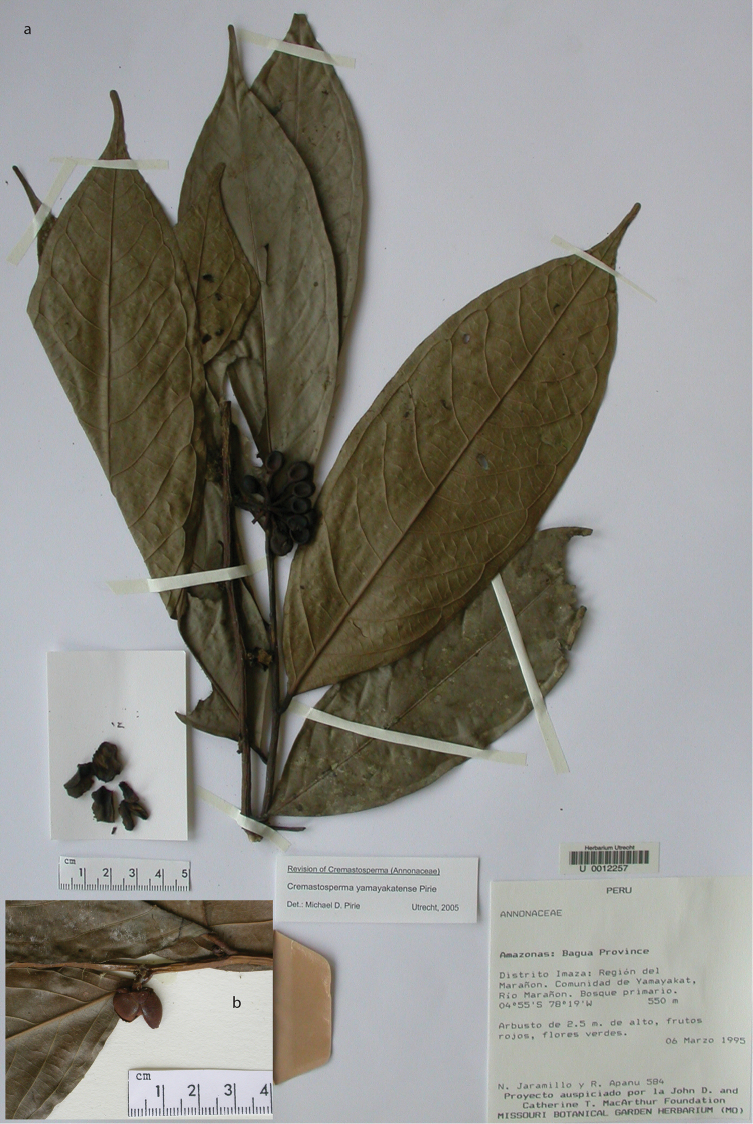
*Cremastospermayamayakatense* Pirie. **a** fruiting specimen **b** flower (**a** Jaramillo & Apanu 584 **b** Barbour 4432).

Figs 37 , 44 , Map 8

Cremastosperma yamayakatense Pirie, Arnaldoa 11: 10, f. 2, 6–8. 2004.

#### Type.

PERU, Amazonas: Bagua, Distr. Imaza, community Yamayakat, trail to Putuim, 22 Nov 2003, *Pirie, M.D. et al. 57* (holotype: U! [U0121237]; isotypes: CUZ!, HAO!, HUT!, K! [K000580473], MO! [MO-1459049], NY! [NY006890], USM!).

#### Description.

*Tree* 1.5–8 (–20) m tall; young twigs and petioles glabrous. *Leaves*: petioles 5–10 by 1–5 mm; lamina narrowly elliptic to elliptic, 11–24 (–38) by 3.5–8 (–13) cm (index 2.4–3.4), chartaceous, olive-grey green above, light brown below, glabrous on both sides, base acute, apex acuminate (acumen 10–25 mm long), primary vein grooved in basal quarter to third, 1–4 mm wide at widest point, secondary veins 8–10 (–14), intersecondary veins occasional, distance between from 5–10 mm at the base to 10–30 mm closer to the apex, angles with primary vein from 70–80°, the angle thereafter decreasing and subsequently increasing again towards the leaf margin, not branching, forming distinct loops, smallest distance between loops and margin 2–6 mm, tertiary veins largely percurrent with some reticulation. *Inflorescences* of single, successively produced, flowers, axillary on leafy branches and on older (leafless) branches (then on brachyblasts); peduncles ca. 1 by 1 mm (in flower), 1–3 by 2–2.5 mm (in fruit), sparsely covered with golden hairs ca. 0.1 mm long; pedicels 5–7 by ca. 1.5 mm at the base (in flower), 8–15(–20) by 2–2.5 mm at the base to 4 mm at the apex (in fruit), glabrous; single lower bract, deltate, 1–2 by 1–2 mm, acute, mostly soon falling off in fruit, rather densely covered with golden hairs 0.1 mm long; upper bract inserted within basal half of pedicel, deltate, 1–2 by 1–2 mm, acute, glabrous; closed flower buds depressed ovoid, remaining closed in development; flowers green maturing to yellow *in vivo*, black *in sicco*, sepals and petals glabrous; sepals basally connate, deltate, appressed, ca. 3 by 3 mm, rounded, soon falling off, rarely persistent; outer petals ovate, 10–15 by 8–12 mm, inner petals elliptic, ca. 12 by 6 mm; androecium 6–7 mm diam., stamens ca. 1 mm long, connective appendage ca. 0.5 mm wide; gynoecium ca. 3 mm diam., carpels length and indument unknown. *Monocarps* 10–22, green maturing through red to black *in vivo*, black *in sicco*, ellipsoid, slightly asymmetrical, 12–14 by 7–8 mm, with an excentric apicule; stipes green maturing to red *in vivo*, 11–12 by ca. 1.5 mm increasing to 3 mm when ripe; fruiting receptacle 5–10 mm diam., monocarps and stipes glabrous or sparsely covered with golden hairs <0.1 mm long, receptacle glabrous. *Seeds* ellipsoid, reddish-brown with small black pits surrounded by a slightly raised rim, 9–13 by 6–7 mm, raphe sunken, regular.

#### Distribution.

Peru (Amazonas), watershed of the upper Río Marañon.

#### Habitat and ecology.

Primary and secondary forest. At elevations of 200–1000 m. Flowering: November, January-March; fruiting: throughout the year except December and April.

#### Vernacular names.

Peru: ciwánim yaáu (*Kayap 33*), Cuicui yais (*Berlin 1588*), Washi yais (Huambisa; *Tunqui 573*), Yais (*R. Vásquez et al. 24797*).

#### Notes.

*Cremastospermayamayakatense* resembles two other species of *Cremastosperma*; *C.gracilipes*, which has been collected in the departments of Napo and Pastaza in Ecuador, Loreto in Peru and in adjacent Colombia and *C.cenepense*, from the Cenepa region of Amazonas, Peru, with which its distribution therefore overlaps. The most important differences between *C.yamayakatense* and *C.gracilipes* are in the flowers. *C.gracilipes* is characterised by flower buds which open during development and which bear indument on all parts. In contrast, the flower buds of *C.yamayakatense* bear virtually no indument and appear to remain closed throughout development, the petals only opening slightly when the flowers are mature. Additionally, the flowers of *C.gracilipes* are borne on longer, more slender pedicels than those of *C.yamayakatense*. *C.yamayakatense* differs from *C.cenepense* in the shape of the leaf base (acute in *C.yamayakatense*, cordate to subcordate in *C.cenepense*) and the length of the stipes (longer than the monocarps in *C.yamayakatense*, shorter than the monocarps in *C.cenepense*). The lack of flowering material of *C.cenepense* makes further distinction currently impossible.

Flowering and fruiting specimens of *C.yamayakatense* of around 1.5 m tall were observed in the province of Bagua, though specimens collected both in this area and particularly those collected further north into the province of Condorcanqui, in the area of the Río Cenepa, have been recorded as reaching heights of 6–8 m and in one case 20 m tall. Differences between collections from these two regions have been observed: the leaves of Condorcanqui specimens are generally larger and the fruits have a slight indument whereas those of the Bagua collections are glabrous. In the absence of floral material from the Cenepa region, it is assumed that these specimens do represent the same species due to the short pedicel, leaf base shape (which excludes the possibility of their representing specimens of *C.cenepense*) and leaf venation.

#### Preliminary conservation status.

*Cremastospermayamayakatense* is restricted in its extent and not found within protected areas. Vulnerable [VU] (Table 1).

#### Selected specimens examined.

**PERU. Amazonas**: Río Cenepa, Huampami, 4°30'S, 78°30'W, 200–250 m a.s.l., 7 Aug 1978, *Ancuash 1324* (U); Bagua, Imaza, 4°45'S, 78°30'W, 750–1000 m a.s.l., 22 Sep 1997, *Chávez 70* (U); Bagua, Yamayakat, 4°55'S, 78°19'W, Jan 1995, *Hodges* & *Gorham 111* (HUT); Bagua, Yamayakat, trail to Putuim, 5°02'55"S, 78°21'06"W, 356–420 m a.s.l., 23 Nov 2003, *Pirie et al. 80* (U, USM); Río Santiago valley, Quebrada Caterpiza, 3°50'S, 77°40'W, 200 m a.s.l., 12 Jan 1980, *Tunqui 573* (MO); Bagua, Aramango, 5°29'54"S, 78°20'00"W, 1650 m a.s.l., 17 Dec 2001, *Vásquez et al. 27434* (U).

**Dubious species**

*Guatteriasocialis* J.F.Macbr., Publ. Field Columbian Mus., Bot. Ser. 4: 171. 1929.

**Type.** PERU, Junín: Chanchamayo Valley, 1500 m a.s.l., Oct 1924–1927., *C. Schunke 395* (holotype: F, isotype: S! [S08-16252]).

The type specimen of *G.socialis* represents a species of *Cremastosperma*, but to which species it might belong is not clear.

**Excluded species**

*Cremastospermaanomalum* R.E.Fr., Kongl. Svenska Vetenskapsakad. Handl. 24: 4, pl. 1c-d. 1948.

**Type.** COLOMBIA, Chocó: Bahia Solano, near Ciudad Mutis, along Quebrada Jella, 0–75 m a.s.l., 21–23 Feb 1939, *Killip, E.P. & Garcia, H. 33600* (holotype: S; isotypes: COL, UC, US).

≡ **Klarobelia anomala** (R.E.Fr.) Chatrou (1998) 123, f. 2.

*Cremastospermaguianense* R.E.Fr., Acta Horti Bergiani 12: 205. 1934.

**Type.** GUYANA, Apoteri: Rupununi River, 21 Jul 1931, Forest Dep. Brit. Guiana 2093 = *Davis, T.A.W. 102* (holotype: K).

≡ **Pseudoxandralucida** R.E.Fr., Acta Horti Bergiani 12: 230, f. 3a-e. 1937:

*Cremastospermapolyphlebum* (Diels) R.E.Fr., Acta Horti Bergiani 10: 331. 1931.

**Type.** BRAZIL, Acre: Rio Jurua-Mirim, Aug 1901, *Ule, E. 5628* (holotype: B; isotypes: F, G, K! [K000485701], MG, S! [S-R-7018]).

≡ **Pseudoxandrapolyphleba** (Diels) R.E.Fr., Acta Horti Bergiani 12: 230. 1937.

*Cremastospermawilliamsii* R.E.Fr., Acta Horti Bergiani 12: 206. 1934.

**Type.** PERU, Loreto: Yurimaguas, Recreo, 23 Oct 1929, *Williams, Ll. 3960* (holotype: F! [V0040605F]; isotype: S [S-R-7031]).

≡ **Pseudoxandrawilliamsii** (R.E.Fr.) R.E.Fr., Acta Horti Bergiani 12: 227, f. 2b,c. 1937.

## Supplementary Material

XML Treatment for Cremastosperma

XML Treatment for Cremastosperma alticola

XML Treatment for Cremastosperma antioquense

XML Treatment for Cremastosperma awaense

XML Treatment for Cremastosperma brachypodum

XML Treatment for Cremastosperma brevipes

XML Treatment for Cremastosperma bullatum

XML Treatment for Cremastosperma cauliflorum

XML Treatment for Cremastosperma cenepense

XML Treatment for Cremastosperma chococola

XML Treatment for Cremastosperma confusum

XML Treatment for Cremastosperma dolichocarpum

XML Treatment for Cremastosperma dolichopodum

XML Treatment for Cremastosperma gracilipes

XML Treatment for Cremastosperma leiophyllum

XML Treatment for Cremastosperma longicuspe

XML Treatment for Cremastosperma longipes

XML Treatment for Cremastosperma macrocarpum

XML Treatment for Cremastosperma magdalenae

XML Treatment for Cremastosperma megalophyllum

XML Treatment for Cremastosperma microcarpum

XML Treatment for Cremastosperma monospermum

XML Treatment for Cremastosperma napoense

XML Treatment for Cremastosperma novogranatense

XML Treatment for Cremastosperma oblongum

XML Treatment for Cremastosperma osicola

XML Treatment for Cremastosperma pacificum

XML Treatment for Cremastosperma panamense

XML Treatment for Cremastosperma pedunculatum

XML Treatment for Cremastosperma pendulum

XML Treatment for Cremastosperma peruvianum

XML Treatment for Cremastosperma stenophyllum

XML Treatment for Cremastosperma venezuelanum

XML Treatment for Cremastosperma westrae

XML Treatment for Cremastosperma yamayakatense

## Appendix 1. Identification list

Collections are identified by the first collector and collector number only. The abbreviations behind the collector numbers refer to the following taxa:

*
Cremastosperma
*

alt *alticola*

ant *antioquense*

awa *awaense*

bra *brachypodum*

bre *brevipes*

bul *bullatum*

cau *cauliflorum*

cen *cenepense*

cho *chococola*

con *confusum*

dol *dolichocarpum*

dop *dolichopodum*

gra *gracilipes*

lei *leiophyllum*

lon *longicuspe*

lop *longipes*

mac *macrocarpum*

mag *magdalenae*

meg *megalophyllum*

mic *microcarpum*

mon *monospermum*

nap *napoense*

nov *novogranatense*

obl *oblongum*

osi *osicola*

pac *pacificum*

pan *panamense*

ped *pedunculatum*

pen *pendulum*

per *peruvianum*

ste *stenophyllum*

ven *venezuelanum*

wes *westrae*

yam *yamayakatense*

Acevedo-Rodriguez 7512: meg; 7523: meg; 7543: gra; 8635: con; 9132 con – Acosta Solís 6429: ste – Aguilar 467: osi; 691: mon; 764: mon; 905: con?; 4371: osi – Aguinda 202: meg; 458: meg; 1581: nap – Alcorn 11: lon – Alvarado 228: nap; 267: nap; 298: meg; 427: nap – Alvarez 2405: gra – Ancuash 262: per; 1324: yam; 1517: per – Arrazola 134: lei – Aulestia, M. 637: awa; 842: awa; 1184: awa; 1513: gra; 1726: gra; 2072: lon; 2127: gra; 3238: cau; 3395: cau: 3520: gra – Ayala 15: cau; 2432: meg; 3485: mic – Ayerbe 130: obl.

Balslev 2418: gra; 4634: gra; 62310: gra – Barbour 4432: yam; 4851: con; 4856: con; 5111: con – Barclay 4755: gra – Barrier 946: meg; 5166: bre – Beck 1675: lei; 8234: mon; 10092: mon; 12221: mon; 16540: mon; 19323: mon – Beltran 3245: con; 3580: obl; 5488: meg; 5782: mic – Berlin 218: per; 1588: yam; 1950: per; 2079: per – Betancur B. 3309 (changed on label to 33011): dol?; 6043: awa – Boom 5039: mon; 10812: bre – Bourdy GB1743: mon; GB1828: mon – Brandbyge 30017: meg; 30472: gra; 31854: cau; 32225: cau; 32566: meg; 32589: gra; 32681: gra; 32685: gra; 33337: gra; 33434: gra; 33503: cau – Buchtien 705: lei; 706: lei – Bulnes 502: obl.

Callejas P. 3110: dol – Camp E1311: ped – Campbell 9026: mon – Campos 4160: ped; 4213: ped; 4268: ped – Cárdenas L. 2899: mag – Cazalet 7528: cau – Cerón 404: meg; 612: meg; 702: gra; 1268: meg; 1353: gra; 2321: gra; 2361: gra; 2378: meg; 2428: gra; 2483: meg; 2500: meg; 2675: gra; 2986: nap; 3009: gra; 3031: meg; 3036: meg; 3037: gra; 3125: meg; 3939: gra; 3949: gra; 4159: meg; 5358: gra; 5436: gra; 5983: gra; 6663: nap; 9324: meg; 14060: sp; 20815: nap – Chatrou 103: osi; 208: mic; 224: cau; 227: mic; 233: cau; 238: cau; 259: meg; 265: meg; 267: gra; 268: meg; 707: osi – Chávez 70: yam – Cheta 6/173: cau – Cid Ferreira 2724: mon; 2868: mon; 4507: mon; 4556: mon; 4590: mon; 4673: mon; 5262: cau; 6301: mon; 9959: meg; 10019: cau; 10120A: mon; 10286: mon – CID-JH 6-235: cau – Clark 3158: meg; 10186: gra – Coello 60: gra – Cogollo P. 6039: dol; 6040: dol; 6044: dol; 6052: dol; 6056: dol; 6345: dol; 7869: dol – Cornejo, F. 2276: mon; 2546 con? – Cornejo, S.X. 4493: gra – Cornejo, X. 6451: awa; 6702: ped; 9000: ped – Couvreur 130: meg; 159: con; 262: con; 267: con – Cremers 15331: bre – Croat 18494: cau; 90749: mic – Cuatrecasas 10575: meg; 10726: cau; 11125: cau; 11331: meg; 17463: pac; 17573: nov; 21288: nov; 26031: nov.

Dahlgren 162: mon – Daly 5764: mic; 6186: meg; 6498: mon; 6573: mon; 6630: mon; 6686: mon; 6909: mon; 7347: mon; 7450: mon; 8043: mon; 9203: mon; 11719: mon – Davidse 23634: pan – Davidson 9869: mic – Dávila 1958: cau; 2784: cau; 2848: cau – Del Carpio 1833: mic; 1923: mic; 1942: mic; 2213: mic; 8009: mon – Devia 2875: nov?; 3762: pac; 4065: pac; 5335: nov – De Nevers 3601: pan; 3906: pan; 4129: pan; 4475: wes; 4571: pan; 4898: pan; 5189: pan; 5221: pan; 5260: pan; 6171: pan; 6513: pan – DeWalt 662: mon – Díaz, S. 825: mon; 1229: cau; 1411: gra; 6851: yam; 8225: per; 8375: yam; 8899: mon; 9517: mon; 9563: mon – Díaz, W. 231: ven; 274: mac – Dik 323: meg; 1732: gra – Dominguez 52: mic; 59: mic –Ducke RB19620: meg – Durand 199: obl – Dwyer 3314: pan; 10248: pan – Dziedzioch 21-161098: meg.

Edwards 397: ven – Ek 1615: bre – Escobar 3309: mag – Espina Z. 3188: cho – Espinoza 129: meg; 399: cau; 578: gra.

Faber-Langendoen 476: nov; 1208: pac – Fernández 8872: lop – Feuillet 10239: bre; 10337: bre – Figuieredo 853: mon; 906: mon – Flora Falcón 937 (= Van der Werff 937): mac – Flores 165: obl: 1241: obl – Folsom 6155: pan – Forero 6576: lop – Forero 6709: sp – Forget 305: bre – Foster, P.F. 230: lei – Foster, R. 2477: con?; 2512: con; 3105: con; 3128: con; 3157: con; 3418: con?; 3502: con?; 5004: con?; 5083: con; 5292: con?; 6026: con?; 6155: con?; 6589: con; 6612: con; 8235: con?; 9482: pen; 10008: pen; 10224: obl; 11495: con; 12549: mon; 13422: mon – Freire 407: meg; 468: nap – Freitas 8: mic – Fróes 23962: cau – Funk 8304: con?.

Galdames 1162A: pan; 1585: pan – García 390: cho – Gentry 5070: pan; 7286: cho; 8828: pan; 8910: mon; 21928: gra; 25111: mic; 29126: cau; 31447: cau; 32009: con; 32153: mic; 35298: pac; 35562: pac; 37169: cau; 38085: mic; 38127: mic; 41879: sp.; 42938: sp.; 42985: mic; 43174: mic; 43776: mic; 45355: ped; 45371: ped; 45481: ped; 45510: ped; 52233: mon; 56210: mic; 57078: nov; 58434: mon; 60032: gra; 60865: cau; 65601: cau; 68663: mon; 70042: awa; 72190: mic; 72235: cau; 72290: mic; 72995: lop; 76343: cau; 76357: mic; 78657: osi; 79039: dol; 80543: ped; 80904: ped – GHS 703: obl – Gómez-Pompa 3067: pan – Gonzales 53: mon – Graham 635: mon; 1713: mon – Grández 222: mic; 491: mic; 1591: cau; 1960: mic; 2032: mic; 2376: mon; 2488: gra; 2702: mic; 4706: mon – de Granville 390: bre; B5168: bre; B5186: bre; 5670: bre; 5672: bre; 7555: bre; 7672: bre; 7799: bre; 8714: bre; 8876: bre; 10176: bre – Grenand 1509: bre – Grijalva 305: meg – Gudiño 310 (U): meg; 310 (MO): gra; 1123: meg; 1787: gra; 2092: meg.

Harling 17581: gra – Hedin 57: con?; 74: con?; 94: mic; 96: mic – Helme 742: mon – Hequet 432: bre – Hernandez 251: mag – Herrera, G. 3972: osi; Herrera, H. 1122: wes; 1231: pan – Hinojosa 1313: lei – Hodges 111: yam; 112: yam – Holm-Nielsen 903: meg; 21501: cau; 22082: meg Homeier 4056: ped; 4173: ped – Host 6890: mon – Huamantupa 7746: gra; 12928: cau; 15601: per – Huashikat 321: mic; 357: per; 493: mic; 577: per – Hurtado 160: nap; 186: nap; 322: nap; 896: nap; 941: nap; 1385: meg; 2096: nap; 2430: meg; 2445: meg; 2448: meg; 3005: meg; 3019: gra; 3156: meg.

Irvine 374: meg; 734: meg; 817: gra – Irwin 47562: bre.

Jacquemin 2349: bre – Jaramillo A., J. 1420: ped; 3533: meg; 3577: gra; 8352: meg; 8364: meg; 12840: nap; 13489: ped; 30773: gra – Jaramillo, N. 403: yam; 584: yam; 686: cau; 918: yam; 942: bul; 972: bul; 1110: yam; 1248: yam; 1409: yam – Jardim 597: mon; 759: mon; 2408: mon – Johnston 1555: pan; 1812: pan – Juncosa 736: mag.

Kajekai: 1740: nap – Kanehira 215: ped? – Kayap 33: yam; 631: per; 1078: cen – Kernan 1224: osi – Killeen 3080: lei; 3085: lei; 3110: lei; 3238: lei; 7640: lei – Killip 23004: con?; 23622: obl; 27902: mon; 28537: lon; 28961: mon; 29020: lon; 37752: ven – Klug 902: cau; 3069: meg; 3726: ped – Knapp 6159: ste; 6354: mon?; 6450: con; 6588: ped; 7178: lon; 7183: lon; 7215: lon; 7645: per; 7864: lon; 8203: lon – Krukoff 4697: mon; 4748: mic; 4975: mic; 6151: mic; 10556: lei – Kuhlmann 1426: lon; 24265: lon – Kvist 892: mic; 1127: mic; 1265: mic; 1355: mic.

Lawesson 39560: gra; 39598: meg – Lescure 549: bre; 2200: meg – Lewis, M. 37979: lei – Lewis, W.H. 11166: lon; 11831: lon; 12049: cau; 12186: lon – Liesner 682: wes; 1362: pan; 9763: mac – Lima 582: mon – Lleras P16879: cau – Lowrie 463: mon – Luteyn 4890: gra; 8630: gra; 9057: gra.

Maas 4592: obl; 6271: cau; 6281: mic; 8064: bre; 8222: mic; 8289: mic; 8300: mic; 8577: meg; 8595: meg; 9029: cau; 9148: obl; 9251: cau; 9328: bre; 9559: pan; P13079: mon – Martin s.n. bre – Mathias 5510: mic; 6007: mon – McDaniel 2570: pen; 14100: mon; 17020: mic; 20264: mic; 20677: mic; 20761: cau – McDonagh 181: pan; 291: wes – McPherson 9957: pan; 11046: pan; 14039: pan 20670: pan – Meneces 395: lei – Miller 794: meg; 947: pan – Mitchell 26-87: mon – Monsalve 1816: nov – Monteagudo 4933: obl; 11777: obl; 12749: mon; 15649: obl; 15917: obl; 16717: obl; 16728: obl; 16809: obl – Morawetz 13-9888: pen; 110-25985: obl – Moretti 12: bre – Mori 2906: pan; 2918: pan; 5527: pan; 6404: pan; 8758: bre; 15079a: bre; 15583: bre; 22721: bre; 22744: bre – Munzinger 1818: bre – Murillo 565: cau – Murray 1520: pan.

Naessany 106: lei – Nee 34402: mon; 34992: mon; 35033: mon; 38126: lei; 38135: lei; 40971: lei; 41022: lei – Neill 6359: meg; 7195: gra; 7210: meg; 7568: gra; 7649: nap; 7883: meg; 8089: nap; 8466: meg; 9088: nap; 9118: gra; 9180: nap; 9334: lei; 9589: ped; 9967: gra; 10005: meg; 11048: nap; 11065: ped?; 11095: gra; 13201: ped? – Nelson 680: mon; 763: mon – Núñez 10146: con; 10589: mon; 10785: mon; 11068: mon; 11202: mon; 12152: mon; 12446: con; 12951: con; 16303: mon; 16898: obl; 19079: con; 19232: con; 19240: con; 19495: con; 21737: con; 26202: sp.

Oldeman T19: bre; B634: bre; B636: bre; B2165: bre – Oliver 3681: wes – Oliveira 488: mon; 500: mon – Öllgaard 35223: gra; 38866: gra; 99056: gra – Ownbey 2715: nap.

Palacios 783: gra; 1620: meg; 1651: gra; 2212: nap; 2311: meg; 2416: gra; 2492: gra; 2494: meg; 2871: meg; 2937: meg; 2992: gra; 3270: meg; 3276: meg; 4150: nap; 4215: meg; 4261: meg; 4323: meg; 4759: gra; 4902: meg; 5123: meg; 5485: nap; 7043: gra; 7602: cau; 8043: cau; 8646: ped; 8925: meg; 10278: meg; 10462: gra; 10663: meg; 11016: meg; 14890: meg; 16384: ste – Paniagua 2119: sp. – Pariona 983: obl – Pennington 12266: nap – Perea 2540: alt – Pérez 832: pan – Phillips 545: mon – Pinkley 558: meg – Pipoly 12331: mic; 12982: cau; 14155: meg; 16951: dol; 17006: dol; 17073: dol – Pirie 2: lei; 3: lei; 4: mon; 5: mon; 7: obl; 33: pen; 35: pen; 54: pen; 57: yam; 58: yam; 59: yam; 60: yam; 66: bul; 68: bul; 71: bul; 80: yam; 94: bul – Pitman 195: gra; 211: meg – Plowman 2551: mic; 7248: gra – Poeppig 1750: pen; 2091: lon – Prance 3527: mic; 3579: mic; 5354: mon; 7885: mon; 13079 (= Maas P13079): mon; 13464: mic; 23783: cau; 24094: cau – Prévost 2284: bre; 2310: bre; 3446: bre.

Quelal 191: awa – Quipuscoa 2324: lon; 2361: lon – Quizhpe 671: ped; 1088: alt; 1505: meg.

Raffauf 102: cau – Ramirez 4881: ped?; 9517: mon – Revelo 264: meg – Revilla 2265: cau; 3327: mic – Riéra 668: bre; 685: bre; 1564: bre – Rimachi Y. 480: cau; 1027: meg; 2121: meg; 2397: cau; 7588: cau; 7597: obl; 10637: lon – Riós 4491: cau; 4422: gra – Rodriguez 724: yam; 1112: per; 1152: bul; 1758: ped – Rojas 0255: cen; 0269: cen; 0351: cen; 666: mon; 1014: ped; 4128: obl; 5093: obl; 5474: obl; 5758: dop; 5785: pen; 5795: obl; 6485: dop; 6486: dop; 6598: obl; 7683: pen; 7692: obl; 8680: obl – Romero-Castañeda 3369: awa – Romoleroux 1834: gra; 1883: gra; 2267: gra; 3197: meg – Rosa 2426: mon – Rubio 19: meg; 320: gra; 812: cau; 2181: awa; 2397: nap – Rudas 2260: mic; 2267: mic; 4514: cau – Rueda 952: mon – Ruiz, H. 19-28: lei; 19117: pen – Ruíz M., J. 1748: lon? – Ruiz, M. 8712: mic – Ruíz Z., T. 3499: mac.

Sabatier 486: bre; 1486: bre – Saldías 530: lei – Sánchez, D. 323: pac; 415: dol; 9615: ped – Sastre 503: meg; 5670: bre; 5771: bre – Scharf 76: bre; 77: bre; 78: bre – Schatz 1002: osi; 1077: pan – Schunke, C. 200: cau – Schunke V., J. 2554: mon; 3320: mon?; 3828: mon?; 4068: mon?; 5645: obl; 5829: obl; 6412: mic; 7251: ped; 7591: mon?; 8484: mon?; 10907: obl; 12442: mon; 15223: mon; 15227: mon – Scolnik 581: lei – Seidel 2003: lei; 2265: lei; 2584: mon; 2674: mon; 2878: lei; 2893: lei; 3543: lei; 4531: mon; 4660: mon; 7234: lei; 7280: mon; 7290: lei – Silva, M.G. 5398: mon – Silva, M.N. 322: mon; 354: mon – Silveira 767: mon; 1464: mon; 3796: mon; 3811: mon; 4171: mon – Smith, D.N. 2018: obl; 2031: obl; 2385: pen; 4611: ped; 4633: ped; 6613: obl; 6850: obl; 13253: lei; 13834: mon; 14006: lei; 14058: lei; 14148: lei; 14216: mon – Smith, S.F. 137: con?; 504: con; 654: con; 794: con; 1577: con; 1578: con – Soejarto 781: gra; 1275: meg; 2798: ant; 3586: ant – Solomon 3523: mic; 6606: lei – Sperling 5784: mon; 6198: mon – Sprague 351: meg – Stahl 2931: ped – Stein 2577: meg; 3036: gra – Steyermark 94314: ven; 123804: mac – Suárez 1331: cau –Succli 1943: con – Sugden 613: wes – Suin 1941: meg – Sytsma 2884: pan.

Teixeira 480: mon; 520: mon – Tessmann 4176: per; 4748: gra – Thomas 1106: lei; 1517: lei –Timaná 1010: con; 1537: mon; 1563: mon; 1593: mon; 1617: mon; 1632: mon; 1673: mon; 1799: mon; 1822: mon; 1824: mon; 3219: mon – Tipaz 1428: awa; 1718: awa; 1912: awa – Tirado 591: awa; 1083: awa – Tostain 33: bre – Tredwell 22: mic – Tuberquia 1416: ant – Tunqui 573: yam; 999: per – Tyson 3314: pan.

Valencia 67352: meg; 68807: meg – Valenzuela 356: mon; 946: mon; 997: mon; 2205: mon; 3343: con?; 8643: con; 9800: con?; 11838: obl; 12121: obl; 12782: obl; 13014: pen; 13016: pen; 13205: sp.; 15543: obl – Van der Werff 937: mac; 10161: lon; 12045: awa; 13001: ped?; 13022: ped; 13838: cau – Vargas C., C. 1113: lei; 1193: lei; 1194: lei – Vargas, H. 612: meg; 671: meg; 1025: meg – Vásquez 1938: cau; 1969: meg; 2994: meg; 4423: gra; 4559: lon; 6932: mon; 7237: meg; 8010: mic; 8100: mic; 8425: cau; 9350: mic; 10088: mic; 10637: cau; 11076: meg; 11192: meg; 11423: cau; 11438: cau; 12295: mic; 12358: cau; 12655: cau; 12675: gra; 12829: mon; 12862: mic; 13007: gra; 13552: mic; 13690: mic; 16885: meg; 17376: gra; 18236: cau; 18990: per; 19055: yam; 19322: con; 24428: meg?; 24797: yam; 24891: bul; 25605: con; 27434: yam; 28501: mon; 31203: pen; 32541: obl; 33077: obl; 33234: obl; 33463: obl; 34206: pen; 35193: obl; 35950: sp. – Vilca 411: obl – Villa 1499: meg – Villiers 2639: bre – Vriesendorp 428: meg.

Wallnöfer 112-7488: mon; 13477: bre; 13499: bre; 25-19588: mon; 117-28988: obl – Warush Juwa RBAE119: cau – Weberbauer 4558: ped – White 913: lei – Whitmore 717: meg; 737: gra; 854: meg; 871: gra – Williams, Ll. 670: mon; 2197: gra; 4092: lon; 4919: mon; 5085: mon; 5287: mon; 5296: mon; 7423: obl – Wingfield 6751: mac – Wisun 446: bul; 1165: cau – Woytkowski 7128: bra; 7592: meg.

Young, H.J. 155: con; 219: con – Young, K. 193: mon.

Zak 4202: meg; 5272: cau – Zamora 444: meg; 518: meg; 581: gra; 657: meg; 671: meg; 2312: osi – Zárate 3176: lei – Zuleta 175: meg.

## Appendix 2. Index to scientific names

Accepted taxa are in roman type and synonyms in italics. Numbers refer to the species number as used in this revision, other species with a *Cremastosperma* basionym are indicated with “”, nomina dubia by nom.dub.

*Aberemoa pedunculata* Diels 28

*Annona nitida* Ruiz & Pav. 14

Cremastosperma alticola Pirie & Chatrou 1

*anomalum* R.E.Fr. excl.

antioquense Pirie 2

awaense Pirie 3

brachypodum Pirie & Chatrou 4

brevipes (DC.) R.E.Fr. 5

bullatum Pirie 6

cauliflorum R.E.Fr. 7

cenepense Pirie & Zapata 8

chococola Pirie 9

confusum Pirie 10

dolichocarpum Pirie 11

dolichopodum Pirie & Maas 12

gracilipes R.E.Fr. 13

*guianense* R.E.Fr. excl.

*juruense* R.E.Fr. 21

*killipii* R.E.Fr. 15

leiophyllum R.E.Fr. 14

longicuspe R.E.Fr. 15

longipes Pirie 16

macrocarpum Maas 17

magdalenae Pirie 18

megalophyllum R.E.Fr. 19

microcarpum R.E.Fr. 20

monospermum (Rusby) R.E.Fr. 21

*monospermum* var. *brachypodum* R.E.Fr. 21

napoense Pirie 22

novogranatense R.E.Fr. 23

oblongum R.E.Fr. 24

osicola Pirie & Chatrou 25

pacificum R.E.Fr. 26

panamense Maas 27

pedunculatum (Diels) R.E.Fr. 28

pendulum (Ruiz & Pav.) R.E.Fr. 29

peruvianum R.E.Fr. 30

*poiteaui* (Diels) R.E.Fr. 5

*polyphlebum* (Diels) R.E.Fr. excl.

stenophyllum Pirie 31

venezuelanum Pirie 32

westrae Pirie 33

*williamsii* R.E.Fr. excl.

yamayakatense Pirie 34

*Cymbopetalum monospermum* Rusby 21

Klarobelia anomala (R.E.Fr.) Chatrou excl.

*Guatteria brevipes* DC. 5

*leiophylla* Diels 14

*lucida* Rusby 14

*pendula* Ruiz & Pav. 29

*poiteaui* Diels 5

*rusbyi* J.F.Macbr. 14

*socialis* J.F.Macbr. nom.dub.

Pseudoxandra lucida R.E.Fr.

polyphleba (Diels) R.E.Fr.

williamsii (R.E.Fr.) R.E.Fr.

## Supplementary materials

Supplementary material 1 A file incorporating the georeferenced part of this data that can be opened in Google Earth Data type: occurences This is an open access article distributed under the terms of the Creative Commons Attribution License (CC BY 4.0), which permits unrestricted use, distribution, and reproduction in any medium, provided the original author and source are credited.Michael D. Pirie, Lars W. Chatrou, Paul J. M. Maas

Supplementary material 2 Full tables of *Cremastosperma* collections data (CSV, XLS and DBF formats) Explanation note: CSV, XLS and DBF formats Data type: occurences This is an open access article distributed under the terms of the Creative Commons Attribution License (CC BY 4.0), which permits unrestricted use, distribution, and reproduction in any medium, provided the original author and source are credited.Michael D. Pirie, Lars W. Chatrou, Paul J. M. Maas
